# Substrate‐Selective Inhibition of the SARS‐CoV‐2 Papain‐Like Protease: Inhibition of Hydrolysis of Human Over Viral Substrates

**DOI:** 10.1002/chem.71248

**Published:** 2026-06-12

**Authors:** Sakshi Sharma, Peter A. C. Wing, Wojtek Treyde, Shyam Basak, Simeon D. Draganov, Taylah Andrews‐Clark, Eidarus Salah, Petra Lukacik, Claire Strain‐Damerell, Adán Pinto‐Fernández, Martin A. Walsh, Fernanda Duarte, Christopher J. Schofield, Lennart Brewitz

**Affiliations:** ^1^ Chemistry Research Laboratory and the Ineos Oxford Institute for Antimicrobial Research University of Oxford Oxford UK; ^2^ Centre for Medicines Discovery, Nuffield Department of Medicine University of Oxford Oxford UK; ^3^ Chinese Academy of Medical Sciences Oxford Institute University of Oxford Oxford UK; ^4^ Harwell Science and Innovation Campus Diamond Light Source Ltd. Didcot UK; ^5^ Harwell Science and Innovation Campus Research Complex at Harwell Didcot UK; ^6^ Centre for Chemical Biology, Institute of Physical Chemistry Polish Academy of Sciences Warsaw Poland

**Keywords:** deubiquitinase / DUB, interferon‐stimulated gene 15 / ISG15, mass spectrometry, protease inhibition, SARS‐CoV‐2 main protease / M^pro^, SARS‐CoV‐2 papain‐like protease / PL^pro^, substrate‐selective inhibition, ubiquitin, virus‐host interactions

## Abstract

The SARS‐CoV‐2 papain‐like protease (PL^pro^) and the main protease (M^pro^) catalyze hydrolysis of the viral polyproteins pp1a/1ab into functional nonstructural proteins. PL^pro^ and M^pro^ are medicinal chemistry targets, with M^pro^ inhibitors being used for COVID‐19 treatment. PL^pro^ also catalyzes hydrolysis of ubiquitin and interferon‐stimulated gene 15 (ISG15) from post‐translationally modified human proteins. Here we report how screening of reported deubiquitinase inhibitors using solid‐phase extraction coupled to mass spectrometry assays with oligopeptide substrates based on pp1a/1ab and on an ISG15‐modified human protein enabled the identification of substrate‐selective PL^pro^ inhibitors. The results reveal that the deubiquitinase inhibitor ML364 selectively inhibits the deISGylase activity of isolated PL^pro^ over its pp1a/1ab‐processing activity. Structure‐activity relationship and computational studies support the assignment of ML364 and derivatives as substrate‐selective PL^pro^ inhibitors. The combined results provide proof‐of‐concept for developing substrate‐selective inhibitors of PL^pro^ and, by implication, related proteolytic enzymes, including deubiquitinases.

## Graphical abstract

The SARS‐CoV‐2 papain‐like protease (PL^pro^) is a medicinal chemistry target. Here we report mass spectrometry assays employing oligopeptide substrates based on the sequences of the viral polyproteins 1a/1ab and on an ISG15‐modified human protein, which enabled the identification of substrate‐selective PL^pro^ inhibitors. The deubiquitinase inhibitor ML364 selectively inhibits the deISGylase activity of isolated PL^pro^ over its pp1a/1ab‐processing activity.

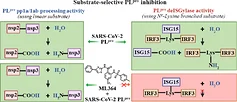

## 1 Introduction

The SARS‐CoV‐2 genome encodes the viral polyproteins 1a/1ab (pp1a/1ab), which are catalytically hydrolyzed into functional nonstructural proteins by two viral nucleophilic cysteine proteases*—*the papain‐like protease (PL^pro^) and the main protease (M^pro^) [1, 2, 3]. Proteolytic processing of pp1a/1ab is essential for viral replication [1, 2, 3]; thus, both PL^pro^ and M^pro^ are targets for small‐molecule inhibitors [4, 5, 6, 7, 8, 9], including for coronaviruses other than SARS‐CoV‐2. Inhibitors of M^pro^, but not yet PL^pro^, are currently in clinical use [10, 11, 12, 13, 14].

**FIGURE 1:**
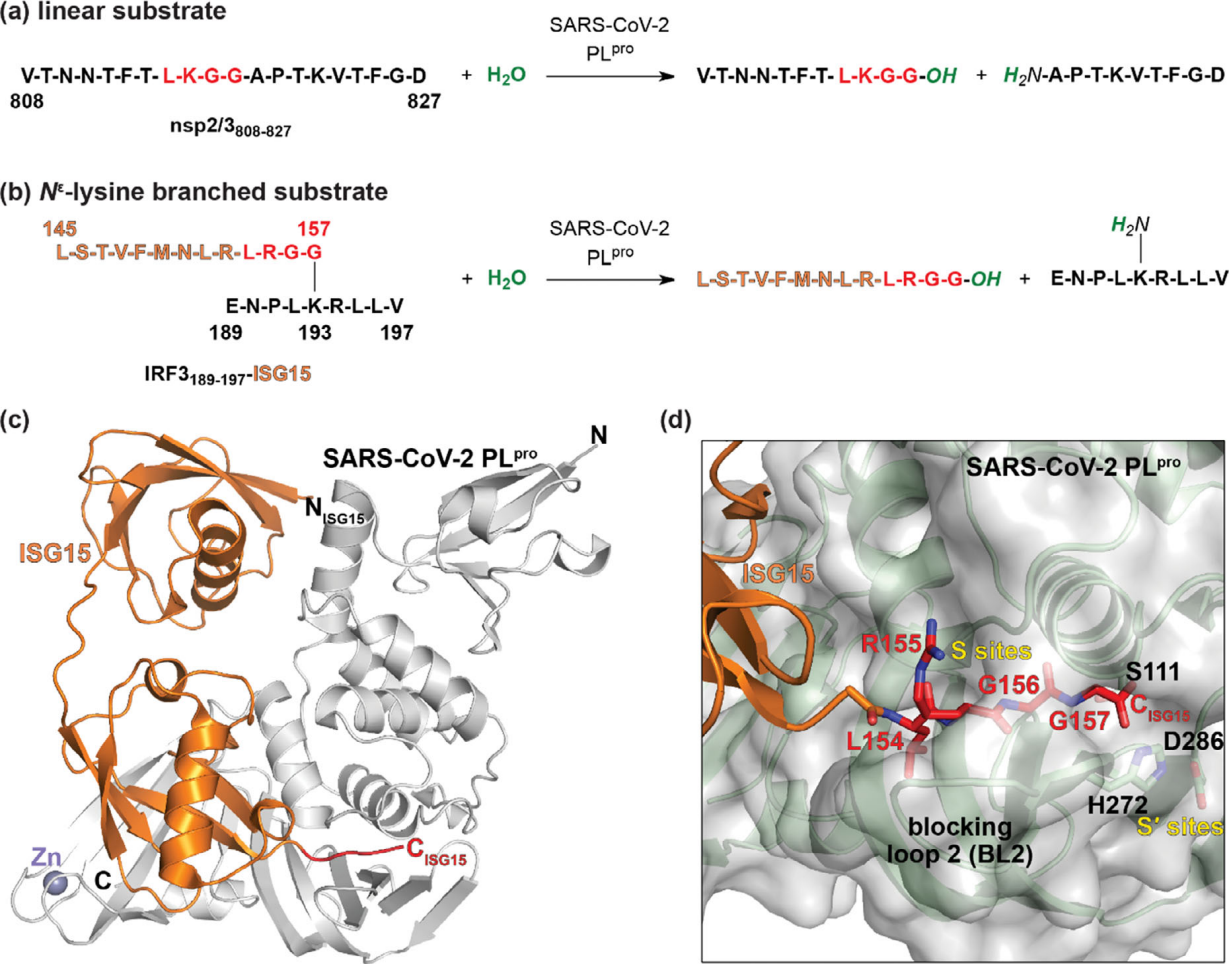
**PL^pro^ catalysis and substrates used in this study**. (**a** and **b**) SARS‐CoV‐2 PL^pro^‐catalysed hydrolysis of: (**a**) the pp1a/1ab‐derived linear oligopeptide **nsp2/3_808‐827_** [33] and (**b**) the *N* ^ε^‐Lys_193_‐branched IRF3_189‐197_‐ISG15 oligopeptide **IRF3‐ISG15** [34], which serve as SPE‐MS assay substrates. The consensus LXGG motif preferred for PL^pro^ catalysis and present in both substrates is in red; (**c**) view from a reported SARS‐CoV‐2 PL^pro^ C111S variant:Zn:ISG15 crystal structure (PDB ID: 7RBS [30]); (**d**) active site view from a reported SARS‐CoV‐2 PL^pro^ C111S variant:Zn:ISG15 crystal structure (PDB ID: 7RBS [30]) showing binding of the C‐terminal LRGG motif of ISG15 in the PL^pro^ S sites near the catalytic triad residues (C111, H272, D286) [3, 35]; note that the binding of substrate residues to the C‐terminal side of the scissile substrate amide bond in the PL^pro^ S′ sites has not yet been crystallographically resolved.

PL^pro^ catalyzes peptide bond hydrolysis of pp1a/1ab on the C‐terminal side of three LXGG motifs (with X being N or K; Figure 1) [3, 15]. PL^pro^ also catalyzes the hydrolysis of isopeptide bonds linking the C‐terminal LRGG motifs of ubiquitin (Ub) or ubiquitin‐like modifiers (UBL) and the *N* ^ε^‐amino groups of lysine residues [16, 17]. Post‐translational protein modification by Ub or UBL has multiple regulatory functions and is linked to diseases, including cancer [18, 19, 20, 21, 22]. Ub/UBL‐protein modifications are directly reversed by deubiquitinases (DUBs), which show varied selectivities for particular Ub/UBL‐modified proteins and/or the Ub/UBL modification [18, 21, 23, 24]. Human DUBs are medicinal chemistry targets, with small‐molecule DUB inhibitors currently in clinical trials [19, 20, 25].

SARS‐CoV‐2 PL^pro^ prefers human protein substrates which are post‐translationally modified with interferon‐stimulated gene 15 (ISG15) over those modified with Ub [16, 17]. PL^pro^‐catalyzed removal of ISG15 from ISGylated interferon regulatory factor 3 (IRF3) enhances the virulence of SARS‐CoV‐2 [16, 26, 27, 28, 29]. ISG15 binds to the PL^pro^ S sites (i.e., those equivalent to the N‐terminal side of the PL^pro^ scissile pp1a/1ab bonds) and allosteric sites [16, 17, 30], while the protein bearing the *N* ^ε^‐lysine‐ISGylation modification binds, at least, to the PL^pro^ S′ sites (i.e., those equivalent to the C‐terminal side of the PL^pro^ scissile pp1a/1ab bonds).

Small‐molecules that selectively block the PL^pro^ deISGylase activity over its pp1a/1ab‐processing activity are of interest as probes for investigating the roles of the PL^pro^ deISGylase activity in SARS‐CoV‐2 pathogenesis. The development of substrate‐selective inhibitors of proteases and other enzyme classes is of general interest from a medicinal chemistry perspective [31], but is challenging in the case of PL^pro^, in part because robust biochemical assays that clearly distinguish between the deISGylase and pp1a/1ab‐processing activities of isolated PL^pro^ have not been reported. Most reported PL^pro^ inhibitors bind to the S1‐S4 pockets, hence likely inhibit both deISGylase and pp1a/1ab‐processing PL^pro^ activities indiscriminately (Figure 1) [6, 7, 8, 9]; note that some PL^pro^ inhibitors have been shown to additionally bind at the S′ sites [32].

Many reported SARS‐CoV‐2 PL^pro^ deISGylase assays monitor hydrolysis of fluorescent groups (e.g., 7‐amino‐4‐methylcoumarin, AMC) conjugated to the C‐terminus of ISG15 derivatives (e.g., ISG15‐AMC) [16, 17, 26, 36, 37]. Such fluorophores, however, likely interfere with optimal binding to the PL^pro^ S′ sites, limiting the ability of these assays to differentiate between deISGylase and pp1a/1ab‐processing activities. Fluorescence‐based PL^pro^ assays are reported that use substrates binding to the S′ sites [38, 39], including assays monitoring the deISGylase/DUB activity by employing Ub derivatives C‐terminally linked via an isopeptide bond to a peptide fragment [40]; these substrates, however, likely suffer from suboptimal active site binding, including because of the sterically bulky fluorophore proximal to the S′ sites [38, 40]. Mass spectrometry (MS)‐based assays have also been employed to monitor PL^pro^ DUB activity; however, the reported assays employ Ub_2_ and derivatives as substrates which are not the preferred cellular PL^pro^ substrates [30, 41, 42].

We have developed solid‐phase extraction (SPE) coupled to MS assays that directly monitor PL^pro^ (or M^pro^)‐catalysed hydrolysis of linear oligopeptide fragments derived from the sequence of SARS‐CoV‐2 pp1a/1ab [33, 43]. The SPE‐MS assays are of use in investigating the effects of small‐molecules on protease catalysis, complementing commonly used absorbance‐ or fluorescence‐based assays [33, 43, 44, 45, 46, 47, 48, 49, 50, 51, 52, 53]. We have extended the substrate scope of the SPE‐MS PL^pro^ assays to comprise *N* ^ε^‐lysine‐branched oligopeptides mimicking post‐translational protein modification by Ub/UBLs; the *N* ^ε^‐lysine‐branched oligopeptides were computationally predicted to bind to both the PL^pro^ S and S′ sites [34]. Importantly, the SPE‐MS assays using *N* ^ε^‐lysine‐branched oligopeptides with sequences based on different UBLs recapitulated the reported cellular substrate preferences of SARS‐CoV‐2 PL^pro^, implying that these SPE‐MS assays can differentiate between the PL^pro^ deISGylase and pp1a/1ab‐processing activities [34].

Here we report how SPE‐MS inhibition assays with linear and *N* ^ε^‐lysine‐branched oligopeptide substrates were used to distinguish between small‐molecules acting on the deISGylase and pp1a/1ab‐processing activities of PL^pro^. The results reveal that the reported deubiquitinase inhibitor ML364 has potential for substrate‐selective PL^pro^ inhibition, as supported by structure‐activity relationship and computational studies. Although the utility of ML364 derivatives for cellular studies may be limited due to cytotoxicity, the results clearly show the potential of SPE‐MS assays to help enable the development of substrate‐selective inhibitors of SARS‐CoV‐2 PL^pro^ and, by implication, of other proteolytic enzymes.

## 2 Results

### 2.1 Development of Mass Spectrometry Assays for Inhibition Studies on the PL^pro^ deISGylase Activity

SPE‐MS‐based SARS‐CoV‐2 PL^pro^ inhibition assays with an *N* ^ε^‐lysine‐branched oligopeptide substrate were developed to complement SPE‐MS PL^pro^ inhibition assays using the pp1a/1ab‐derived linear oligopeptide **nsp2/3_808‐827_** as a substrate (Figure 1) [33], in order to distinguish between the effects of small‐molecules on the PL^pro^ deISGylase and pp1a/1ab‐processing activities. The *N* ^ε^‐Lys_193_‐branched IRF3_189‐197_‐ISG15 oligopeptide (**IRF3‐ISG15**) was selected as an SPE‐MS assay substrate, because it was amongst the most efficient *N* ^ε^‐Lys‐branched PL^pro^ substrates reported and because it can be prepared via solid‐phase peptide synthesis in high quantity and purity [34].

SPE‐MS PL^pro^ assays with **IRF3‐ISG15** as the substrate were performed using the conditions previously used with **nsp2/3_808‐827_** as the substrate [33]. Assays were performed in the presence of the inert N‐terminally *N*‐acetylated C‐ and N‐terminal product peptides (i.e., Ac‐LSTVFMNLRLRGG‐NH_2_, Ac‐ENPLKRLLV‐NH_2_) to enable quantification of PL^pro^ catalysis [34]. This setup also helps mitigate potential false‐positive results due to compound‐induced ionization suppression of the C‐ and N‐terminal product peptides [33, 47].

The SPE‐MS PL^pro^ inhibition assays with **IRF3‐ISG15** were used to determine the half‐maximal inhibitory concentrations (IC_50_ values) of nine reported small‐molecule PL^pro^ inhibitors. The inhibition results with **IRF3‐ISG15** were similar to those reported with **nsp2/3_808‐827_** (Supplementary Table S1) [33]; signal‐to‐noise (S/N) ratios and Z′‐factors [54] indicated high assay robustness (Supplementary Figure S1). The validated PL^pro^ inhibitor GRL0617 [55] efficiently inhibited PL^pro^ irrespective of the substrate used, with potencies in the range of those reported using orthogonal assays (i.e. IC_50_ ∼1.6‐2.4 µM [16, 36, 42, 56, 57, 58]; Table 1, entry i). GRL0617 was therefore used as a positive inhibition control in subsequent studies. As previously demonstrated with **nsp2/3_808‐827_**, many other reported PL^pro^ inhibitors working via noncovalent substrate‐competitive binding failed to efficiently inhibit in the SPE‐MS assays [33], likely due to their inability to compete with the relatively tight‐binding **IRF3‐ISG15** substrate; by contrast, they are reported to inhibit in fluorescence‐based PL^pro^ assays which employ relatively less efficient substrates [39].

**TABLE 1:** Effects of broad‐spectrum DUB inhibitors on PL^pro^ catalysis.

	DUB inhibitor	^a^IC_50_ [µM] (using linear nsp2/3_808‐827_)	^b^IC_50_ [µM] (using *N* ^ε^‐Lys‐branched IRF3‐ISG15)
i	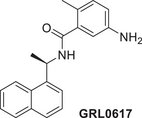	3.4 ± 0.4	2.9 ± 0.4
ii	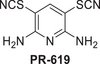	0.14 ± 0.03	0.11 ± 0.01
iii	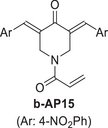	0.82 ± 0.15	0.79 ± 0.18
iv	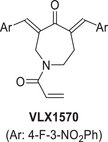	2.2 ± 0.5	2.2 ± 0.5
v	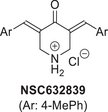	34 ± 12	46 ± 17
vi	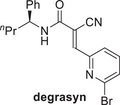	13 ± 4	>50
vii	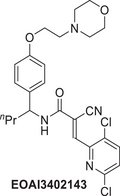	>50	>50

^a^ SPE‐MS inhibition assays were performed as reported [33], using: SARS‐CoV‐2 PL^pro^ (0.2 µM), **nsp2/3_808‐827_** (VTNNTFTLKGGAPTKVTFGI‐NH_2_ [33]; 2.0 µM), and the product standards: Ac‐VTNNTFTLKGG‐NH_2_ (0.2 µM), and Ac‐APTKVTFGI‐NH_2_ (0.2 µM) in buffer (50 mM Tris, pH 8.0, 37°C);

^b^ SPE‐MS inhibition assays were performed using: SARS‐CoV‐2 PL^pro^ (0.2 µM), *N* ^ε^‐Lys‐branched **IRF3‐ISG15** [34] (Figure 1; 2.0 µM), and the product standards: Ac‐LSTVFMNLRLRGG‐NH_2_ (0.2 µM) [34], and Ac‐ENPLKRLLV‐NH_2_ (0.2 µM) [34] in buffer (50 mM Tris, pH 8.0, 37°C). Inhibitors were commercially sourced and used as received. Selected dose‐response curves are shown in Figure 2. Results are means of three independent runs (n = 3; mean ± standard deviation, SD).

### 2.2 Effects of Broad‐Spectrum DUB Inhibitors on the deISGylase Activity of PL^pro^

Following assay validation, the effects of six reported broad‐spectrum DUB inhibitors on catalysis by isolated recombinant SARS‐CoV‐2 PL^pro^ were assessed using SPE‐MS assays with the linear **nsp2/3_808‐827_** [33] and the branched **IRF3‐ISG15** [34] substrates to investigate their potential for substrate‐selective PL^pro^ inhibition. The results reveal that PR‐619 [59] inhibited PL^pro^ >25‐fold more efficiently than GRL0617 [55] regardless of the substrate employed (Table 1, entries i‐ii), contrasting with fluorescence‐based assay results using Ub‐AMC as a substrate [60]. The inhibition potencies of structurally related b‐AP15 [61] and VLX1570 [62] were also substrate‐independent, with b‐AP15 being ∼2‐fold more efficient in inhibiting PL^pro^ than VLX1570 and GRL0617 (Table 1, entries iii and iv). NSC632839 [63] inhibited PL^pro^ substantially less efficiently than structurally related b‐AP15 (Table 1, entry v); although NSC632839 might have a marginal preference for inhibition of PL^pro^ with **nsp2/3_808‐827_** (IC_50_ ∼ 34 µM) than with **IRF3‐ISG15** (IC_50_ ∼ 46 µM), the difference is within experimental error.

**FIGURE 2:**
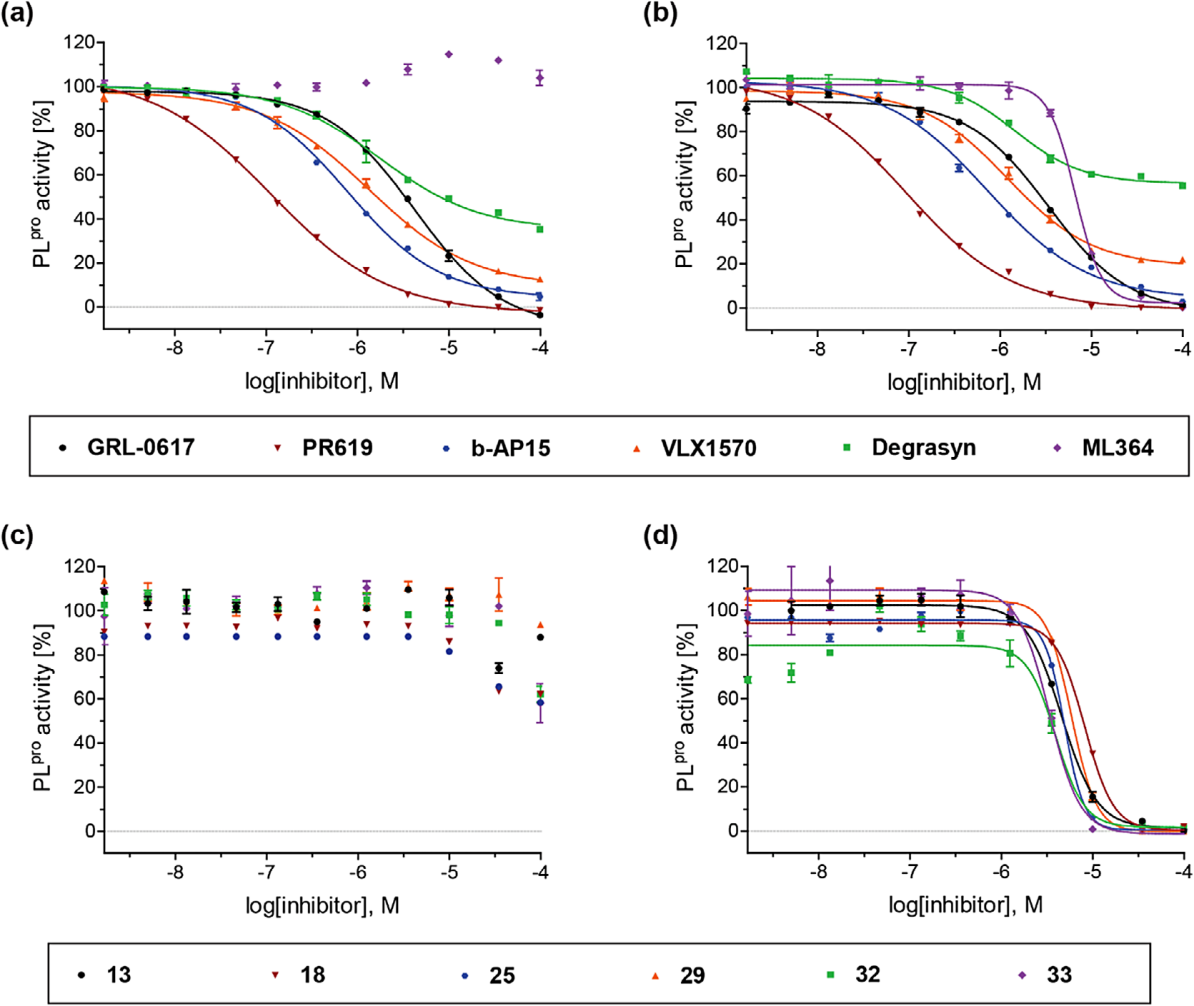
**The reported DUB inhibitor ML364 has potential for substrate‐selective PL^pro^ inhibition**. (**a**‐**b**) Representative dose‐response curves used to determine IC_50_‐values for reported DUB inhibitors obtained with isolated recombinant SARS‐CoV‐2 PL^pro^ using SPE‐MS assays with: (**a**) the linear **nsp2/3_808‐827_** [33] or (**b**) the *N* ^ε^‐Lys‐branched **IRF3‐ISG15** [34] (Figure 1). Color code: GRL0617 [55] (black circles), PR‐619 [59] (red inverse triangles), b‐AP15 [61] (blue circles), VLX1570 [62] (orange triangles), Degrasyn (green boxes), and ML364 [66] (violet diamonds). (**c**‐**d**) Representative dose‐response curves used to determine IC_50_‐values for ML364 derivatives with isolated recombinant SARS‐CoV‐2 PL^pro^ using SPE‐MS assays with: (**c**) the linear **nsp2/3_808‐827_** [33] or (**d**) the *N* ^ε^‐Lys‐branched **IRF3‐ISG15** [34] (Figure 1). Color code: **13** (black circles), **18** (red inverse triangles), **25** (blue circles), **29** (orange triangles), **32** (green boxes), and **33** (violet diamonds). Results are means of technical duplicates (n = 2; mean ± SD), three dose‐response curves, each composed of technical duplicates, were independently determined using SPE‐MS PL^pro^ inhibition assays.

The DUB inhibitor Degrasyn (WP1130) [64] apparently inhibited PL^pro^‐catalyzed hydrolysis of **nsp2/3_808‐827_** (IC_50_ ∼ 13 µM; Table 1, entry vi), but not of **IRF3‐ISG15** (IC_50_> 50 µM; Table 1, entry vi). However, analysis of the corresponding dose‐response curves show imperfect levels of PL^pro^ inhibition (Figure 2), indicating that Degrasyn was not an efficient PL^pro^ inhibitor. This conclusion is further supported by the observation that the Degrasyn derivative EOAI3402143 [65] did not inhibit PL^pro^ (Table 1, entry vii).

### 2.3 ML364 is a Substrate‐Selective PL^pro^ Inhibitor In Vitro

SPE‐MS assays were then used to investigate the effects of 12 small‐molecules, which are reported to selectively inhibit subsets of human DUBs via noncovalent binding [66, 67, 68, 69, 70, 71, 72, 73, 74, 75, 76, 77], on catalysis by isolated PL^pro^ (Table 2). The results reveal that most of the tested DUB inhibitors did not efficiently inhibit PL^pro^, irrespective of whether **nsp2/3_808‐827_** or **IRF3‐ISG15** were employed as substrates (IC_50_> 50 µM; Table 2, entries i‐xi), consistent with their high selectivity for inhibition of human DUBs. Note that reported results on the effects of some of these DUB inhibitors on PL^pro^ catalysis suffered from imperfect reproducibility [60].

**TABLE 2:** Effects of reported DUB inhibitors on PL^pro^ catalysis.

	DUB inhibitor	^a^IC_50_ [µM] (using linear nsp2/3_808‐827_)	^b^IC_50_ [µM] (using *N* ^ε^‐Lys‐branched IRF3‐ISG15)		DUB inhibitor	^a^IC_50_ [µM] (using linear nsp2/3_808‐827_)	^b^IC_50_ [µM] (using *N* ^ε^‐Lys‐branched IRF3‐ISG15)
i	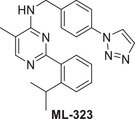	>50	>50	vii	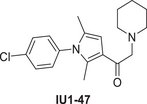	>50	>50
ii	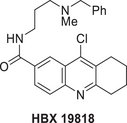	>50	>50	viii	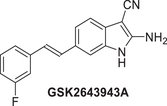	>50	>50
iii	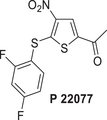	>50	>50	ix	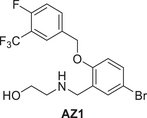	>50	>50
iv	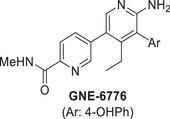	>50	>50	x	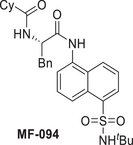	>50	>50
v	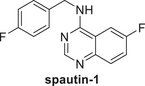	>50	>50	xi	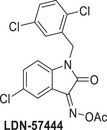	>50	>50
vi	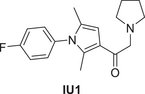	>50	>50	xii	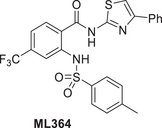	**>50**	**6.4 ± 0.5**

^a^ SPE‐MS inhibition assays were performed as reported [33], using: SARS‐CoV‐2 PL^pro^ (0.2 µM), **nsp2/3_808‐827_** (VTNNTFTLKGGAPTKVTFGI‐NH_2_ [33]; 2.0 µM), and the product standards: Ac‐VTNNTFTLKGG‐NH_2_ (0.2 µM), and Ac‐APTKVTFGI‐NH_2_ (0.2 µM) in buffer (50 mM Tris, pH 8.0, 37°C);

^b^ SPE‐MS inhibition assays were performed using: SARS‐CoV‐2 PL^pro^ (0.2 µM), *N* ^ε^‐Lys‐branched **IRF3‐ISG15** [34] (Figure 1; 2.0 µM), and the product standards: Ac‐LSTVFMNLRLRGG‐NH_2_ (0.2 µM) [34], and Ac‐ENPLKRLLV‐NH_2_ (0.2 µM) [34] in buffer (50 mM Tris, pH 8.0, 37°C). Selected dose‐response curves are shown in Figure 2. Results are means of three independent runs (n = 3; mean ± SD).

**FIGURE 3:**
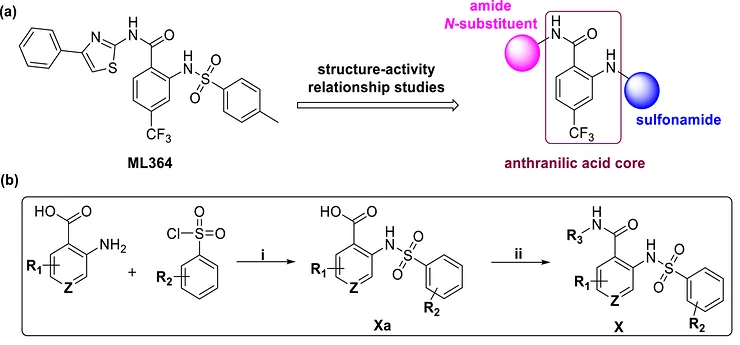
**The modular structure of ML364 facilitates structure‐activity relationship studies**. (**a**) ML364 bears an anthranilic acid core with sulfonamide and amide substituents [66]. (**b**) Outline synthesis of ML364 derivatives. Reagents and conditions: i) Na_2_CO_3_, H_2_O, 80°C, 50–85%; ii) amine, HATU [78], *^i^* Pr_2_NEt [79], DMF, 80°C, 7–65%. Z: CR or N. For details, see Supporting Information and Tables 3, 4, 5.

Interestingly, the reported ubiquitin‐specific protease 2 (USP2)‐selective inhibitor ML364 [66] inhibited PL^pro^‐catalysed hydrolysis of **IRF3‐ISG15** (IC_50_ ∼ 6.4 µM; Table 2, entry xii), but not of **nsp2/3_808‐827_** (IC_50_> 50 µM; Table 2, entry xii), indicating potential for substrate‐selective inhibition. The Hill slope of the ML364 dose‐response curves obtained with **IRF3‐ISG15** derived from the predicted value of ‐1 (Figure 2), suggesting the possibility for a complex inhibition mode including e.g., binding at multiple sites and/or binding of ML364 aggregates. Note that fluorescence‐based assays with Z‐LRGG‐AMC as the substrate have shown that ML364 inhibits PL^pro^ at a concentration of 0.2 mM [60]. Hence, data indicating that ML364 may be a substrate‐selective PL^pro^ inhibitor should be interpreted with care. Given the modular structure of ML364 (Figure 3), structure‐activity relationship studies were performed aiming to obtain more potent inhibition and investigate roles of the three main structural elements of ML364 (i.e., the amide, sulfonamide, and anthranilic acid groups), including on the apparent selectivity for inhibition of the PL^pro^ deISGylase activity over its pp1a/1ab‐processing activity.

### 2.4 Effects of the ML364 Amide Group on PL^pro^ Inhibition

Following the reported synthesis of ML364 [66], a set of ML364 derivatives with varied amide *N*‐substituents was synthesized from reported 2‐((4‐methylphenyl)sulfonamido)‐4‐(trifluoromethyl)benzamide **1a** [66] in 18–55% overall yields (Figure 3b) to investigate the effects of the amide group on PL^pro^ inhibition. The effects of the resultant ML364 derivatives on catalysis by isolated PL^pro^ were investigated using SPE‐MS assays with both **IRF3‐ISG15** and **nsp2/3_808‐827_** as substrates. The combined results reveal that none of the synthesized ML364 derivatives tested inhibited the pp1a/1ab‐processing ability of PL^pro^, whereas some of the ML364 derivatives inhibited the deISGylase activity of PL^pro^, supporting the assignment of ML364 as a substrate‐selective inhibitor.

The results indicate that the 2‐amino‐4‐phenylthiazole group of ML364 is important for the efficient inhibition of the PL^pro^ deISGylase activity, because: (i) the ML364 *N*‐alkyl amides **1–3** did not inhibit both PL^pro^ activities (Table 3, entries ii‐iv), (ii) substitution of the thiazole sulfur atom for an oxygen atom or an NH group reduced inhibition potency by ∼2‐ and ∼5‐fold, respectively (Table 3, entries v‐vi), and (iii) ML364 derivatives lacking the thiazole C4 phenyl group (i.e., **6**), bearing the phenyl substituent at the thiazole C3 position (i.e., **7**) or bearing a benzothiazole group instead of the 4‐phenylthiazole group (i.e., **8**), did not inhibit PL^pro^ (Table 3, entries vii‐ix). The ML364 derivative **9** with a *para*‐methoxy‐substituted C4 phenyl group inhibited PL^pro^ with similar potency as ML364 (Table 3, entry x). By contrast, the *para*‐chloro‐substituted and *para*‐nitro‐substituted derivatives **10** and **11** inhibited PL^pro^ with ∼5‐fold reduced potency, whereas the *para*‐phenyl‐substituted derivative **12** did not inhibit (Table 3, entries xi‐xiii). Notably, the naphthyl‐substituted ML364 derivative **13** inhibited PL^pro^ with similar potency as ML364, indicating that groups sterically bulkier than phenyl groups are tolerated at the thiazole C4 position (Table 3, entry xiv).

**TABLE 3:** Effects of ML364 amide group on PL^pro^ catalysis.

	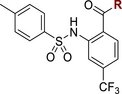	^a^IC_50_ [µM] (using linear nsp2/3_808‐827_)	^b^IC_50_ [µM] (using *N* ^ε^‐Lys‐branched IRF3‐ISG15)		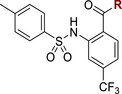	^a^IC_50_ [µM] (using linear nsp2/3_808‐827_)	^b^IC_50_ [µM] (using *N* ^ε^‐Lys‐branched IRF3‐ISG15)
	R	R		
i	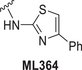	>50	6.4 ± 0.5	viii	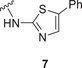	>50	>50
ii	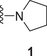	>50	>50	ix	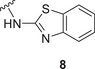	>50	>50
iii	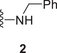	>50	>50	x	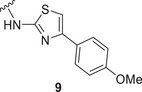	>50	7.5 ± 0.6
iv	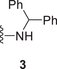	>50	33 ± 1	xi	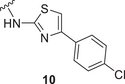	>50	35 ± 8
v	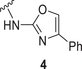	>50	17 ± 2	xii	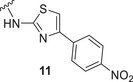	>50	32 ± 1
vi	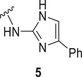	>50	32 ± 1	xiii	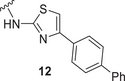	>50	>50
vii	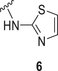	>50	>50	xiv	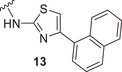	>50	5.3 ± 0.5

^a^ SPE‐MS inhibition assays were performed as reported [33], using: SARS‐CoV‐2 PL^pro^ (0.2 µM), **nsp2/3_808‐827_** (VTNNTFTLKGGAPTKVTFGI‐NH_2_ [33]; 2.0 µM), and the product standards: Ac‐VTNNTFTLKGG‐NH_2_ (0.2 µM), and Ac‐APTKVTFGI‐NH_2_ (0.2 µM) in buffer (50 mM Tris, pH 8.0, 37°C);

^b^ SPE‐MS inhibition assays were performed using: SARS‐CoV‐2 PL^pro^ (0.2 µM), *N* ^ε^‐Lys‐branched **IRF3‐ISG15** [34] (Figure 1; 2.0 µM), and the product standards: Ac‐LSTVFMNLRLRGG‐NH_2_ (0.2 µM) [34], and Ac‐ENPLKRLLV‐NH_2_ (0.2 µM) [34] in buffer (50 mM Tris, pH 8.0, 37°C). Selected dose‐response curves are shown in Figure 2. Results are means of two independent runs (n = 2; mean ± SD).

### 2.5 Effects of the ML364 Sulfonamide Group on PL^pro^ Inhibition

ML364 sulfonamide derivatives **14–20** were synthesized in two steps [66] via the corresponding acid intermediates **14a‐20a** in 3–21% overall yields (Figure 3b) to investigate the effects of the sulfonamide group on PL^pro^ inhibition potency and substrate selectivity. The chloro‐substituted ML364 derivative **10** was used as a template for sulfonamide modifications, because the corresponding sulfonamide derivatives were more potent inhibitors than those lacking the chlorine (Supplementary Table S2). The SPE‐MS assay results reveal that substitution of the ML364 sulfone group for a methylene or a carbonyl group ablates PL^pro^ inhibition (Table 4, entries ii‐iii). Thus, subsequent efforts focused on altering the sulfonamide aryl group.

**TABLE 4:** Effects of the ML364 aniline *N*‐substituent and anthranilic acid group on PL^pro^ catalysis.

	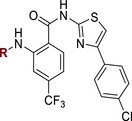	^a^IC_50_ [µM] (using linear nsp2/3_808‐827_)	^b^IC_50_ [µM] (using *N* ^ε^‐Lys‐branched IRF3‐ISG15)		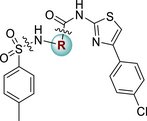	^a^IC_50_ [µM] (using linear nsp2/3_808‐827_)	^b^IC_50_ [µM] (using *N* ^ε^‐Lys‐branched IRF3‐ISG15)
	R	R		
i	ML364	>50	6.4 ± 0.5	ix	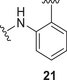	>50	20 ± 1
ii	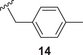	>50	>50	x	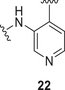	>50	20 ± 3
iii	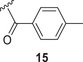	>50	>50	xi	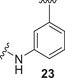	>50	>50
iv	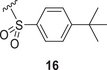	>50	13 ± 6	xii	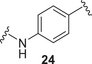	>50	>50
v	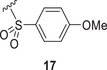	>50	8.1 ± 2.4	xiii	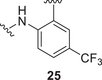	>50	4.5 ± 0.3
vi	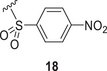	>50	6.4 ± 1.0	xiv	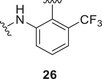	>50	>50
vii	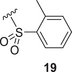	>50	>50	xv	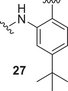	>50	29 ± 2
viii	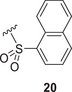	>50	7.6 ± 3.8	xvi	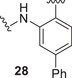	>50	>50

^a^ SPE‐MS inhibition assays were performed as reported [33], using: SARS‐CoV‐2 PL^pro^ (0.2 µM), **nsp2/3_808‐827_** (VTNNTFTLKGGAPTKVTFGI‐NH_2_ [33]; 2.0 µM), and the product standards: Ac‐VTNNTFTLKGG‐NH_2_ (0.2 µM), and Ac‐APTKVTFGI‐NH_2_ (0.2 µM) in buffer (50 mM Tris, pH 8.0, 37°C);

^b^ SPE‐MS inhibition assays were performed using: SARS‐CoV‐2 PL^pro^ (0.2 µM), *N* ^ε^‐Lys‐branched **IRF3‐ISG15** [34] (Figure 1; 2.0 µM), and the product standards: Ac‐LSTVFMNLRLRGG‐NH_2_ (0.2 µM) [34], and Ac‐ENPLKRLLV‐NH_2_ (0.2 µM) [34] in buffer (50 mM Tris, pH 8.0, 37°C). Selected dose‐response curves are shown in Figure 2. Results are means of two independent runs (n = 2; mean ± SD).

Substitution of the tolyl methyl group of ML364 for a sterically bulkier *tert*‐butyl group decreased PL^pro^ inhibition potency ∼2‐fold with **IRF3‐ISG15** (IC_50_ ∼ 13 µM; Table 4, entry iv), while its substitution for an electron‐donating methoxy or an electron‐withdrawing nitro group did not substantially affect inhibition potency compared to ML364 (Table 4, entries v and vi). The 1‐naphthyl‐substituted sulfonamide **20** inhibited the PL^pro^ deISGylase activity with similar potency as ML364 (IC_50_ ∼ 8 µM; Table 4, entry viii), whereas changing the position of the ML364 tolyl methyl group from the *para*‐ to the *ortho*‐sulfonamide position ablated inhibition (Table 4, entry vii). The combined results indicate that the sulfonamide group is important for inhibition and that, while different sulfonamide aryl groups are tolerated, they do not substantially increase inhibition potency.

### 2.6 Effects of the ML364 Anthranilic Acid Group on PL^pro^ Inhibition

Given that modifications to the amide and sulfonamide groups of ML364 did not improve potency for inhibition of the PL^pro^ deISGylase activity, we investigated the effects of the ML364 anthranilic acid group on PL^pro^ inhibition. Nine chloro‐substituted ML364 derivatives (i.e., **21–28**) with altered anthranilic acid groups were synthesized using different anthranilic acid derivatives as starting materials (Figure 3b).

The results reveal that substitution of the ML364 anthranilic acid CF_3_ substituent for a hydrogen reduced potency for inhibition of the PL^pro^ deISGylase activity ∼3‐fold compared to ML364, as did substitution of its C‐CF_3_ group for a nitrogen (Table 4, entries ix‐x); by contrast, the corresponding 3‐aminobenzoic acid‐ and 4‐aminobenzoic acid‐derived isomers of **21** did not inhibit (Table 4, entries xi‐xii). Notably, altering the position of the ML364 CF_3_ group from the position *para* to the carboxamide to the *meta* position resulted in a ∼2‐fold increased potency for inhibition of the PL^pro^ deISGylase activity compared to ML364, while the corresponding *ortho*‐CF_3_ isomer did not inhibit (Table 4, entries xiii‐xiv). Substitution of the CF_3_ group of **10** for a sterically bulkier *tert*‐butyl group decreased PL^pro^ inhibition potency by ∼4‐fold compared to ML364, while its substitution for a phenyl group ablated inhibition (Table 4, entries xv and xvi).

### 2.7 Development of Improved ML364‐Based PL^pro^ Inhibitors

The ML364 amide, sulfonamide, and anthranilic acid groups associated with the most efficient PL^pro^ inhibition were combined to synthesize the ML364 derivatives **29–33** in 8–24% overall yields (Table 5). The SPE‐MS assay results reveal that the ML364 derivatives **32** and **33** inhibited isolated PL^pro^ with ∼2‐fold improved potency compared to ML364, indicating that bulky 1‐naphthyl substituents at both the sulfonamide and amide positions have beneficial effects on inhibition potency and substrate selectivity. Thus, none of the ML364 derivatives substantially inhibited the pp1a/1ab‐processing activity of PL^pro^ (Table 5, entries v‐vi); they inhibited the deISGylase activity of PL^pro^ over its pp1a/1ab‐processing activity with >27:1 selectivity.

**TABLE 5:** Effects of optimized ML364‐based inhibitors on PL^pro^ inhibition potency and substrate selectivity.

	ML364 derivative	^a^IC_50_ [µM] (using linear nsp2/3_808‐827_)	^b^IC_50_ [µM] (using *N* ^ε^‐Lys‐branched IRF3‐ISG15)	Selectivity for IRF3‐ISG15 versus nsp2/3_808‐827_ inhibition
i	ML364	>100	6.4 ± 0.5	>15:1
ii	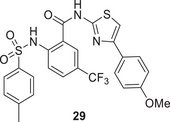	>100	6.7 ± 1.2	>14:1
iii	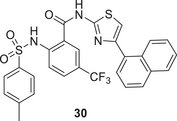	>100	7.8 ± 2.6	>13:1
iv	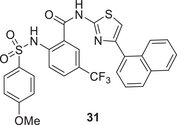	>100	23 ± 2	>4:1
v	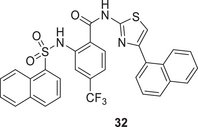	>100	3.4 ± 0.2	>29:1
vi	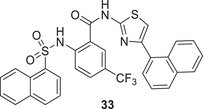	>100	3.6 ± 0.1	>27:1

^a^ SPE‐MS inhibition assays were performed as reported [33], using: SARS‐CoV‐2 PL^pro^ (0.2 µM), **nsp2/3_808‐827_** (VTNNTFTLKGGAPTKVTFGI‐NH_2_ [33]; 2.0 µM), and the product standards: Ac‐VTNNTFTLKGG‐NH_2_ (0.2 µM), and Ac‐APTKVTFGI‐NH_2_ (0.2 µM) in buffer (50 mM Tris, pH 8.0, 37°C);

^b^ SPE‐MS inhibition assays were performed using: SARS‐CoV‐2 PL^pro^ (0.2 µM), *N* ^ε^‐Lys‐branched **IRF3‐ISG15** [34] (Figure 1; 2.0 µM), and the product standards: Ac‐LSTVFMNLRLRGG‐NH_2_ (0.2 µM) [34], and Ac‐ENPLKRLLV‐NH_2_ (0.2 µM) [34] in buffer (50 mM Tris, pH 8.0, 37°C). Selected dose‐response curves are shown in Figure 2. Results are means of two independent runs (n = 2; mean ± SD).

### 2.8 Selectivity of the ML364 Derivatives for PL^pro^ Inhibition

The selectivity of the ML364 derivatives for inhibition of PL^pro^ over M^pro^ was investigated using SPE‐MS assays [43, 47] to inform on possible off‐target effects. The results reveal that eight of the tested 28 ML364 derivatives inhibited isolated recombinant SARS‐CoV‐2 M^pro^ with moderate potency (i.e., 5–15 µM), albeit substantially less efficiently than clinically used M^pro^ inhibitors (Supplementary Table S3).

### 2.9 Cytotoxicity Studies

We investigated the effects of the ML364 derivatives on Vero cells using DAPI staining and on human‐derived MCF7 cells using resazurin assays. The results reveal that the ML364 derivatives were typically cytotoxic at concentrations >50 µM (Supplementary Table S4 and Figure S2). Although further investigation is required, it is possible that at least some of the tested ML364 derivatives inhibit catalysis of host cysteine proteases given that ML364 is a reported USP2 inhibitor [66].

### 2.10 Modeling Studies

**FIGURE 4:**
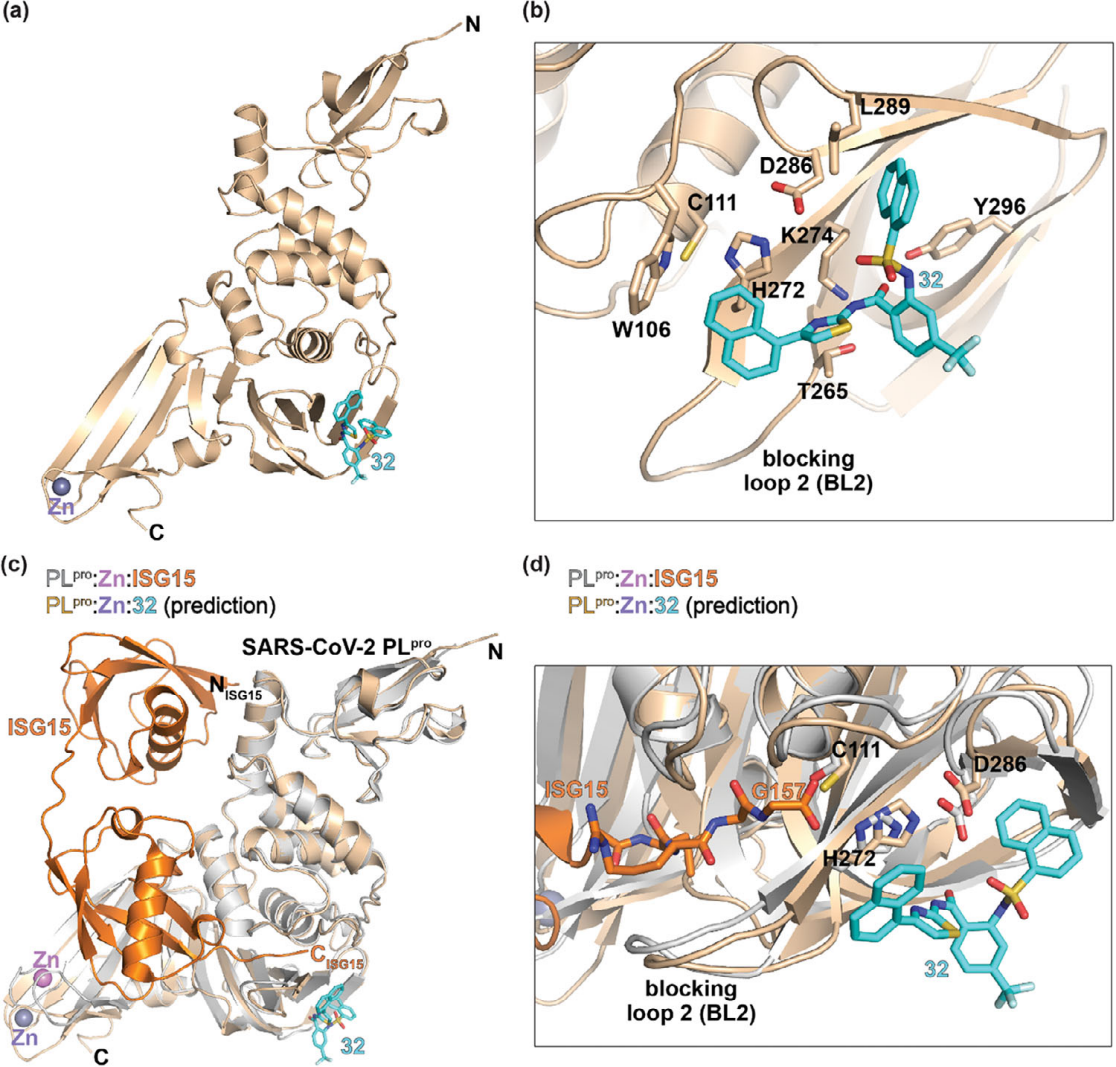
**MD simulations predict that the ML364 derivative 32 binds at the PL^pro^ S′ sites**. (**a**) Predicted binding mode of **32** to the S′ sites of SARS‐CoV‐2 PL^pro^ determined from cluster analysis of three 100 ns MD simulations. (**b**) Interactions of **32** with the side chains of K274, W106, H272, and T265 in the S′ sites. (**c** and **d**).Superimposition of the reported PL^pro^ C111S variant:Zn:ISG15 crystal structure (PDB ID: 7RBS [30]) with the PL^pro^:Zn:**32** prediction suggests that the ML364 derivative **32** does not interfere with ISG15 binding, including with respect to the C‐terminal LRGG motif of ISG15 in the PL^pro^ S sites near the catalytic triad (C111, H272, D286) [3, 35].

We performed docking and molecular dynamics (MD) simulations with the ML364 derivative **32** and SARS‐CoV‐2 PL^pro^ to investigate the mechanism by which **32** selectively inhibits the deISGylase activity of PL^pro^ over its pp1a/1ab‐processing activity (Table 5, entry v). Based on reported computational results on the binding of ML364 derivatives to PL^pro^ [60, 80], we investigated the binding of **32** to both the S and S′ sites of PL^pro^ (Figure 4, Supplementary Figures S3 and S4). MD simulations predict a stable pose of **32** at the S′ sites, in which the thiazole N and amide group of **32** are positioned to hydrogen bond with the side chains of K274 and T265, respectively. The thiazole C4 naphthyl group of **32** also interacts with the side chains of W106 and H272 via π‐stacking interactions (Figure 4). H272 is part of the catalytic triad, but the predicted binding pose of **32** appears to preserve the hydrogen‐bonding network with C111 and D286 (Supplementary Figure S5). Note that, in principle, **32** may adopt alternative binding modes and/or conformations at the PL^pro^ active site, as predicted by reported docking and MD studies with ML364 derivatives [60, 80].

Although binding energy calculations using molecular mechanics with Poisson–Boltzmann or generalized Born and surface area continuum solvation (MMG/PBSA) indicate binding to the S sites to be as favorable as binding to the S′ sites (Supplementary Table S5), the predicted binding pose of **32** at the PL^pro^ S′ sites was observed for a larger fraction of the simulation time than that at the S sites (57% versus 17%; Supplementary Figure S6, Tables S6 and S7). The combined computational results thus suggest that **32** preferentially binds to the S′ sites in a manner that prevents binding of branched substrates, while not directly interfering with the binding mode of linear substrates, in accord with selective inhibition of deISGylase activity (Figure 2, Supplementary Figure S4). Note, however, that it has not yet been crystallographically defined how the substrate residues to the C‐terminal side of the scissile substrate amide bond bind in the PL^pro^ S′ sites. The PL^pro^ docking and MD simulations protocol was validated using the reported inhibitor GRL0617 [55] (Supplementary Figure S7).

## 3 Discussion

Novel COVID‐19 therapeutics are needed to complement clinically‐used inhibitors of the SARS‐CoV‐2 M^pro^ and the SARS‐CoV‐2 RNA‐dependent RNA polymerase, the efficacy of which is increasingly challenged by resistance [81, 82, 83, 84, 85]. In addition to these targets, other SARS‐CoV‐2‐encoded proteins are under investigation for COVID‐19 treatment [86, 87, 88, 89], including PL^pro^, which, like M^pro^, is a protease essential for pp1a/1ab‐processing [1, 2, 3]. Although inhibitors of PL^pro^ have demonstrated efficacy in infection models [90, 91, 92, 93], their use may be susceptible to resistance [94, 95]. Thus, medicinal chemistry efforts are ongoing to develop novel types of PL^pro^ inhibitors [8, 32, 96, 97, 98, 99, 100, 101, 102].

The functions of PL^pro^ extend beyond pp1a/1ab‐processing, in particular to catalyzing the hydrolysis of isopeptide bonds linking ISG15 (and Ub) with human host proteins [16, 17]. The development of probe compounds that distinguish between the pp1a/1ab‐processing and deISGylase activities of PL^pro^ is of interest to better understand the roles of PL^pro^ during infection [103]. Substrate‐selective PL^pro^ inhibitors are also of interest because they can inform on how the deISGylase/deubiquitinase activities of PL^pro^s from different coronaviruses affect pathogenicity. To our knowledge, substrate‐selective PL^pro^ inhibitors have not yet been reported, likely in part reflecting a lack of assays with isolated PL^pro^ that differentiate its pp1a/1ab‐processing and deISGylase activities.

We employed SPE‐MS assays with the pp1a/1ab‐derived linear oligopeptide **nsp2/3_808‐827_** [33] and the *N* ^ε^‐Lys_193_‐branched IRF3_189‐197_‐ISG15 oligopeptide **IRF3‐ISG15** mimicking post‐translational ISGylation of IRF3 to distinguish between the effects of small‐molecules on the deISGylase and pp1a/1ab‐processing activities of isolated recombinant SARS‐CoV‐2 PL^pro^. The combined inhibition results imply that the reported USP2 inhibitor ML364 [66] selectively inhibits the deISGylase activity of PL^pro^ (IC_50_ ∼ 6.7 µM with **IRF3‐ISG15** compared to >50 µM with **nsp2/3_808‐827_**; Table 2, entry xii). Structure‐activity relationship studies reveal that the ML364 derivatives **32** and **33** with sterically bulky 1‐naphthyl substituents inhibited the PL^pro^ deISGylase activity ∼2‐fold more efficiently than ML364 (Table 5). Notably, none of the tested ML364 derivatives inhibited the pp1a/1ab‐processing activity of PL^pro^ (Tables 3, 4, 5).

MD simulations of **32** suggest that ML364 derivatives may exert substrate‐selective inhibition of the PL^pro^ deISGylase activity by binding in the PL^pro^ S′ sites rather than the S sites (Figure 4), as supported by a larger fraction of simulation time for which the S′ binding mode was observed (Supplementary Figure S6, Tables S6 and S7). These results are consistent with previous modelling studies on ML364‐related molecules [80], although alternative binding modes cannot be ruled out (Supplementary Figure S3) [60, 104].

Binding to the PL^pro^ S′ sites could enable substrate‐selective inhibition, because post‐translationally modified human host proteins are branched C‐terminal to the scissile amide bond and so likely have additional binding interactions in the PL^pro^ S′ site region compared to pp1a/1ab (Figure 1). The branched structure of **IRF3‐ISG15** likely mimics these additional binding interactions in the PL^pro^ S′ site region, as implied by reported competition studies showing that PL^pro^ preferred **IRF3‐ISG15** as a substrate over **nsp2/3_808‐827_** [34]. Further experimental work is required to validate the potential of ML364 and derivatives for substrate‐selective PL^pro^ inhibition within cells.

The potential of ML364 derivatives for substrate‐selective inhibition of PL^pro^ is *inter alia* precedented by reported substrate‐selective inhibitors for: thrombin [105], γ‐secretase [106, 107], cyclooxygenase‐2 [108, 109, 110], a disintegrin and metalloproteinase domain‐containing protein 10 [111], prolyl oligopeptidase [112], sirtuin 2 [113], and insulin‐degrading enzyme [114, 115]. Substrate‐selective enzyme inhibition can be achieved through different mechanisms, including by [31]: (i) substrate‐competitive inhibitors, which impede binding of relatively weakly binding substrates but not of tight‐binding substrates, (ii) binding to sites not occupied by all the substrates, such as S′ sites or exosites, (iii) allosteric binding modes which alter protein conformations and/or hinder binding of a subset of substrates, and (iv) molecules that selectively bind one substrate over others, as precedented by inhibitors of enzymes other than PL^pro^ [116, 117, 118, 119, 120, 121, 122]. By contrast, active site inhibitors reacting with the nucleophilic active site cysteine (C111) should not directly enable substrate‐selective PL^pro^ inhibition [123, 124, 125].

The modest potency of the current ML364 derivatives suggests that the relatively shallow PL^pro^ S′ sites are more challenging to target than the well‐defined S sites, suggesting that different types of inhibitors may achieve higher levels of substrate selectivity. Cyclic peptides are an attractive alternative to improve substrate‐selective PL^pro^ inhibition due to their ability to bind to protein surfaces with high affinity [126, 127, 128]. Although cyclic peptide inhibitors of PL^pro^ [129], M^pro^ [130, 131, 132, 133, 134, 135, 136, 137, 138], and human DUBs [139, 140] are reported, their potential for substrate‐selective inhibition remains to be investigated.

Further work with folded proteins is required to validate the potential of ML364 derivatives for selective inhibition of the PL^pro^ deISGylase activity in cells. However, such studies may be compromised by the cytotoxicity of the current ML364 derivatives, which might also inhibit human DUBs given the similar active site geometries and substrate scopes of PL^pro^ and DUBs (ML364 is a reported USP2 inhibitor [66]), and possibly other proteins, given that at least some of the ML364 derivatives inhibited isolated M^pro^ (Supplementary Table S3). Of particular interest is the potential of ML364 and derivatives for dual PL^pro^ and M^pro^ inhibition, which could be advantageous for the development of more potent and robust COVID‐19 therapeutics, reducing the likelihood of drug‐induced resistance [141, 142].

The combined results suggest that SPE‐MS assays may also have potential to enable identification of inhibitors distinguishing the PL^pro^ deISGylase and DUB activities [16, 17], provided two different *N* ^ε^‐Lys‐branched oligopeptides with sequences derived from ISG15 and Ub are employed as substrates [34]. Molecules selectively inhibiting the PL^pro^ deISGylase or DUB activity could complement functional assignment work on Middle East Respiratory Syndrome coronavirus PL^pro^ variants, which showed only deISGylase or DUB activity [143, 144].

In addition to the PL^pro^ pp1a/1ab‐processing, deISGylase, and DUB activities, PL^pro^ is also reported to catalyse hydrolysis of peptide bonds C‐terminal to LXGG motifs in the coding sequence of host proteins [145, 146, 147, 148]. Thus, SPE‐MS assays with linear oligopeptides whose sequences derive from those of proposed host protein substrates might enable identification of molecules which inhibit PL^pro^‐catalysed hydrolysis of specific host substrates to inform on their relevance during infection [149].

The utility of the SPE‐MS assays for identification of substrate‐selective PL^pro^ inhibitors might extend to viruses other than SARS‐CoV‐2 that also encode for PL^pro^‐related proteases with DUB activity [150, 151, 152, 153], as well as  human DUBs, given their diverse substrate scope, with several human DUBs showing selectivity for specific substrates. For example, like PL^pro^, human ubiquitin‐specific peptidase 18 (USP18) preferentially catalyses deISGylation over deubiquitinylation reactions [154, 155, 156]. Considering the importance of DUBs in human physiology and as medicinal chemistry targets [18, 19, 20, 21, 25, 155], identification of substrate‐selective DUB inhibitors may enable development of safer medicines.

## 4 Materials and Methods

### Synthesis of PL^pro^ Inhibitors

The synthesis and characterization of ML364 derivatives are described in the associated Supporting Information.

### Production and Purification of Isolated Recombinant SARS‐CoV‐2 PL^pro^

Recombinant isolated PL^pro^ from the SARS‐CoV‐2 nsp3 (E746‐T1063) Wuhan‐Hu‐1 [157] strain was produced using *E. coli* Lemo21(DE3) cells and purified (as validated by SDS‐PAGE and MS analyses) as reported [33].

### Peptide Synthesis

Linear (e.g., **nsp2/3_808‐827_** and the *N*‐acetylated product standards) and *N* ^ε^‐Lys‐branched (i.e., **IRF3‐ISG15**) oligopeptides were prepared by solid phase peptide synthesis (SPPS) using a Liberty Blue peptide synthesizer (CEM Microwave Technology Ltd.), as reported [33, 34].

### SPE‐MS Inhibition Assays

SPE‐MS PL^pro^ inhibition assays were performed using isolated recombinant SARS‐CoV‐2 PL^pro^ and either the **nsp2/3_808‐827_** substrate (VTNNTFTLKGGAPTKVTFGI‐NH_2_ [33]; Figure 1; 2.0 µM) together with the Ac‐VTNNTFTLKGG‐NH_2_ (0.2 µM) and Ac‐APTKVTFGI‐NH_2_ (0.2 µM) product standards as reported [33], or the **IRF3‐ISG15** substrate (Figure 1; 2.0 µM) [34] together with the Ac‐LSTVFMNLRLRGG‐NH_2_ (0.2 µM) [34] and Ac‐ENPLKRLLV‐NH_2_ (0.2 µM) [34] product standards. Note that PL^pro^ aliquots were used for inhibition assays, which were thawed and frozen not more than twice. Solutions of the inhibitors (100%_v/v_ DMSO) were dry dispensed across 384‐well polypropylene assay plates (Greiner, #781280) in a ∼3‐fold and 11‐point dilution series (100 µM top concentration) using an ECHO 550 acoustic dispenser (Labcyte). DMSO and formic acid were used as negative and positive inhibition controls, respectively. The total DMSO concentration was kept constant at 0.5%_v/v_ in all wells of the assay plate. Each reaction was performed in technical duplicates in adjacent wells of the assay plates; assays were also performed at least in independent duplicates (n = 2).

The Enzyme Mixture (25 µL/well), containing SARS‐CoV‐2 PL^pro^ (0.4 µM) in 50 mM Tris buffer (pH 8.0), was dispensed across the inhibitor‐containing 384‐well assay plates with a multidrop dispenser (Thermo Fischer Scientific) at 20°C under an ambient atmosphere. The plates were subsequently centrifuged (1000 rpm, ∼5 s) and incubated for 15 min at 20°C. Substrate Mixtures A or B (25 µL/well) were added using the multidrop dispenser (Substrate Mixture A: **IRF3‐ISG15** [34] (4.0 µM), Ac‐LSTVFMNLRLRGG‐NH_2_ [34] (0.4 µM), and Ac‐ENPLKRLLV‐NH_2_ [34] (0.4 µM) in 50 mM Tris buffer (pH 8.0); Substrate Mixture B: **nsp2/3_808‐827_** [33] (4.0 µM), Ac‐VTNNTFTLKGG [33] (0.4 µM), and Ac‐APTKVTFGD [33] (0.4 µM) in 50 mM Tris buffer (pH 8.0)). The plates were centrifuged (1000 rpm, ∼5 s) and incubated for 100 min (Substrate Mixture A) or 120 min (Substrate Mixture B) at 37°C; the enzyme reaction was then halted by addition of 10%_v/v_ aqueous formic acid (5 µL/well). The plates were then centrifuged (1000 rpm, ∼10 s) and analyzed by MS.

MS analyses were performed using a RapidFire RF 365 high‐throughput sampling robot (Agilent) attached to an iFunnel Agilent 6550 accurate mass quadrupole time‐of‐flight (Q‐TOF) mass spectrometer operated in the positive ionization mode. Assay samples were aspirated under vacuum for 0.6 s, then loaded onto a C4 solid phase extraction (SPE) cartridge. After loading, the C4 SPE cartridge was washed with 0.1%_v/v_ aqueous formic acid to remove nonvolatile buffer salts (5.5 s, 1.5 mL/min). Peptides were eluted from the SPE cartridge with 0.1%_v/v_ aqueous formic acid in 85/15_v/v_ acetonitrile/water into the mass spectrometer (5.5 s, 1.25 mL/min), and the SPE cartridge was re‐equilibrated with 0.1%_v/v_ aqueous formic acid (0.5 s, 1.25 mL/min). The mass spectrometer parameters were: capillary voltage: 4000 V; nozzle voltage: 1000 V; fragmentor voltage: 365 V; gas temperature: 280°C; gas flow: 13 L/min; sheath gas temperature: 350°C; sheath gas flow: 12 L/min.

For analysis of data obtained with Substrate Mixture A, the m/z +1 charge states of the IRF3_189‐197_‐derived product peptide (i.e., ENPLKRLLV‐NH_2_ [34]) and the corresponding *N*‐acetylated product peptide, which was used as an internal standard, were used to extract ion chromatogram data [34]. Additionally, the m/z +2 charge states of the ISG15_145‐157_‐derived product peptide (i.e., LSTVFMNLRLRGG‐NH_2_ [34]) and the corresponding *N*‐acetylated product peptide, which was used as an internal standard, were used to extract ion chromatogram data [34]; peak areas were integrated using the RapidFire Integrator software (Agilent).

For analysis of data obtained with Substrate Mixture B, the m/z +1 charge states of both the C‐terminal and the N‐terminal product peptides, as well as the corresponding *N*‐acetylated C‐terminal and N‐terminal product peptides, which were used as internal standards, were used to extract ion chromatogram data [33].

### Statistical Analysis of SPE‐MS Data

Raw MS data were exported into Microsoft Excel and manually checked for outliers; outliers identified (<3% of all data points) were excluded from subsequent analyses. Processed data were used to calculate the averaged product peptide concentrations using the equation:

Averaged product peptide concentrations = (0.2 µM × (integral C‐terminal product peptide) / (integral *N*‐acetylated C‐terminal product peptide) + 0.2 µM × (integral N‐terminal product peptide) / (integral *N*‐acetylated N‐terminal product peptide)) / 2.

Normalized dose‐response curves (DMSO and formic acid controls) were obtained from the raw data by nonlinear regression and used to determine IC_50_‐values (GraphPad Prism 5). The standard deviation (SD) of two (n = 2; Tables 3, 4, 5) or three (n = 3; Tables 1, 2) independent IC_50_ determinations, each composed of technical duplicates, was calculated using GraphPad Prism 5. Z′‐factors were calculated according to the literature using Microsoft Excel [54]. Data are presented as means of two or three independent runs (n = 2 or 3; mean ± SD) in Tables 1, 2, 3, 4, 5 and as means of technical duplicates (n = 2; mean ± SD) in Figure 2.

### Cell Viability Assays

To investigate the effects of the ML364 derivatives on healthy cells, monolayers of Green monkey Vero cells (RRID:CVCL_0059) were treated with each ML364 derivative at different concentrations to generate a 0.5 log dose‐response curve. Cells were incubated for 48 h and then fixed in 4% PFA. Cells were stained with 4′,6‐diamidino‐2‐phenylindole (DAPI) (5 µg/mL) for 10 min at room temperature and the fluorescence signal quantified using a plate reader (BMG Clariostar). Data were normalized to a DMSO control and cytotoxicity values (CC_50_) were calculated.

### Resazurin Assays

∼7000 MCF7 human cells (RRID:CVCL_0031; American Type Culture Collection (ATCC): CRL‐12584) were seeded in 100 µL DMEM media + 10%_v/v_ FBS per well in a 96‐well plate, in triplicate, for 6 h. For IFN‐α‐treated cells, IFN‐α (#11105‐1, final concentration 1000 U/mL of media) was added to the seeding media. After seeding, 0.8 µL of the relevant DMSO compound stock (DMSO only as a negative control, 1.25 mM, 2.5 mM, 5 mM or 10 mM) were added to the corresponding wells at the appropriate final concentration (0 µM, 10 µM, 20 µM, 40 µM or 80 µM, respectively), and the plate was incubated in a humidified incubator (37°C, 5% CO_2_) for 67 h. Following incubation, media in each well were replaced with fresh DMEM media (+ 10%_v/v_ FBS) containing resazurin (10 µg/mL final concentration, prepared from a 10 mg/mL resazurin PBS stock) and incubated for 270 min. The plate was then imaged in fluorescence mode using a plate reader (CLARIOstar, *λ*_ex_ = 545 nm, *λ*_em_ = 600 nm). Data were processed using GraphPad Prism (v. 10.2.3) by averaging all replicates for each condition and normalizing relative to DMSO controls, with the DMSO control intensity representing 100% cell viability. Viability curves were generated using the nonlinear regression curve fit (variable slope) built‐in function.

## Author Contributions

Conceptualization: LB; methodology: SS, WT, LB; validation: SS, PACW, WT, SDD, AP‐F, MAW, FD, CJS, LB; investigation: SS, PACW, WT, SDD, LB; resources: TA‐C, ES, PL, CS‐D; visualization: SS, PACW, WT, SDD, LB; supervision: SB, AP‐F, MAW, FD, CJS, LB; writing – original draft: SS, CJS, LB; writing – review & editing: SS, PACW, WT, SDD, AP‐F, MAW, FD, CJS, LB.

## Conflicts of Interest

The authors declare no conflicts of interest.

## Supporting information




**Supporting File 1**: chem71248‐sup‐0001‐SuppMat.pdf

## Data Availability

The data that support the findings of this study are available in the supplementary material of this article. Relevant simulation input files, scripts, and final poses are also available in a GitHub repository (https://github.com/duartegroup/selective‐plpro‐inhibition).

## References

[1] P. V'kovski , A. Kratzel , S. Steiner , H. Stalder , and V. Thiel , “Coronavirus Biology and Replication: Implications for SARS‐CoV‐2,” Nature Reviews Microbiology 19 (2021): 155–170. 10.1038/s41579-020-00468-6 PMC7592455 33116300

[2] E. Hartenian , D. Nandakumar , A. Lari , M. Ly , J. M. Tucker , and B. A. Glaunsinger , “The Molecular Virology of Coronaviruses,” Journal of Biological Chemistry 295 (2020): 12910–12934. 10.1074/jbc.REV120.013930 PMC7489918 32661197

[3] S. Ullrich and C. Nitsche , “SARS‐CoV‐2 Papain‐Like Protease: Structure, Function and Inhibition,” ChemBioChem 23 (2022): e202200327. 10.1002/cbic.202200327 PMC9538446 35993805

[4] R. Cannalire , C. Cerchia , A. R. Beccari , F. S. Di Leva , and V. Summa , “Targeting SARS‐CoV‐2 Proteases and Polymerase for COVID‐19 Treatment: State of the Art and Future Opportunities,” Journal of Medicinal Chemistry 65 (2022): 2716–2746. 10.1021/acs.jmedchem.0c01140 PMC7688049 33186044

[5] G. Li , R. Hilgenfeld , R. Whitley , and E. De Clercq , “Therapeutic Strategies for COVID‐19: Progress and Lessons Learned,” Nature Review Drug Discovery 22 (2023): 449–475. 10.1038/s41573-023-00672-y PMC10113999 37076602

[6] A.‐T. Ton , M. Pandey , J. R. Smith , F. Ban , M. Fernandez , and A. Cherkasov , “Targeting SARS‐CoV‐2 Papain‐Like Protease in the Postvaccine Era,” Trends in Pharmacological Sciences 43 (2022): 906–919. 10.1016/j.tips.2022.08.008 PMC9399131 36114026

[7] H. Tan , Y. Hu , P. Jadhav , B. Tan , and J. Wang , “Progress and Challenges in Targeting the SARS‐CoV‐2 Papain‐Like Protease,” Journal of Medicinal Chemistry 65 (2022): 7561–7580. 10.1021/acs.jmedchem.2c00303 PMC9159073 35620927

[8] D. J. Calleja , G. Lessene , and D. Komander , “Inhibitors of SARS‐CoV‐2 PLpro,” Frontiers in Chemistry 10 (2022): 876212. 10.3389/fchem.2022.876212 PMC9086436 35559224

[9] L. Kerti and V. Frecer , “Design of Inhibitors of SARS‐CoV‐2 Papain‐Like Protease Deriving From GRL0617: Structure–activity Relationships,” Bioorganic & Medicinal Chemistry 113 (2024): 117909. 10.1016/j.bmc.2024.117909 39288705

[10] D. R. Owen , C. M. N. Allerton , A. S. Anderson , et al., “An Oral SARS‐CoV‐2 Mpro Inhibitor Clinical Candidate for the Treatment of COVID‐19,” Science 374 (2021): 1586–1593. 10.1126/science.abl4784 34726479

[11] Y. Unoh , S. Uehara , K. Nakahara , et al., “Discovery of S‐217622, a Noncovalent Oral SARS‐CoV‐2 3CL Protease Inhibitor Clinical Candidate for Treating COVID‐19,” Journal of Medicinal Chemistry 65 (2022): 6499–6512. 10.1021/acs.jmedchem.2c00117 PMC8982737 35352927

[12] X. Jiang , H. Su , W. Shang , et al., “Structure‐based Development and Preclinical Evaluation of the SARS‐CoV‐2 3C‐Like Protease Inhibitor Simnotrelvir,” Nature Communications 14 (2023): 6463. 10.1038/s41467-023-42102-y PMC10575921 37833261

[13] X. Chen , X. Huang , Q. Ma , et al., “Preclinical Evaluation of the SARS‐CoV‐2 M^pro^ Inhibitor RAY1216 Shows Improved Pharmacokinetics Compared With Nirmatrelvir,” Nature Microbiology 9 (2024): 1075–1088. 10.1038/s41564-024-01618-9 PMC10994847 38553607

[14] G. Zhang , J. Mao , H. He , et al., “Discovery of GST‐HG171, a Potent and Selective Oral 3CL Protease Inhibitor for the Treatment of COVID‐19,” SM Journal of Infectious Diseases 6 (2023): 9.

[15] Y. M. Báez‐Santos , S. E. St John , and A. D. Mesecar , “The SARS‐coronavirus Papain‐Like Protease: Structure, Function and Inhibition by Designed Antiviral Compounds,” Antivirus Research 115 (2015): 21–38. 10.1016/j.antiviral.2014.12.015 PMC5896749 25554382

[16] D. Shin , R. Mukherjee , D. Grewe , et al., “Papain‐Like Protease Regulates SARS‐CoV‐2 Viral Spread and Innate Immunity,” Nature 587 (2020): 657–662. 10.1038/s41586-020-2601-5 PMC7116779 32726803

[17] T. Klemm , G. Ebert , D. J. Calleja , et al., “Mechanism and Inhibition of the Papain‐Like Protease, PLpro, of SARS‐CoV‐2,” EMBO Journal 39 (2020): e106275. 10.15252/embj.2020106275 PMC7461020 32845033

[18] S. M. Lange , L. A. Armstrong , and Y. Kulathu , “Deubiquitinases: From Mechanisms to Their Inhibition by Small Molecules,” Molecular Cell 82 (2022): 15–29. 10.1016/j.molcel.2021.10.027 34813758

[19] J. A. Harrigan , X. Jacq , N. M. Martin , and S. P. Jackson , “Deubiquitylating Enzymes and Drug Discovery: Emerging Opportunities,” Nature Review Drug Discovery 17 (2018): 57–78. 10.1038/nrd.2017.152 PMC7097658 28959952

[20] G. Dewson , P. J. A. Eichhorn , and D. Komander , “Deubiquitinases in Cancer,” Nature Reviews Cancer 23 (2023): 842–862. 10.1038/s41568-023-00633-y 37935888

[21] A. Y. Amerik and M. Hochstrasser , “Mechanism and Function of Deubiquitinating Enzymes,” Biochimica Et Biophysica Acta 1695 (2004): 189–207. 10.1016/j.bbamcr.2004.10.003 15571815

[22] D. C. Schwartz and M. Hochstrasser , “A Superfamily of Protein Tags: Ubiquitin, SUMO and Related Modifiers,” Trends in Biochemical Sciences 28 (2003): 321–328. 10.1016/S0968-0004(03)00113-0 12826404

[23] T. E. T. Mevissen and D. Komander , “Mechanisms of Deubiquitinase Specificity and Regulation,” Annual Review of Biochemistry 86 (2017): 159–192. 10.1146/annurev-biochem-061516-044916 28498721

[24] E. S. Johnson , “Protein Modification by SUMO,” Annual Review of Biochemistry 73 (2004): 355–382. 10.1146/annurev.biochem.73.011303.074118 15189146

[25] P. Liu , Z. Chen , Y. Guo , Q. He , and C. Pan , “Recent Advances in Small Molecule Inhibitors of Deubiquitinating Enzymes,” European Journal of Medicinal Chemistry 287 (2025): 117324. 10.1016/j.ejmech.2025.117324 39908798

[26] Y. Xiong , B. Huang , Y. Yang , et al., “The Substrate Selectivity of Papain‐Like Proteases From Human‐infecting Coronaviruses Correlates With Innate Immune Suppression,” Science Signaling 16 (2023): eade1985. 10.1126/scisignal.ade1985 37130166

[27] I. M. Gold , N. Reis , F. Glaser , and M. H. Glickman , “Coronaviral PLpro Proteases and the Immunomodulatory Roles of Conjugated versus Free Interferon Stimulated Gene Product‐15 (ISG15),” Seminars in Cell & Developmental Biology 132 (2022): 16–26. 10.1016/j.semcdb.2022.06.005 PMC9233553 35764457

[28] L. Ketscher , R. Hannß , D. J. Morales , et al., “Selective Inactivation of USP18 Isopeptidase Activity in Vivo Enhances ISG15 Conjugation and Viral Resistance,” Proceedings of the National Academy of Science USA 112 (2015): 1577–1582. 10.1073/pnas.1412881112 PMC4321242 25605921

[29] Y.‐C. Perng and D. J. Lenschow , “ISG15 in Antiviral Immunity and Beyond,” Nature Reviews Microbiology 16 (2018): 423–439. 10.1038/s41579-018-0020-5 PMC7097117 29769653

[30] P. M. Wydorski , J. Osipiuk , B. T. Lanham , et al., “Dual Domain Recognition Determines SARS‐CoV‐2 PLpro Selectivity for Human ISG15 and K48‐linked Di‐ubiquitin,” Nature Communications 14 (2023): 2366. 10.1038/s41467-023-38031-5 PMC10126577 37185902

[31] H. Lin , “Substrate‐selective Small‐molecule Modulators of Enzymes: Mechanisms and Opportunities,” Current Opinion in Chemical Biology 72 (2023): 102231. 10.1016/j.cbpa.2022.102231 PMC9870951 36455490

[32] D. N. Garad , X. Li , T. K. Chua , B. K. Moku , C. B. Mishra , and Y. Song , “Targeting the S1′ Pocket of SARS‐CoV‐2 Papain‐Like Protease Yields Highly Potent Inhibitors,” ACS Medicinal Chemistry Letters 16 (2025): 2280–2285. 10.1021/acsmedchemlett.5c00482 PMC12621062 41256988

[33] L. Brewitz , J. J. A. G. Kamps , P. Lukacik , et al., “Mass Spectrometric Assays Reveal Discrepancies in Inhibition Profiles for the SARS‐CoV‐2 Papain‐Like Protease,” ChemMedChem 17 (2022): e202200016. 10.1002/cmdc.202200016 PMC9015526 35085423

[34] L. Brewitz , H. T. Henry Chan , P. Lukacik , et al., “Mass Spectrometric Assays Monitoring the Deubiquitinase Activity of the SARS‐CoV‐2 Papain‐Like Protease Inform on the Basis of Substrate Selectivity and Have Utility for Substrate Identification,” Bioorganic & Medicinal Chemistry 95 (2023): 117498. 10.1016/j.bmc.2023.117498 PMC10933793 37857256

[35] Q. Yue , J. Hua‐Juan , Y. Yu‐Shun , H. Xiao‐Qin , and Z. Xue‐Wen , “Review of the Crystallized Structures of the SARS‐CoV‐2 Papain‐Like Protease,” Journal of Molecular Structure 1333 (2025): 141730.

[36] B. T. Freitas , I. A. Durie , J. Murray , et al., “Characterization and Noncovalent Inhibition of the Deubiquitinase and deISGylase Activity of SARS‐CoV‐2 Papain‐Like Protease,” ACS Infectious Diseases 6 (2020): 2099–2109. 10.1021/acsinfecdis.0c00168 32428392

[37] S. Patchett , Z. Lv , W. Rut , et al., “A Molecular Sensor Determines the Ubiquitin Substrate Specificity of SARS‐CoV‐2 Papain‐Like Protease,” Cell Reports 36 (2021): 109754. 10.1016/j.celrep.2021.109754 PMC8423903 34547223

[38] J. Zhang , J. Allen , S. J. Ward , L. V. Dekker , and I. Dreveny , “A Versatile Fluorescence Polarization Based Deubiquitination Assay Using an Isopeptide Bond Substrate Mimetic (IsoMim),” Journal of Biological Chemistry 301 (2025): 110342. 10.1016/j.jbc.2025.110342 PMC12273560 40482917

[39] C. Ma and J. Wang , “Validation and Invalidation of SARS‐CoV‐2 Papain‐Like Protease Inhibitors,” ACS Pharmacology and Translational Science 5 (2022): 102–109. 10.1021/acsptsci.1c00240 PMC8806001 35178512

[40] Z. Zhao , R. O'Dea , K. Wendrich , N. Kazi , and M. Gersch , “Native Semisynthesis of Isopeptide‐linked Substrates for Specificity Analysis of Deubiquitinases and Ubl Proteases,” Journal of the American Chemical Society 145 (2023): 20801–20812. 10.1021/jacs.3c04062 PMC10540217 37712884

[41] M. S. Ritorto , R. Ewan , A. B. Perez‐Oliva , et al., “Screening of DUB Activity and Specificity by MALDI‐TOF Mass Spectrometry,” Nature Communications 5 (2014): 4763. 10.1038/ncomms5763 PMC4147353 25159004

[42] L. A. Armstrong , S. M. Lange , V. Dee Cesare , et al., “Biochemical Characterization of Protease Activity of Nsp3 From SARS‐CoV‐2 and Its Inhibition by Nanobodies,” PLoS One 16 (2021): e0253364. 10.1371/journal.pone.0253364 PMC8284666 34270554

[43] T. R. Malla , A. Tumber , T. John , et al., “Mass Spectrometry Reveals Potential of β‐Lactams as SARS‐CoV‐2 Mpro Inhibitors,” Chemical Communications 57 (2021): 1430–1433. 10.1039/d0cc06870e PMC8006714 33462575

[44] M. A. Redhead , C. D. Owen , L. Brewitz , et al., “Bispecific Repurposed Medicines Targeting the Viral and Immunological Arms of COVID‐19,” Scientific Reports 11 (2021): 13208. 10.1038/s41598-021-92416-4 PMC8225628 34168183

[45] S. T. D. Thun‐Hohenstein , T. F. Suits , T. R. Malla , et al., “Structure‐activity Studies Reveal Scope for Optimisation of Ebselen‐type Inhibition of SARS‐CoV‐2 Main Protease,” ChemMedChem 17 (2022): e202100582. 10.1002/cmdc.202100582 PMC9015279 34850566

[46] T. R. Malla , L. Brewitz , D.‐G. Muntean , et al., “Penicillin Derivatives Inhibit the SARS‐CoV‐2 Main Protease by Reaction With Its Nucleophilic Cysteine,” Journal of Medicinal Chemistry 65 (2022): 7682–7696. 10.1021/acs.jmedchem.1c02214 PMC9115881 35549342

[47] L. Brewitz , L. Dumjahn , Y. Zhao , et al., “Alkyne Derivatives of SARS‐CoV‐2 Main Protease Inhibitors Including Nirmatrelvir Inhibit by Reacting Covalently With the Nucleophilic Cysteine,” Journal of Medicinal Chemistry 66 (2023): 2663–2680. 10.1021/acs.jmedchem.2c01627 PMC9924091 36757959

[48] B. Mahjour , R. Zhang , Y. Shen , et al., “Rapid Planning and Analysis of High‐throughput Experiment Arrays for Reaction Discovery,” Nature Communications 14 (2023): 3924. 10.1038/s41467-023-39531-0 PMC10318092 37400469

[49] M. de Munnik , J. Lithgow , L. Brewitz , et al., “αβ,α′β′‐Diepoxyketones Are Mechanism‐based Inhibitors of Nucleophilic Cysteine Enzymes,” Chemical Communications 59 (2023): 12859–12862. 10.1039/d3cc02932h PMC10601815 37815791

[50] L. B. Gayatri , L. Ibbotson , E. Salah , S. Basak , H. Choudhry , and C. J. Schofield , “Thiophene‐fused γ‐Lactams Inhibit the SARS‐CoV‐2 Main Protease via Reversible Covalent Acylation,” Chemical Science 15 (2024): 7667–7678. 10.1039/d4sc01027b PMC11110133 38784729

[51] D. Laczi , S. S. Huamán , T. Andrews‐Clark , et al., “Silaproline‐bearing Nirmatrelvir Derivatives Are Potent Inhibitors of the SARS‐CoV‐2 Main Protease Highlighting the Value of Silicon‐derivatives in Structure‐activity‐relationship Studies,” European Journal of Medicinal Chemistry 291 (2025): 117603. 10.1016/j.ejmech.2025.117603 40220677

[52] D.‐G. Muntean , W. Treyde , L. Kinena , et al., “C6‐Alkoxy Substituted Penicillins Are Potent Non‐covalently Binding Inhibitors of the SARS‐CoV‐2 Main Protease,” RSC Medicinal Chemistry 16 (2025): 6351–6367. 10.1039/d5md00789e PMC12588090 41199995

[53] M. Kawai , T. R. Malla , H. T. H. Chan , et al., “RaPID Discovery of Cell‐permeable Helical Peptide Inhibitors Containing Cyclic β‐amino Acids Against SARS‐CoV‐2 Main Protease,” RSC Chemical Biology 6 (2025): 1089–1099. 10.1039/d5cb00021a PMC12093385 40406165

[54] J.‐H. Zhang , T. D. Y. Chung , and K. R. Oldenburg , “A Simple Statistical Parameter for Use in Evaluation and Validation of High Throughput Screening Assays,” Journal of Biomolecular Screening 4 (1999): 67–73. 10.1177/108705719900400206 10838414

[55] K. Ratia , S. Pegan , J. Takayama , et al., “A Noncovalent Class of Papain‐Like Protease/Deubiquitinase Inhibitors Blocks SARS Virus Replication,” Proceedings of the National Academy of Science USA 105 (2008): 16119–16124. 10.1073/pnas.0805240105 PMC2571001 18852458

[56] Z. Fu , B. Huang , J. Tang , et al., “The Complex Structure of GRL0617 and SARS‐CoV‐2 PLpro Reveals a Hot Spot for Antiviral Drug Discovery,” Nature Communications 12 (2021): 488. 10.1038/s41467-020-20718-8 PMC7817691 33473130

[57] Y. Zhao , X. Du , Y. Duan , et al., “High‐throughput Screening Identifies Established Drugs as SARS‐CoV‐2 PLpro Inhibitors,” Protein Cell 12 (2021): 877–888. 10.1007/s13238-021-00836-9 PMC8052528 33864621

[58] Z. Shen , K. Ratia , L. Cooper , et al., “Design of SARS‐CoV‐2 PLpro Inhibitors for COVID‐19 Antiviral Therapy Leveraging Binding Cooperativity,” Journal of Medicinal Chemistry 65 (2022): 2940–2955. 10.1021/acs.jmedchem.1c01307 PMC8547495 34665619

[59] M. Altun , H. B. Kramer , L. I. Willems , et al., “Activity‐based Chemical Proteomics Accelerates Inhibitor Development for Deubiquitylating Enzymes,” Chemistry & Biology 18 (2011): 1401–1412. 10.1016/j.chembiol.2011.08.018 22118674

[60] C.‐C. Cho , S. G. Li , T. J. Lalonde , et al., “Drug Repurposing for the SARS‐CoV‐2 Papain‐Like Protease,” ChemMedChem 17 (2022): e202100455. 10.1002/cmdc.202100455 PMC8653067 34423563

[61] P. D'Arcy , S. Brnjic , M. H. Olofsson , et al., “Inhibition of Proteasome Deubiquitinating Activity as a New Cancer Therapy,” Nature Medicine 17 (2011): 1636–1640. 10.1038/nm.2536 22057347

[62] X. Wang , P. D'Arcy , T. R. Caulfield , et al., “Synthesis and Evaluation of Derivatives of the Proteasome Deubiquitinase Inhibitor b‐AP15,” Chemical Biology and Drug Design 86 (2015): 1036–1048. 10.1111/cbdd.12571 PMC4846425 25854145

[63] E. Aleo , C. J. Henderson , A. Fontanini , B. Solazzo , and C. Brancolini , “Identification of New Compounds That Trigger Apoptosome‐independent Caspase Activation and Apoptosis,” Cancer Research 66 (2006): 9235–9244. 10.1158/0008-5472.CAN-06-0702 16982768

[64] V. Kapuria , L. F. Peterson , D. Fang , W. G. Bornmann , M. Talpaz , and N. J. Donato , “Deubiquitinase Inhibition by Small‐molecule WP1130 Triggers Aggresome Formation and Tumor Cell Apoptosis,” Cancer Research 70 (2010): 9265–9276. 10.1158/0008-5472.CAN-10-1530 21045142

[65] L. F. Peterson , H. Sun , Y. Liu , et al., “Targeting Deubiquitinase Activity With a Novel Small‐molecule Inhibitor as Therapy for B‐cell Malignancies,” Blood 125 (2015): 3588–3597. 10.1182/blood-2014-10-605584 25814533

[66] M. I. Davis , R. Pragani , J. T. Fox , et al., “Small Molecule Inhibition of the Ubiquitin‐specific Protease USP2 Accelerates Cyclin D1 Degradation and Leads to Cell Cycle Arrest in Colorectal Cancer and Mantle Cell Lymphoma Models,” Journal of Biological Chemistry 291 (2016): 24628–24640. 10.1074/jbc.M116.738567 PMC5114414 27681596

[67] Q. Liang , T. S. Dexheimer , P. Zhang , et al., “A Selective USP1–UAF1 Inhibitor Links Deubiquitination to DNA Damage Responses,” Nature Chemical Biology 10 (2014): 298–304. 10.1038/nchembio.1455 PMC4144829 24531842

[68] C. Reverdy , S. Conrath , R. Lopez , et al., “Discovery of Specific Inhibitors of Human USP7/HAUSP Deubiquitinating Enzyme,” Chemistry & Biology 19 (2012): 467–477. 10.1016/j.chembiol.2012.02.007 22520753

[69] X. Tian , N. S. Isamiddinova , R. J. Peroutka , et al., “Characterization of Selective Ubiquitin and Ubiquitin‐Like Protease Inhibitors Using a Fluorescence‐based Multiplex Assay Format,” Assay and Drug Development Technologies 9 (2010): 165–173. 10.1089/adt.2010.0317 PMC3065724 21133675

[70] L. Kategaya , P. Di Lello , L. Rougé , et al., “USP7 Small‐molecule Inhibitors Interfere With Ubiquitin Binding,” Nature 550 (2017): 534–538. 10.1038/nature24006 29045385

[71] J. Liu , H. Xia , M. Kim , et al., “Beclin1 Controls the Levels of p53 by Regulating the Deubiquitination Activity of USP10 and USP13,” Cell 147 (2011): 223–234. 10.1016/j.cell.2011.08.037 PMC3441147 21962518

[72] B.‐H. Lee , M. J. Lee , S. Park , et al., “Enhancement of Proteasome Activity by a Small‐molecule Inhibitor of USP14,” Nature 467 (2010): 179–184. 10.1038/nature09299 PMC2939003 20829789

[73] M. Boselli , B.‐H. Lee , J. Robert , et al., “An Inhibitor of the Proteasomal Deubiquitinating Enzyme USP14 Induces Tau Elimination in Cultured Neurons,” Journal of Biological Chemistry 292 (2017): 19209–19225. 10.1074/jbc.M117.815126 PMC5702663 28972160

[74] M. Kemp in , Chapter Three—Recent Advances in the Discovery of Deubiquitinating Enzyme Inhibitors, ed. G. Lawton and D. R. Witty (Elsevier, 2016), 149–192. 10.1016/bs.pmch.2015.10.002 PMC7112275 26852935

[75] J. D. Wrigley , G. Gavory , I. Simpson , et al., “Identification and Characterization of Dual Inhibitors of the USP25/28 Deubiquitinating Enzyme Subfamily,” ACS Chemical Biology 12 (2017): 3113–3125. 10.1021/acschembio.7b00334 29131570

[76] A. F. Kluge , B. R. Lagu , P. Maiti , et al., “Novel Highly Selective Inhibitors of Ubiquitin Specific Protease 30 (USP30) Accelerate Mitophagy,” Bioorganic & Medicinal Chemistry Letters 28 (2018): 2655–2659. 10.1016/j.bmcl.2018.05.013 29935771

[77] Y. Liu , H. A. Lashuel , S. Choi , et al., “Discovery of Inhibitors That Elucidate the Role of UCH‐L1 Activity in the H1299 Lung Cancer Cell Line,” Chemistry & Biology 10 (2003): 837–846. 10.1016/j.chembiol.2003.08.010 14522054

[78] L. A. Carpino , “1‐Hydroxy‐7‐azabenzotriazole. An Efficient Peptide Coupling Additive,” Journal of the American Chemical Society 115 (1993): 4397–4398.

[79] S. Hünig and M. Kiessel , “Spezifische Protonenacceptoren als Hilfsbasen bei Alkylierungs‐ und Dehydrohalogenierungsreaktionen,” Chemische Berichte 91 (1958): 380–392.

[80] M. U. Mirza , S. Ahmad , I. Abdullah , and M. Froeyen , “Identification of Novel Human USP2 Inhibitor and Its Putative Role in Treatment of COVID‐19 by Inhibiting SARS‐CoV‐2 Papain‐Like (PLpro) Protease,” Computational Biology and Chemistry 89 (2020): 107376. 10.1016/j.compbiolchem.2020.107376 PMC7487165 32979815

[81] S. Iketani , H. Mohri , B. Culbertson , et al., “Multiple Pathways for SARS‐CoV‐2 Resistance to Nirmatrelvir,” Nature 613 (2023): 558–564. 10.1038/s41586-022-05514-2 PMC9849135 36351451

[82] J. D. Ip , A. Wing‐Ho Chu , W.‐M. Chan , et al., “Global Prevalence of SARS‐CoV‐2 3CL Protease Mutations Associated With Nirmatrelvir or Ensitrelvir Resistance,” eBioMedicine 91 (2023): 104559. 10.1016/j.ebiom.2023.104559 PMC10101811 37060743

[83] T. Sanderson , R. Hisner , I. A. Donovan‐Banfield , et al., “A Molnupiravir‐associated Mutational Signature in Global SARS‐CoV‐2 Genomes,” Nature 623 (2023): 594–600. 10.1038/s41586-023-06649-6 PMC10651478 37748513

[84] L. Brewitz and C. J. Schofield , “Fixing the Achilles Heel of Pfizer's Paxlovid for COVID‐19 Treatment,” Journal of Medicinal Chemistry 67 (2024): 11656–11661. 10.1021/acs.jmedchem.4c01342 PMC11284777 38967233

[85] M. Westberg , Y. Su , X. Zou , et al., “An Orally Bioavailable SARS‐CoV‐2 Main Protease Inhibitor Exhibits Improved Affinity and Reduced Sensitivity to Mutations,” Science Translational Medicine 16 (2024): eadi0979. 10.1126/scitranslmed.adi0979 PMC11193659 38478629

[86] E. Van Damme , P. Abeywickrema , Y. Yin , et al., “A Small‐molecule SARS‐CoV‐2 Inhibitor Targeting the Membrane Protein,” Nature 640 (2025): 506–513. 10.1038/s41586-025-08651-6 PMC11981937 40140563

[87] J. A. Newman , A. Douangamath , S. Yadzani , et al., “Structure, Mechanism and Crystallographic Fragment Screening of the SARS‐CoV‐2 NSP13 Helicase,” Nature Communications 12 (2021): 4848. 10.1038/s41467-021-25166-6 PMC8358061 34381037

[88] J. Chen , Y. Zhou , X. Wei , et al., “Development of Pan‐anti‐SARS‐CoV‐2 Agents Through Allosteric Inhibition of nsp14/nsp10 Complex,” ACS Infectious Diseases 10 (2024): 858–869. 10.1021/acsinfecdis.3c00356 37897418

[89] M. Laporte , D. Jochmans , D. Bardiot , et al., “A Coronavirus Assembly Inhibitor that Targets the Viral Membrane Protein,” Nature 640 (2025): 514–523. 10.1038/s41586-025-08773-x PMC11981944 40140569

[90] Y. Lu , Q. Yang , T. Ran , et al., “Discovery of Orally Bioavailable SARS‐CoV‐2 Papain‐Like Protease Inhibitor as a Potential Treatment for COVID‐19,” Nature Communications 15 (2024): 10169. 10.1038/s41467-024-54462-0 PMC11585628 39580525

[91] B. Tan , X. Zhang , A. Ansari , et al., “Design of a SARS‐CoV‐2 Papain‐Like Protease Inhibitor With Antiviral Efficacy in a Mouse Model,” Science 383 (2024): 1434–1440. 10.1126/science.adm9724 PMC12178660 38547259

[92] S. M. Bader , D. J. Calleja , S. M. Devine , et al., “A Novel PLpro Inhibitor Improves Outcomes in a Pre‐clinical Model of Long COVID,” Nature Communications 16 (2025): 2900. 10.1038/s41467-025-57905-4 PMC11969009 40180914

[93] M. R. Garnsey , M. C. Robinson , L. T. Nguyen , et al., “Discovery of SARS‐CoV‐2 Papain‐Like Protease (PLpro) Inhibitors With Efficacy in a Murine Infection Model,” Science Advances 10 (2024): eado4288. 10.1126/sciadv.ado4288 PMC11364104 39213347

[94] H. Tan , Q. Zhang , K. Georgiou , et al., “Identification of Naturally Occurring Drug‐resistant Mutations of SARS‐CoV‐2 Papain‐Like Protease,” Nature Communications 16 (2025): 4548. 10.1038/s41467-025-59922-9 PMC12084387 40379662

[95] X. Wu , M. Go , J. V. Nguyen , et al., “Mutational Profiling of SARS‐CoV‐2 Papain‐Like Protease Reveals Requirements for Function, Structure, and Drug Escape,” Nature Communications 15 (2024): 6219. 10.1038/s41467-024-50566-9 PMC11266423 39043718

[96] C. Wu , M. Ou , B. Qin , et al., “Discovery of 3‐phenyl‐1H‐5‐pyrazolylamides as PLpro Inhibitors Through Virtual Screening and Structure Optimization,” Bioorganic & Medicinal Chemistry Letters 127 (2025): 130293. 10.1016/j.bmcl.2025.130293 40473007

[97] A. J. Taylor , K. Amporndanai , T. A. Rietz , et al., “Fragment‐based Screen of SARS‐CoV‐2 Papain‐Like Protease (PLpro),” ACS Medicinal Chemistry Letters 15 (2024): 1351–1357. 10.1021/acsmedchemlett.4c00238 PMC11318101 39140055

[98] Q. Wei , A. J. Taylor , M. A. Barmade , et al., “Discovery of Fragment‐based Inhibitors of SARS‐CoV‐2 PLPro,” Journal of Medicinal Chemistry 69 (2026): 1419–1433. 10.1021/acs.jmedchem.5c02832 PMC12833870 41521555

[99] Q. Wei , A. J. Taylor , N. Miriyala , et al., “Discovery of Spiro[chromane‐2,4′‐piperidine] Derivatives as Irreversible Inhibitors of SARS‐CoV‐2 Papain‐Like Protease,” Journal of Medicinal Chemistry 69 (2026): 3588–3608. 10.1021/acs.jmedchem.5c03704 PMC12910659 41629155

[100] M. R. Gannarapu , D. Indukuri , C. Holberg , et al., “Discovery of the SARS‐CoV‐2 Papain‐Like Protease Inhibitor MR1–114: From Structure‐based Design to in Vivo Antiviral Efficacy,” Journal of Medicinal Chemistry 69 (2026): 8433–8450. 10.1021/acs.jmedchem.5c03846 PMC13071871 41886743

[101] K. Shinohara , T. Kobayakawa , K. Tsuji , Y. Takamatsu , H. Mitsuya , and H. Tamamura , “SARS‐CoV‐2 Papain‐Like Protease Inhibitors Based on Naphthalen‐1‐ylethanamine and Halogenated Benzene Moieties,” ACS Omega 10 (2025): 47165–47175. 10.1021/acsomega.5c05856 PMC12529399 41114162

[102] M. Sharafi , W. P. Teh , J. Green , et al., “Structure‐guided Design of Potent and Selective Covalent Inhibitors Targeting the SARS‐CoV‐2 Papain‐Like Protease,” Journal of Medicinal Chemistry 69 (2026): 2197–2214. 10.1021/acs.jmedchem.5c01973 41557701

[103] M. van Huizen , J. R. B.‐T. Horst , H. L. M. de Gruyter , et al., “Deubiquitinating Activity of SARS‐CoV‐2 Papain‐Like Protease Does Not Influence Virus Replication or Innate Immune Responses in Vivo,” PloS Pathogens 20 (2024): e1012100. 10.1371/journal.ppat.1012100 PMC10994560 38527094

[104] J. C. Ferreira , A. J. Villanueva , K. Al Adem , et al., “Identification of Novel Allosteric Sites of SARS‐CoV‐2 Papain‐Like Protease (PLpro) for the Development of COVID‐19 Antivirals,” Journal of Biological Chemistry 300 (2024): 107821. 10.1016/j.jbc.2024.107821 PMC11538808 39342997

[105] D. T. Berg , M. R. Wiley , and B. W. Grinnell , “Enhanced Protein C Activation and Inhibition of Fibrinogen Cleavage by a Thrombin Modulator,” Science 273 (1996): 1389–1391. 10.1126/science.273.5280.1389 8703074

[106] K. W. Gillman , J. E. Starrett Jr. , M. F. Parker , et al., “Discovery and Evaluation of BMS‐708163, a Potent, Selective and Orally Bioavailable γ‐Secretase Inhibitor,” AC Medicinal Chemistry Letters 1 (2010): 120–124. 10.1021/ml1000239 PMC4007960 24900185

[107] G. S. Basi , S. Hemphill , E. F. Brigham , et al., “Amyloid Precursor Protein Selective Gamma‐secretase Inhibitors for Treatment of Alzheimer's Disease,” Alzheimer's Research & Therapy 2 (2010): 36. 10.1186/alzrt60 PMC3031881 21190552

[108] K. C. Duggan , D. J. Hermanson , J. Musee , et al., “(R)‐Profens Are Substrate‐selective Inhibitors of Endocannabinoid Oxygenation by COX‐2,” Nature Chemical Biology 7 (2011): 803–809. 10.1038/nchembio.663 PMC3298755 22053353

[109] D. J. Hermanson , N. D. Hartley , J. Gamble‐George , et al., “Substrate‐selective COX‐2 Inhibition Decreases Anxiety via Endocannabinoid Activation,” Nature Neuroscience 16 (2013): 1291–1298. 10.1038/nn.3480 PMC3788575 23912944

[110] D. J. Hermanson , J. C. Gamble‐George , L. J. Marnett , and S. Patel , “Substrate‐selective COX‐2 Inhibition as a Novel Strategy for Therapeutic Endocannabinoid Augmentation,” Trends in Pharmacological Sciences 35 (2014): 358–367. 10.1016/j.tips.2014.04.006 PMC4074568 24845457

[111] F. Madoux , D. Dreymuller , J.‐P. Pettiloud , et al., “Discovery of an Enzyme and Substrate Selective Inhibitor of ADAM10 Using an Exosite‐binding Glycosylated Substrate,” Scientific Reports 6 (2016): 11. 10.1038/s41598-016-0013-4 PMC5431342 28442704

[112] B. Da'adoosh , K. Kaito , K. Miyashita , M. Sakaguchi , and A. Goldblum , “Computational Design of Substrate Selective Inhibition,” Plos Computational Biology 16 (2020): e1007713. 10.1371/journal.pcbi.1007713 PMC7112232 32196495

[113] K. L. Roche , S. Remiszewski , M. J. Todd , et al., “An Allosteric Inhibitor of Sirtuin 2 Deacetylase Activity Exhibits Broad‐spectrum Antiviral Activity,” Journal of Clinical Investigation 133 (2023): e158978. 10.1172/JCI158978 PMC10266789 37317966

[114] J. Charton , M. Gauriot , Q. Guo , et al., “Imidazole‐derived 2‐[N‐carbamoylmethyl‐alkylamino]acetic Acids, Substrate‐dependent Modulators of Insulin‐degrading Enzyme in Amyloid‐β Hydrolysis,” European Journal of Medicinal Chemistry 79 (2014): 184–193. 10.1016/j.ejmech.2014.04.009 PMC4128174 24735644

[115] J. P. Maianti , G. A. Tan , A. Vetere , et al., “Substrate‐selective Inhibitors That Reprogram the Activity of Insulin‐degrading Enzyme,” Nature Chemical Biology 15 (2019): 565–574. 10.1038/s41589-019-0271-0 PMC6551522 31086331

[116] A. Hennig , G. Ghale , and W. M. Nau , “Effects of Cucurbit[7]uril on Enzymatic Activity,” Chemical Communications (2007): 1614–1616. 10.1039/b618703j 17530077

[117] L. A. Logsdon and A. R. Urbach , “Sequence‐specific Inhibition of a Nonspecific Protease,” Journal of the American Chemical Society 135 (2013): 11414–11416. 10.1021/ja406032x 23883194

[118] X. Li , T. M. Palhano Zanela , E. S. Underbakke , and Y. Zhao , “Controlling Kinase Activities by Selective Inhibition of Peptide Substrates,” Journal of the American Chemical Society 143 (2021): 639–643. 10.1021/jacs.0c11566 33395291

[119] E. Faggi , Y. Pérez , S. V. Luis , and I. Alfonso , “Supramolecular Protection From the Enzymatic Tyrosine Phosphorylation in a Polypeptide,” Chemical Communications 52 (2016): 8142–8145. 10.1039/c6cc03875a 27271350

[120] T. Kodadek , “Inhibition of Proteolysis and Other Posttranslational Modifications With Substrate‐targeted Inhibitors,” Peptide Science 66 (2002): 134–140. 10.1002/bip.10233 12325163

[121] X. Li , K. Chen , and Y. Zhao , “Sequence‐selective Protection of Peptides From Proteolysis,” Angewandte Chemie International Edition 60 (2021): 11092–11097. 10.1002/anie.202102148 PMC8252432 33725413

[122] T. L. Kukar , T. B. Ladd , M. A. Bann , et al., “Substrate‐targeting γ‐SAecretase Modulators,” Nature 453 (2008): 925–929. 10.1038/nature07055 PMC2678541 18548070

[123] W. Rut , Z. Lv , M. Zmudzinski , et al., “Activity Profiling and Crystal Structures of Inhibitor‐bound SARS‐CoV‐2 Papain‐Like Protease: A Framework for Anti–COVID‐19 Drug Design,” Science Advances 6 (2020): eabd4596. 10.1126/sciadv.abd4596 PMC7567588 33067239

[124] B. Tan , X. Liang , A. Ansari , et al., “Structure‐based Design of Covalent SARS‐CoV‐2 Papain‐Like Protease Inhibitors,” Journal of Medicinal Chemistry 67 (2024): 20399–20420. 10.1021/acs.jmedchem.4c01872 PMC12182049 39499574

[125] B. C. Sanders , S. Pokhrel , A. D. Labbe , et al., “Potent and Selective Covalent Inhibition of the Papain‐Like Protease From SARS‐CoV‐2,” Nature Communications 14 (2023): 1733. 10.1038/s41467-023-37254-w PMC10044120 36977673

[126] X. Ji , A. L. Nielsen , and C. Heinis , “Cyclic Peptides for Drug Development,” Angewandte Chemie International Edition 63 (2024): e202308251. 10.1002/anie.202308251 37870189

[127] H. Zhang and S. Chen , “Cyclic Peptide Drugs Approved in the Last Two Decades (2001–2021),” RSC Chemical Biology 3 (2022): 18–31. 10.1039/d1cb00154j PMC8729179 35128405

[128] A. A. Vinogradov , Y. Yin , and H. Suga , “Macrocyclic Peptides as Drug Candidates: Recent Progress and Remaining Challenges,” Journal of the American Chemical Society 141 (2019): 4167–4181. 10.1021/jacs.8b13178 30768253

[129] N. Liu , Y. Zhang , Y. Lei , et al., “Design and Evaluation of a Novel Peptide–drug Conjugate Covalently Targeting SARS‐CoV‐2 Papain‐Like Protease,” Journal of Medicinal Chemistry 65 (2022): 876–884. 10.1021/acs.jmedchem.1c02022 34981929

[130] J. Johansen‐Leete , S. Ullrich , S. E. Fry , et al., “Antiviral Cyclic Peptides Targeting the Main Protease of SARS‐CoV‐2,” Chemical Science 13 (2022): 3826–3836. 10.1039/d1sc06750h PMC8966731 35432913

[131] S. Yin , S. Mei , Z. Li , et al., “Non‐covalent Cyclic Peptides Simultaneously Targeting Mpro and NRP1 Are Highly Effective Against Omicron BA.2.75,” Frontiers in Pharmacology 13 (2022): 1037993. 10.3389/fphar.2022.1037993 PMC9666779 36408220

[132] A. G. Kreutzer , M. Krumberger , E. M. Diessner , et al., “A Cyclic Peptide Inhibitor of the SARS‐CoV‐2 Main Protease,” European Journal of Medicinal Chemistry 221 (2021): 113530. 10.1016/j.ejmech.2021.113530 PMC8096527 34023738

[133] T. Miura , T. R. Malla , C. D. Owen , et al., “In Vitro Selection of Macrocyclic Peptide Inhibitors Containing Cyclic γ^2,4^‐Amino Acids Targeting the SARS‐CoV‐2 Main Protease,” Nature Chemistry 15 (2023): 998–1005. 10.1038/s41557-023-01205-1 PMC10322702 37217786

[134] K. Harrison , P. W. Carlos , S. Ullrich , et al., “Exploiting Hydrophobic Amino Acid Scanning to Develop Cyclic Peptide Inhibitors of the SARS‐CoV‐2 Main Protease With Antiviral Activity,” Chemistry ‐ A European Journal 30 (2024): e202401606. 10.1002/chem.202401606 38801240

[135] Y. Tan , J. Yang , M. Wang , et al., “De Novo Discovery of a Noncovalent Cell‐penetrating Bicyclic Peptide Inhibitor Targeting SARS‐CoV‐2 Main Protease,” Journal of Medicinal Chemistry 67 (2024): 20258–20274. 10.1021/acs.jmedchem.4c01639 39552553

[136] T. Miura , T. R. Malla , L. Brewitz , et al., “Cyclic β^2,3^‐Amino Acids Improve the Serum Stability of Macrocyclic Peptide Inhibitors Targeting the SARS‐CoV‐2 Main Protease,” Bulletin of the Chemical Society of Japan 97 (2024): uoae018. 10.1093/bulcsj/uoae018 PMC11141402 38828441

[137] S. Ullrich , V. M. Sasi , M. C. Mahawaththa , et al., “Challenges of Short Substrate Analogues as SARS‐CoV‐2 Main Protease Inhibitors,” Bioorganic & Medicinal Chemistry Letters 50 (2021): 128333. 10.1016/j.bmcl.2021.128333 PMC8378659 34418570

[138] Q. Wang , Y. Wang , J. Li , H. Liu , and S. Chen , “Bicyclic Peptide‐enhanced Covalent Inhibitor of SARS‐CoV‐2 3CL Protease,” Exploration of Drug Science 2 (2024): 719–733.

[139] M. Morgan , T. Ikenoue , H. Suga , and C. Wolberger , “Potent Macrocycle Inhibitors of the human SAGA Deubiquitinating Module,” Cell Chemical Biology 29 (2022): 544–554. 10.1016/j.chembiol.2021.12.004 PMC9035043 34936860

[140] R. Miranda , F. Anson , S. T. Smith , et al., “Discovery and Characterization of Potent Macrocycle Inhibitors of Ubiquitin‐specific Protease‐7,” Structure 33 (2025): 705–717. 10.1016/j.str.2025.01.021 39983720

[141] V. Di Sarno , G. Lauro , S. Musella , et al., “Identification of a Dual Acting SARS‐CoV‐2 Proteases Inhibitor Through in Silico Design and Step‐by‐step Biological Characterization,” European Journal of Medicinal Chemistry 226 (2021): 113863. 10.1016/j.ejmech.2021.113863 PMC8457654 34571172

[142] J. Chen , Y. Lin , C. Gao , et al., “Design, Synthesis and Activity Evaluation of Dual‐target Inhibitors Against Papain‐Like and Main Proteases of Porcine Epidemic Diarrhea Virus,” Journal of Advanced Research 80 (2026): 1009–1030. 10.1016/j.jare.2025.04.021 PMC12869208 40252827

[143] C. M. Daczkowski , O. Y. Goodwin , J. V. Dzimianski , J. J. Farhat , and S. D. Pegan , “Structurally Guided Removal of deISGylase Biochemical Activity From Papain‐Like Protease Originating From Middle East Respiratory Syndrome Coronavirus,” Journal of Virology 91 (2017): e01067–01117. 10.1128/JVI.01067-17 PMC5686735 28931677

[144] J. R. Clasman , R. K. Everett , K. Srinivasan , and A. D. Mesecar , “Decoupling deISGylating and Deubiquitinating Activities of the MERS Virus Papain‐Like Protease,” Antivirus Research 174 (2020): 104661. 10.1016/j.antiviral.2019.104661 PMC7114298 31765674

[145] M. Moustaqil , E. Ollivier , H.‐P. Chiu , et al., “SARS‐CoV‐2 Proteases PLpro and 3CLpro Cleave IRF3 and Critical Modulators of Inflammatory Pathways (NLRP12 and TAB1): Implications for Disease Presentation Across Species,” Emerging Microbes and Infections 10 (2021): 178–195. 10.1080/22221751.2020.1870414 PMC7850364 33372854

[146] N. D. Reynolds , N. M. Aceves , J. L. Liu , et al., “The SARS‐CoV‐2 SSHHPS Recognized by the Papain‐Like Protease,” ACS Infectious Diseases 7 (2021): 1483–1502. 10.1021/acsinfecdis.0c00866 PMC8171221 34019767

[147] Y. Mohamud , Y. C. Xue , H. Liu , et al., “The Papain‐Like Protease of Coronaviruses Cleaves ULK1 to Disrupt Host Autophagy,” Biochemical and Biophysical Research Communications 540 (2021): 75–82. 10.1016/j.bbrc.2020.12.091 PMC7836930 33450483

[148] B. Meyer , J. Chiaravalli , S. Gellenoncourt , et al., “Characterising Proteolysis During SARS‐CoV‐2 Infection Identifies Viral Cleavage Sites and Cellular Targets With Therapeutic Potential,” Nature Communications 12 (2021): 5553. 10.1038/s41467-021-25796-w PMC8455558 34548480

[149] H. T. H. Chan , L. Brewitz , P. Lukacik , et al., “Studies on the Selectivity of the SARS‐CoV‐2 Papain‐Like Protease Reveal the Importance of the P2′ Proline of the Viral Polyprotein,” RSC Chemical Biology 5 (2024): 117–130. 10.1039/d3cb00128h PMC10849127 38333195

[150] A. M. Mielech , A. Kilianski , Y. M. Baez‐Santos , A. D. Mesecar , and S. C. Baker , “MERS‐CoV Papain‐Like Protease Has deISGylating and Deubiquitinating Activities,” Virology 450–451 (2014): 64–70. 10.1016/j.virol.2013.11.040 PMC3957432 24503068

[151] N. Barretto , D. Jukneliene , K. Ratia , Z. Chen , A. D. Mesecar , and S. C. Baker , “The Papain‐Like Protease of Severe Acute Respiratory Syndrome Coronavirus Has Deubiquitinating Activity,” Journal of Virology 79 (2005): 15189–15198. 10.1128/JVI.79.24.15189-15198.2005 PMC1316023 16306590

[152] J. Lei , Y. Kusov , and R. Hilgenfeld , “Nsp3 of Coronaviruses: Structures and Functions of a Large Multi‐domain Protein,” Antiviral Research 149 (2018): 58–74. 10.1016/j.antiviral.2017.11.001 PMC7113668 29128390

[153] B. A. Bailey‐Elkin , R. C. M. Knaap , M. Kikkert , and B. L. Mark , “Structure and Function of Viral Deubiquitinating Enzymes,” Journal of Molecular Biology 429 (2017): 3441–3470. 10.1016/j.jmb.2017.06.010 PMC7094624 28625850

[154] M. P. Malakhov , O. A. Malakhova , K. I. Kim , K. J. Ritchie , and D.‐E. Zhang , “UBP43 (USP18) Specifically Removes ISG15 From Conjugated Proteins,” Journal of Biological Chemistry 277 (2002): 9976–9981. 10.1074/jbc.M109078200 11788588

[155] D. J. Fernández , S. Hess , and K.‐P. Knobeloch , “Strategies to Target ISG15 and USP18 Toward Therapeutic Applications,” Frontiers in Chemistry 7 (2020): 923. 10.3389/fchem.2019.00923 PMC6985271 32039148

[156] G. J. Davis , A. O. Omole , Y. Jung , et al., “Chemical Tools to Define and Manipulate Interferon‐inducible Ubl Protease USP18,” Nature Communications 16 (2025): 957. 10.1038/s41467-025-56336-5 PMC11754618 39843430

[157] National Center for Biotechnology Information (NCBI) reference sequence: NC_045512.2; https://www.ncbi.nlm.nih.gov/nuccore/NC_045512.

