# Supplementary material for: Substrate‐Selective Inhibition of the SARS‐CoV‐2 Papain‐Like Protease: Inhibition of Hydrolysis of Human Over Viral Substrates
Source: Chemistry. 2026 Jun 12;32(36):e71248. doi: 10.1002/chem.71248 (PMC13602897; doi:10.1002/chem.71248)
Supplement: Supplementary file 1 [file CHEM-32-e71248-s001.pdf]

Supporting Information

Substrate-Selective Inhibition of the SARS-CoV-2 Papain-like Protease: Inhibition of Hydrolysis of Human over Viral Substrates

Sakshi Sharma,[1] Peter A. C. Wing,[2] Wojtek Trede,[1] Shyam Basak,[1] Simeon D. Draganov,[2,3] Taylah Andrews-Clark,[1,4,5] Eidarus Salah,[1] Petra Lukacik,[4,5] Claire Strain-Damerell,[4,5] Adán Pinto-Fernández,[2,3] Martin A. Walsh,[4,5] Fernanda Duarte,[1] Christopher J. Schofield,*[1] and Lennart Brewitz*[1,6]

[1] Chemistry Research Laboratory and the Ineos Oxford Institute for Antimicrobial Research, University of Oxford, 12 Mansfield Road, OX1 3TA, Oxford, United Kingdom.

[2] Chinese Academy of Medical Sciences Oxford Institute, University of Oxford, Oxford, United Kingdom.

[3] Centre for Medicines Discovery, Nuffield Department of Medicine, University of Oxford, Oxford, United Kingdom.

[4] Diamond Light Source Ltd., Harwell Science and Innovation Campus, OX11 0DE, Didcot, United Kingdom.

[5] Research Complex at Harwell, Harwell Science and Innovation Campus, OX11 0FA, Didcot, United Kingdom.

[6] Centre for Chemical Biology, Institute of Physical Chemistry, Polish Academy of Sciences, Kasprzaka 44/52, 01-224 Warsaw, Poland.

*E-mail: lennart.brewitz@chem.ox.ac.uk or christopher.schofield@chem.ox.ac.uk

Table of content

1. Supplementary figures S2-S8
2. Supplementary tables S9-S16
3. Computational methods S17-S18
4. General information S19
5. General synthetic procedures S20
6. Synthetic procedures and analytical data S21-S41
7. References S42-S44
8. ^{1}H, ^{13}C and ^{19}F NMR spectra of synthesised ML364 derivatives S45-S110

1. Supplementary figures

Supplementary Figure S1. Robustness of the SPE-MS PL^{pro} inhibition assays using an N^{ε}-Lys-branched oligopeptide substrate. (**a**) Z'-factors[1] (circles) and (**b**) signal-to-noise ratios (S/N; boxes) for all SARS-CoV-2 PL^{pro} inhibition assay plates analysed to determine dose-response curves with the reported N^{ε}-Lys$_{193}$-branched IRF3$_{189\text{-}197}$-ISG15 peptide (**IRF3-ISG15**)[2] as the substrate. The Z'-factors >0.5 (grey line) indicate a stable and robust assay of high quality.[1] Z'-factors and S/N-ratios were determined using Microsoft Excel as reported.[1] SPE-MS inhibition assays were performed as described in the Experimental Section, using: SARS-CoV-2 PL^{pro} (0.2 μM), **IRF3-ISG15**[2] (2.0 μM), and the product standards: Ac-LSTVFMNLRLRGG-NH_2 (0.2 μM)[2] and Ac-ENPLKRLLV-NH_2 (0.2 μM)[2] in buffer (50 mM Tris, pH 8.0, 37 °C).

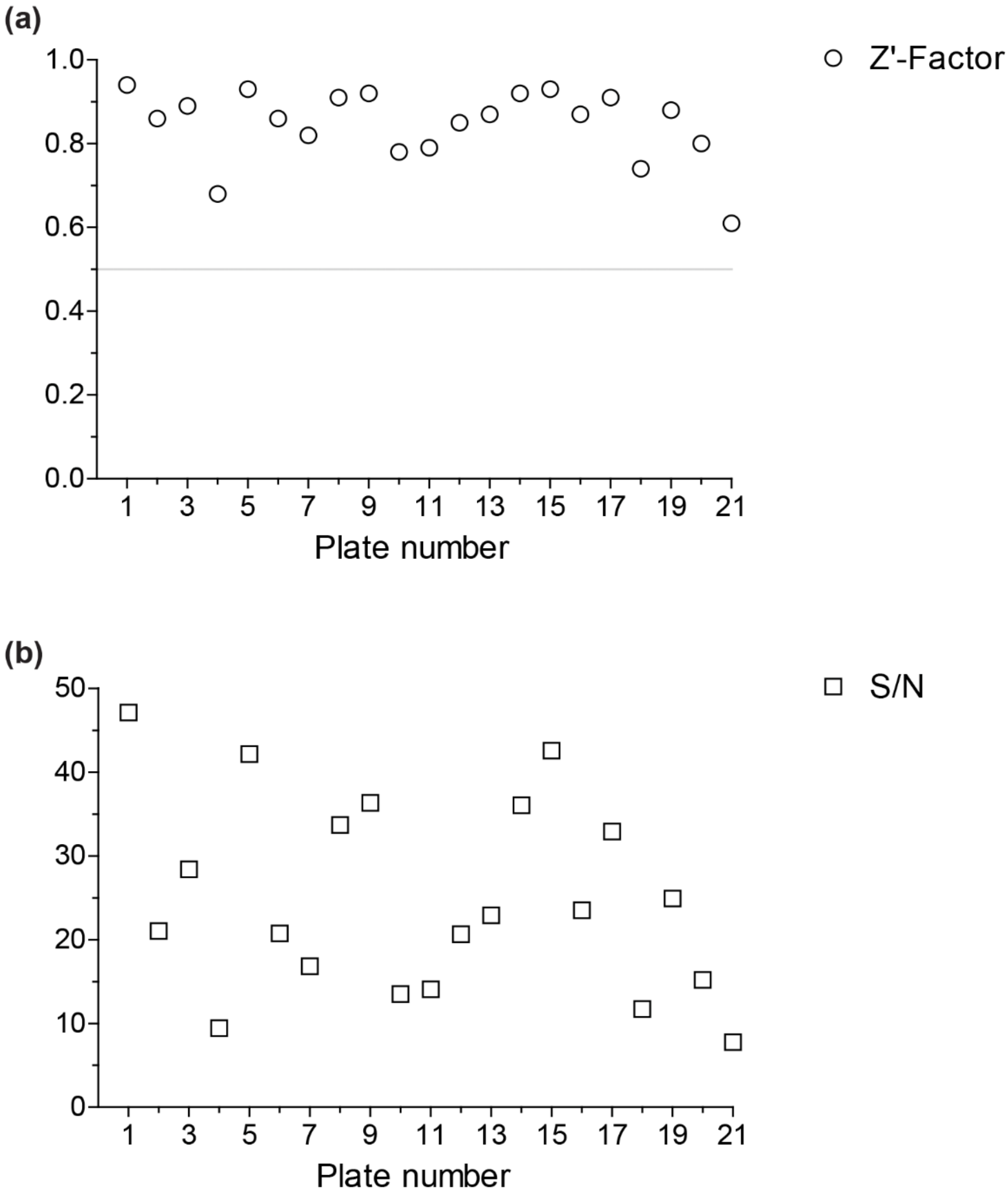

Supplementary Figure S2. Cytotoxicity of ML364 derivatives 9 and 18 toward MCF7 cells. (**a**, **c**) Viability of human MCF7 cells following treatment with the indicated concentrations of ML364 derivatives **9** and **18**, in the absence or presence of IFN-α, as determined by resazurin fluorescence assays (see Experimental Section). Data are means ± standard deviation (SD) of technical triplicates. (**b**, **d**) The corresponding dose-response curves.

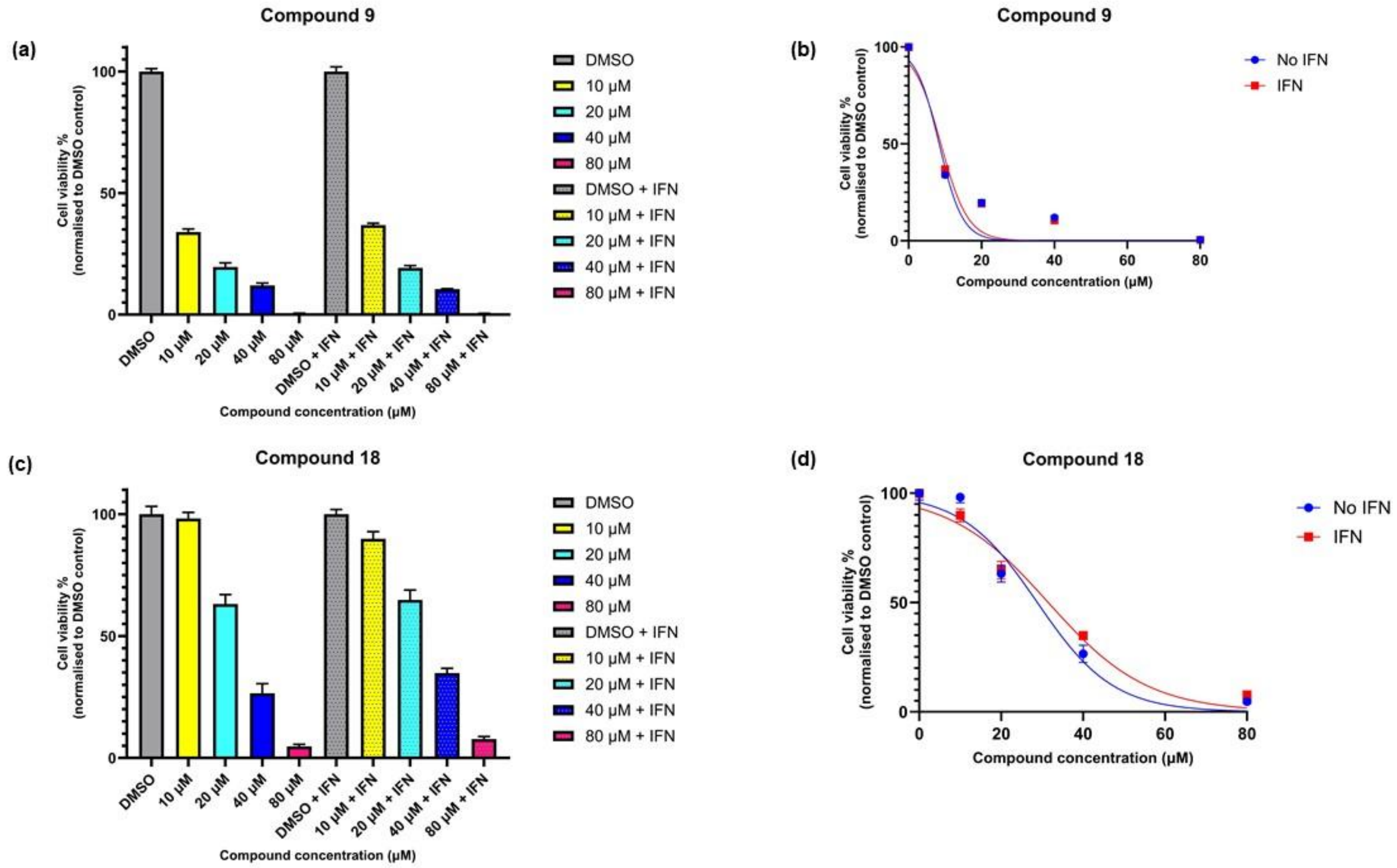

Supplementary Figure S3. Binding mode of ML364 derivative 32 at the PL^{pro} S sites from cluster analysis of three 100 ns MD simulations. (**a**) Computationally predicted binding mode of **32** to the S sites of SARS-CoV-2 PL^{pro} (Figure 4 shows binding to the S′ sites). (**b**) Interactions of **32** with residues in the S sites, including hydrogen bonding and π,π-stacking between the anthranilic acid group of **32** and the side chain of Y268. The sulfonamide naphthyl group of **32** is predicted to bind into the hydrophobic S4 pocket, formed *inter alia* by the hydrophobic side chains of M208, P247, P248, and Y264. (**c**) Overlay of a reported PL^{pro} C111S variant:Zn:ISG15 crystal structure (PDB ID: 7RBS[3]) with the PL^{pro}:Zn:**32** prediction suggests that this binding mode of **32** interferes with ISG15 binding and the binding of pp1a/1ab-derived substrates. (**d**) Overlay of the active site residues for the reported PL^{pro} C111S variant:Zn:ISG15 crystal structure (PDB ID: 7RBS[3]) and the PL^{pro}:Zn:**32** prediction further indicates that this binding mode of **32** interferes with binding of the C-terminal LRGG motif of ISG15 in the PL^{pro} S sites near the catalytic triad (C111, H272, D286).[4, 5] The sulfonamide naphthyl group of **32** is predicted to bind into the hydrophobic S4 pocket; this binding mode may result in substrate-independent PL^{pro} inhibition.

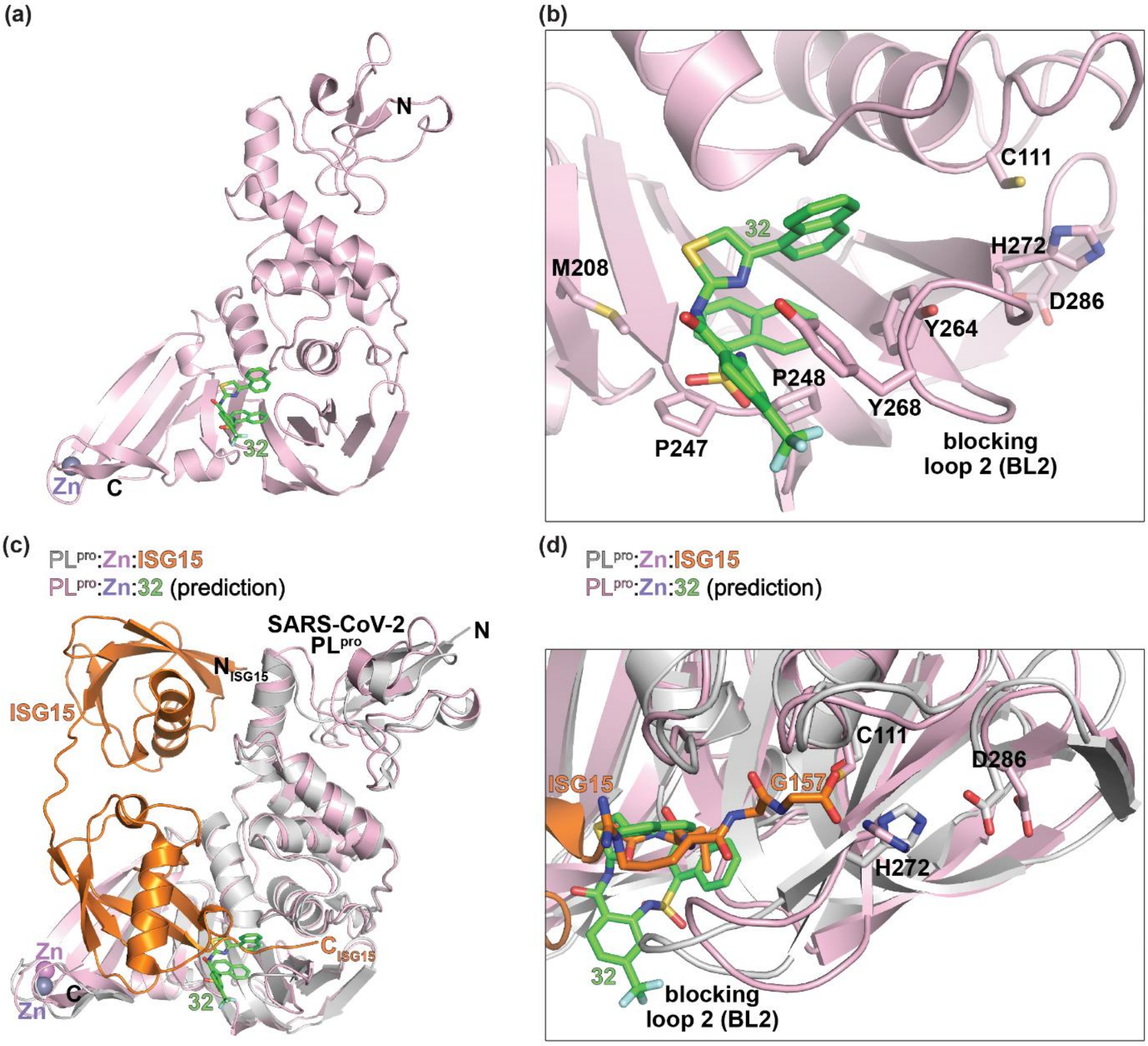

Supplementary Figure S4. The experimentally observed substrate-selective inhibition of ML364 derivative 32 is consistent with binding to the PL^{pro} S′ sites. (**a**) Superposition of the two alternative SARS-CoV-2 PL^{pro}:Zn:**32** predictions from cluster analysis of three 100 ns MD simulations indicates that the ML364 derivative **32** can bind to PL^{pro} on either side of the blocking loop 2 (BL2), binding either to the S (green) or S′ (cyan) sites. (**b** and **c**) Superposition of active site views from a reported PL^{pro} C111S variant:Zn:ISG15 crystal structure (PDB ID: 7RBS[3]) and the two PL^{pro}:Zn:**32** predictions suggest that **32** interferes with binding of the C-terminal LRGG motif of ISG15 in the PL^{pro} S sites near the catalytic triad (C111, H272, D286)[4, 5] when binding to the S sites (**b**), but not when binding to the S′ sites (**c**). The latter binding pose is therefore consistent with the experimentally observed substrate-selective PL^{pro} inhibition by **32** (Table 5). (**d**) Superposition of active site views from a reported computationally predicted PL^{pro}:**IRF3-ISG15** complex[2] and the PL^{pro}:Zn:**32** prediction suggests that binding of **32** (blue) to the S′ sites impairs binding of the N^{ε}-Lys branched (via Lys193; pink) IRF3-ISG15 substrate (yellow). Note, however, that human proteins post-translationally modified with ISG15 may adapt conformations at the PL^{pro} S′ sites that differ from those computationally predicted for **IRF3-ISG15**.

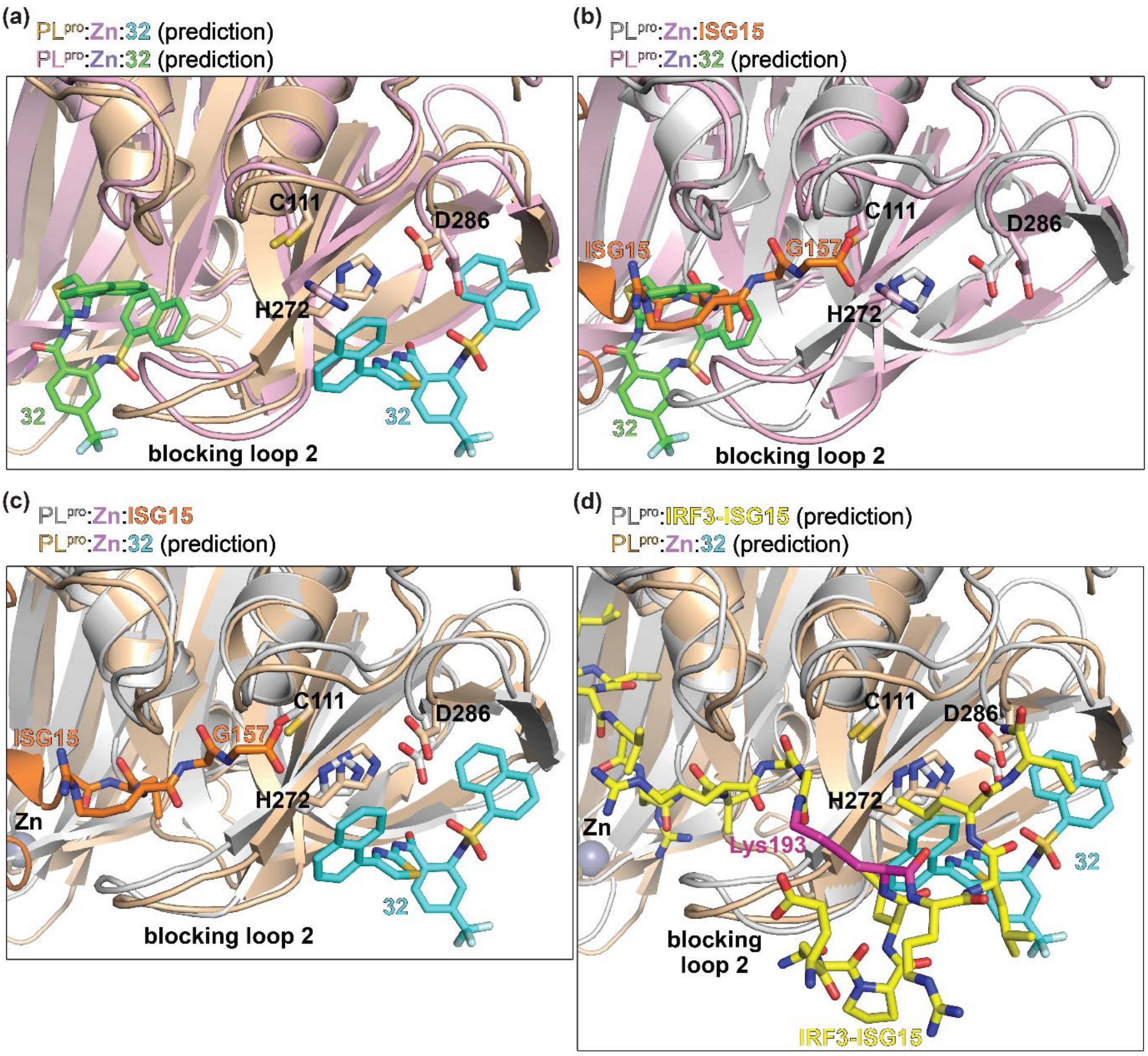

Supplementary Figure S5. Binding of ML364 derivative 32 at the PL^{pro} S′ sites does not interfere with the active site hydrogen bonding network. Distances between N$^{\varepsilon}$ of H272 and: (**a**) O$^{\delta1}$ and (**b**) O$^{\delta2}$ of D286, as well as between (**c**) N$^{\delta}$ of H272 and S of C111, respectively. (**d-f**) The corresponding hydrogen-hydrogen bond donor-hydrogen bond acceptor angles. H272 is positioned to hydrogen-bond with C111 and D286 for 33% and 50% of the simulation time, respectively.

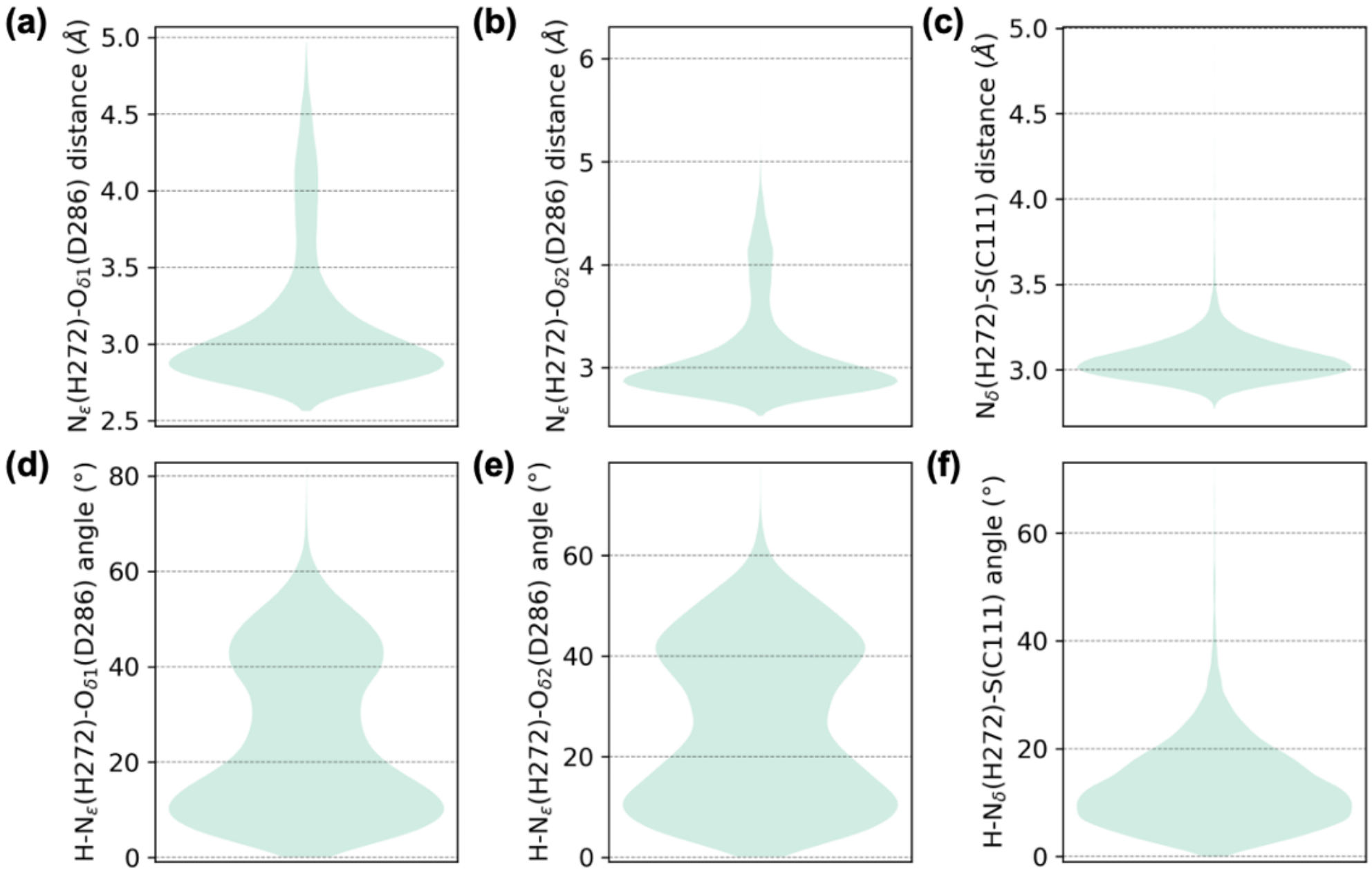

Supplementary Figure S6. Ligand root-mean-square deviation (RMSD) analysis indicates that the binding of ML364 derivative 32 to the PL^{pro} S′ sites is more stable than to the S sites. Ligand RMSD values relative to the cluster representative of the largest cluster for the ML364 derivative **32** bound to (**a**) the S sites and (**b**) the S′ sites of PL^{pro} over three independent replicas of 100 ns MD simulations.

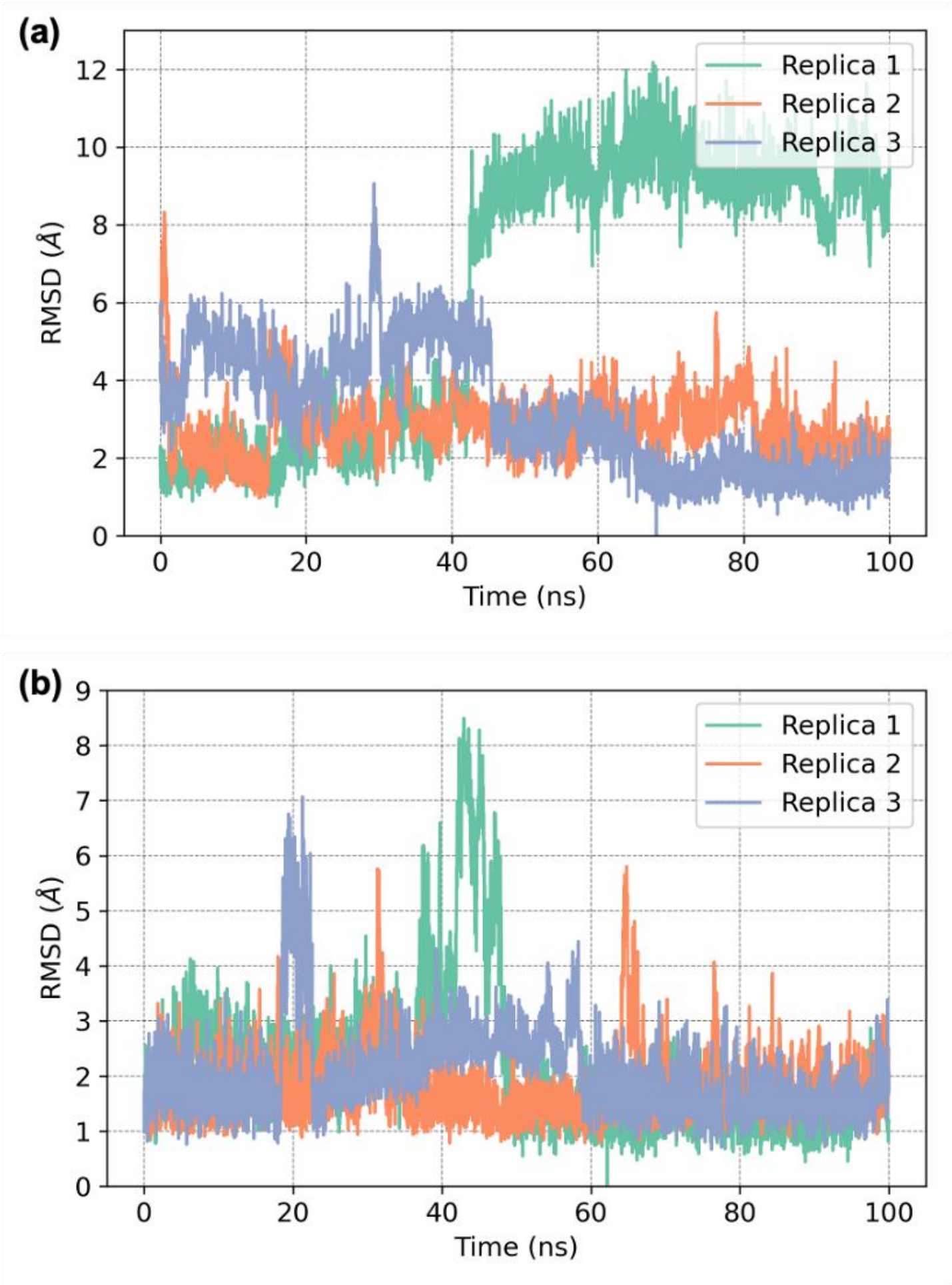

Supplementary Figure S7. Validation of PL^{pro} docking and MD simulations protocol using the crystallographically characterised inhibitor GRL0617. The PL^{pro} docking and MD simulations protocol was validated by docking the reported PL^{pro} inhibitor GRL0617[6] into the *holo* PL^{pro} structure (PDB ID: 7JIW[7]). (**a** and **b**) Superposition of the computationally predicted PL^{pro}:GRL0617 complexes with: (**a**) a neutral catalytic dyad and (**b**) a zwitterionic catalytic dyad from cluster analysis of three 100 ns MD simulations with the reported PL^{pro}:GRL0617 complex crystal structure (PDB ID: 7JRN[8]). The comparisons indicate similar but not identical binding modes of GRL0617 at the PL^{pro} active site. (**c** and **d**) The corresponding ligand RMSD values relative to the cluster representative of the largest cluster for GRL0617 bound to (**c**) neutral and (**d**) the zwitterionic PL^{pro} over three independent replicas of 100 ns MD simulations. The MD simulations indicate stable binding poses.

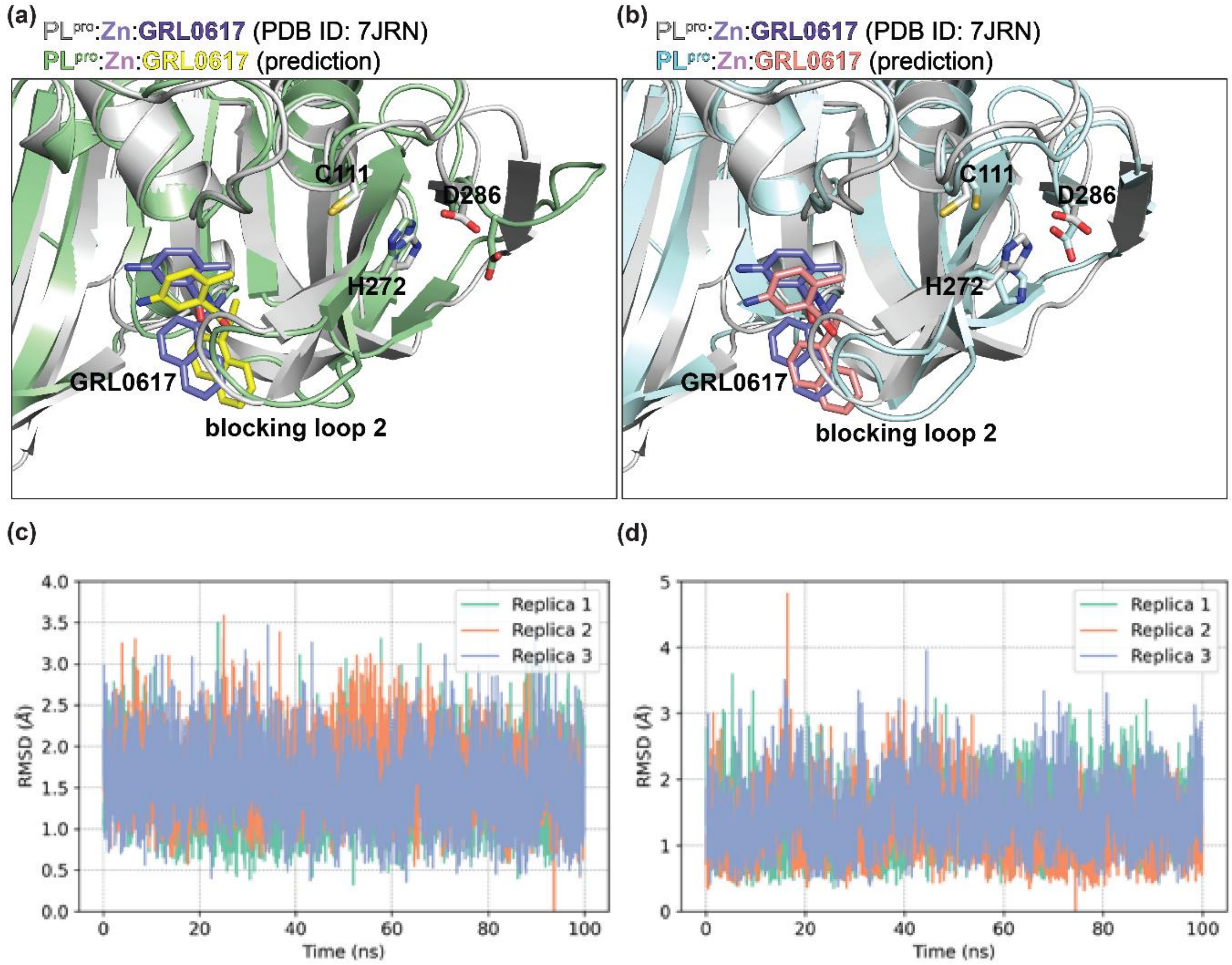

2. Supplementary tables

Supplementary Table S1. The effects of selected reported PL^{pro} inhibitors on catalysis are independent of the assay substrate (continues on the following page). SPE-MS inhibition assays were performed using isolated recombinant SARS-CoV-2 PL^{pro} with the linear pp1a/1ab-derived **nsp2/3$_{808-827}$** or the *N*$^{\varepsilon}$-Lys-branched **IRF3-ISG15** substrates.[2, 9]

The results reveal that the tested reported small-molecule PL^{pro} inhibitors show similar potency in SPE-MS assays, regardless of whether **nsp2/3$_{808-827}$** or **IRF3-ISG15** were used as the substrate. The results are consistent with our reported observations that some, but not all, of the reported small-molecule PL^{pro} inhibitors do not potently inhibit in the SPE-MS assays.[9] This observation possibly reflects the improved binding kinetics of the **nsp2/3$_{808-827}$** and **IRF3-ISG15** substrates compared to the relatively short and inefficient substrates typically used in fluorescence-based assays, *e.g.*, Ac-RLRGG-AMC (AMC: 7-amino-4-methylcoumarin).[9]

	**Reported PL^{pro} inhibitor**	a)**IC$_{50}$** [μM] reported using fluorescence-based assays	b)**IC$_{50}$** [μM] reported using SPE-MS and **nsp2/3$_{808-827}$**	c) **IC$_{50}$** [μM] using SPE-MS and **IRF3-ISG15**
i	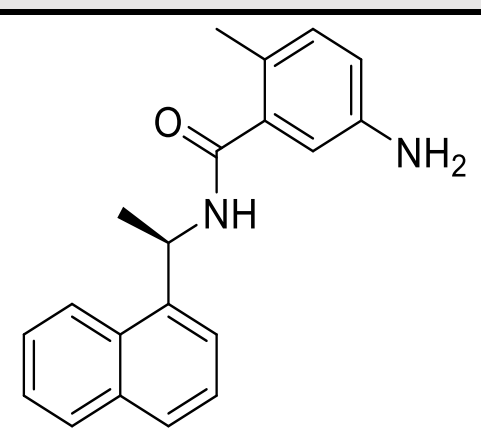<br><br>**GRL0617**	2.21[10]<br>2.4 ± 0.2[11]<br>2.1 ± 0.2[12]<br>1.61 ± 0.09[13]	3.8 ± 1.0[9]	2.9 ± 0.4
ii	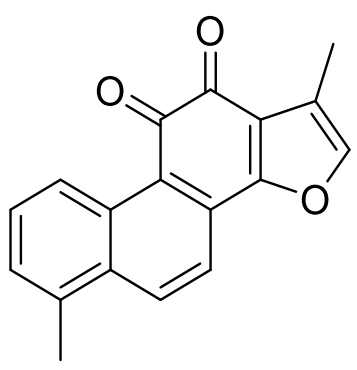<br><br>**tanshinone I**	2.21 ± 0.10[14]	>50[9]	>50
iii	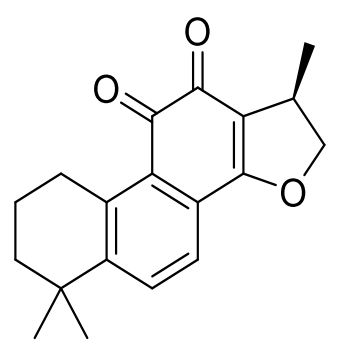<br><br>**cryptotanshinone**	5.63 ± 1.45[14]	19.4 ± 8.2[9]	12 ± 2
iv	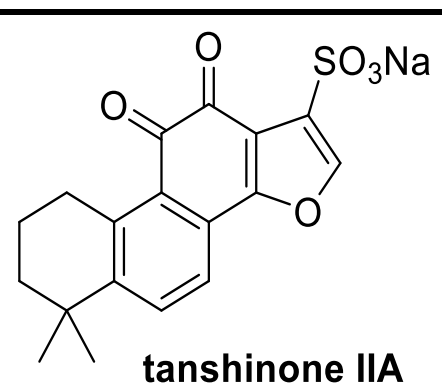<br><br>**tanshinone IIA sulfonate sodium**	1.65 ± 0.13[15]	>50[9]	42 ± 28

	Reported PL^{pro} inhibitor	a) IC_{50} [µM] reported using fluorescence-based assays	b) IC_{50} [µM] reported using SPE-MS and **nsp2/3$_{808-827}$**	c) IC_{50} [µM] using SPE-MS and **IRF3-ISG15**
v	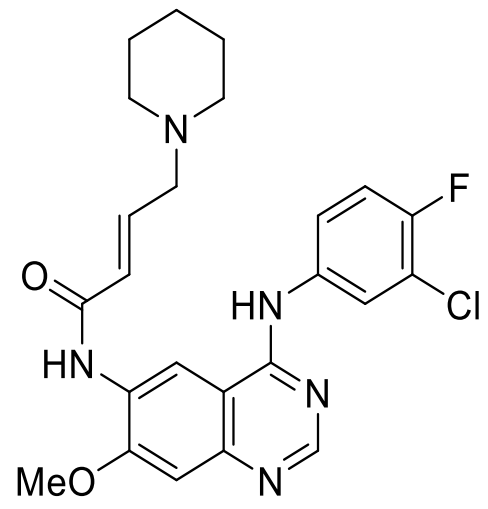 **dacomitinib**	3 – 5[16]	>50[9]	>50
vi	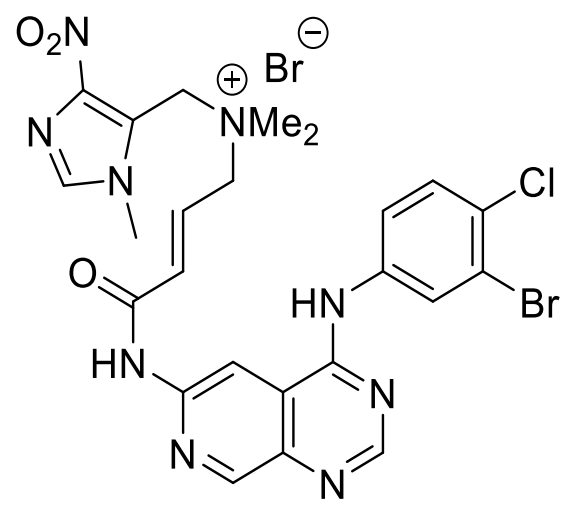 **tarloxotinib bromide**	0.1 – 0.5[16]	>50[9]	>50
vii	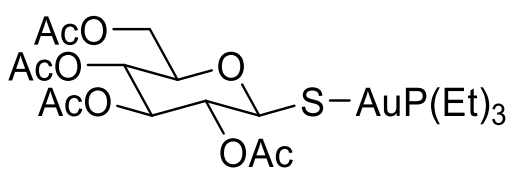 **auranofin**	1.08[10]<br>0.75 ± 0.13[17]	0.4 ± 0.1[9]	0.26 ± 0.16
viii	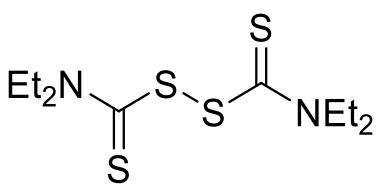 **disulfiram**	0.69[10]<br>1.05 ± 0.34[17]	0.5 ± 0.1[9]	0.24 ± 0.05
ix	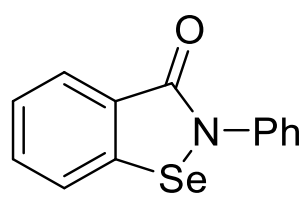 **ebselen**	2.26 ± 1.05[18]<br>1.12 ± 0.06[19]	0.3 ± 0.1[9]	0.13 ± 0.07

a) Reported IC_{50}-values were obtained using fluorescence-based assays which monitor the PL^{pro}-catalysed hydrolysis of Ac-RLRGG-AMC, RLRGG-AMC, ISG15-AMC or related substrates, as described in the cited literature. AMC: 7-amino-4-methylcoumarin; b) the reported results were determined using SPE-MS;[9] conditions: SARS-CoV-2 PL^{pro} (0.2 µM), **nsp2/3$_{808-827}$** (VTNNTFTLKGGAPTKVTFGI-NH_2;[9] 2.0 µM), and the product standards: Ac-VTNNTFTLKGG-NH_2 (0.2 µM)[9] and Ac-APTKVTFGI-NH_2 (0.2 µM)[9] in buffer (50 mM Tris, pH 8.0, 37 °C);[9] c) SPE-MS inhibition assays were performed using: SARS-CoV-2 PL^{pro} (0.2 µM), **IRF3-ISG15**[2] (2.0 µM), and the product standards: Ac-LSTVFMNLRLRGG-NH_2 (0.2 µM)[2] and Ac-ENPLKRLLV-NH_2 (0.2 µM)[2] in buffer (50 mM Tris, pH 8.0, 37 °C). Inhibitors were commercially sourced and used as received. Results are means of three independent runs (n = 3; mean ± standard deviation, SD).

Supplementary Table S2. ML364 derivatives with a *para*-chlorophenyl group at the thiazole C4 position can be more potent SARS-CoV-2 PL^{pro} inhibitors than those lacking the chloro substituent. SPE-MS inhibition assays were performed using isolated recombinant SARS-CoV-2 PL^{pro} with the linear pp1a/1ab-derived **nsp2/3$_{808-827}$** or the N^{ε}-Lys-branched **IRF3-ISG15** substrates,[2, 9] as described.

	**ML364 derivatives**	a)**IC$_{50}$** [µM] (using linear **nsp2/3$_{808-827}$**)	b)**IC$_{50}$** [µM] (using N^{ε}-Lys-branched **IRF3-ISG15**)		**ML364 derivatives**	a)**IC$_{50}$** [µM] (using linear **nsp2/3$_{808-827}$**)	b)**IC$_{50}$** [µM] (using N^{ε}-Lys-branched **IRF3-ISG15**)
i	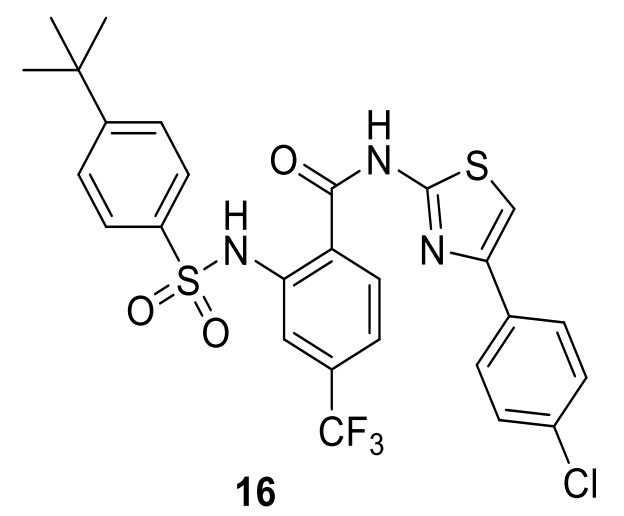 **16**	>50	13 ± 6	v	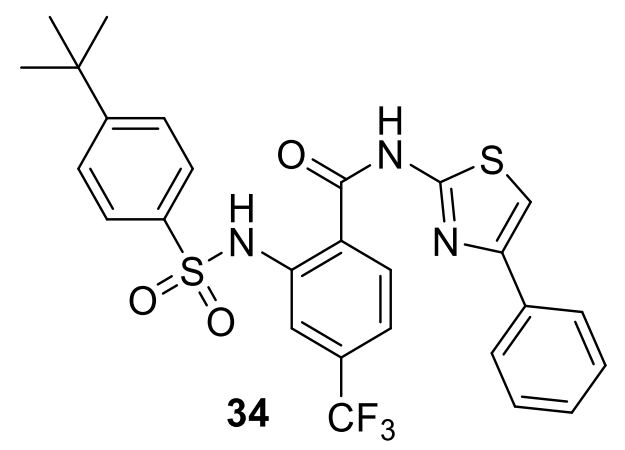 **34**	>50	>50
ii	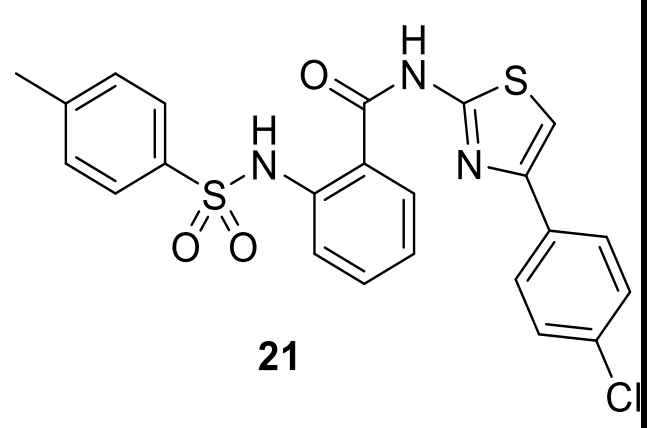 **21**	>50	20 ± 1	vi	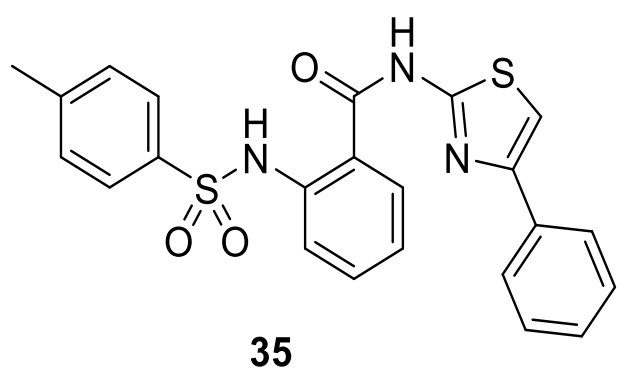 **35**	>50	>50
iii	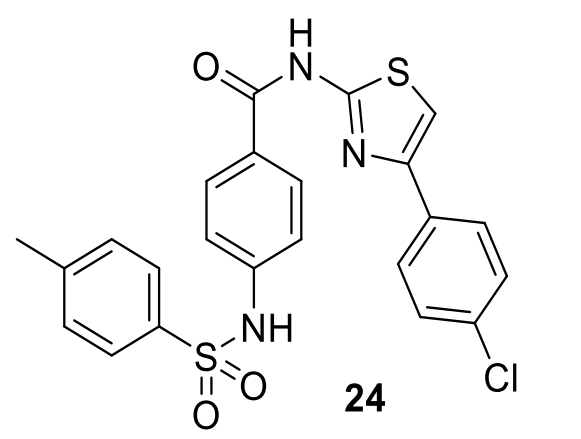 **24**	>50	>50	vii	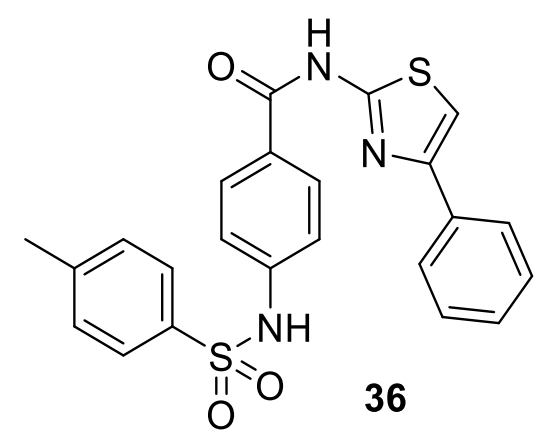 **36**	>50	>50
iv	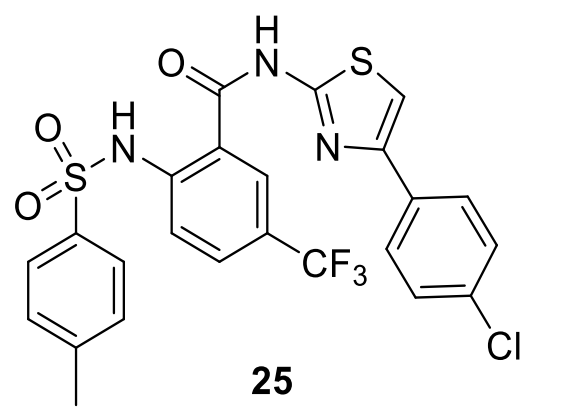 **25**	>50	4.5 ± 0.3	viii	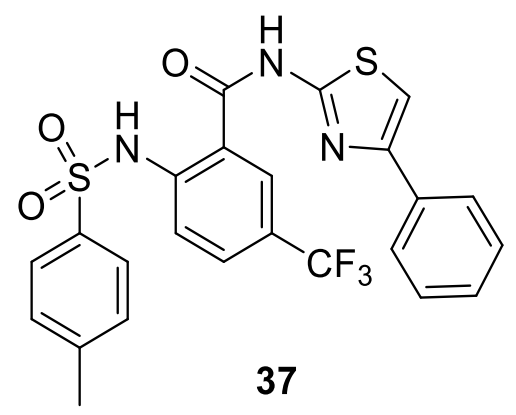 **37**	>50	6 ± 1

Supplementary Table S3. Effects of selected ML364 derivatives on catalysis by isolated SARS-CoV-2 M^{pro} (continues on the following 2 pages). SPE-MS inhibition assays were performed as reported,[20-22] using isolated recombinant SARS-CoV-2 M^{pro} (0.05 µM) and a 37-mer oligopeptide substrate (2.0 µM). Results are means of two independent experiments (n = 2; mean ± SD), each in technical duplicates. The results reveal that some, but not all ML364 derivatives inhibit isolated M^{pro} with moderate potency (>5 µM).

	ML364 derivatives	IC_{50} [µM]		ML364 derivatives	IC_{50} [µM]
i	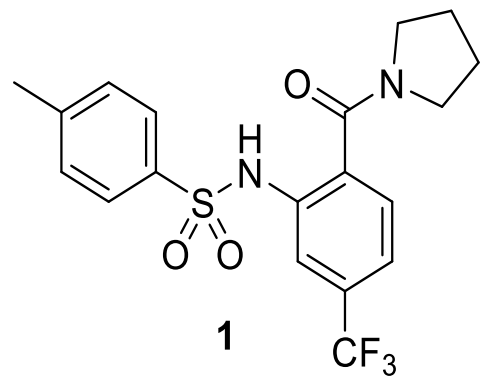 **1**	>50	vii	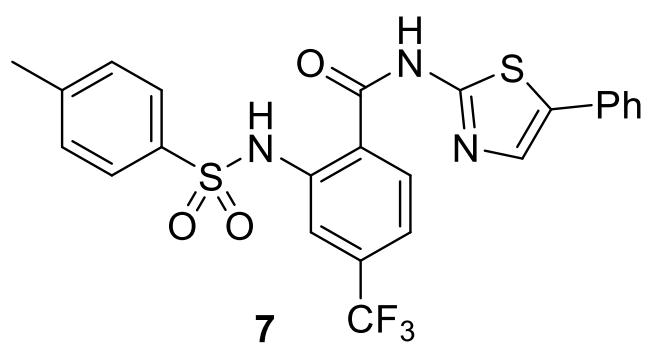 **7**	>50
ii	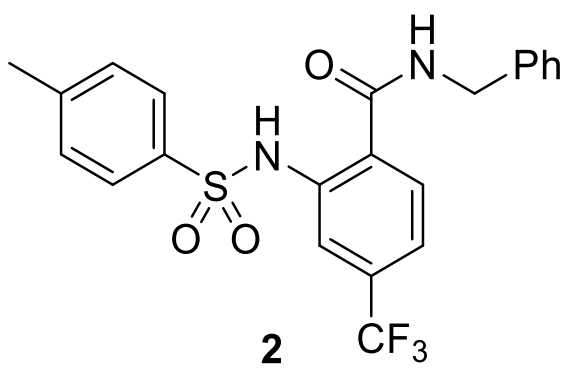 **2**	40 ± 2	viii	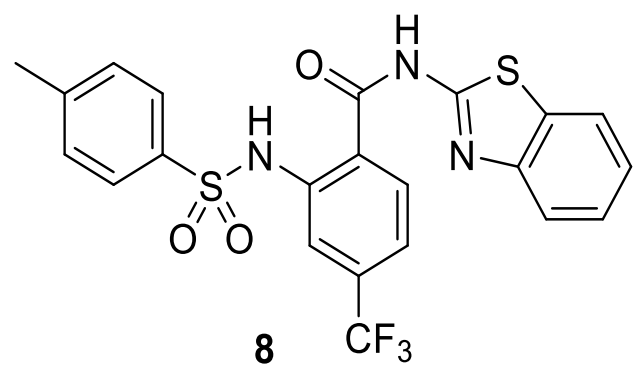 **8**	>50
iii	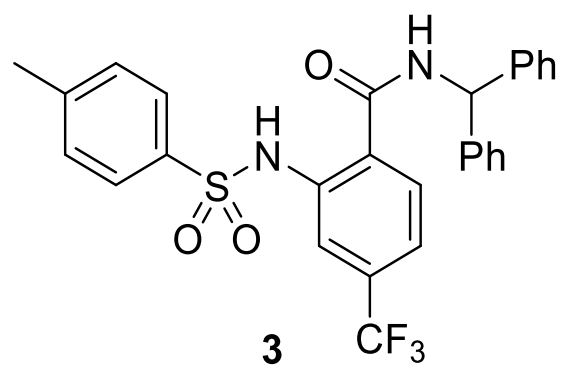 **3**	15 ± 4	ix	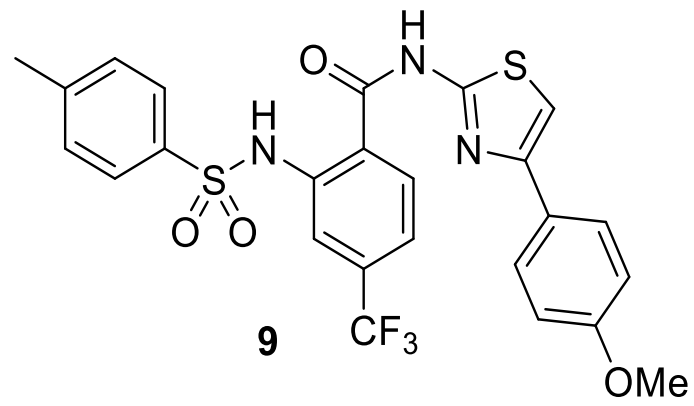 **9**	>50
iv	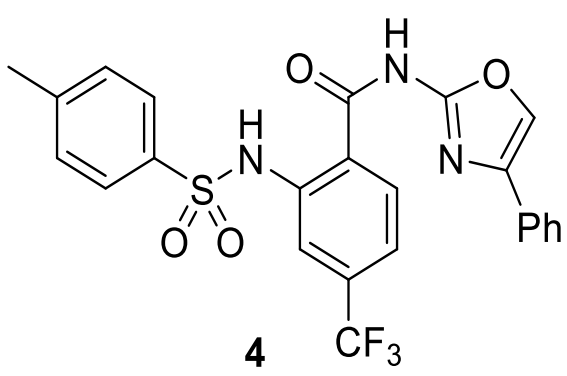 **4**	>50	x	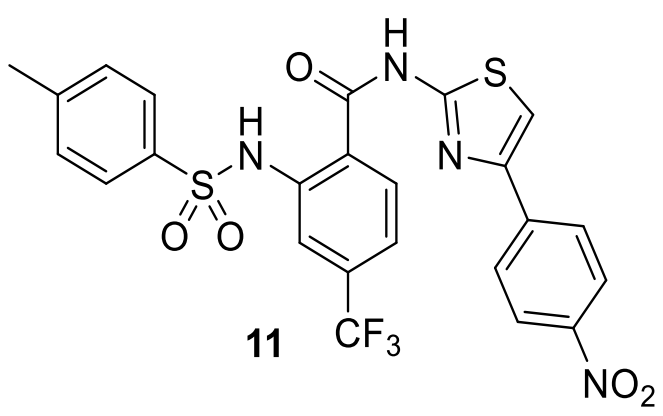 **11**	>50
v	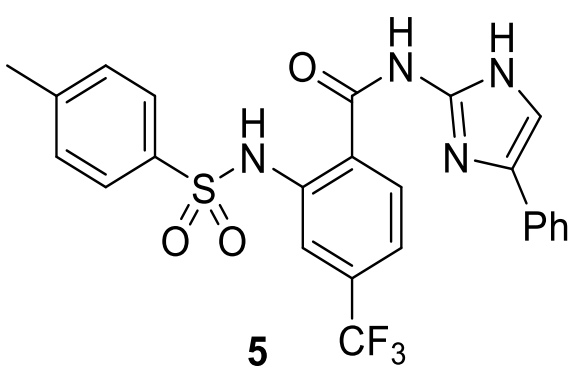 **5**	32 ± 4	xi	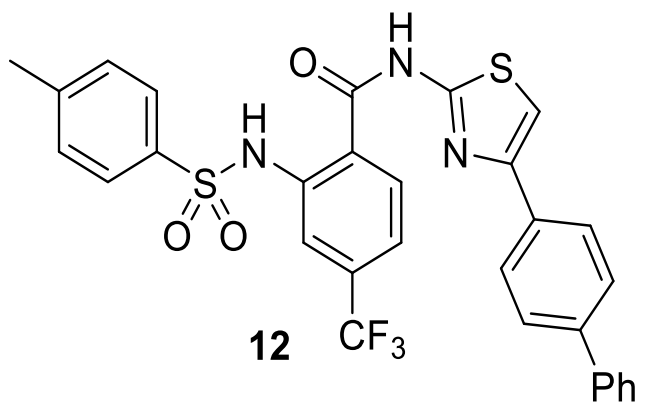 **12**	>50
vi	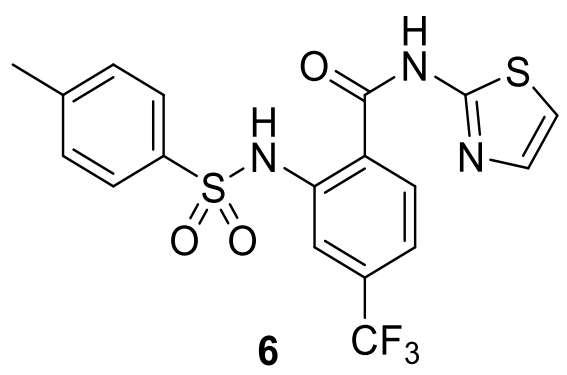 **6**	>50	xii	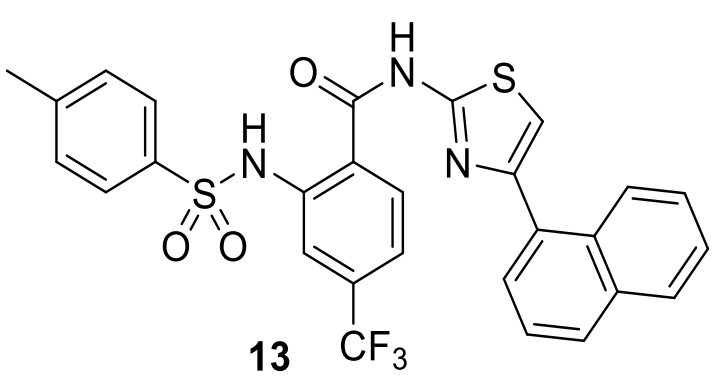 **13**	9.1 ± 2.1

	ML364 derivatives	IC_{50} [µM]		ML364 derivatives	IC_{50} [µM]
xiii	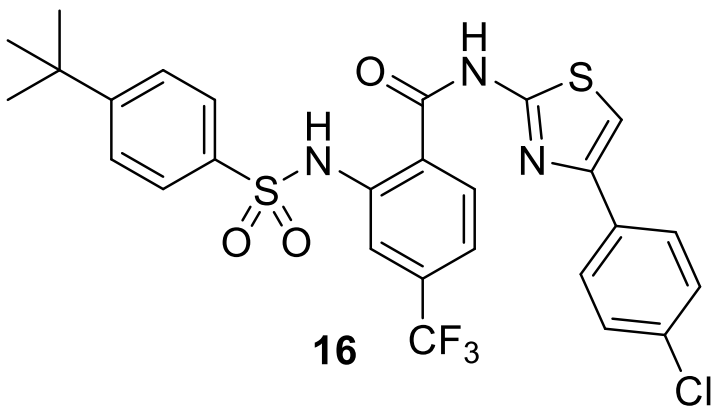 **16**	>50	xix	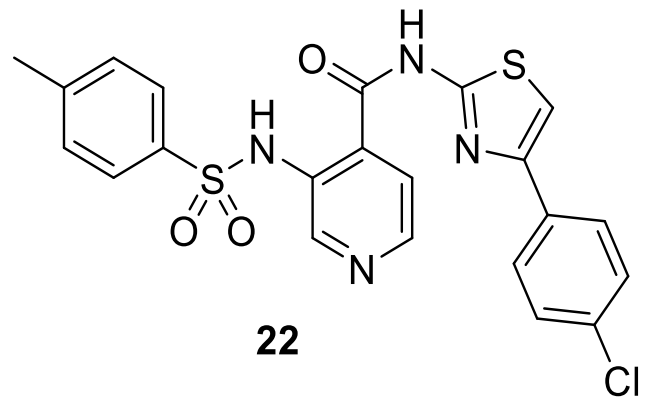 **22**	>50
xiv	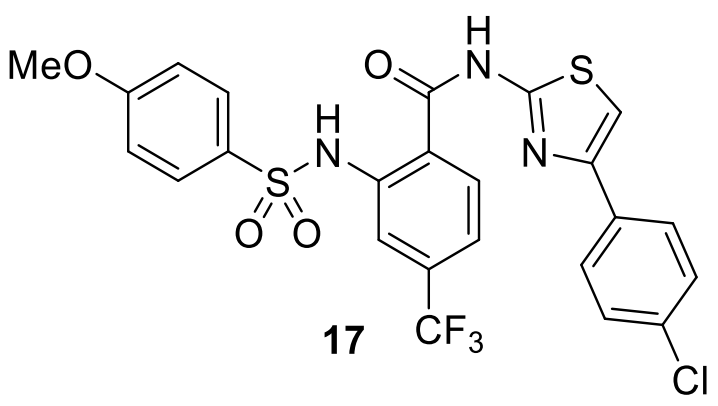 **17**	15 ± 3	xx	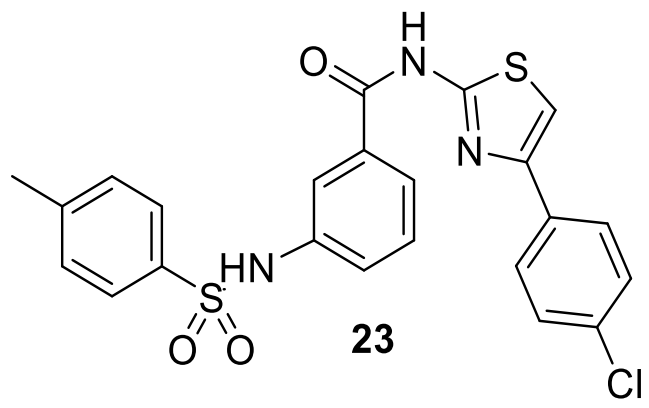 **23**	>50
xv	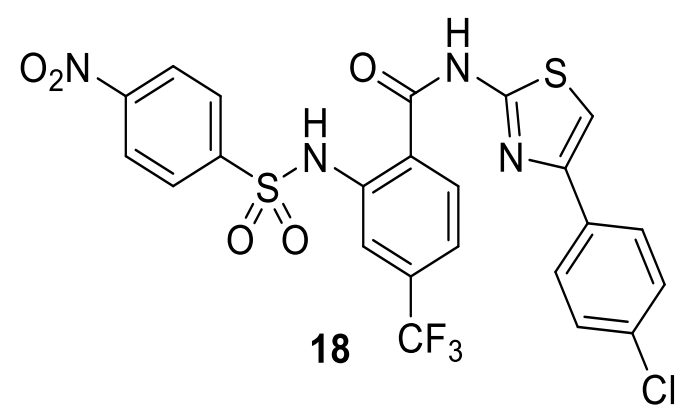 **18**	19 ± 5	xxi	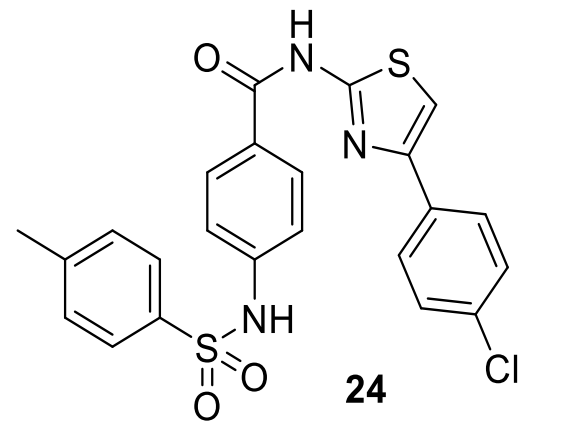 **24**	>50
xvi	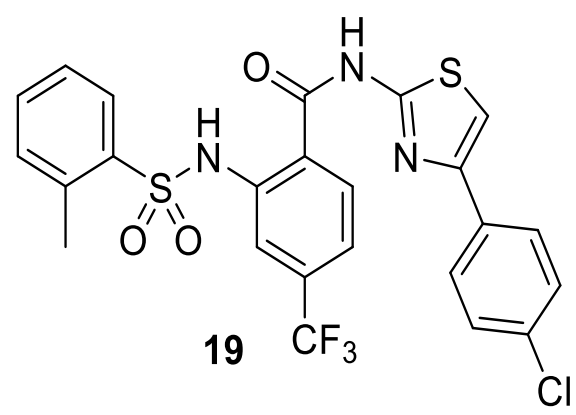 **19**	>50	xxii	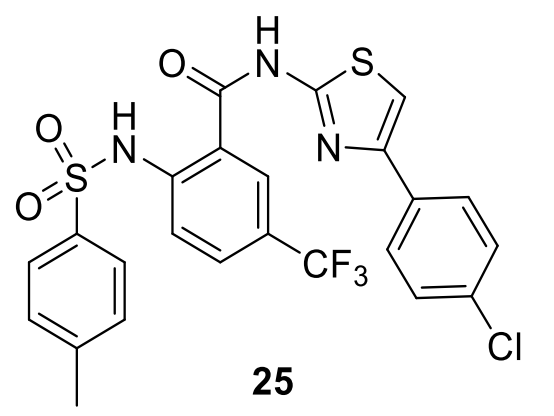 **25**	12 ± 2
xvii	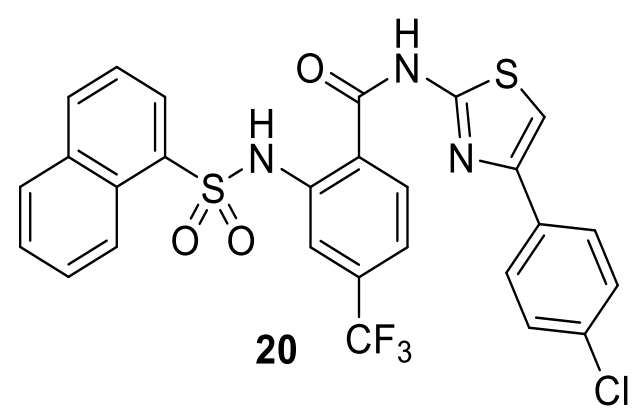 **20**	8.6 ± 0.3	xxiii	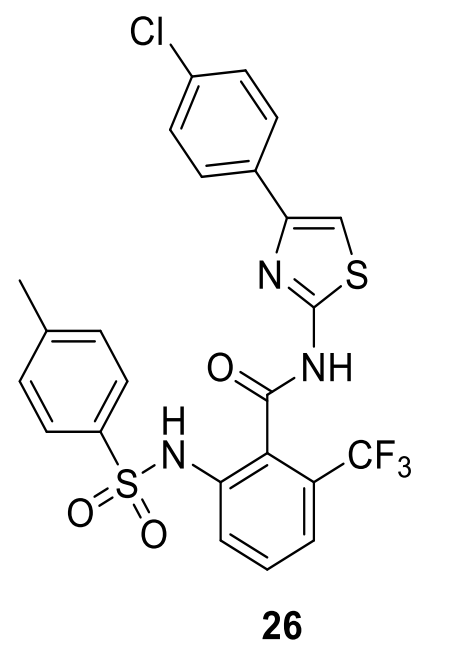 **26**	>50
xviii	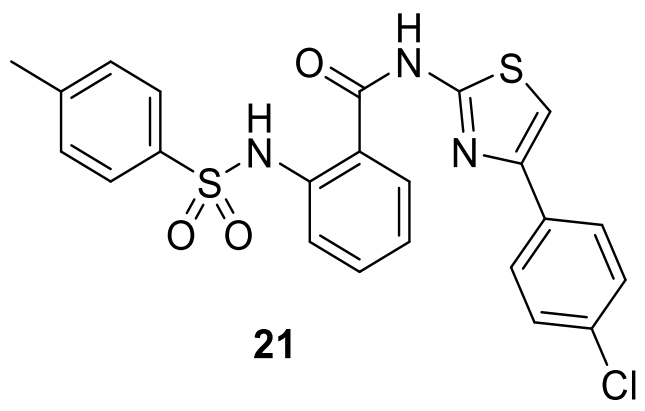 **21**	16 ± 4	xxiv	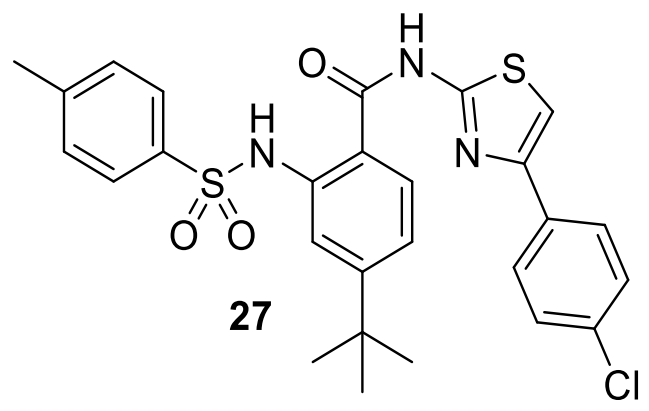 **27**	6.9 ± 1.4

	ML364 derivatives	IC_{50} [μM]		ML364 derivatives	IC_{50} [μM]
xxv	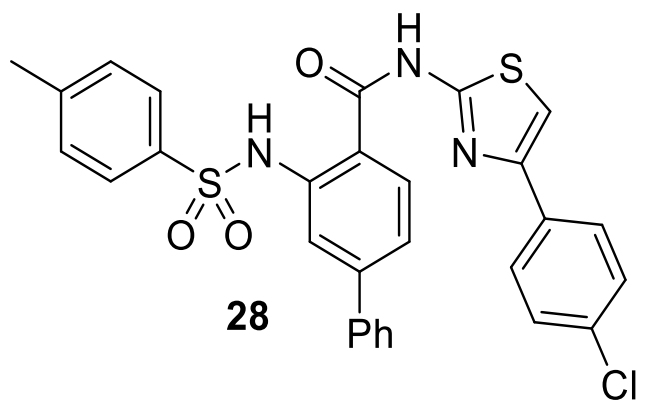 **28**	>50	xxvii	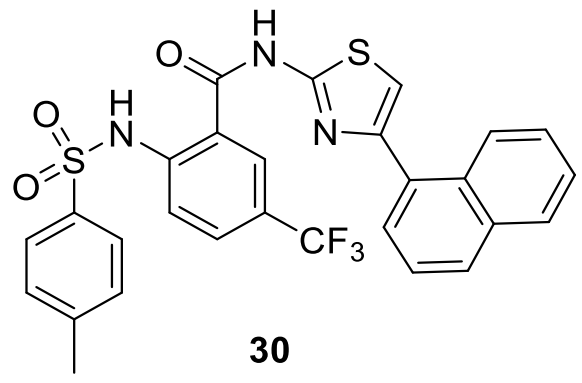 **30**	13 ± 1
xxvi	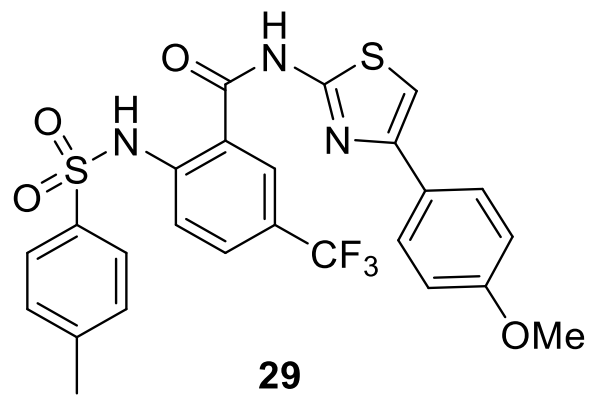 **29**	28 ± 3	xxviii	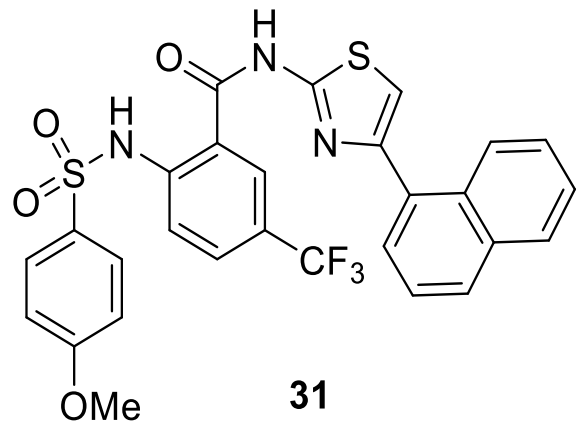 **31**	11 ± 1

Supplementary Table S4. Viability of Vero cells in the presence of selected ML364 derivatives. (see Experimental Section)

	ML364 derivatives	µM		ML364 derivatives	µM
i	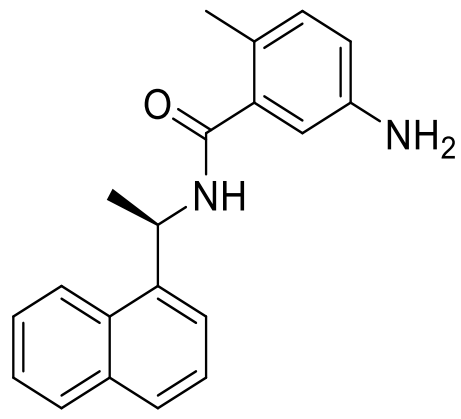 GRL0617	>10	vii	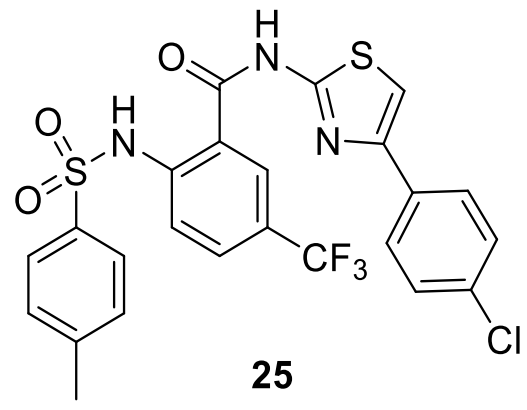 25	9.99
ii	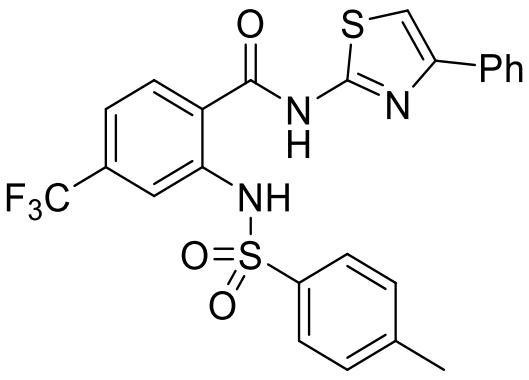 ML364	>10	viii	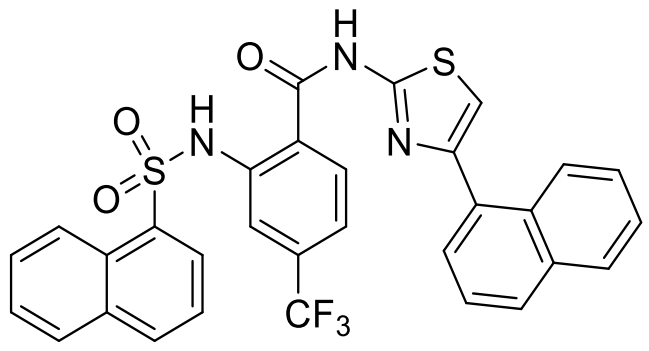 32	>10
iii	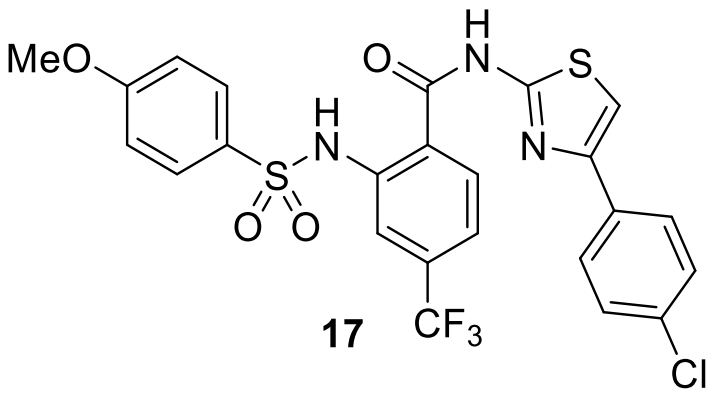 17	>10	ix	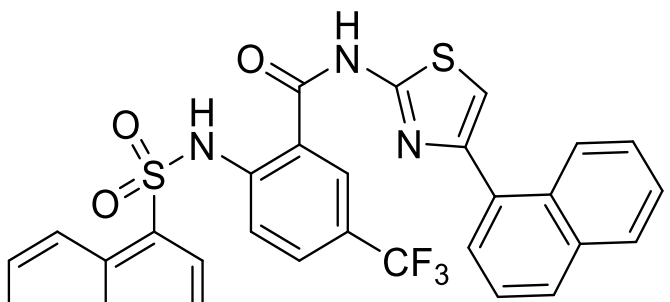 33	>10
iv	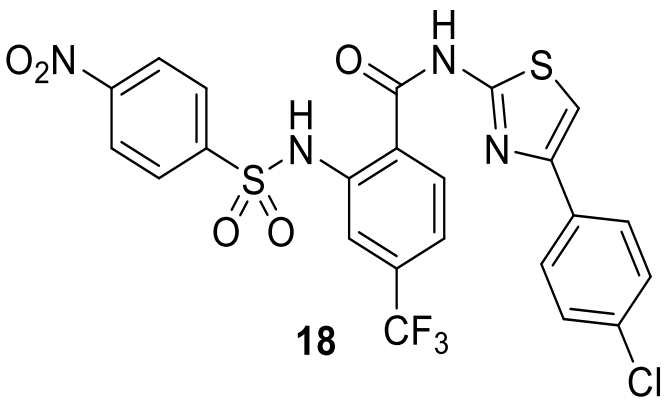 18	>10	x	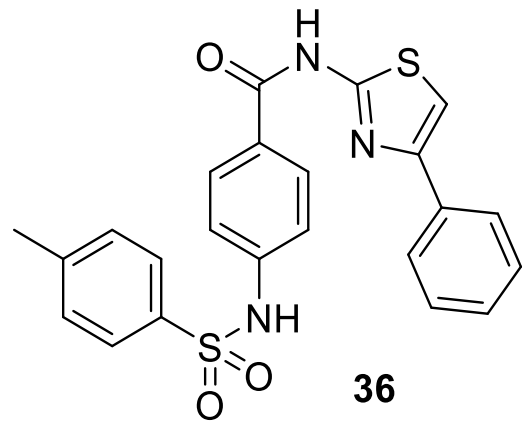 36	>10
v	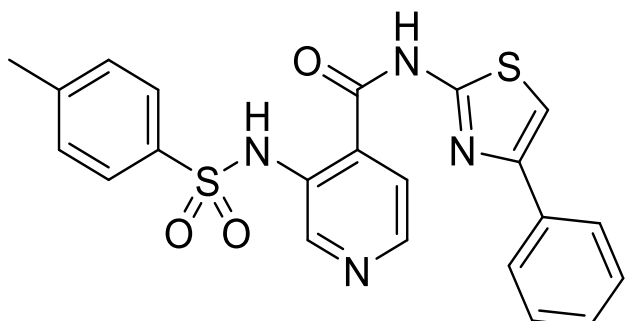 22	>10	xi	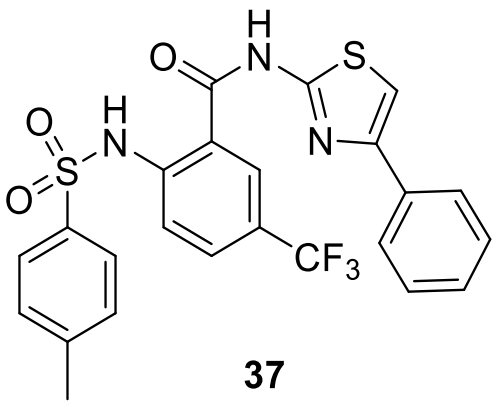 37	>10
vi	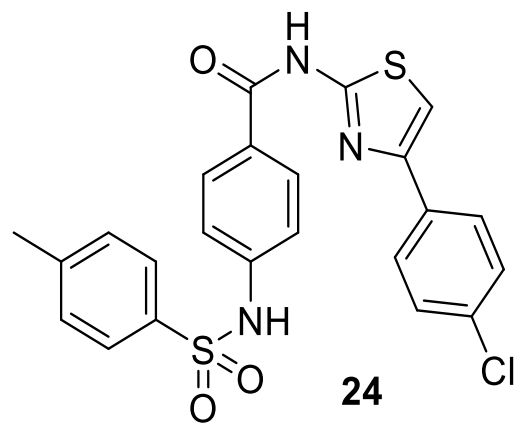 24	>10			

Supplementary Table S5. Molecular mechanics with Poisson–Boltzmann or generalised Born and surface area continuum solvation (MMG/PBSA) binding energy calculations for binding of ML364 derivative 32

to the S and S′ sites.

Binding site	MMGBSA binding energy	MMPBSA binding energy
S site	-28.0 ± 2.8 kcal/mol	-0.4 ± 4.7 kcal/mol
S′ site	-24.1 ± 4.1 kcal/mol	-0.1 ± 3.9 kcal/mol

Supplementary Table S6. Fractions of simulation time that the representative binding mode determined using cluster analysis were observed. This is defined as the fraction of frames for which the root-mean-square deviation of ligand heavy atoms to the representative binding mode was less than 2 Å after aligning the protein backbone.

Binding site	Replica 1	Replica 2	Replica 3
S site	8%	9%	33%
S′ site	56%	64%	51%

Supplementary Table S7. Fractions of simulation time across the concatenated three independent replicas that the indicated hydrogen bonds and π,π-interactions were present.

Interaction	Fraction of simulation time
S site	
H bond with Y268	14%
π-stacking with Y268	61%
S′ site	
H bond with T265	19%
H bond with K274	19%
π-stacking with W106	12%
π-stacking with H272	73%

3. Computational methods

3.1 Docking

Docking calculations were performed using a *holo* PL^{pro} structure (PDB ID: 7JIW,[7] 2.3 Å resolution) for the S sites and an *apo* PL^{pro} structure (PDB ID: 6WX4,[23] 1.66 Å resolution) for the S′ sites. We generated small-molecule input conformations using Schrödinger's LigPrep tool, and prepared the protein using Schrödinger's Protein Preparation Workflow, both with default settings.[24, 25] The pK_as of the sulfonamide and amide N-H groups were calculated using Epik Classic to be 6.2 ± 1.0 and 7.2 ± 2.2, respectively; we thus considered the neutral, the anionic sulfonamide and the anionic amide states of the ligand. Crystallographic waters, buffer molecules, co-crystallised ligands, and all ions except for the zinc-finger domain Zn were removed. H89, H175, and H275 were modelled in the N^{δ}-protonated state; H47, H50, H73, and H255 were modelled in the N^{ε}-protonated state; H17 was modelled in the doubly protonated state. For PDB ID: 6WX4,[23] H255 was modelled in the doubly protonated state, H319 being N^{ε}-protonated, and H320 being N^{δ}-protonated. The four Zn-coordinating residues C189, C192, C224, and C226 were modelled in their anionic state. The active site C111 and H272 residues were considered in their neutral (N; N^{ε}-protonated H272) and zwitterionic (Z) states.

Docking was performed using Glide XP,[26] keeping a maximum of 30 poses per ligand. For PDB ID: 7JIW,[7] the docking grid was set up around the PL^{pro} ligand. For PDB ID: 6WX4,[23] the docking grid was set up around the active site D286. The inner and outer edge lengths were 10 Å and 30 Å, respectively. The output poses were clustered based on their interaction fingerprints with default settings. Poses that were closest to the centroid of each cluster were rescored using molecular mechanics with generalized Born and surface area continuum solvation (MMGBSA) calculations with energy minimisation of protein atoms withing 5 Å of the ligand. The top three poses for both the N and Z states were retained for further analysis.

3.2 Parametrisation

The inhibitor was parameterised using Antechamber.[27, 28] The highest-ranking conformers for each ligand protonation state docked into the Z state were optimised at the HF/6-31G(d) level of theory using the Gaussian16 software package[29] and partial charges were computed using the Restrained Electrostatic Potential (RESP)[30] procedure. The zinc-finger domain was parameterised using the Zinc Amber Force Field (ZAFF).[31] The ff14SB[32] force field was used for the protein and GAFF2 for the ligand.[27] For PDB ID: 7JIW,[7] missing loops were filled using Schrödinger's Protein Preparation Workflow with default settings.

3.3 Molecular dynamics simulations

Docked complexes were prepared for molecular dynamics (MD) simulations using LEaP.[33, 34] The system was placed in a truncated octahedral box with a 12 Å buffer, solvated with TIP3P waters,[35] and neutralised with one sodium ion (for anionic ligands docked into PDB ID: 7JIW[7]). MD simulations were performed using Amber24[33]. The system was subjected to a two-stage energy minimisation, first with positional restraints on protein and ligand atoms at 500 kcal/mol $Å^{-2}$, followed by an unrestrained minimisation. The system temperature was then increased from 0 to 300 K in 50 K increments using Langevin dynamics under constant volume with a time step of 2 fs, with gradually released positional restraints on the protein and ligand (starting at 210 kcal/mol $Å^{-2}$ and decreasing to 10 kcal/mol $Å^{-2}$). The system was equilibrated for 2 ns using a 2 fs time step in the NPT ensemble at 1 bar and 300 K. Temperature and pressure were maintained using a Langevin thermostat (collision frequency = 1.0 ps^{-1}) and a Berendsen barostat (relaxation time = 2.0 ps),[36] respectively.

MD simulations were performed for 100 ns using the NPT ensemble at 1 bar and 300 K under periodic boundary conditions. Covalent bonds involving hydrogen atoms were constrained using the SHAKE algorithm.[37] A 10 Å cut-off was applied for non-bonded interactions, and particle mesh Ewald[32] was used for long-range

electrostatics. Three independent replicas of minimisation, heating, equilibration, and production simulations were performed.
Trajectories were analysed using CPPTRAJ.[33, 38] We determined the stability of a pose by ligand root-mean-square deviation (RMSD) compared to the initial, docked pose after aligning the protein backbone, and validated by visual inspection. To identify representative ligand binding modes, the trajectories from the independent replicas were concatenated and aligned to the protein backbone atoms. Water molecules and ions were removed, and hierarchical agglomerative clustering based on ligand heavy atom RMSD was performed using the average linkage method with an epsilon cut-off of 1.0 Å. The largest cluster was considered to represent the most probable binding mode, and the observed frame closest to the cluster centroid.
The most probable binding modes for both the S and the S′ sites were used as starting points for a second round of MD simulations using the same protocol to confirm their stability. In both cases, the active site was in the Z state. Subsequent analysis used the most probable binding modes as determined from the second round of MD simulations. We quantified the stability of the binding modes to the S and the S′ sites by determining the fraction of frames for which the RMSD of ligand heavy atoms to the representative binding mode determined using cluster analysis was less than 2 Å after aligning the protein backbone. The docking and MD simulations protocol was validated by docking of the reported PL^{pro} inhibitor GRL0617[6] into the used *holo* PL^{pro} structure (PDB ID: 7JIW[7]) and by comparing its binding mode to that crystallographically observed in a reported PL^{pro}:GRL0617 complex structure (PDB ID: 7JRN;[8] Supplementary Figure S7).
H bonds were required to have a donor-acceptor distance of <3.0 Å and an angle between the H bond donor, the H atom, and the H bond acceptor of >135°. π,π-Interactions were required to have a distance between the centres of mass of the π systems of <5.0 Å and an angle between the normals between 0° and 45° for stacking and between 45° and 135° for T-shaped interactions. Interactions were considered relevant if they were present for at least 10% of the simulation time (Supplementary Table S6). We considered the two phenyl rings of naphthalene groups separately. Visualisations were generated using PyMOL.

3.4 MMPB(GB)SA calculations

Per-frame binding energies for the two binding modes were computed using the molecular mechanics with Poisson–Boltzmann or generalized Born and surface area continuum solvation (MMPBSA and MMGBSA) methods as implemented in MMPBSA.py with mbondi3 radii at a 0.15 M salt concentration.[39] Polar solvation terms in MMGBSA were calculated using the GB-Neck2 model;[34] non-polar solvation terms were calculated from the surface area obtained using the Linear Combinations of Pairwise Overlaps model.[40] The first 10 ns of the final trajectories were discarded and only considered frames for which the RMSD of ligand heavy atoms to the representative binding mode, determined using cluster analysis, was less than 2 Å after aligning the protein backbone. Final values are reported as the average ± standard deviation across all analysed frames. We note that this overestimates the uncertainty of the mean estimate.

Simulation input files, scripts, and final poses are available in a GitHub repository (https://github.com/duartegroup/selective-plpro-inhibition).

4. General information

All reagents employed were obtained from commercial sources (Sigma-Aldrich, Inc.; Fluorochem Ltd.; Alfa Aesar; Aurum Pharmatech LLC) and were used as received. Anhydrous solvents were from Sigma-Aldrich, Inc. and stored under a nitrogen atmosphere. Transfers of solvents, liquids, and solutions were carried out using stainless-steel needles and syringes flushed with nitrogen gas. All air-sensitive reactions were performed under a nitrogen atmosphere using oven-dried glassware and dry solvents. Reactions were monitored by thin-layer chromatography (TLC) on Merck silica gel 60 F_{254} plates and visualised using UV irradiation (λ_{max} = 254 or 365 nm). Flash column chromatography was performed using Biotage® Selekt One or Biotage® Isolera flash purification systems with pre-packed Biotage® Sfär Silica D Duo cartridge or Biotage® Sfär C18 D Duo Ultra flash cartridges. HPLC-grade solvents were used for reaction work-ups, extractions, and purifications.

Melting points (m.p.) were determined using a Stuart SMP-40 automated melting point apparatus. ^{1}H, ^{13}C, ^{19}F, COSY, HSQC, and HMBC NMR spectra were recorded using a Bruker AVIII HD 400 machine equipped with a 5 mm z-gradient BBFO probe, a Bruker NEO 400 machine equipped with a 5 mm z-gradient broadband multinuclear SMART probe, a Bruker AVIII HD 500 machine equipped with a 5mm z-gradient BBFO SMART probe, a Bruker NEO 600 machine equipped with a 5 mm BB-F/^{1}H helium-cooled cryoprobe or a Bruker AVIII HD 600 machine equipped with a BB-F/^{1}H Prodigy N_2 cryoprobe. Deuterated solvents containing tetramethylsilane (TMS) as an internal standard were from Sigma-Aldrich and used for NMR measurements; signals at 0 ppm in the ^{1}H and ^{13}C spectra are due to TMS. Chemical shifts (δ) are reported in parts per million (ppm) downfield from TMS or residual solvent peaks. Multiplicities are s (singlet), d (doublet), t (triplet), q (quartet), m (multiplet), br (broad), app (apparent), or combinations. Coupling constants (J) are reported in Hertz (Hz).

Infrared (IR) spectra were recorded using a Bruker Tensor 27 FT-IR spectrometer; wavenumbers (ν_{max}) are in cm^{-1}. High-resolution mass spectrometry (HRMS) analyses were performed using a Thermo Scientific Exactive mass spectrometer (ThermoFisher Scientific) with electrospray ionization (ESI) in positive or negative mode. Results are reported as mass-to-charge ratios (m/z).

5. General synthetic procedures

5.1 General Procedure A

The procedure is based on one reported for the synthesis of ML364:[41] To a mixture of a carboxylic acid (1.0 equiv.) and Na_2CO_3 (2.5 equiv.) in H_2O (0.5 M) was added a sulfonyl chloride (1.0 equiv.). The reaction mixture was stirred for 4 h at 80 °C, before being cooled to room temperature. The reaction mixture was acidified with 1 N aqueous HCl to pH 2-3; the suspension was filtered under vacuum and the crude solid was purified using flash column chromatography.

5.2 General Procedure B

The procedure is based on one reported for the synthesis of ML364:[41] To a solution of a carboxylic acid (1.0 equiv.) in DMF (0.4 M) were added DIPEA (4.0 equiv.) and (dimethylamino)-*N*,*N*-dimethyl(3*H*-triazolo[4,5-*b*]pyridin-3-yloxy)methaniminium hexafluorophosphate[42] (HATU) (1.2 equiv.). The mixture was stirred for 15 min, before an amine (1.0 equiv.) was added. The reaction mixture was stirred for 2 h at 80 °C, then allowed to cool to ambient temperature, extracted with ethyl acetate, then washed with saturated aqueous NH_4Cl (2 times), saturated aqueous $NaHCO_3$ (2 times), and brine (2 times), dried over $MgSO_4$, then concentrated *in vacuo*. The crude residue was purified using flash column chromatography.

6. Experimental procedures and compound characterisations

4-Methyl-*N*-(2-(pyrrolidine-1-carbonyl)-5-(trifluoromethyl)phenyl)benzenesulfonamide (1)

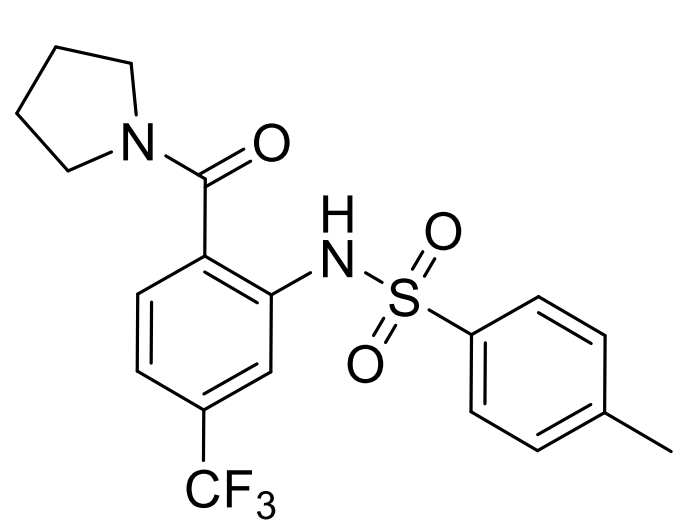

According to General Procedure B, amide **1** (103 mg, 0.25 mmol, 25%) was obtained from the reported sulfonamide **1a**[41] (359 mg, 1.0 mmol) and commercially-sourced pyrrolidine (82.1 µL, 1.0 mmol), followed by flash column chromatography (10 g Sfär Silica Duo cartridge; 40 mL/min; 100%$_{v/v}$ cyclohexane (2 CV), followed by a linear gradient (25 CV): 0%$_{v/v}$→50%$_{v/v}$ ethyl acetate in cyclohexane) and subsequent purification using reverse phase column chromatography (12 g Sfär C18 Duo cartridge; 12 mL/min; 100%$_{v/v}$ (H_2O + 0.1%$_{v/v}$ Formic acid, FA) (2 CV), followed by a gradient (8 CV): 0%$_{v/v}$→40%$_{v/v}$ and (20 CV): 40%$_{v/v}$→100%$_{v/v}$ (acetonitrile + 0.1%$_{v/v}$ FA) in (H_2O + 0.1%$_{v/v}$ FA)).

Yellow oil; ^{1}H NMR (400 MHz, 300 K, acetone-*d$_6$*): δ = 9.50 (s, 1H), 7.95 (d, *J* = 1.8 Hz, 1H), 7.69 (dd, *J* = 8.1, 1.0 Hz, 1H), 7.66 – 7.58 (m, 2H), 7.52 (ddd, *J* = 8.1, 1.8, 0.8 Hz, 1H), 7.41 – 7.33 (m, 2H), 3.44 (t, *J* = 7.0 Hz, 2H), 3.05 (t, *J* = 6.7 Hz, 2H), 2.38 (s, 3H), 1.88 (p, *J* = 6.8 Hz, 2H), 1.72 ppm (p, *J* = 6.5 Hz, 2H); ^{13}C NMR (100 MHz, 300 K, acetone-*d$_6$*): δ = 166.7, 145.2, 138.0, 137.3, 132.7 (q, *J* = 32.7 Hz), 131.5, 130.7 (2C), 130.1, 127.9 (2C), 124.4 (q, *J* = 272.3 Hz), 121.8 (q, *J* = 3.8 Hz), 120.8 (q, *J* = 3.8 Hz), 50.0, 47.1, 26.8, 24.6, 21.4 ppm; ^{19}F NMR (375 MHz, 300 K, acetone-*d$_6$*): δ = -63.7 ppm (s, 3F); IR (film): ṽ = 3019, 2927, 1702, 1217 cm^{-1}; HRMS (ESI): m/z calculated for $C_{19}H_{20}F_3N_2O_3S$ $[M+H]^+$: 413.1141, found: 413.1152.

***N*-Benzyl-2-((4-methylphenyl)sulfonamido)-4-(trifluoromethyl)benzamide (2)**

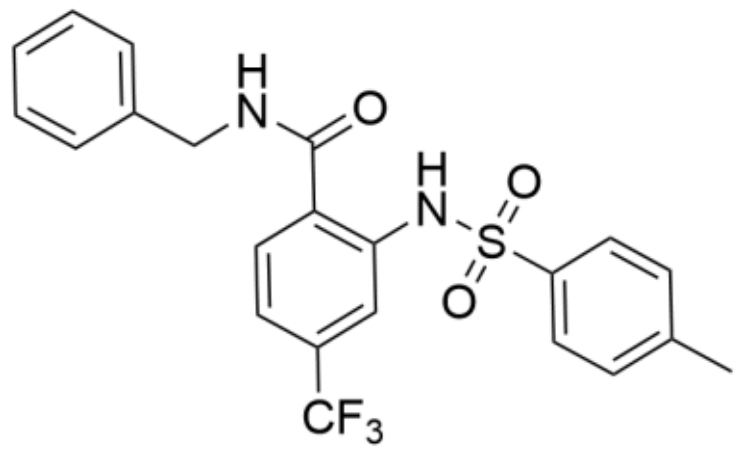

According to General Procedure B, amide **2** (146 mg, 0.32 mmol, 65%) was obtained from the reported sulfonamide **1a**[41] (180 mg, 0.50 mmol) and commercially-sourced benzylamine (54.6 µL, 0.50 mmol), followed by reverse phase column chromatography (12 g Sfär C18 Duo cartridge; 12 mL/min; 100%$_{v/v}$ (H_2O + 0.1%$_{v/v}$ FA) (2 CV), followed by a gradient (8 CV): 0%$_{v/v}$→40%$_{v/v}$ and (20 CV): 40%$_{v/v}$→100%$_{v/v}$ (acetonitrile + 0.1%$_{v/v}$ FA) in (H_2O + 0.1%$_{v/v}$ FA)).

White solid; m.p.: 186–188 °C; ^{1}H NMR (600 MHz, 300 K, CD_3CN): δ = 11.11 (s, 1H), 7.89 (dd, *J* = 2.0, 0.5 Hz, 1H), 7.73 – 7.67 (m, 2H), 7.60 – 7.55 (m, 2H), 7.43 (dd, *J* = 8.5, 2.0, 1H), 7.40 – 7.36 (m, 2H), 7.35 – 7.29 (m, 3H), 7.25 – 7.20 (m, 2H), 4.46 (d, *J* = 6.0 Hz, 2H), 2.33 ppm (s, 3H); ^{13}C NMR (150 MHz, 300 K, CD_3CN): δ = 168.2, 145.7, 140.3, 139.1, 136.6, 134.1 (q, *J* = 33.0 Hz), 130.8 (2C), 130.0, 129.5 (2C), 128.7 (2C), 128.3, 128.0 (2C), 125.9, 124.4 (q, *J* = 272.0 Hz), 121.3 (q, *J* = 4.0 Hz), 118.5 (q, *J* = 7.0 Hz), 44.2, 21.5 ppm; ^{19}F NMR (565 MHz, 300 K, CD_3CN): δ = -64.9 ppm (s, 3F); IR (film): ṽ = 3659, 2980, 1645, 1542, 1401, 1331, 1251, 1157, 1080 cm^{-1}; HRMS (ESI): m/z calculated for $C_{22}H_{18}F_3N_2O_3S$ $[M-H]^-$: 447.0996, found: 447.1018.

***N*-Benzhydryl-2-((4-methylphenyl)sulfonamido)-4-(trifluoromethyl)benzamide (3)**

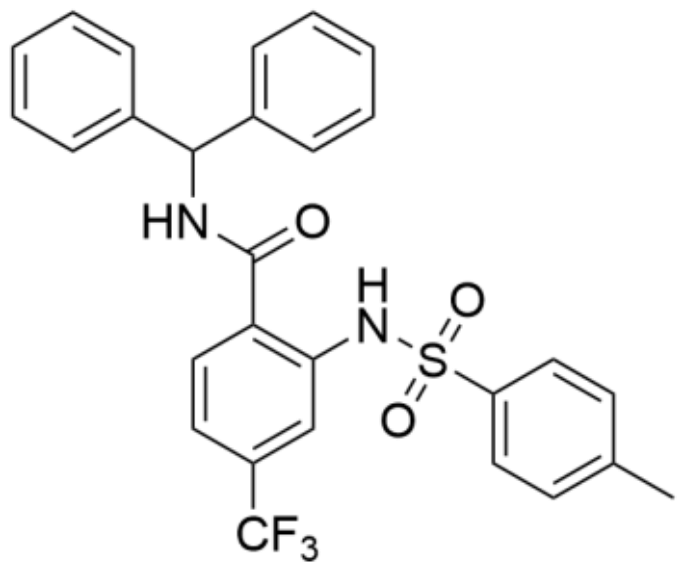

According to General Procedure B, amide **3** (138 mg, 0.26 mmol, 53%) was obtained from the reported sulfonamide **1a**[41] (180 mg, 0.50 mmol) and commercially-sourced diphenylmethanamine (91.6 mg, 0.50 mmol), followed by reverse phase column chromatography (12 g Sfär C18 Duo cartridge; 12 mL/min; 100%$_{v/v}$ (H_2O + 0.1%$_{v/v}$ FA) (2 CV), followed by a gradient (8 CV): 0%$_{v/v}$→40%$_{v/v}$ and (20 CV): 40%$_{v/v}$→100%$_{v/v}$ (acetonitrile + 0.1%$_{v/v}$ FA) in (H_2O + 0.1%$_{v/v}$ FA)).

White solid; m.p.: 166–168 °C; ^{1}H NMR (600 MHz, 300 K, CD_3CN): δ = 10.89 (s, 1H), 7.93 (s, 1H), 7.88 (d, *J* = 1.5 Hz, 1H), 7.84 (d, *J* = 8.0 Hz, 1H), 7.59 – 7.54 (m, 2H), 7.44 – 7.41 (d, *J* = 8.0 Hz, 1H), 7.40 – 7.30 (m, 10H), 7.20 – 7.15 (m, 2H), 6.31 (d, *J* = 7.5 Hz, 1H), 2.28 ppm (s, 3H); ^{13}C NMR (150 MHz, 300 K, CD_3CN): δ = 167.8, 145.7, 142.1 (2C), 140.2, 136.5, 134.2 (q, *J* = 33.0 Hz), 130.8 (3C), 129.6 (4C), 128.6 (2C), 128.5 (4C), 127.9 (2C), 125.6, 124.4 (q, *J* = 272.2 Hz), 121.0 (q, *J* = 4.0 Hz), 118.1 (q, *J* = 4.5 Hz), 58.8, 21.5 ppm; ^{19}F NMR (565 MHz, 300 K, CD_3CN): δ = -64.9 ppm (s, 3F); IR (film): ṽ = 3651, 3331, 2981, 1636, 1553, 1430, 1334, 1254, 1157 cm^{-1}; HRMS (ESI): m/z calculated for $C_{28}H_{22}F_3N_2O_3S$ [M-H]$^-$: 523.1309, found: 523.1299.

2-((4-Methylphenyl)sulfonamido)-*N*-(4-phenyloxazol-2-yl)-4-(trifluoromethyl)benzamide (4)

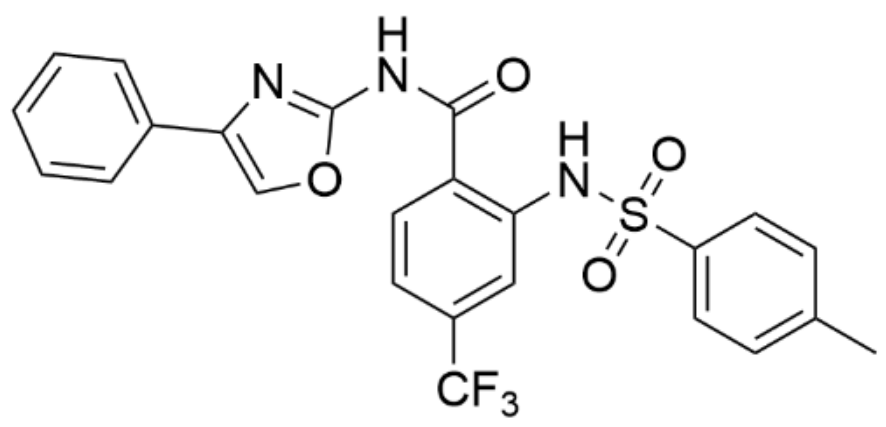

According to General Procedure B, amide **4** (103 mg, 0.21 mmol, 38%) was obtained from the reported sulfonamide **1a**[41] (200 mg, 0.56 mmol) and commercially-sourced 4-phenyloxazol-2-amine (89.7 mg, 0.3 mmol), followed by reverse phase column chromatography (12 g Sfär C18 Duo cartridge; 12 mL/min; 100%$_{v/v}$ (H_2O + 0.1%$_{v/v}$ FA) (2 CV), followed by a gradient (8 CV): 0%$_{v/v}$→40%$_{v/v}$ and (20 CV): 40%$_{v/v}$→100%$_{v/v}$ (acetonitrile + 0.1%$_{v/v}$ FA) in (H_2O + 0.1%$_{v/v}$ FA)) and subsequent purification using flash column chromatography (10 g Sfär Silica Duo cartridge; 40 mL/min; 100%$_{v/v}$ cyclohexane (2 CV), followed by a linear gradient (25 CV): 0%$_{v/v}$→50%$_{v/v}$ ethyl acetate in cyclohexane).

White solid; m.p.: 172–174 °C; ^{1}H NMR (600 MHz, 300 K, DMSO-*d$_6$*): δ = 11.97 (s, 1H), 10.39 (s, 1H), 8.46 (s, 1H), 7.91 (s, 1H), 7.77 (d, *J* = 7.7 Hz, 2H), 7.66 (d, *J* = 8.0 Hz, 2H), 7.60 (br s, 1H), 7.52 (br s, 1H), 7.45 (t, *J* = 7.6 Hz, 2H), 7.39 – 7.30 (m, 3H), 2.32 ppm (s, 3H); ^{13}C NMR (150 MHz, 300 K, DMSO-*d$_6$*): δ = 164.3, 153.0, 143.9, 139.2, 136.8, 135.7, 131.9 (q, *J* = 32.4 Hz), 131.5, 130.8 (2C), 129.7 (2C), 128.7 (2C), 128.0, 126.9 (2C), 125.0 (2C), 123.2 (q, *J* = 272.7 Hz), 120.9 (2C), 118.3, 20.9 ppm; ^{19}F NMR (565 MHz, 300 K, DMSO-*d$_6$*): δ = -62.6 ppm (s, 3F); IR (film): ṽ = 3653, 2980, 2925, 1739, 1611, 1576, 1495, 1455, 1393, 1324, 1290, 1258, 1166, 1090 cm^{-1}; HRMS (ESI): m/z calculated for $C_{24}H_{19}F_3N_3O_4S$ [M+H]$^+$: 502.1043, found: 502.1044.

2-((4-Methylphenyl)sulfonamido)-*N*-(4-phenyl-1*H*-imidazol-2-yl)-4-(trifluoromethyl)benzamide (5)

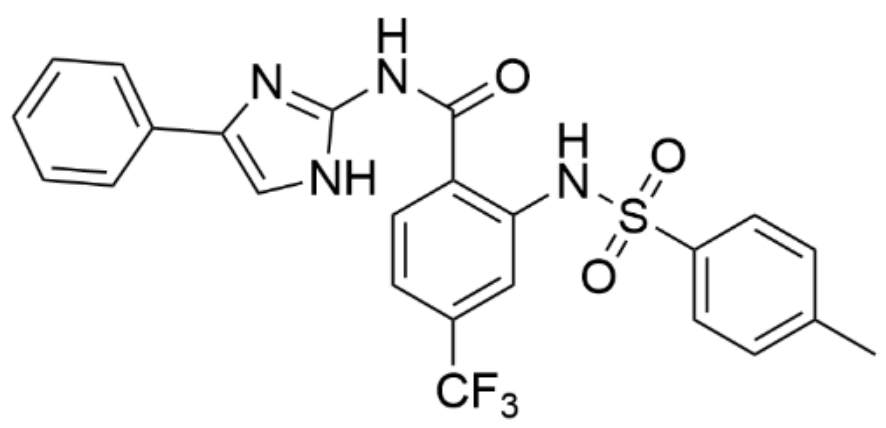

According to General Procedure B, amide **5** (92.0 mg, 0.18 mmol, 25%) was obtained from the reported sulfonamide **1a**[41] (270 mg, 0.75 mmol) and commercially-sourced 4-phenyl-1*H*-imidazol-2-amine (119 mg, 0.75 mmol), followed by reverse phase column chromatography (12 g Sfär C18 Duo cartridge; 12 mL/min; 100%$_{v/v}$ (H_2O + 0.1%$_{v/v}$ FA) (2 CV), followed by a gradient (8 CV): 0%$_{v/v}$→40%$_{v/v}$ and (20 CV): 40%$_{v/v}$→100%$_{v/v}$ (acetonitrile + 0.1%$_{v/v}$ FA) in (H_2O + 0.1%$_{v/v}$ FA)).

White solid; m.p.: 176–178 °C; ^{1}H NMR (400 MHz, 300 K, acetone-d_6): δ = 8.36 (dd, *J* = 8.2, 1.1 Hz, 1H), 7.98 – 7.93 (m, 1H), 7.84 – 7.76 (m, 2H), 7.74 – 7.70 (m, 2H), 7.53 – 7.43 (m, 3H), 7.40 – 7.35 (m, 1H), 7.33 – 7.29 (m, 1H), 7.30 – 7.24 (m, 2H), 2.30 ppm (s, 3H); ^{13}C NMR (100 MHz, 300 K, acetone-d_6): δ = 172.1, 150.2, 144.6, 141.6, 138.1, 133.3 (q, *J* = 33.0 Hz), 132.9, 130.5 (2C), 129.9 (2C), 129.8, 128.9, 128.7, 128.1, 127.9 (2C), 125.5 (2C), 124.8 (q, *J* = 271.0 Hz), 119.2, 116.6, 110.0, 21.3 ppm; ^{19}F NMR (375 MHz, 300 K, acetone-d_6): δ = -63.7 ppm (s, 3F); IR (film): ṽ = 3655, 2981, 2956, 1735, 1638, 1601, 1587, 1443, 1392, 1351, 1169, 1123, 1074 cm^{-1}; HRMS (ESI): m/z calculated for $C_{24}H_{20}F_3N_4O_3S$ $[M+H]^+$: 501.1203, found: 501.1194.

2-((4-Methylphenyl)sulfonamido)-*N*-(thiazol-2-yl)-4-(trifluoromethyl)benzamide (6)

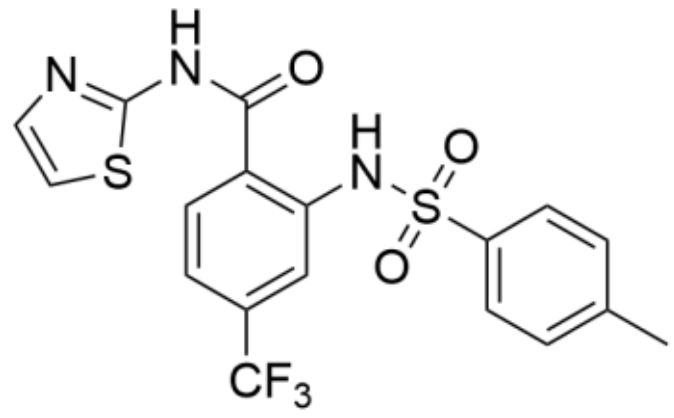

According to General Procedure B, amide **6** (40.7 mg, 0.092 mmol, 31%) was obtained from the reported sulfonamide **1a**[41] (108 mg, 0.30 mmol) and commercially-sourced thiazol-2-amine (30.2 mg, 0.30 mmol), followed by reverse phase column chromatography (12 g Sfär C18 Duo cartridge; 12 mL/min; 100%$_{v/v}$ (H_2O + 0.1%$_{v/v}$ FA) (2 CV), followed by a gradient (8 CV): 0%$_{v/v}$→40%$_{v/v}$ and (20 CV): 40%$_{v/v}$→100%$_{v/v}$ (acetonitrile + 0.1%$_{v/v}$ FA) in (H_2O + 0.1%$_{v/v}$ FA)) and subsequent purification using flash column chromatography (10 g Sfär Silica Duo cartridge; 40 mL/min; 100%$_{v/v}$ cyclohexane (2 CV), followed by a linear gradient (25 CV): 0%$_{v/v}$→50%$_{v/v}$ ethyl acetate in cyclohexane).

White solid; m.p.: 190–192 °C; ^{1}H NMR (400 MHz, 300 K, $CDCl_3$): δ = 8.02 (d, *J* = 1.7 Hz, 1H), 7.82 (d, *J* = 8.1 Hz, 1H), 7.72 – 7.64 (m, 2H), 7.37 – 7.30 (m, 1H), 7.09 – 7.00 (m, 3H), 6.75 (d, *J* = 3.7 Hz, 1H), 2.19 ppm (s, 3H); ^{13}C NMR (100 MHz, 300 K, $CDCl_3$): δ = 166.0, 160.3, 144.6, 139.8, 135.8 (2C), 135.4 (q, *J* = 33.4 Hz), 129.9 (2C), 129.3, 127.4 (2C), 124.0, 123.1 (q, *J* = 271.0 Hz), 120.4 (q, *J* = 4.0 Hz), 119.1 (q, *J* = 4.2 Hz), 114.6, 21.6 ppm; ^{19}F NMR (375 MHz, 300 K, $CDCl_3$): δ = -63.6 ppm (s, 3F); IR (film): ṽ = 3281, 2926, 1661, 1554, 1395, 1313, 1102, 1073 cm^{-1}; HRMS (ESI): m/z calculated for $C_{18}H_{13}F_3N_3O_3S_2$ $[M-H]^-$: 440.0356, found: 440.0368.

2-((4-Methylphenyl)sulfonamido)-*N*-(5-phenylthiazol-2-yl)-4(trifluoromethyl)benzamide (7)

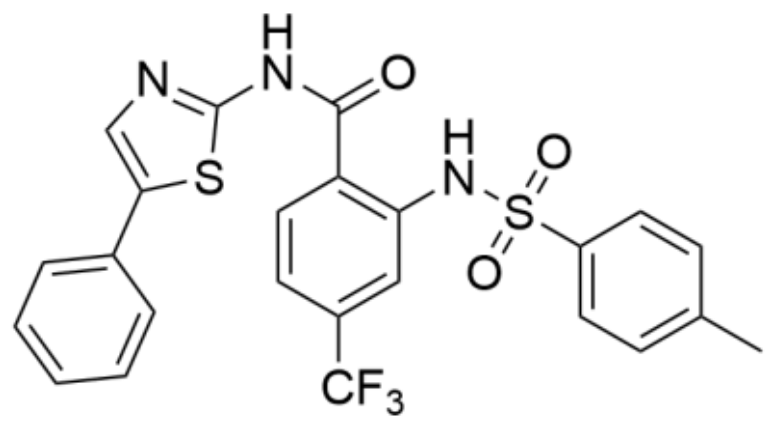

According to General Procedure B, amide **7** (67.2 mg, 0.13 mmol, 26%) was obtained from the reported sulfonamide **1a**[41] (180 mg, 0.50 mmol) and commercially-sourced 5-phenylthiazol-2-amine (88.1 mg, 0.50 mmol), followed by reverse phase column chromatography (12 g Sfär C18 Duo cartridge; 12 mL/min; 100%$_{v/v}$ (H_2O + 0.1%$_{v/v}$ FA) (2 CV), followed by a gradient (8 CV): 0%$_{v/v}$→40%$_{v/v}$ and (20 CV): 40%$_{v/v}$→100%$_{v/v}$ (acetonitrile + 0.1%$_{v/v}$ FA) in (H_2O + 0.1%$_{v/v}$ FA)).

Yellow solid; m.p.: > 200 °C (decomposition); ^{1}H NMR (500 MHz, 300 K, acetone-d_6): δ = 8.33 (d, *J* = 8.2 Hz, 1H), 7.97 (d, *J* = 1.8 Hz, 1H), 7.93 (s, 1H), 7.75 – 7.67 (m, 4H), 7.53 – 7.45 (m, 3H), 7.43 – 7.36 (m, 1H), 7.30 – 7.24 (m, 2H), 2.30 ppm (s, 3H); ^{13}C NMR (125 MHz, 300 K, acetone-d_6): δ = 169.7, 162.1, 145.2, 140.9, 137.3, 134.2 (q, *J* = 32.5 Hz), 132.4, 132.2, 131.6, 130.7 (2C), 130.1 (2C), 129.1, 128.0 (2C), 126.7 (3C), 126.5, 124.5 (q, *J* = 272.1 Hz), 120.4, 117.9, 21.4 ppm; note that the ^{13}C NMR signal at 162.1 ppm was assigned using HMBC. ^{19}F NMR (470 MHz, 300 K, acetone-d_6): δ = -63.9 ppm (s, 3F); IR (film): ṽ = 3660, 2980, 2884, 1712, 1566, 1419, 1384, 1323, 1240, 1132, 1102 cm^{-1}; HRMS (ESI): m/z calculated for $C_{24}H_{19}F_3N_3O_3S_2$ $[M+H]^+$: 518.0815, found: 518.0801.

***N*-(Benzo[*d*]thiazol-2-yl)-2-((4-methylphenyl)sulfonamido)-4 (trifluoromethyl)benzamide (8)**

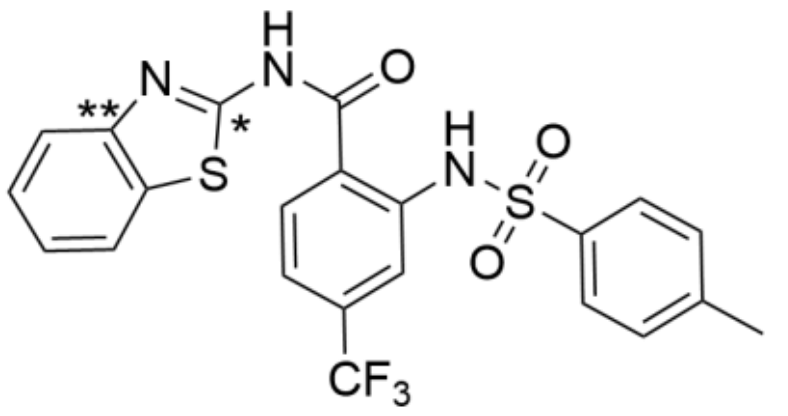

According to General Procedure B, amide **8** (32.8 mg, 0.067 mmol, 22%) was obtained from the reported sulfonamide **1a**[41] (108 mg, 0.30 mmol) and commercially-sourced benzo[*d*]thiazol-2-amine (45.1 mg, 0.30 mmol), followed by reverse phase column chromatography (12 g Sfär C18 Duo cartridge; 12 mL/min; 100%$_{v/v}$ (H_2O + 0.1%$_{v/v}$ FA) (2 CV), followed by a gradient (8 CV): 0%$_{v/v}$→40%$_{v/v}$ and (20 CV): 40%$_{v/v}$→100%$_{v/v}$ (acetonitrile + 0.1%$_{v/v}$ FA) in (H_2O + 0.1%$_{v/v}$ FA)).

Yellow solid; m.p.: > 230 °C (decomposition); ^{1}H NMR (600 MHz, 300 K, acetone-*d$_6$*): δ = 8.38 (d, *J* = 8.2 Hz, 1H), 8.01 – 7.96 (m, 2H), 7.72 (dd, *J* = 8.6, 7.1 Hz, 3H), 7.55 (ddd, *J* = 8.1, 7.3, 1.2 Hz, 1H), 7.52 – 7.47 (m, 1H), 7.43 (td, *J* = 7.5, 1.1 Hz, 1H), 7.29 – 7.23 (m, 2H), 2.28 ppm (s, 3H); ^{13}C NMR (150 MHz, 300 K, acetone-*d$_6$*): δ = 171.4, 145.3, 140.9, 137.2, 134.5 (q, *J* = 32.6 Hz), 132.8, 130.7 (2C), 130.0, 128.1, 128.0 (2C), 126.6, 125.3, 124.5 (q, *J* = 271.5 Hz), 123.3, 120.6, 117.8, 117.3, 21.4 ppm; note that the ^{13}C NMR signals corresponding to carbon C* and C ** were not visible, likely due to low sample concentration and/or peak broadening. ^{19}F NMR (565 MHz, 300 K, acetone-*d$_6$*): δ = -64.0 ppm (s, 3F); IR (film): ṽ = 3285, 2924, 1403, 1321, 1113, 1078 cm^{-1}; HRMS (ESI): m/z calculated for $C_{22}H_{15}F_3N_3O_3S_2$ [M-H]$^-$: 490.0512, found: 490.0535.

***N*-(4-(4-Methoxyphenyl)thiazol-2-yl)-2-((4-methylphenyl)sulfonamido)-4-(trifluoromethyl)benzamide (9)**

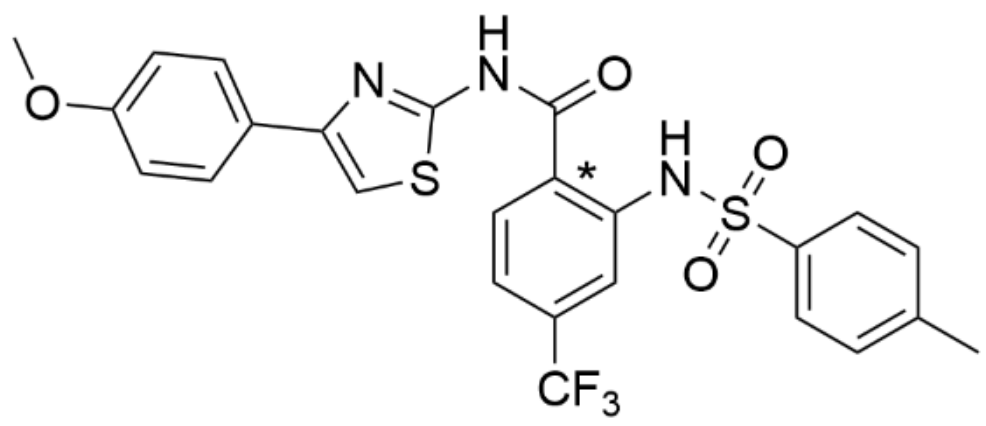

According to General Procedure B, amide **9** (88.0 mg, 0.16 mmol, 21%) was obtained from the reported sulfonamide **1a**[41] (270 mg, 0.75 mmol) and commercially-sourced 4-(4-methoxyphenyl)thiazol-2-amine (155 mg, 0.75 mmol), followed by reverse phase column chromatography (12 g Sfär C18 Duo cartridge; 12 mL/min; 100%$_{v/v}$ (H_2O + 0.1%$_{v/v}$ FA) (2 CV), followed by a gradient (8 CV): 0%$_{v/v}$→40%$_{v/v}$ and (20 CV): 40%$_{v/v}$→100%$_{v/v}$ (acetonitrile + 0.1%$_{v/v}$ FA) in (H_2O + 0.1%$_{v/v}$ FA)).

White solid; m.p.: >230 °C (decomposition); ^{1}H NMR (600 MHz, 300 K, DMSO-*d$_6$*): δ = 12.79 (s, 1H), 10.29 (s, 1H), 7.98 (s, 1H), 7.87 (d, *J* = 8.6 Hz, 2H), 7.63 – 7.57 (m, 4H), 7.51 (s, 1H, H-11), 7.30 – 7.25 (m, 2H), 7.02 (d, *J* = 8.7 Hz, 2H), 3.80 (s, 3H), 2.26 ppm (s, 3H); ^{13}C NMR (150 MHz, 300 K, DMSO-*d$_6$*): δ = 164.6, 159.1, 157.6, 149.1, 143.9, 136.8, 135.6, 131.8 (q, *J* = 32.2 Hz), 131.1, 129.8 (2C), 129.3, 127.2 (2C), 126.8 (2C), 123.2 (q, *J* = 272.7 Hz), 121.2, 119.2, 114.2 (2C), 106.8, 55.2, 20.9 ppm; note that the ^{13}C NMR signal corresponding to carbon C* was not visible, likely due to low sample concentration and/or peak broadening. ^{19}F NMR (565 MHz, 300 K, DMSO-*d$_6$*): δ = -62.5 ppm (s, 3F); IR (film): ṽ = 3688, 3350, 2980, 2924, 2853, 1650, 1580, 1464, 1439, 1391, 1338, 1261, 1175, 1126, 1027 cm^{-1}; HRMS (ESI): m/z calculated for $C_{25}H_{21}F_3N_3O_4S_2$ [M+H]$^+$: 548.0920, found: 548.0924.

***N*-(4-(4-Chlorophenyl)thiazol-2-yl)-2-((4-methylphenyl)sulfonamido)-4-(trifluoromethyl)benzamide (10)**

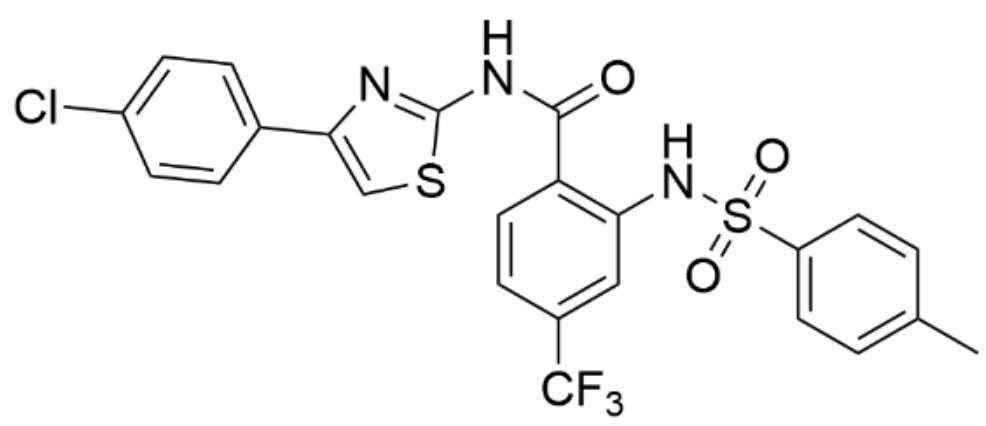

According to General Procedure B, amide **10** (108 mg, 0.20 mmol, 26%) was obtained from the reported sulfonamide **1a**[41] (270 mg, 0.75 mmol) and commercially-sourced 4-(4-chlorophenyl)thiazol-2-amine (158 mg, 0.75 mmol), followed by reverse phase column chromatography (12 g Sfär C18 Duo cartridge; 12 mL/min; 100%$_{v/v}$ (H_2O + 0.1%$_{v/v}$ FA) (2 CV), followed by a gradient (8 CV): 0%$_{v/v}$→40%$_{v/v}$ and (20

CV): 40$\%_{v/v}$→100$\%_{v/v}$ (acetonitrile + 0.1$\%_{v/v}$ FA) in (H_2O + 0.1$\%_{v/v}$ FA)) and subsequent flash column chromatography (10 g Sfär Silica Duo cartridge; 40 mL/min; 100$\%_{v/v}$ cyclohexane (2 CV), followed by a linear gradient (25 CV): 0$\%_{v/v}$→50$\%_{v/v}$ ethyl acetate in cyclohexane).

White solid; m.p.: 120–122 °C; ^{1}H NMR (400 MHz, 300 K, acetone-*d*$_6$): δ = 8.23 (dd, *J* = 8.1, 1.0 Hz, 1H), 8.01 – 7.93 (m, 3H), 7.71 (s, 1H), 7.68 – 7.58 (m, 3H), 7.52 – 7.43 (m, 2H), 7.27 – 7.19 (m, 2H), 2.24 ppm (s, 3H); ^{13}C NMR (100 MHz, 300 K, acetone-*d*$_6$): δ = 166.6, 156.9, 147.2, 145.5, 140.0, 136.7, 134.7 (q, *J* = 32.8 Hz),134.1, 134.0, 131.3, 130.8 (2C), 129.7 (2C), 128.4 (2C), 128.0 (2C), 126.4, 124.3 (q, *J* = 272.4 Hz), 121.6, 119.9, 110.3, 21.4 ppm; note that the ^{13}C NMR signals at 164.2 and 157.7 ppm were assigned using HMBC. ^{19}F NMR (375 MHz, 300 K, acetone-*d*$_6$): δ = -65.0 ppm (s, 3F); IR (film): ṽ = 3281, 2980, 2359, 1701, 1639, 1422, 1330, 1163, 1147, 1090, 1015 cm^{-1}; HRMS (ESI): m/z calculated for $C_{24}H_{18}ClF_3N_3O_3S_2$ $[M+H]^+$: 552.0425, found: 552.0412.

2-((4-Methylphenyl)sulfonamido)-*N*-(4-(4-nitrophenyl)thiazol-2-yl)-4-(trifluoromethyl)benzamide (11)

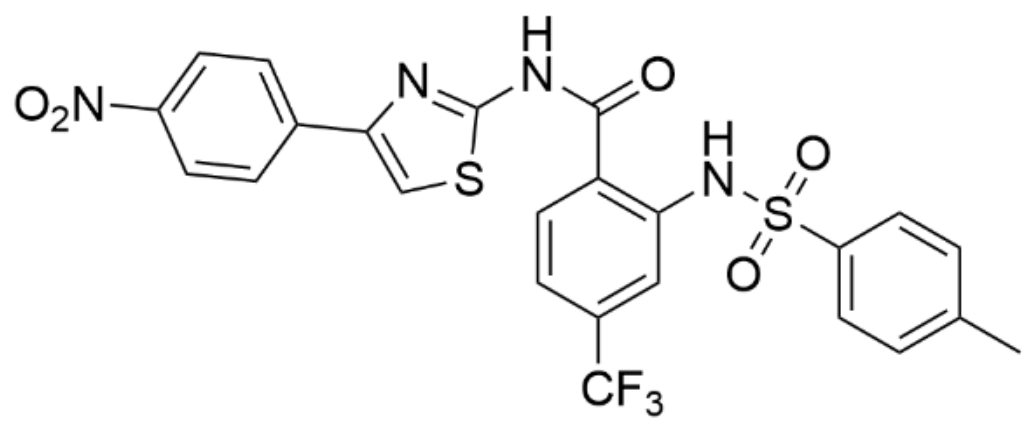

According to General Procedure B, amide **11** (118 mg, 0.21 mmol, 28%) was obtained from the reported sulfonamide **1a**[41] (270 mg, 0.75 mmol) and commercially-sourced 4-(4-nitrophenyl)thiazol-2-amine (155 mg, 0.75 mmol), followed by reverse phase column chromatography (12 g Sfär C18 Duo cartridge; 12 mL/min; 100$\%_{v/v}$ (H_2O + 0.1$\%_{v/v}$ FA) (2 CV), followed by a gradient (8 CV): 0$\%_{v/v}$→40$\%_{v/v}$ and (20 CV): 40$\%_{v/v}$→100$\%_{v/v}$ (acetonitrile + 0.1$\%_{v/v}$ FA) in (H_2O + 0.1$\%_{v/v}$ FA)).

White solid; m.p.: 167–169 °C; ^{1}H NMR (600 MHz, 300 K, DMSO-*d*$_6$): δ = 12.94 (s, 1H), 10.26 (s, 1H), 8.36 – 8.31 (m, 2H), 8.24 – 8.19 (m, 2H), 8.13 (app d, *J* = 3.2 Hz, 1H), 7.96 (d, *J* = 8.1 Hz, 1H), 7.60 (d, *J* = 8.0 Hz, 3H), 7.48 (s, 1H), 7.30 – 7.26 (m, 2H), 2.26 ppm (s, 3H); ^{13}C NMR (150 MHz, 300 K, DMSO-*d*$_6$): δ = 164.9, 163.0, 158.4, 146.9, 146.6, 143.9, 140.2, 136.6, 135.6, 132.1 (q, *J* = 32.2 Hz), 131.1, 129.7 (2C), 126.8 (2C), 126.6 (2C), 124.3 (2C), 123.2 (q, *J* = 273.0 Hz), 121.4, 119.4, 113.2, 20.9 ppm; ^{19}F NMR (565 MHz, 300 K, DMSO-*d*$_6$): δ = -62.0 ppm (s, 3F); IR (film): ṽ = 3657, 3279, 2981, 2900, 1935, 1916, 1799, 1640, 1522, 1445, 1392, 1258, 1148, 1109, 1020 cm^{-1}; HRMS (ESI): m/z calculated for $C_{24}H_{18}F_3N_4O_5S_2$ $[M+H]^+$: 563.0665, found: 563.0679.

***N*-(4-([1,1'-Biphenyl]-4-yl)thiazol-2-yl)-2-((4-methylphenyl)sulfonamido)-4-(trifluoromethyl)benzamide (12)**

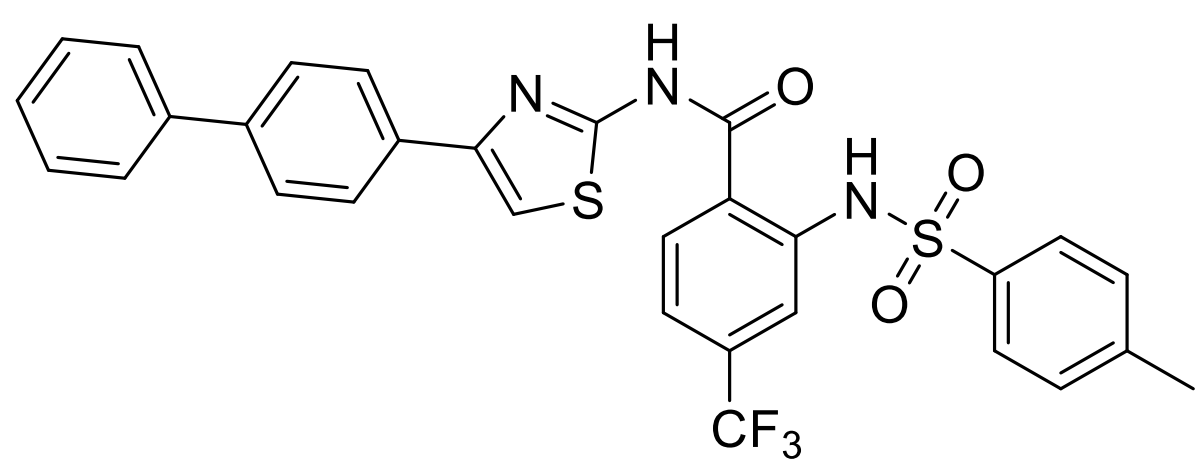

According to General Procedure B, amide **12** (78.0 mg, 0.13 mmol, 23%) was obtained from the reported sulfonamide **1a**[41] (200 mg, 0.56 mmol) and commercially-sourced 4-([1,1'-biphenyl]-4-yl)thiazol-2-amine (141 mg, 0.3 mmol), followed by reverse phase column chromatography (12 g Sfär C18 Duo cartridge; 12 mL/min; 100$\%_{v/v}$ (H_2O + 0.1$\%_{v/v}$ FA) (2 CV), followed by a gradient (8 CV): 0$\%_{v/v}$→40$\%_{v/v}$ and (20 CV): 40$\%_{v/v}$→100$\%_{v/v}$ (acetonitrile + 0.1$\%_{v/v}$ FA) in (H_2O + 0.1$\%_{v/v}$ FA)) and subsequent purification using flash column chromatography (10 g Sfär Silica Duo cartridge; 40 mL/min; 100$\%_{v/v}$ cyclohexane (2 CV), followed by a linear gradient (25 CV): 0$\%_{v/v}$→50$\%_{v/v}$ ethyl acetate in cyclohexane).

Yellow solid; m.p.: >240 °C (decomposition); ^{1}H NMR (600 MHz, 300 K, acetone-*d*$_6$): δ = 11.74 (s, 1H), 8.26 (d, *J* = 8.1 Hz, 1H), 8.07 – 8.02 (m, 2H), 7.97 (d, *J* = 1.7 Hz, 1H), 7.78 – 7.75 (m, 2H), 7.74 – 7.68 (m, 3H), 7.68 –

7.63 (m, 2H), 7.62 (dd, J = 8.5, 1.8 Hz, 1H), 7.51 – 7.45 (m, 2H), 7.41 – 7.35 (m, 1H), 7.24 (d, J = 8.0 Hz, 2H), 2.25 ppm (s, 3H); ^{13}C NMR (150 MHz, 300 K, acetone-d_6): δ = 166.7, 158.3, 148.6, 145.5, 141.5, 141.2, 140.0, 136.7, 134.6 (q, J = 32.8 Hz), 134.0, 131.4, 130.8 (2C), 129.8 (2C), 128.4, 128.1 (2C), 128.0 (2C), 127.6 (2C), 127.4 (2C), 126.5, 124.4 (q, J = 272.2 Hz), 121.6, 119.8, 109.7, 21.4 ppm; note that the ^{13}C NMR signals at 166.7, 158.3 and 148.6 ppm were assigned using HMBC. ^{19}F NMR (565 MHz, 300 K, acetone-d_6): δ = -64.0 ppm (s, 3F); IR (film): ṽ = 3257, 2959, 2851, 1731, 1597, 1550, 1480, 1446, 1375, 1219, 1164, 1103 cm^{-1}; HRMS (ESI): m/z calculated for $C_{30}H_{23}F_3N_3O_3S_2$ [M+H]$^+$: 594.1128, found: 594.1145.

2-((4-Methylphenyl)sulfonamido)-*N*-(4-(naphthalen-1-yl)thiazol-2-yl)-4-(trifluoromethyl)benzamide (13)

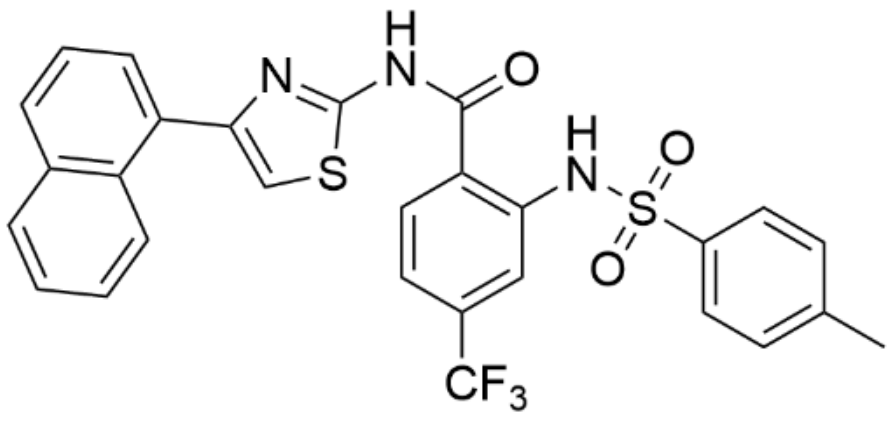

According to General Procedure B, amide **13** (55.0 mg, 0.096 mmol, 13%) was obtained from the reported sulfonamide **1a**[41] (270 mg, 0.75 mmol) and commercially-sourced ethyl 2-(2-aminothiazol-4-yl)acetate 4-(naphthalen-1-yl)thiazol-2-amine (170 mg, 0.75 mmol), followed by reverse phase column chromatography (12 g Sfär C18 Duo cartridge; 12 mL/min; 100%$_{v/v}$ (H_2O + 0.1%$_{v/v}$ FA) (2 CV), followed by a gradient (8 CV): 0%$_{v/v}$→40%$_{v/v}$ and (20 CV): 40%$_{v/v}$→100%$_{v/v}$ (acetonitrile + 0.1%$_{v/v}$ FA) in (H_2O + 0.1%$_{v/v}$ FA)) and subsequent purification using HPLC (phenyl-hexyl HPLC column (250 mm length, 19 mm internal diameter, 5 µm particle size); 10 mL/min, 25%$_{v/v}$→80%$_{v/v}$ (100 min) acetonitrile in (H_2O + 0.1%$_{v/v}$ FA)).

White solid; m.p.: >230 °C (decomposition); ^{1}H NMR (600 MHz, 300 K, DMSO-d_6): δ = 12.92 (s, 1H), 10.33 (s, 1H), 8.38 (s, 1H), 8.09 – 7.92 (m, 3H), 7.74 (dd, J = 7.1, 1.2 Hz, 1H), 7.67 – 7.42 (m, 8H), 7.30 (d, J = 8.0 Hz, 2H), 2.28 ppm (s, 3H); ^{13}C NMR (150 MHz, 300 K, DMSO-d_6): δ = 164.6, 157.4, 149.0, 144.0, 136.8, 135.7, 133.5, 132.6, 131.8 (q, J = 32.2 Hz), 131.2, 130.7, 129.8 (2C), 129.4, 128.6, 128.4, 127.4, 126.9 (2C), 126.4, 126.0, 125.7, 125.5, 123.2 (q, J = 272.6 Hz), 121.2, 119.2, 112.7, 21.0 ppm; ^{19}F NMR (375 MHz, 300 K, DMSO-d_6): δ = -62.0 ppm (s, 3F); IR (film): ṽ = 3651, 2981, 2888, 1771, 1640, 1595, 1493, 1391, 1336, 1253, 1239, 1121 cm^{-1}; HRMS (ESI): m/z calculated for $C_{28}H_{21}F_3N_3O_3S_2$ [M+H]$^+$: 568.0971, found: 568.0972.

2-((4-Methylbenzyl)amino)-4-(trifluoromethyl)benzoic acid (14a)

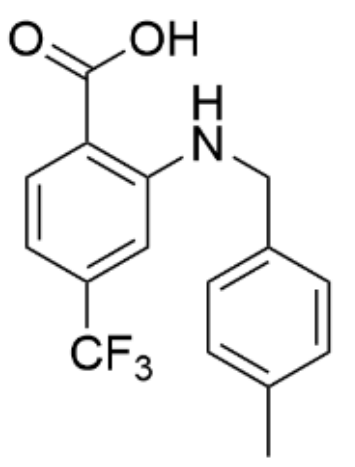

Acetic acid (5%$_{v/v}$) was added to a solution of 2-amino-5-(trifluoromethyl)benzoic acid (1.03 g, 5.0 mmol, 1.0 equiv.) and 4-methylbenzaldehyde (595 µL, 5.0 mmol, 1.0 equiv.) in anhydrous methanol (0.2 M) and the resultant mixture was refluxed for 8 h, before being cooled to 60 °C. Then $NaBH_3CN$ (624 mg, 10 mmol, 2.0 equiv.) was added slowly and the mixture was stirred at 60 °C for 16 h before being concentrated, washed with saturated aqueous $NaHCO_3$ (2 times), brine (2 times) and dried over $MgSO_4$. The crude residue was purified using reverse phase column chromatography (30 g Sfär C18 Duo cartridge; 30 mL/min; 100%$_{v/v}$ (H_2O + 0.1%$_{v/v}$ FA) (2 CV), followed by a gradient (30 CV): 0%$_{v/v}$→100%$_{v/v}$ (acetonitrile + 0.1%$_{v/v}$ FA) in (H_2O + 0.1%$_{v/v}$ FA)) to yield the desired secondary amine (488 mg, 1.6 mmol, 32%).

White solid; m.p.: 165–167 °C; ^{1}H NMR (400 MHz, 300 K, MeOD-d_4): δ = 8.03 (dt, J = 8.5, 1.0 Hz, 1H), 7.23 (d, J = 8.0 Hz, 2H), 7.19 – 7.12 (m, 2H), 6.88 (d, J = 1.7 Hz, 1H), 6.78 (dd, J = 8.3, 1.7 Hz, 1H), 4.39 (s, 2H), 2.30 ppm (s, 3H); ^{13}C NMR (100 MHz, 300 K, MeOD-d_4): δ = 171.2, 152.2, 138.1, 136.6, 136.5 (q, J = 32.2 Hz), 134.1, 130.4 (2C), 128.2 (2C), 125.3 (q, J = 272.2 Hz), 114.4, 111.4 (q, J = 3.7 Hz), 109.2 (q, J = 4.2 Hz), 47.4, 21.1 ppm; ^{19}F NMR (375 MHz, 300 K, MeOD-d_4): δ = -65.3 ppm (s, 3F); IR (film): ṽ = 3387, 1665, 1582, 1335, 1249, 1230, 1120 cm^{-1}; HRMS (ESI): m/z calculated for $C_{16}H_{15}F_3NO_2$ [M+H]$^+$: 310.1049, found: 310.1036.

***N*-(4-(4-Chlorophenyl)thiazol-2-yl)-2-((4-methylbenzyl)amino)-4-(trifluoromethyl)benzamide (14)**

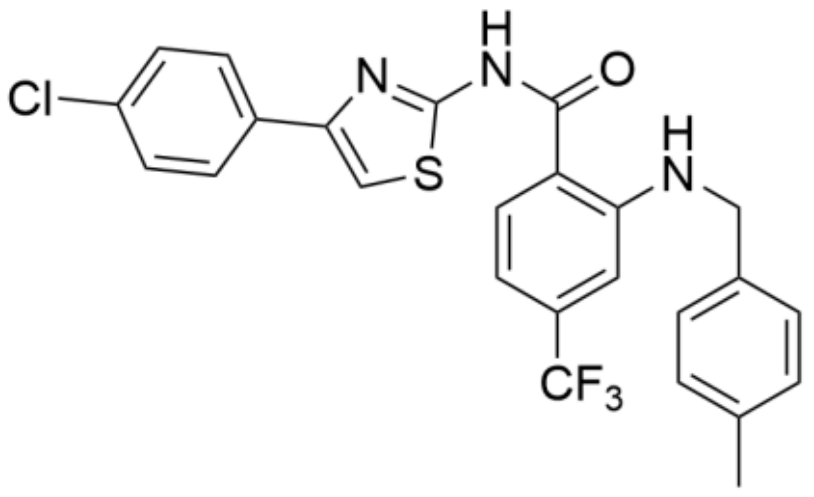

According to General Procedure B, amide **14** (92.9 mg, 0.18 mmol, 37%) was obtained from the carboxylic acid **14a** (155 mg, 0.50 mmol) and commercially-sourced 4-(4-chlorophenyl)thiazol-2-amine (105 mg, 0.5 mmol), followed by reverse phase column chromatography (12 g Sfär C18 Duo cartridge; 12 mL/min; 100%$_{v/v}$ (H_2O + 0.1%$_{v/v}$ FA) (2 CV), followed by a gradient (8 CV): 0%$_{v/v}$→40%$_{v/v}$ and (20 CV): 40%$_{v/v}$→100%$_{v/v}$ (acetonitrile + 0.1%$_{v/v}$ FA) in (H_2O + 0.1%$_{v/v}$ FA)) and subsequent purification using flash column chromatography (10 g Sfär Silica Duo cartridge; 40 mL/min; 100%$_{v/v}$ cyclohexane (2 CV), followed by a linear gradient (25 CV): 0%$_{v/v}$→50%$_{v/v}$ ethyl acetate in cyclohexane).

White solid; m.p.: 94–96 °C; ^{1}H NMR (600 MHz, 300 K, acetone-*d$_6$*): δ = 11.52 (s, 1H), 8.38 – 8.33 (m, 1H), 8.21 (d, *J* = 8.2 Hz, 1H), 7.99 – 7.93 (m, 2H), 7.59 (s, 1H), 7.49 – 7.43 (m, 2H), 7.38 – 7.34 (m, 2H), 7.20 (d, *J* = 7.8 Hz, 2H), 7.08 (d, *J* = 1.7 Hz, 1H), 6.94 (dd, *J* = 8.2, 1.7 Hz, 1H), 4.60 – 4.56 (m, 2H), 2.32 ppm (s, 3H); ^{13}C NMR (150 MHz, 300 K, acetone-*d$_6$*): δ = 167.6, 159.0, 151.1, 149.5, 137.7, 136.5, 135.4 (q, *J* = 31.8 Hz), 134.4, 133.9, 131.2, 130.2 (2C), 129.6 (2C), 128.4 (2C), 128.3 (2C), 124.9 (q, *J* = 272.4 Hz), 116.4, 111.5, 109.5 (2C), 47.2, 21.1 ppm; ^{19}F NMR (565 MHz, 300 K, acetone-*d$_6$*): δ = -64.3 ppm (s, 3F); IR (film): ṽ = 3017, 2918, 2849, 1562, 1578, 1528, 1447, 1336, 1277, 1175, 1130, 1051 cm^{-1}; HRMS (ESI): m/z calculated for $C_{25}H_{20}ClF_3N_3OS$ $[M+H]^+$: 502.0962, found: 502.0974.

***N*-(4-(4-Chlorophenyl)thiazol-2-yl)-2-(4-methylbenzamido)-4-(trifluoromethyl)benzamide (15)**

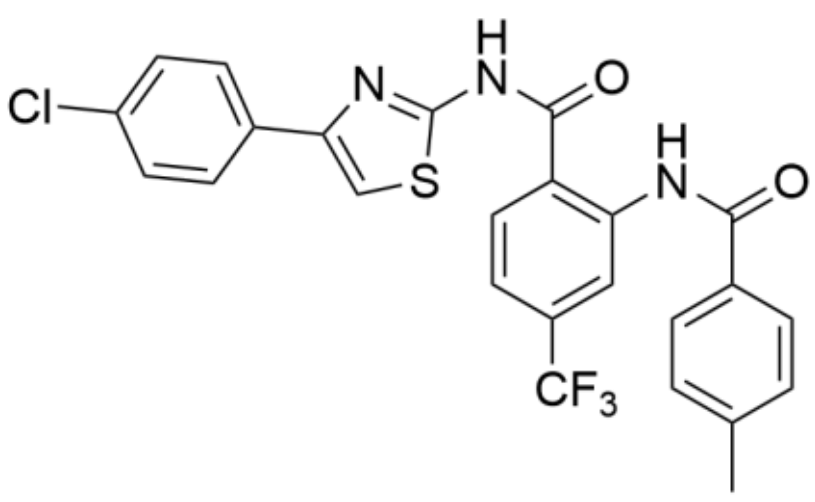

According to General Procedure B, amide **15** (42.6 mg, 0.082 mmol, 17%) was obtained from the reported amide **15a**[43] (162 mg, 0.50 mmol) and commercially-sourced 4-(4-chlorophenyl)thiazol-2-amine (105 mg, 0.50 mmol), followed by reverse phase column chromatography (12 g Sfär C18 Duo cartridge; 12 mL/min; 100%$_{v/v}$ (H_2O + 0.1%$_{v/v}$ FA) (2 CV), followed by a gradient (8 CV): 0%$_{v/v}$→40%$_{v/v}$ and (20 CV): 40%$_{v/v}$→100%$_{v/v}$ (acetonitrile + 0.1%$_{v/v}$ FA) in (H_2O + 0.1%$_{v/v}$ FA)).

White solid; m.p.: > 220 °C (decomposition); ^{1}H NMR (400 MHz, 300 K, acetone-*d$_6$*): δ = 11.86 (s, 2H), 9.24 (d, *J* = 1.8 Hz, 1H), 8.44 (d, *J* = 8.2 Hz, 1H), 8.02 – 7.91 (m, 4H), 7.69 (s, 1H), 7.60 (ddd, *J* = 8.2, 1.9, 0.8 Hz, 1H), 7.50 – 7.39 (m, 4H), 2.44 ppm (s, 3H); ^{13}C NMR (100 MHz, 300 K, acetone-*d$_6$*): δ = 167.6, 166.1, 159.0, 149.5, 143.9, 141.9, 134.8 (q, *J* = 32.6 Hz), 134.2, 134.0, 132.6, 131.0, 130.4 (2C), 129.6 (2C), 128.4 (2C), 128.3 (2C), 124.7 (q, *J* = 272.2 Hz), 123.1, 119.9, 118.3, 110.2, 21.5 ppm; ^{19}F NMR (375 MHz, 300 K, acetone-*d$_6$*): δ = -63.9 ppm (s, 3F); IR (film): ṽ = 3037, 2942, 2843, 1769, 1683, 1535, 1480, 1432, 1336, 1181, 1134 cm^{-1}; HRMS (ESI): m/z calculated for $C_{25}H_{18}ClF_3N_3O_2S$ $[M+H]^+$: 516.0755, found: 516.0767.

2-((4-(*tert*-Butyl)phenyl)sulfonamido)-4-(trifluoromethyl)benzoic acid (16a)

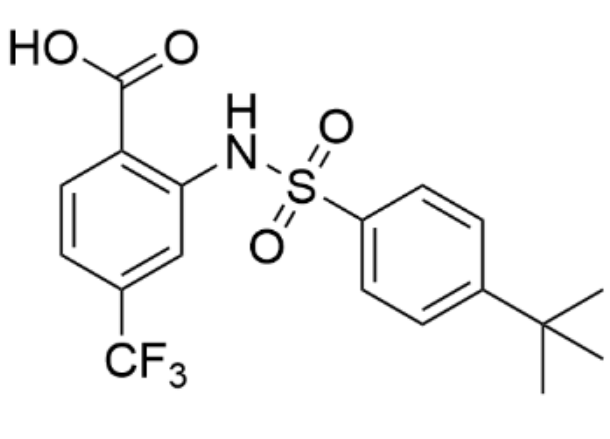

According to General Procedure A, sulfonamide **16a** (349 mg, 0.85 mmol, 85%) was obtained from commercially-sourced 2-amino-4-(trifluoromethyl)benzoic acid (205 mg, 1.0 mmol) and 4-(*tert*-butyl)benzenesulfonyl chloride (233 mg, 1.0 mmol), followed by reverse phase column chromatography (12 g Sfär C18 Duo cartridge; 12 mL/min; 100%$_{v/v}$ (H_2O + 0.1%$_{v/v}$ FA) (2 CV), followed by a gradient (30 CV): 0%$_{v/v}$→100%$_{v/v}$ (acetonitrile + 0.1%$_{v/v}$ FA) in (H_2O + 0.1%$_{v/v}$ FA)).

Brown solid; m.p.: >250 °C (decomposition); ^{1}H NMR (600 MHz, 300 K, MeOD-d_4): δ = 8.10 (d, J = 8.2 Hz, 1H), 7.90 (d, J = 1.7 Hz, 1H), 7.77 – 7.71 (m, 2H), 7.56 – 7.50 (m, 2H), 7.32 – 7.27 (m, 1H), 1.28 ppm (s, 9H); ^{13}C NMR (150 MHz, 300 K, MeOD-d_4): δ = 171.3, 158.5, 142.0, 137.5, 135.0 (q, J = 32.6 Hz), 133.7, 128.2, 127.4, 127.2, 124.9 (q, J = 271.2 Hz), 124.7, 120.2, 120.0, 116.4, 36.0, 31.4 ppm (3C); ^{19}F NMR (565 MHz, 300 K, MeOD-d_4): δ = -65.7 ppm (s, 3F); IR (film): ṽ = 3456, 3006, 2970, 2948, 1738, 1526, 1442, 1228, 1216 cm^{-1}; HRMS (ESI): m/z calculated for $C_{18}H_{17}F_3NO_4S$ [M-H]$^-$: 400.0836, found: 400.0822.

2-((4-(*tert*-Butyl)phenyl)sulfonamido)-*N*-(4-(4-chlorophenyl)thiazol-2-yl)-4-(trifluoromethyl)benzamide (16)

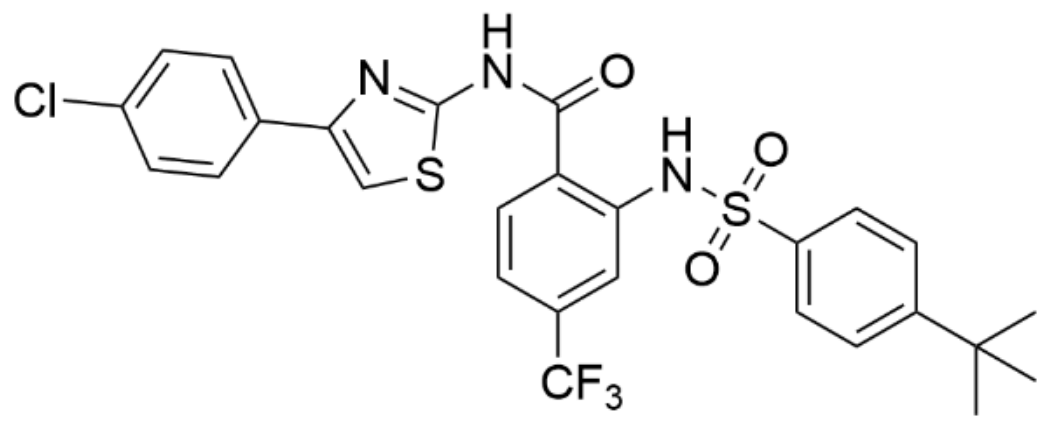

According to General Procedure B, amide **16** (29.0 mg, 0.049 mmol, 16%) was obtained from the sulfonamide **16a** (108 mg, 0.30 mmol) and commercially-sourced 4-(4-chlorophenyl)thiazol-2-amine (63.2 mg, 0.30 mmol), followed by reverse phase column chromatography (12 g Sfär C18 Duo cartridge; 12 mL/min; 100%$_{v/v}$ (H_2O + 0.1%$_{v/v}$ FA) (2 CV), followed by a gradient (8 CV): 0%$_{v/v}$→40%$_{v/v}$ and (20 CV): 40%$_{v/v}$→100%$_{v/v}$ (acetonitrile + 0.1%$_{v/v}$ FA) in (H_2O + 0.1%$_{v/v}$ FA)).

White solid; m.p.: >220 °C (decomposition); ^{1}H NMR (400 MHz, 300 K, DMSO-d_6): δ = 12.83 (s, 1H), 10.25 (s, 1H), 8.03 – 7.88 (m, 3H), 7.83 (s, 1H), 7.68 – 7.60 (m, 3H), 7.55 – 7.46 (m, 4H), 7.41 (s, 1H), 1.19 ppm (s, 9H); ^{13}C NMR (150 MHz, 300 K, DMSO-d_6): δ = 164.6, 157.9, 156.6, 147.9, 136.6, 135.6, 133.1, 132.4, 131.8 (q, J = 32.3 Hz), 131.1, 130.2, 128.8 (2C), 127.5 (2C), 126.8 (2C), 126.1 (2C), 123.2 (q, J = 272.7 Hz), 121.6, 120.1, 109.6, 34.8, 30.6 (3C) ppm; ^{19}F NMR (375 MHz, 300 K, DMSO-d_6): δ = -62.0 ppm (s, 3F); IR (film): ṽ = 3453, 3258, 3016, 2970, 2948 1748, 1728, 1649, 1558, 1368, 1228, 1206 cm^{-1}; HRMS (ESI): m/z calculated for $C_{27}H_{24}ClF_3N_3O_3S_2$ [M+H]$^+$: 594.0894, found: 594.0910.

2-((4-Methoxyphenyl)sulfonamido)-4-(trifluoromethyl)benzoic acid (17a)

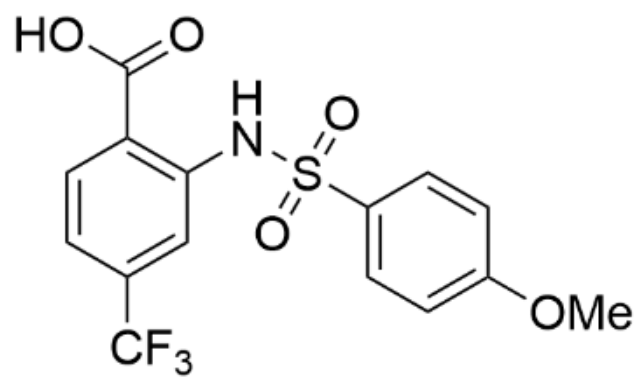

According to General Procedure A, sulfonamide **17a** (286 mg, 0.76 mmol, 76%) was obtained from commercially-sourced 2-amino-4-(trifluoromethyl)benzoic acid (205 mg, 1.0 mmol) and 4-methoxybenzenesulfonyl chloride (206 mg, 1.0 mmol), followed by reverse phase column chromatography (12 g Sfär C18 Duo cartridge; 12 mL/min; 100%$_{v/v}$ (H_2O + 0.1%$_{v/v}$ FA) (2 CV), followed by a gradient (30 CV): 0%$_{v/v}$→100%$_{v/v}$ (acetonitrile + 0.1%$_{v/v}$ FA) in (H_2O + 0.1%$_{v/v}$ FA)).

Yellow solid; m.p.: >210 °C (decomposition); ^{1}H NMR (600 MHz, 300 K, MeOD-d_4): δ = 8.11 (d, J = 8.2 Hz, 1H), 7.90 (d, J = 1.8 Hz, 1H), 7.78 – 7.71 (m, 2H), 7.34 – 7.30 (m, 1H), 7.01 – 6.96 (m, 2H), 3.80 ppm (s, 3H); ^{13}C NMR (150 MHz, 300 K, MeOD-d_4): δ = 170.8, 165.1, 142.2, 135.5 (q, J = 32.6 Hz), 133.8, 131.5, 130.5 (2C), 124.8 (q, J = 272.1 Hz), 123.3, 120.3 (q, J = 3.8 Hz), 116.7 (q, J = 3.9 Hz), 115.4 (2C), 56.2 ppm; ^{19}F NMR (565 MHz, 300 K, MeOD-d_4): δ = -65.8 ppm (s, 3F); IR (film): ṽ = 3457, 3015, 2970, 2947, 1738, 1597, 1500, 1423, 1229, 1154, 1092 cm^{-1}; HRMS (ESI): m/z calculated for $C_{15}H_{11}F_3NO_5S$ [M-H]$^-$: 374.0315, found: 374.0318.

***N*-(4-(4-Chlorophenyl)thiazol-2-yl)-2-((4-methoxyphenyl)sulfonamido)-4-(trifluoromethyl)benzamide (17)**

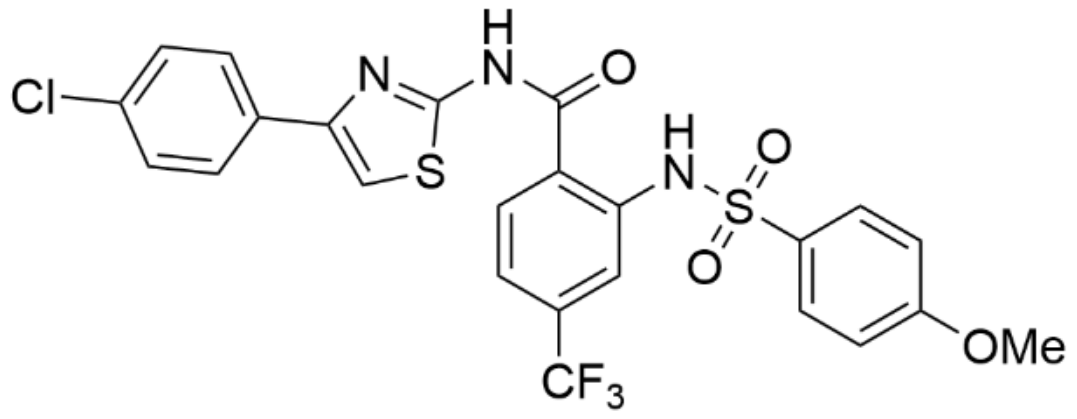

According to General Procedure B, amide **17** (43.3 mg, 0.076 mmol, 25%) was obtained from the carboxylic acid **17a** (112 mg, 0.30 mmol) and commercially-sourced 4-(4-chlorophenyl)thiazol-2-amine (63.2 mg, 0.30 mmol), followed by reverse phase column chromatography (12 g Sfär C18 Duo cartridge; 12 mL/min; 100%$_{v/v}$ (H_2O + 0.1%$_{v/v}$ FA) (2 CV), followed by a gradient (8 CV): 0%$_{v/v}$→40%$_{v/v}$ and (20 CV): 40%$_{v/v}$→100%$_{v/v}$ (acetonitrile + 0.1%$_{v/v}$ FA) in (H_2O + 0.1%$_{v/v}$ FA)).

White solid; m.p.: 182–184 °C; ^{1}H NMR (600 MHz, 300 K, acetone-*d$_6$*): δ = 8.24 (d, *J* = 8.1 Hz, 1H), 7.99 – 7.93 (m, 3H), 7.70 (dd, *J* = 6.6, 2.4 Hz, 3H), 7.61 (d, *J* = 8.2 Hz, 1H), 7.50 – 7.44 (m, 2H), 6.95 – 6.90 (m, 2H), 3.71 ppm (s, 3H); ^{13}C NMR (150 MHz, 300 K, acetone-*d$_6$*): δ = 166.6, 164.5, 157.8, 148.4, 140.6, 134.6 (q, *J* = 32.9 Hz), 134.1, 134.0, 131.3, 131.1, 130.3 (2C), 129.7 (2C), 128.4 (2C), 126.3, 124.4 (q, *J* = 272.3 Hz), 121.5, 119.8, 115.3 (2C), 110.3, 56.1 ppm; note that the ^{13}C NMR signals at 157.8 and 148.4 ppm were assigned using HMBC. ^{19}F NMR (565 MHz, 300 K, acetone-*d$_6$*): δ = -64.0 ppm (s, 3F); IR (film): ṽ = 3460, 3025, 2970, 2945, 1738, 1436, 1366, 1228, 1216 cm^{-1}; HRMS (ESI): m/z calculated for $C_{24}H_{18}ClF_3N_3O_4S_2$ $[M+H]^+$: 568.0374, found: 568.0371.

***N*-(4-(4-Chlorophenyl)thiazol-2-yl)-2-((4-nitrophenyl)sulfonamido)-4-(trifluoromethyl)benzamide (18)**

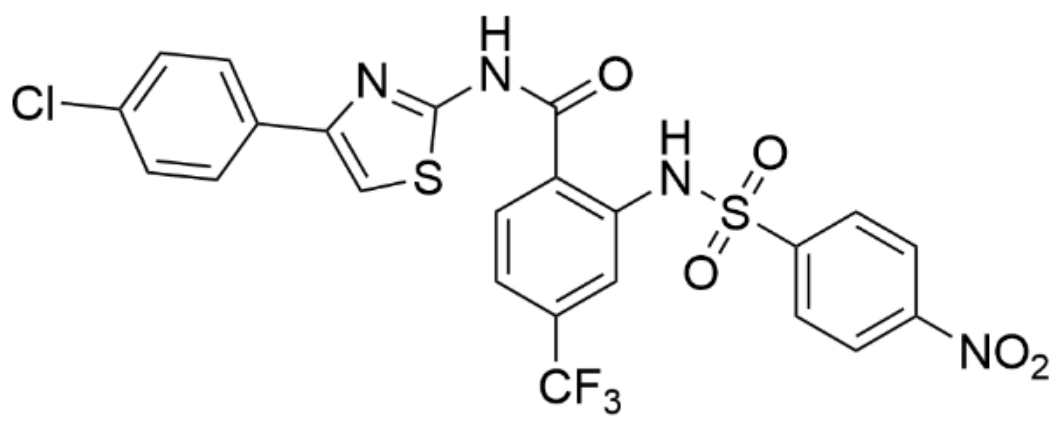

According to General Procedure B, amide **18** (42.9 mg, 0.073 mmol, 24%) was obtained from the reported carboxylic acid **18a**[44] (108 mg, 0.30 mmol) and commercially-sourced 4-(4-chlorophenyl)thiazol-2-amine (63.2 mg, 0.30 mmol), followed by reverse phase column chromatography (12 g Sfär C18 Duo cartridge; 12 mL/min; 100%$_{v/v}$ (H_2O + 0.1%$_{v/v}$ FA) (2 CV), followed by a gradient (8 CV): 0%$_{v/v}$→40%$_{v/v}$ and (20 CV): 40%$_{v/v}$→100%$_{v/v}$ (acetonitrile + 0.1%$_{v/v}$ FA) in (H_2O + 0.1%$_{v/v}$ FA)).

Yellow solid; m.p.: >200 °C (decomposition); ^{1}H NMR (600 MHz, 300 K, CD_3CN): δ = 8.10 – 8.05 (m, 2H), 7.91 – 7.89 (m, 1H), 7.88 (d, *J* = 8.1 Hz, 1H), 7.87 – 7.83 (m, 2H), 7.83 – 7.79 (m, 2H), 7.70 – 7.65 (m, 1H), 7.49 – 7.43 ppm (m, 3H); ^{13}C NMR (150 MHz, 300 K, CD_3CN): δ = 166.0, 156.8, 151.3, 147.4, 144.6, 138.1, 134.8 (q, *J* = 33.0 Hz), 134.3, 133.7, 131.3, 129.7 (2C), 129.5 (2C), 129.1, 128.5 (3C), 125.5 (2C), 124.3 (q, *J* = 272.2 Hz), 122.9, 110.3 ppm; ^{19}F NMR (565 MHz, 300 K, CD_3CN): δ = -64.8 ppm (s, 3F); IR (film): ṽ = 2979, 2961, 1812, 1662, 1535, 1404, 1329, 1264, 1169, 1135, 1091 cm^{-1}; HRMS (ESI): m/z calculated for $C_{23}H_{15}ClF_3N_4O_5S_2$ $[M+H]^+$: 583.0119, found: 583.0108.

2-((2-Methylphenyl)sulfonamido)-4-(trifluoromethyl)benzoic acid (19a)

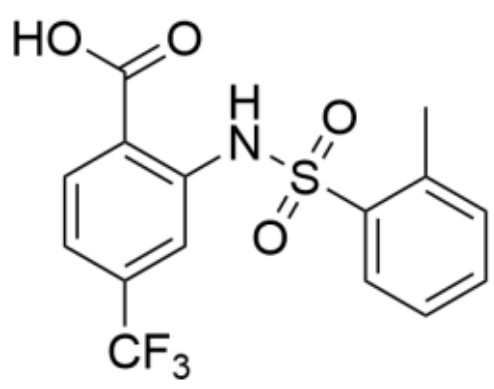

According to General Procedure A, sulfonamide **19a** (267 mg, 0.76 mmol, 76%) was obtained from commercially-sourced 2-amino-4-(trifluoromethyl)benzoic acid (205 mg, 1.0 mmol) and 2-methylbenzenesulfonyl chloride (191 mg, 1.0 mmol), followed by reverse phase column chromatography (12 g Sfär C18 Duo cartridge; 12 mL/min; 100%$_{v/v}$ (H_2O + 0.1%$_{v/v}$ FA) (2 CV), followed by a gradient (30 CV): 0%$_{v/v}$→100%$_{v/v}$ (acetonitrile + 0.1%$_{v/v}$ FA) in (H_2O + 0.1%$_{v/v}$ FA)).

Yellow solid; m.p.: 181–183 °C; ^{1}H NMR (600 MHz, 300 K, MeOD-*d$_4$*): δ = 8.16 (dd, *J* = 8.3, 1.0 Hz, 1H), 8.03

(dd, J = 7.9, 1.3 Hz, 1H), 7.79 (d, J = 1.7 Hz, 1H), 7.50 (td, J = 7.5, 1.4 Hz, 1H), 7.41 – 7.33 (m, 2H), 7.32 – 7.28 (m, 1H), 2.62 ppm (s, 3H); ^{13}C NMR (150 MHz, 300 K, MeOD-d_4): δ = 170.4, 142.1, 138.8, 137.9, 136.3 (q, J = 32.7 Hz), 135.0, 134.2, 134.0, 131.2, 127.3, 124.6 (q, J = 272.1 Hz), 121.9, 119.9, 115.2, 20.1 ppm; ^{19}F NMR (565 MHz, 300 K, MeOD-d_4): δ = -66.1 ppm (s, 3F); IR (film): ṽ = 3453, 3016, 2970, 2949, 1739, 1441, 1216 cm^{-1}; HRMS (ESI): m/z calculated for $C_{15}H_{11}F_3NO_4S$ [M-H]$^-$: 358.0366, found: 358.0380.

_N_-(4-(4-Chlorophenyl)thiazol-2-yl)-2-((2-methylphenyl)sulfonamido)-4-(trifluoromethyl)benzamide (19)

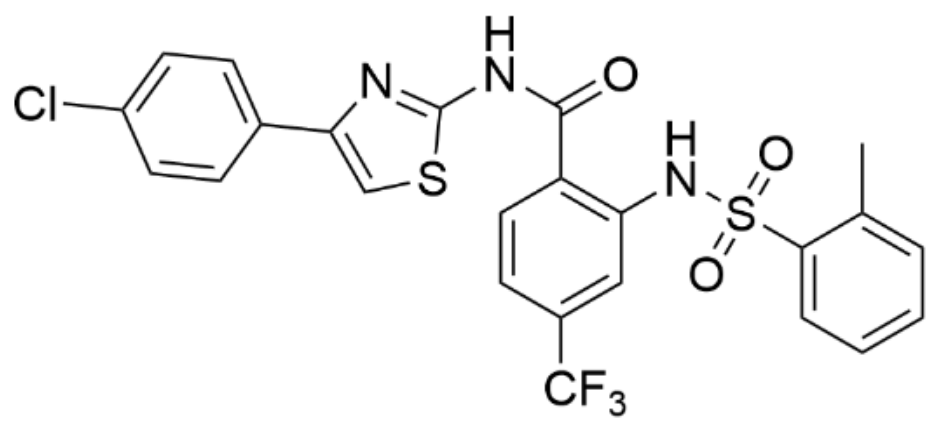

According to General Procedure B, amide **19** (43.9 mg, 0.079 mmol, 27%) was obtained from the carboxylic acid **19a** (108 mg, 0.30 mmol) and commercially-sourced 4-(4-chlorophenyl)thiazol-2-amine (63.2 mg, 0.30 mmol), followed by reverse phase column chromatography (12 g Sfär C18 Duo cartridge; 12 mL/min; 100%$_{v/v}$ (H_2O + 0.1%$_{v/v}$ FA) (2 CV), followed by a gradient (8 CV): 0%$_{v/v}$→40%$_{v/v}$ and (20 CV): 40%$_{v/v}$→100%$_{v/v}$ (acetonitrile + 0.1%$_{v/v}$ FA) in (H_2O + 0.1%$_{v/v}$ FA)).

Yellow solid; m.p.: >250 °C (decomposition); ^{1}H NMR (600 MHz, 300 K, acetone-d_6): δ = 8.33 (d, J = 8.2 Hz, 1H), 8.00 (dd, J = 8.0, 1.4 Hz, 1H), 7.97 (d, J = 2.0 Hz, 2H), 7.90 (d, J = 1.7 Hz, 1H), 7.71 (s, 1H), 7.56 – 7.45 (m, 4H), 7.39 (t, J = 7.7 Hz, 1H), 7.34 (d, J = 7.5 Hz, 1H), 2.66 ppm (s, 3H); ^{13}C NMR (150 MHz, 300 K, acetone-d_6): δ = 166.1, 154.2, 149.0, 139.1, 138.4, 136.8, 134.7, 134.2, 133.9, 131.6, 130.7, 130.6 (2C), 129.7 (2C), 128.4 (2C), 127.3, 125.2, 124.0 (q, J = 270.3 Hz), 120.6, 117.2, 110.3, 20.3 ppm; ^{19}F NMR (565 MHz, 300 K, acetone-d_6): δ = -64.2 ppm (s, 3F); IR (film): ṽ = 3456, 3016, 2970, 2946, 1738, 1435, 1366, 1229, 1206 cm^{-1}; HRMS (ESI): m/z calculated for $C_{24}H_{18}ClF_3N_3O_3S_2$ [M+H]$^+$: 552.0425, found: 552.0414.

2-(Naphthalene-1-sulfonamido)-4-(trifluoromethyl)benzoic acid (20a)

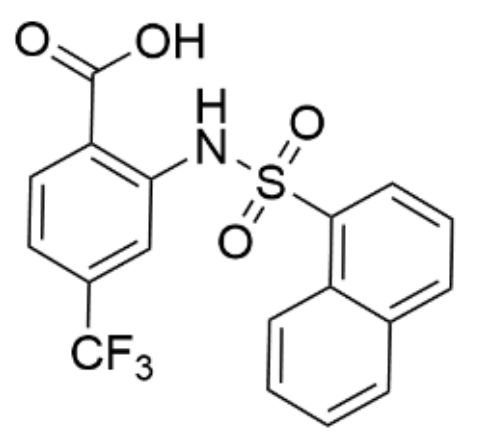

According to General Procedure A, sulfonamide **20a** (217 mg, 0.55 mmol, 55%) was obtained from commercially-sourced 2-amino-4-(trifluoromethyl)benzoic acid (205 mg, 1.0 mmol) and naphthalene-1-sulfonyl chloride (233 mg, 1.0 mmol), followed by reverse phase column chromatography (12 g Sfär C18 Duo cartridge; 12 mL/min; 100%$_{v/v}$ (H_2O + 0.1%$_{v/v}$ FA) (2 CV), followed by a gradient (30 CV): 0%$_{v/v}$→100%$_{v/v}$ (acetonitrile + 0.1%$_{v/v}$ FA) in (H_2O + 0.1%$_{v/v}$ FA)). The analytical data of **20a** are in agreement with those reported.[45]

Yellow solid; m.p.: > 200 °C (decomposition); ^{1}H NMR (600 MHz, 300 K, MeOD-d_4): δ = 8.68 (dt, J = 8.6, 0.9 Hz, 1H), 8.33 (dd, J = 7.4, 1.2 Hz, 1H), 8.12 – 8.08 (m, 1H), 7.97 (d, J = 8.1 Hz, 1H), 7.95 – 7.91 (m, 1H), 7.77 (d, J = 1.8 Hz, 1H), 7.67 (ddd, J = 8.6, 6.9, 1.4 Hz, 1H), 7.61 – 7.51 (m, 2H), 7.16 – 7.11 ppm (m, 1H); ^{13}C NMR (150 MHz, 300 K, MeOD-d_4): δ = 171.6, 141.7, 136.1, 135.7, 135.0, 134.4 (q, J = 32.5 Hz), 133.5, 131.8, 130.1, 129.5, 129.1, 128.1, 125.5, 125.0, 124.9 (q, J = 270.8 Hz), 124.9, 119.3 (q, J = 3.8 Hz), 114.8 ppm (q, J = 4.0 Hz); ^{19}F NMR (565 MHz, 300 K, MeOD-d_4): δ = -65.7 ppm (s, 3F); IR (film): ṽ = 3463, 3016, 2970, 2947, 1739, 1448, 1297 cm^{-1}; HRMS (ESI): m/z calculated for $C_{18}H_{11}F_3NO_4S$ [M-H]$^-$: 394.0366, found: 394.0372.

N-(4-(4-Chlorophenyl)thiazol-2-yl)-2-(naphthalene-1-sulfonamido)-4-(trifluoromethyl)benzamide (20)

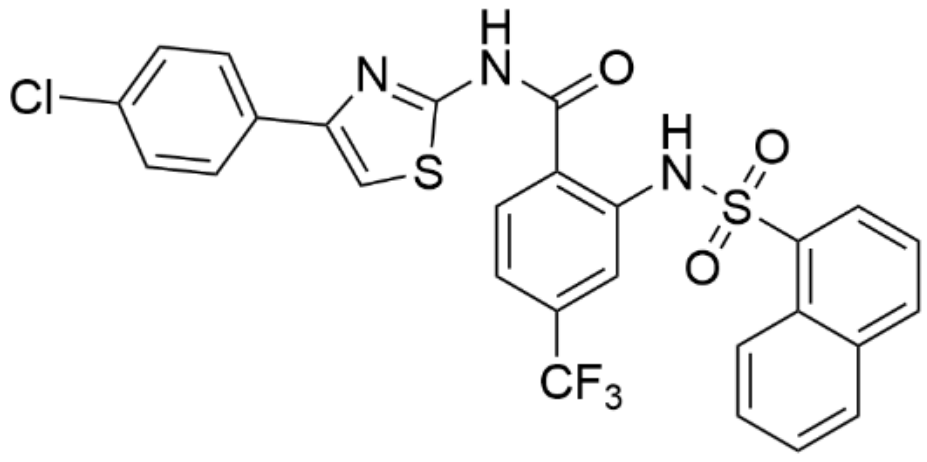

According to General Procedure B, amide **20** (35.3 mg, 0.060 mmol, 20%) was obtained from the carboxylic acid **20a** (119 mg, 0.30 mmol) and commercially sourced 4-(4-chlorophenyl)thiazol-2-amine (63.2 mg, 0.30 mmol), followed by reverse phase column chromatography (12 g Sfär C18 Duo cartridge; 12 mL/min; 100%$_{v/v}$ (H_2O + 0.1%$_{v/v}$ FA) (2 CV), followed by a gradient (8 CV): 0%$_{v/v}$→40%$_{v/v}$ and (20 CV): 40%$_{v/v}$→100%$_{v/v}$ (acetonitrile + 0.1%$_{v/v}$ FA) in (H_2O + 0.1%$_{v/v}$ FA)).

Brown solid; m.p.: > 250 °C (decomposition); ^{1}H NMR (500 MHz, 300 K, acetone-d_6): δ = 11.57 (s, 1H), 10.87 (s, 1H), 8.65 (d, J = 8.7 Hz, 1H), 8.30 (d, J = 7.4 Hz, 1H), 8.20 – 8.11 (m, 2H), 7.95 (dt, J = 7.4, 2.3 Hz, 4H), 7.70 (s, 1H), 7.64 – 7.53 (m, 2H), 7.52 – 7.43 ppm (m, 4H); ^{13}C NMR (125 MHz, 300 K, acetone-d_6): δ = 165.6, 157.0, 148.7, 145.2, 140.0, 136.2, 135.2, 134.7 (q, J = 33.1 Hz), 134.2 (2C), 131.6, 131.4, 130.6, 130.2, 129.7 (2C), 129.5, 128.5 (2C), 127.9, 125.0, 124.7, 124.2 (q, J = 271.2 Hz), 123.9, 121.0, 117.9, 110.3 ppm; note that the ^{13}C NMR signals at 165.6, 157.0 and 148.7 ppm were assigned using HMBC. ^{19}F NMR (470 MHz, 300 K, acetone-d_6): δ = -64.2 ppm (s, 3F); IR (film): ṽ = 3456, 3025, 2970, 2946, 1739, 1437, 1366, 1228, 1217 cm^{-1}; HRMS (ESI): m/z calculated for $C_{27}H_{18}ClF_3N_3O_3S_2$ [M+H]$^+$: 588.0425, found: 588.0442.

2-((4-Methylphenyl)sulfonamido)benzoic acid (21a)

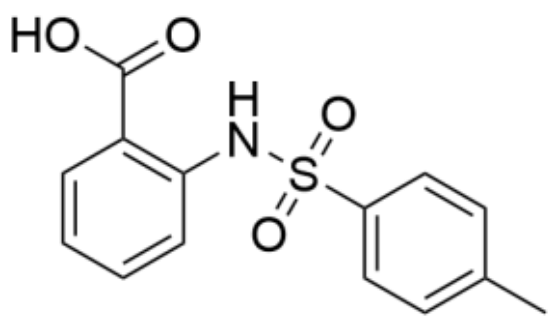

According to General Procedure A, sulfonamide **21a** (208 mg, 0.71 mmol, 71%) was obtained from commercially-sourced 2-aminobenzoic acid (137 mg, 1.0 mmol) and 4-methylbenzenesulfonyl chloride (191 mg, 1.0 mmol), followed by reverse phase column chromatography (12 g Sfär C18 Duo cartridge; 12 mL/min; 100%$_{v/v}$ (H_2O + 0.1%$_{v/v}$ FA) (2 CV), followed by a gradient (30 CV): 0%$_{v/v}$→100%$_{v/v}$ (acetonitrile + 0.1%$_{v/v}$ FA) in (H_2O + 0.1%$_{v/v}$ FA)). The analytical data of **21a** are in agreement with those reported.[46]

White solid; m.p.: > 225 °C (decomposition); ^{1}H NMR (600 MHz, 300 K, DMSO-d_6): δ = 13.96 (s, 1H), 11.14 (s, 1H), 7.92 – 7.87 (m, 1H), 7.72 – 7.67 (m, 2H), 7.56 – 7.50 (m, 2H), 7.38 – 7.33 (m, 2H), 7.11 (ddd, J = 8.0, 7.0, 1.5 Hz, 1H), 2.33 ppm (s, 3H); ^{13}C NMR (150 MHz, 300 K, DMSO-d_6): δ = 169.8, 144.1, 139.9, 135.7, 134.5, 131.5, 129.9 (2C), 126.9 (2C), 123.2, 118.2, 116.6, 20.9 ppm; IR (film): ṽ = 3042, 1672, 1492, 1258, 1089 cm^{-1}; HRMS (ESI): m/z calculated for $C_{14}H_{12}NO_4S$ [M-H]$^-$: 290.0493, found: 290.0488.

N-(4-(4-Chlorophenyl)thiazol-2-yl)-2-((4-methylphenyl)sulfonamido)benzamide (21)

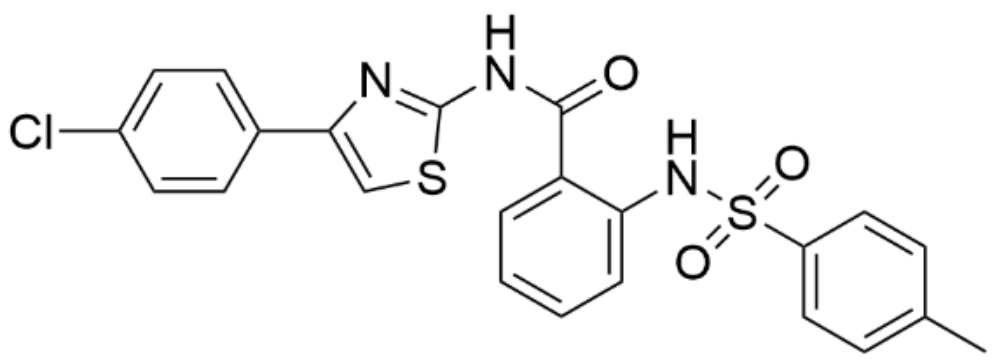

According to General Procedure B, amide **21** (61.4 mg, 0.13 mmol, 25%) was obtained from the carboxylic acid **21a** (146 mg, 0.50 mmol) and commercially-sourced 4-(4-chlorophenyl)thiazol-2-amine (105 mg, 0.50 mmol), followed by reverse phase column chromatography (12 g Sfär C18 Duo cartridge; 12 mL/min; 100%$_{v/v}$ (H_2O + 0.1%$_{v/v}$ FA) (2 CV), followed by a gradient (8 CV): 0%$_{v/v}$→40%$_{v/v}$ and (20 CV): 40%$_{v/v}$→100%$_{v/v}$ (acetonitrile + 0.1%$_{v/v}$ FA) in (H_2O + 0.1%$_{v/v}$ FA)).

White solid; m.p.: > 240 °C (decomposition); ^{1}H NMR (600 MHz, 300 K, CD_3CN): δ = 10.25 (s, 1H), 9.72 (s, 1H), 7.94 – 7.89 (m, 2H), 7.73 (dd, J = 8.0, 1.5 Hz, 1H), 7.64 – 7.55 (m, 2H), 7.52 – 7.48 (m, 2H), 7.49 – 7.43 (m, 3H), 7.29 (ddd, J = 8.0, 7.0, 1.5 Hz, 1H), 7.14 – 7.08 (m, 2H), 2.20 ppm (s, 3H); ^{13}C NMR (150 MHz, 300 K, CD_3CN): δ = 167.3, 158.5, 149.4, 145.5, 138.9, 136.3, 134.5, 134.2, 134.1, 130.7 (2C), 129.9, 129.8 (2C), 128.5

(2C), 128.0 (2C), 125.8, 124.5, 123.6, 110.2, 21.5 ppm; IR (film): ṽ = 3252, 3034, 2921, 2835, 1651, 1541, 1445, 1335, 1169, 1015 cm^{-1}; HRMS (ESI): m/z calculated for $C_{23}H_{19}ClN_3O_3S_2$ $[M+H]^+$: 484.0551, found: 484.0538.

***N*-(4-(4-chlorophenyl)thiazol-2-yl)-3-((4-methylphenyl)sulfonamido)isonicotinamide (22)**

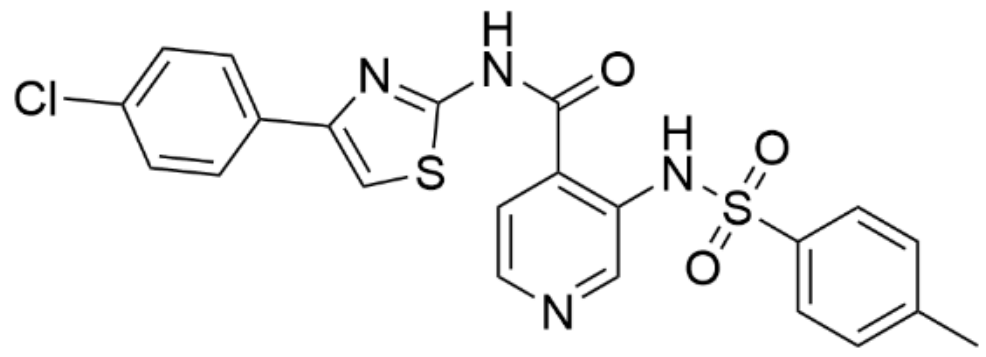

According to General Procedure B, amide **22** (52.2 mg, 0.12 mmol, 22%) was obtained from the reported carboxylic acid **22a** [41] (140 mg, 0.48 mmol) and commercially-sourced 4-(4-chlorophenyl)thiazol-2-amine (103 mg, 0.50 mmol), followed by reverse phase column chromatography (12 g Sfär C18 Duo cartridge; 12 mL/min; 100%$_{v/v}$ (H_2O + 0.1%$_{v/v}$ FA) (2 CV), followed by a gradient (8 CV): 0%$_{v/v}$ →40%$_{v/v}$ and (20 CV): 40%$_{v/v}$ →100%$_{v/v}$ (acetonitrile + 0.1%$_{v/v}$ FA) in (H_2O + 0.1%$_{v/v}$ FA)).

Yellow solid; m.p.: >230 (decomposition); ^{1}H NMR (600 MHz, 300 K, acetone-*d$_6$*): δ = 8.86 (s, 1H), 8.54 (d, *J* = 5.1 Hz, 1H), 8.00 – 7.93 (m, 2H), 7.87 (d, *J* = 5.0 Hz, 1H), 7.70 (s, 1H), 7.62 – 7.57 (m, 2H), 7.51 – 7.45 (m, 2H), 7.18 (d, *J* = 8.0 Hz, 2H), 2.20 ppm (s, 3H); ^{13}C NMR (150 MHz, 300 K, acetone-*d$_6$*): δ = 165.5, 158.6, 149.1, 146.9, 146.7, 145.2, 136.8, 134.1, 134.0, 133.9, 131.2, 130.7 (2C), 129.7 (2C), 128.4 (2C), 128.0 (2C), 122.6, 110.2, 21.4 ppm; IR (film): ṽ = 3120, 2960, 2854, 1730, 1602, 1539, 1501, 1403, 1316, 1205, 1194, 1080 cm^{-1}; HRMS (ESI): m/z calculated for $C_{22}H_{18}ClN_4O_3S_2$ $[M+H]^+$: 485.0503, found: 485.0503.

3-((4-Methylphenyl)sulfonamido)benzoic acid (23a)

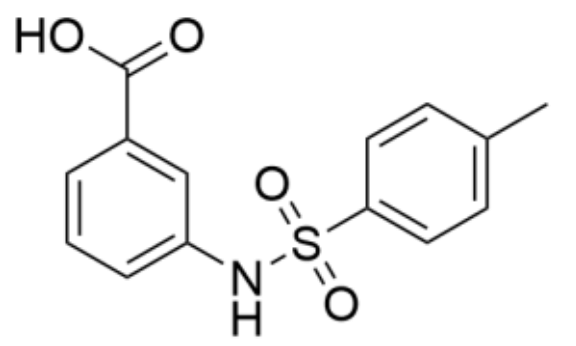

According to General Procedure A, sulfonamide **23a** (78.7 mg, 0.27 mmol, 54%) was obtained from commercially-sourced 3-aminobenzoic acid (68.6 mg, 0.50 mmol) and 4-methylbenzenesulfonyl chloride (95.3 mg, 0.50 mmol). The crude product was used in the following step without further purification. The analytical data of **23a** are in agreement with those reported.[46]

White solid; m.p.: 158–160 °C; ^{1}H NMR (600 MHz, 300 K, DMSO-*d$_6$*): δ = 13.01 (s, 1H), 10.43 (s, 1H), 7.68 (d, *J* = 2.0 Hz, 1H), 7.66 – 7.61 (m, 2H), 7.57 (dt, 1H), 7.38 – 7.30 (m, 4H), 2.32 ppm (s, 3H); ^{13}C NMR (150 MHz, 300 K, DMSO-*d$_6$*): δ = 166.7, 143.4, 138.1, 136.4, 131.8, 129.8 (2C), 129.4, 126.6 (2C), 124.7, 123.8, 120.3, 20.9 ppm; IR (film): ṽ = 3258, 3032, 2911, 1682, 1587, 1408, 1160, 1091 cm^{-1}; HRMS (ESI): m/z calculated for $C_{14}H_{12}NO_4S$ $[M-H]^-$: 290.0493, found: 290.0505.

***N*-(4-(4-Chlorophenyl)thiazol-2-yl)-3-((4-methylphenyl)sulfonamido)benzamide (23)**

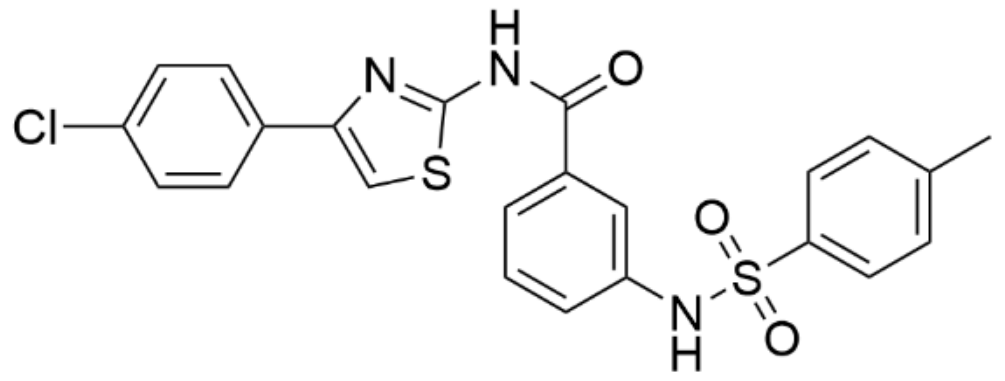

According to General Procedure B, amide **23** (17.3 mg, 0.035 mmol, 14%) was obtained from the carboxylic acid **23a** (72.8 mg, 0.25 mmol) and commercially-sourced 4-(4-chlorophenyl)thiazol-2-amine (52.7 mg, 0.25 mmol), followed by reverse phase column chromatography (12 g Sfär C18 Duo cartridge; 12 mL/min; 100%$_{v/v}$ (H_2O + 0.1%$_{v/v}$ FA) (2 CV), followed by a gradient (8 CV): 0%$_{v/v}$→40%$_{v/v}$ and (20 CV): 40%$_{v/v}$→100%$_{v/v}$ (acetonitrile + 0.1%$_{v/v}$ FA) in (H_2O + 0.1%$_{v/v}$ FA)).

Yellow solid; m.p.: >200 °C (decomposition); ^{1}H NMR (600 MHz, 300 K, acetone-*d$_6$*): δ = 11.53 (s, 1H), 9.22 (s, 1H), 8.00 (t, *J* = 1.9 Hz, 1H), 7.97 – 7.93 (m, 2H), 7.91 (dt, *J* = 7.6, 1.4 Hz, 1H), 7.76 – 7.71 (m, 2H), 7.59 (s, 1H), 7.53 – 7.42 (m, 4H), 7.37 – 7.31 (m, 2H), 2.36 ppm (s, 3H); ^{13}C NMR (150 MHz, 300 K, acetone-*d$_6$*): δ = 165.5, 159.3, 149.5, 144.7, 139.7, 139.6, 137.9, 134.5, 133.8, 130.6 (2C), 130.5, 129.6 (2C), 128.4 (2C), 128.0

(2C), 125.2, 124.3, 121.0, 109.5, 21.4 ppm; note that the ^{13}C NMR signals at 164.2 and 157.7 ppm were assigned using HMBC. IR (film): ṽ = 3346, 3258, 2955, 2956, 1658, 1536, 1502, 1397, 1288, 1162, 1091 cm^{-1}; HRMS (ESI): m/z calculated for $C_{23}H_{19}ClN_3O_3S_2$ $[M+H]^+$: 484.0551, found: 484.0536.

4-((4-Methylphenyl)sulfonamido)benzoic acid (24a)

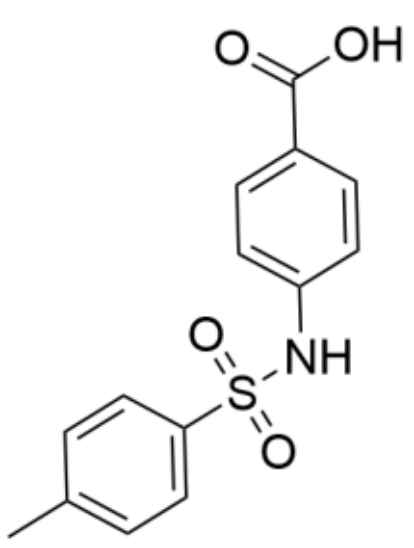

According to General Procedure A, sulfonamide **24a** (83.0 mg, 0.28 mmol, 57%) was obtained from commercially-sourced 4-aminobenzoic acid (68.6 mg, 0.50 mmol) and 4-methylbenzenesulfonyl chloride (95.3 mg, 0.50 mmol). The crude residue was used in the following step without further purification. The analytical data of **24a** are in agreement with those reported.[46]

Brown solid; m.p.: > 200 °C (decomposition); ^1H NMR (600 MHz, 300 K, DMSO-d_6): δ = 12.71 (s, 1H), 10.72 (s, 1H), 7.81 – 7.76 (m, 2H), 7.73 – 7.66 (m, 2H), 7.39 – 7.33 (m, 2H), 7.21 – 7.15 (m, 2H), 2.33 ppm (s, 3H); ^{13}C NMR (150 MHz, 300 K, DMSO-d_6): δ = 166.7, 143.6, 142.1, 136.4, 130.7 (2C), 129.8 (2C), 126.7 (2C), 125.5, 118.0 (2C), 20.9 ppm; IR (film): ṽ = 3246, 2974, 1682, 1589, 1512, 1429, 1315, 1159, 1088 cm^{-1}; HRMS (ESI): m/z calculated for $C_{14}H_{12}NO_4S$ $[M-H]^-$: 290.0493, found: 290.0494.

***N*-(4-(4-Chlorophenyl)thiazol-2-yl)-4-((4-methylphenyl)sulfonamido)benzamide (24)**

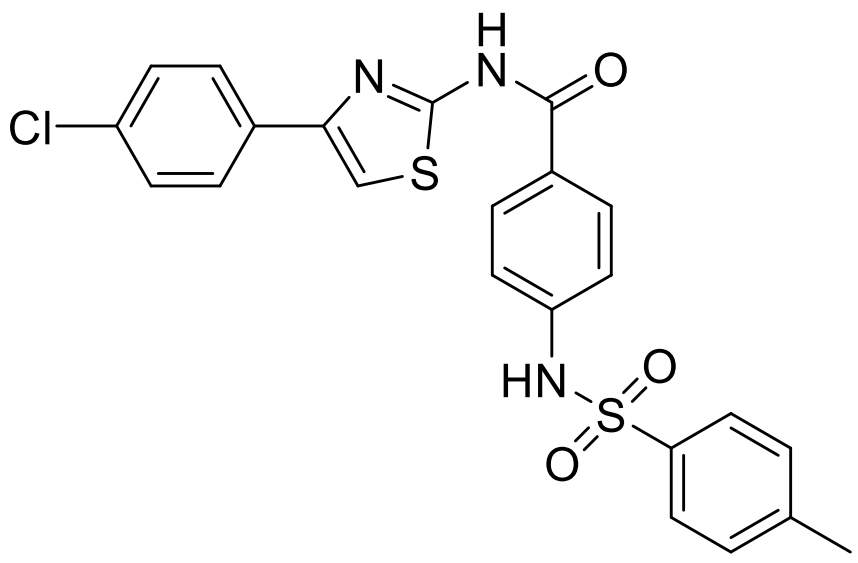

According to General Procedure B, amide **24** (38.9 mg, 0.080 mmol, 38%) was obtained from the carboxylic acid **24a** (62.4 mg, 0.21 mmol) and commercially-sourced 4-(4-chlorophenyl)thiazol-2-amine (44.2 mg, 0.21 mmol), followed by reverse phase column chromatography (12 g Sfär C18 Duo cartridge; 12 mL/min; $100\%_{v/v}$ (H_2O + $0.1\%_{v/v}$ FA) (2 CV), followed by a gradient (8 CV): $0\%_{v/v}\rightarrow40\%_{v/v}$ and (20 CV): $40\%_{v/v}\rightarrow100\%_{v/v}$ (acetonitrile + $0.1\%_{v/v}$ FA) in (H_2O + $0.1\%_{v/v}$ FA)).

White solid; m.p.: 138–140 °C; ^1H NMR (600 MHz, 300 K, CD_3CN): δ = 7.91 – 7.86 (m, 4H), 7.77 – 7.73 (m, 2H), 7.45 – 7.42 (m, 2H), 7.38 (s, 1H), 7.36 – 7.33 (m, 2H), 7.29 – 7.24 (m, 2H), 2.37 ppm (s, 3H); ^{13}C NMR (150 MHz, 300 K, CD_3CN): δ = 165.3, 159.4, 149.4, 145.6, 142.7, 137.2, 134.3, 134.0, 130.8 (2C), 130.3 (2C), 129.7 (2C), 128.5, 128.4 (2C), 128.1 (2C), 119.6 (2C), 109.6, 21.5 ppm; IR (film): ṽ = 3449, 2970, 2954, 1653, 1607, 1580, 1551, 1304, 1157, 1091 cm^{-1}; HRMS (ESI): m/z calculated for $C_{23}H_{19}ClN_3O_3S_2$ $[M+H]^+$: 484.0551, found: 484.0538.

2-((4-Methylphenyl)sulfonamido)-5-(trifluoromethyl)benzoic acid (25a)

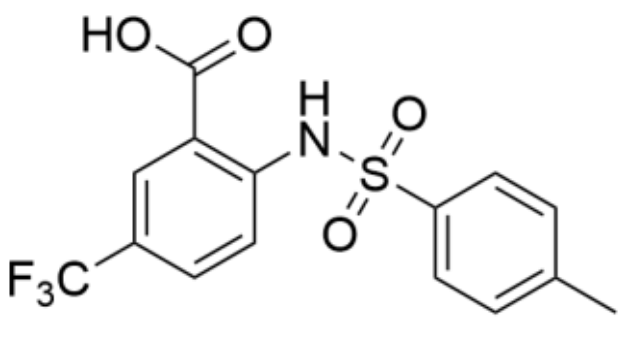

According to General Procedure A, sulfonamide **25a** (493 mg, 1.4 mmol, 69%) was obtained from commercially-sourced 2-amino-5-(trifluoromethyl)benzoic acid (408 mg, 2.0 mmol) and 4-methylbenzenesulfonyl chloride (382 mg, 2.0 mmol). The crude residue was used in the following step without further purification. The analytical data of **25a** are in agreement with those reported.[47]

Yellow amorphous solid; ^1H NMR (400 MHz, 300 K, MeOD-d_4): δ = 8.20 (d, J = 2.2 Hz, 1H), 7.81 (d, J = 8.8 Hz, 1H), 7.77 – 7.69 (m, 3H), 7.31 (d, J = 8.1 Hz, 2H), 2.34 ppm (s, 3H); ^{13}C NMR (100 MHz, 300 K, MeOD-d_4): δ = 170.2, 146.2, 145.0, 137.2, 131.8 (q, J = 3.5 Hz), 131.0 (2C), 129.8, 128.4 (2C), 125.7 (q, J = 33.5 Hz), 122.9 (q, J = 271.2 Hz), 117.6, 119.8, 21.4 ppm; ^{19}F NMR (375 MHz, 300 K, MeOD-d_4): δ = -64.7 ppm (s, 3F); IR (film): ṽ = 3016, 2970, 2945, 1738, 1452, 1366, 1271 cm^{-1}; HRMS (ESI): m/z calculated for $C_{15}H_{11}F_3NO_4S$

[M-H]$^-$: 358.0366, found: 358.0375.

N-(4-(4-Chlorophenyl)thiazol-2-yl)-2-((4-methylphenyl)sulfonamido)-5-(trifluoromethyl)benzamide (25)

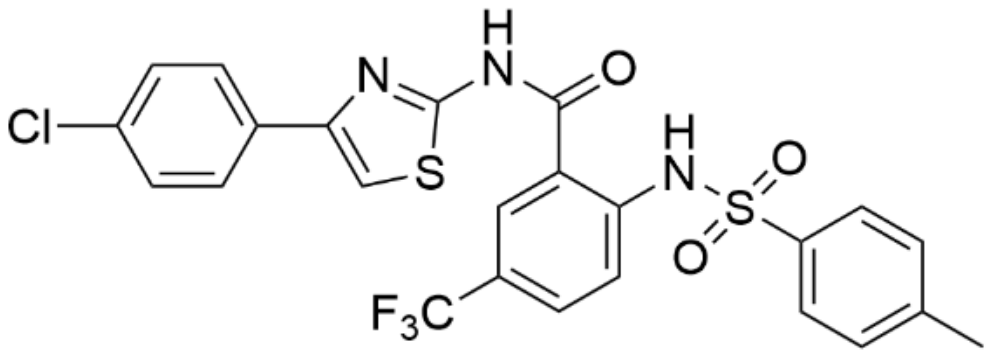

According to General Procedure B, amide **25** (59.6 mg, 0.12 mmol, 54%) was obtained from the carboxylic acid **25a** (74.7 mg, 0.20 mmol) and commercially-sourced 4-(4-chlorophenyl)thiazol-2-amine (42.2 mg, 0.20 mmol), followed by reverse phase column chromatography (12 g Sfär C18 Duo cartridge; 12 mL/min; 100%$_{v/v}$ (H_2O + 0.1%$_{v/v}$ FA) (2 CV), followed by a gradient (8 CV): 0%$_{v/v}$→40%$_{v/v}$ and (20 CV): 40%$_{v/v}$→100%$_{v/v}$ (acetonitrile + 0.1%$_{v/v}$ FA) in (H_2O + 0.1%$_{v/v}$ FA)).

Pale yellow solid; m.p.: >230 °C (decomposition); ^{1}H NMR (600 MHz, 300 K, acetone-d_6): δ = 11.91 (s, 1H), 10.70 (s, 1H), 8.43 (d, J = 1.9 Hz, 1H), 7.99 – 7.88 (m, 4H), 7.79 – 7.74 (m, 2H), 7.70 (s, 1H), 7.50 – 7.45 (m, 2H), 7.31 – 7.27 (m, 2H), 2.29 ppm (s, 3H); ^{13}C NMR (150 MHz, 300 K, acetone-d_6): δ = 167.1, 158.0, 148.2, 145.5, 143.2, 137.1, 134.2, 133.9, 131.0, 130.8 (2C), 129.7 (2C), 128.4 (2C), 128.1 (2C), 127.7, 125.5, 124.8 (q, J = 271.3 Hz), 121.9, 121.4, 110.2, 21.4 ppm; note that the ^{13}C NMR signals at 158.0 and 148.2 ppm were assigned from HMBC. ^{19}F NMR (565 MHz, 300 K, acetone-d_6): δ = -62.8 ppm (s, 3F); IR (film): ṽ = 3629, 1650, 1478, 1317, 1213, 1166, 1090 cm^{-1}; HRMS (ESI): m/z calculated for $C_{24}H_{18}ClF_3N_3O_3S_2$ [M+H]$^+$: 552.0425, found: 552.0432.

2-((4-Methylphenyl)sulfonamido)-6-(trifluoromethyl)benzoic acid (26a)

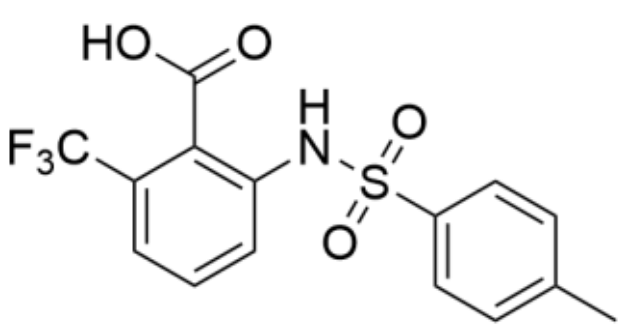

According to General Procedure A, sulfonamide **26a** (359 mg, 1.0 mmol, 50%) was obtained from commercially-sourced 2-amino-6-(trifluoromethyl)benzoic acid (410 mg, 2.0 mmol) and 4-methylbenzenesulfonyl chloride (382 mg, 2.0 mmol) followed by reverse phase column chromatography (12 g Sfär C18 Duo cartridge; 12 mL/min; 100%$_{v/v}$ (H_2O + 0.1%$_{v/v}$ FA) (2 CV), followed by a gradient (30 CV): 0%$_{v/v}$→100%$_{v/v}$ (acetonitrile + 0.1%$_{v/v}$ FA) in (H_2O + 0.1%$_{v/v}$ FA)). The analytical data of **26a** are in agreement with those reported.[47]

White solid; m.p.: 194–196 °C; ^{1}H NMR (400 MHz, 300 K, MeOD-d_4): δ = 7.69 (dd, J = 5.5, 3.9 Hz, 1H), 7.65 – 7.59 (m, 2H), 7.58 – 7.49 (m, 2H), 7.33 – 7.25 (m, 2H), 2.37 ppm (s, 3H); ^{13}C NMR (100 MHz, 300 K, MeOD-d_4): δ = 168.7, 145.62, 137.8, 137.0, 131.8, 130.8 (2C), 130.1 (q, J = 33.5 Hz), 129.5, 128.3 (2C), 127.9, 124.6 (q, J = 271.2 Hz), 124.6, 21.5 ppm; ^{19}F NMR (375 MHz, 300 K, MeOD-d_4): δ = -61.3 ppm (s, 3F); IR (film): ṽ = 3456, 3015, 2970, 2947, 1739, 1425, 1366, 1228, 1217 cm^{-1}; HRMS (ESI): m/z calculated for $C_{15}H_{11}F_3NO_4S$ [M-H]$^-$: 358.0366, found: 358.0357.

N-(4-(4-Chlorophenyl)thiazol-2-yl)-2-((4-methylphenyl)sulfonamido)-6-(trifluoromethyl)benzamide (26)

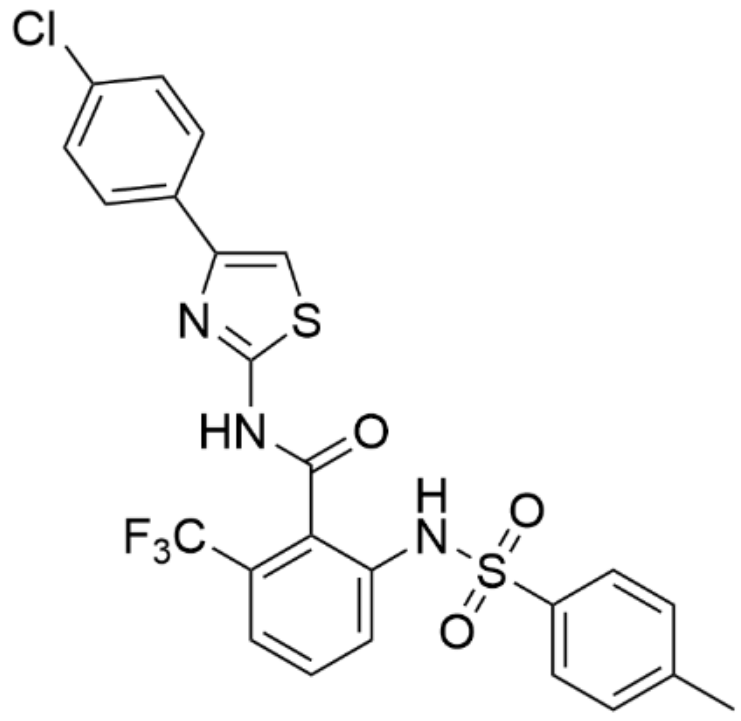

According to General Procedure B, amide **26** (7.10 mg, 0.013 mmol, 4%) was obtained from the carboxylic acid **26a** (108 mg, 0.30 mmol) and commercially-sourced 4-(4-chlorophenyl)thiazol-2-amine (63.2 mg, 0.30 mmol) (Propylphosphonic anhydride (T3P) was used as a coupling reagent instead of HATU), followed by reverse phase column chromatography (12 g Sfär C18 Duo cartridge; 12 mL/min; 100%$_{v/v}$ (H_2O + 0.1%$_{v/v}$ FA) (2 CV), followed by a gradient (8 CV): 0%$_{v/v}$→40%$_{v/v}$ and (20 CV): 40%$_{v/v}$→100%$_{v/v}$ (acetonitrile + 0.1%$_{v/v}$ FA) in (H_2O + 0.1%$_{v/v}$ FA)).

Yellow solid; m.p.: 102–104 °C; ^{1}H NMR (600 MHz, 300 K, CD_3CN): δ = 8.14 (dd, *J* = 7.9, 1.4 Hz, 1H), 8.01 (m, 2H), 7.91 (m, 2H), 7.52 (m, 2H), 7.44 (s, 1H), 7.13 – 7.08 (m, 2H), 6.93 – 6.85 (m, 2H), 2.21 ppm (s, 3H); ^{13}C NMR (150 MHz, 300 K, CD_3CN): δ = 168.8, 158.2, 157.4, 148.5, 146.7, 138.7, 134.6, 134.4, 134.0, 132.2, 130.5 (2C), 129.9 (2C), 128.6 (2C), 127.8, 127.4 (2C), 125.0 (q, *J* = 7.9 Hz), 123.4, 123.2, 111.3, 40.6 ppm; ^{19}F NMR (565 MHz, 300 K, CD_3CN): δ = -59.7 ppm (s, 3F); IR (film): ṽ = 3285, 2924, 1403, 1321, 1113, 1078 cm^{-1}; HRMS (ESI): m/z calculated for $C_{24}H_{18}ClF_3N_3O_3S_2$ $[M+H]^+$: 552.0425, found: 552.0425.

4-(*tert*-Butyl)-2-((4-methylphenyl)sulfonamido)benzoic acid (27a)

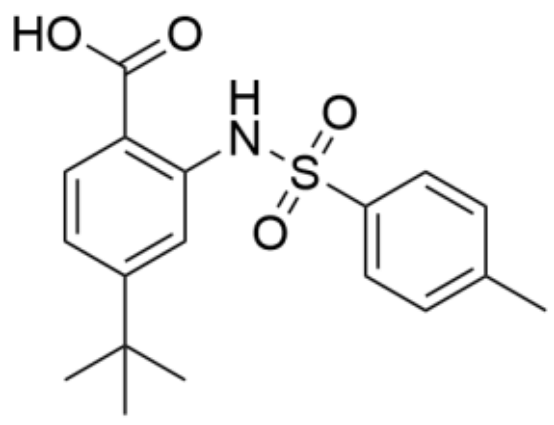

According to General Procedure A, sulfonamide **27a** (211 mg, 0.61 mmol, 61%) was obtained from commercially-sourced 2-amino-4-(*tert*-butyl)benzoic acid (193 mg, 1.0 mmol) and 4-methylbenzenesulfonyl chloride (191 mg, 1.0 mmol), followed by reverse phase column chromatography (12 g Sfär C18 Duo cartridge; 12 mL/min; 100%$_{v/v}$ (H_2O + 0.1%$_{v/v}$ FA) (2 CV), followed by a gradient (30 CV): 0%$_{v/v}$→100%$_{v/v}$ (acetonitrile + 0.1%$_{v/v}$ FA) in (H_2O + 0.1%$_{v/v}$ FA)).

White solid; m.p.: 183–185 °C; ^{1}H NMR (600 MHz, 300 K, MeOD-*d$_4$*): δ = 7.83 (d, *J* = 8.3 Hz, 1H), 7.70 – 7.62 (m, 3H), 7.28 – 7.22 (m, 2H), 7.06 (dd, *J* = 8.3, 1.9 Hz, 1H), 2.32 (s, 3H), 1.26 ppm (s, 9H); ^{13}C NMR (150 MHz, 300 K, MeOD-*d$_4$*): δ = 172.2, 158.4, 145.4, 141.4, 137.5, 132.5, 130.6 (2C), 128.5 (2C), 121.1, 117.5, 117.1, 36.0, 31.3, 21.4 (3C) ppm; IR (film): ṽ = 3691, 2963, 2360, 1802, 1665, 1391, 1161 cm^{-1}; HRMS (ESI): m/z calculated for $C_{18}H_{20}NO_4S$ $[M-H]^-$: 346.1119, found: 346.1110.

4-(*tert*-Butyl)-N-(4-(4-chlorophenyl)thiazol-2-yl)-2-((4-methylphenyl)sulfonamido)benzamide (27)

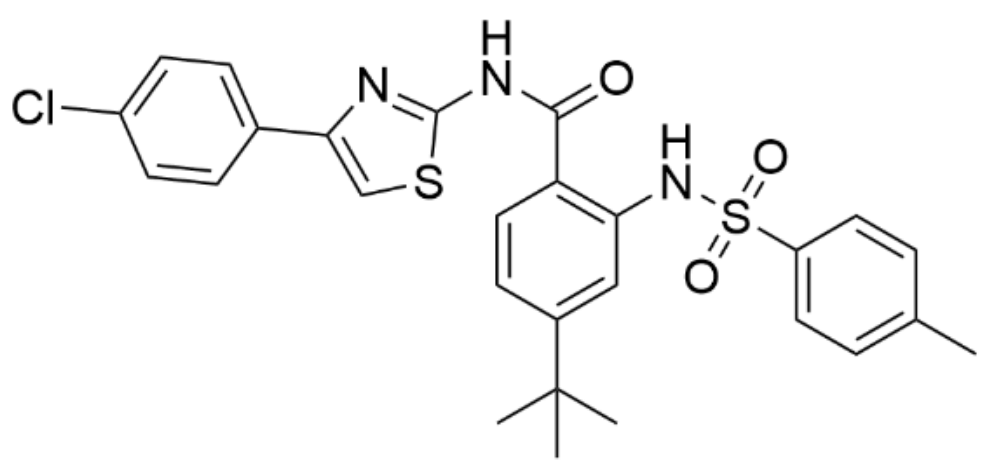

According to General Procedure B, amide **27** (32.8 mg, 0.061 mmol, 20%) was obtained from the carboxylic acid **27a** (104 mg, 0.30 mmol) and commercially-sourced 4-(4-chlorophenyl)thiazol-2-amine (63.2 mg, 0.30 mmol), followed by reverse phase column chromatography (12 g Sfär C18 Duo cartridge; 12 mL/min; 100%$_{v/v}$ (H_2O + 0.1%$_{v/v}$ FA) (2 CV), followed by a gradient (8 CV): 0%$_{v/v}$→40%$_{v/v}$ and (20 CV): 40%$_{v/v}$→100%$_{v/v}$ (acetonitrile + 0.1%$_{v/v}$ FA) in (H_2O + 0.1%$_{v/v}$ FA)).

White solid; m.p.: 126–128 °C; ^{1}H NMR (600 MHz, 300 K, CD_3CN): δ = 10.32 (s, 1H), 9.96 (s, 1H), 7.94 – 7.89 (m, 2H), 7.71 (d, *J* = 8.3 Hz, 1H), 7.60 – 7.54 (m, 3H), 7.49 – 7.43 (m, 3H), 7.30 (dd, *J* = 8.3, 1.9 Hz, 1H), 7.19 – 7.14 (m, 2H), 2.24 (s, 3H), 1.29 ppm (s, 9H); ^{13}C NMR (150 MHz, 300 K, CD_3CN): δ = 167.2, 158.6, 158.6,

149.5, 145.6, 139.2, 136.4, 134.1 (2C), 130.7 (2C), 129.8 (2C), 129.8, 128.4 (2C), 128.2 (2C), 122.6, 120.7, 119.9, 110.1, 35.9, 31.0 (3C), 21.5 ppm; IR (film): ṽ = 2967, 1701, 1644, 1305, 1251, 1120 cm^{-1}; HRMS (ESI): m/z calculated for $C_{27}H_{27}ClN_3O_3S_2$ $[M+H]^+$: 540.1177, found: 540.1181.

3-((4-Methylphenyl)sulfonamido)-[1,1'-biphenyl]-4-carboxylic acid (28a)

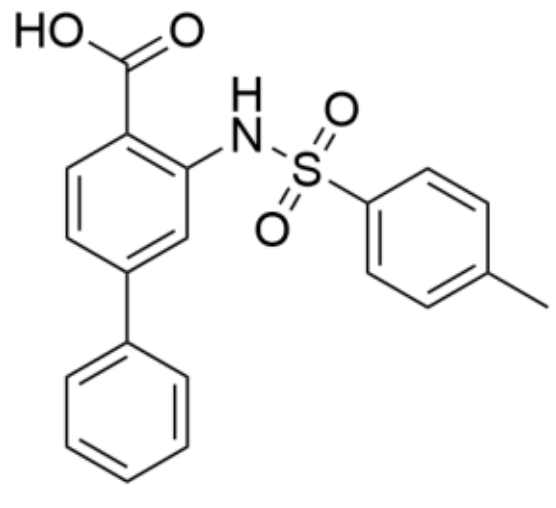

According to General Procedure A, sulfonamide **28a** (232 mg, 0.63 mmol, 63%) was obtained from commercially-sourced 3-amino-[1,1'-biphenyl]-4-carboxylic acid (213 mg, 1.0 mmol) and 4-methylbenzenesulfonyl chloride (191 mg, 1.0 mmol). The crude residue was used in the following step without further purification.

Brown solid; m.p.: >200 °C (decomposition); ^{1}H NMR (400 MHz, 300 K, MeOD-*d₄*): δ = 7.98 (d, *J* = 8.2 Hz, 1H), 7.85 (d, *J* = 1.8 Hz, 1H), 7.75 – 7.67 (m, 2H), 7.59 – 7.51 (m, 2H), 7.49 – 7.42 (m, 2H), 7.41 – 7.35 (m, 1H), 7.27 (m, 3H), 2.31 ppm (s, 3H); ^{13}C NMR (100 MHz, 300 K, MeOD-d_4): δ = 172.1, 147.3, 145.6, 142.0, 140.8, 137.5, 133.3, 130.7 (2C), 130.1 (2C), 129.5, 128.4 (2C), 128.0 (2C), 122.6, 118.9, 118.2, 21.4 ppm; IR (film): ṽ = 3610, 1667, 1613, 1483, 1342, 1185, 1161 cm^{-1}; HRMS (ESI): m/z calculated for $C_{20}H_{16}NO_4S$ $[M-H]^-$: 366.0806, found: 366.0790.

***N*-(4-(4-Chlorophenyl)thiazol-2-yl)-3-((4-methylphenyl)sulfonamido)-[1,1'-biphenyl]-4-carboxamide (28)**

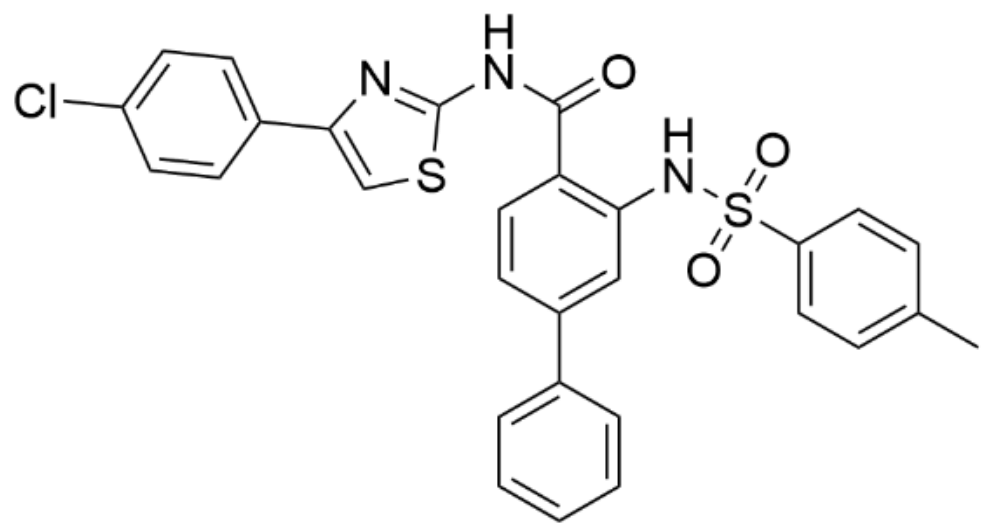

According to General Procedure B, amide **28** (15.2 mg, 0.027 mmol, 9%) was obtained from the carboxylic acid **28a** (110 mg, 0.30 mmol) and commercially-sourced 4-(4-chlorophenyl)thiazol-2-amine (63.2 mg, 0.30 mmol), followed by reverse phase column chromatography (12 g Sfär C18 Duo cartridge; 12 mL/min; 100%$_{v/v}$ (H_2O + 0.1%$_{v/v}$ FA) (2 CV), followed by a gradient (8 CV): 0%$_{v/v}$→40%$_{v/v}$ and (20 CV): 40%$_{v/v}$→100%$_{v/v}$ (acetonitrile + 0.1%$_{v/v}$ FA) in (H_2O + 0.1%$_{v/v}$ FA)).

Yellow amorphous solid; ^{1}H NMR (600 MHz, 300 K, DMSO-d_6): δ = 12.71 (s, 1H), 10.35 (s, 1H), 8.01 – 7.95 (m, 3H), 7.83 (s, 1H), 7.64 (d, *J* = 7.9 Hz, 2H), 7.61 – 7.49 (m, 8H), 7.45 (t, *J* = 7.4 Hz, 1H, H-31), 7.27 (d, *J* = 8.1 Hz, 2H), 2.25 ppm (s, 3H); ^{13}C NMR (150 MHz, 300 K, DMSO-d_6): δ = 165.9, 158.0, 147.9, 144.2, 143.9, 138.2, 137.5, 135.7, 133.1, 132.3, 130.4, 129.8 (2C), 129.2 (2C), 128.8 (2C), 128.7, 127.5 (2C), 126.9 (2C), 126.7 (2C), 122.7, 122.3, 120.3, 109.7, 20.9 ppm; IR (film): ṽ = 2926, 2854, 1773, 1643, 1544, 1401, 1292, 1244, 1164, 1014 cm^{-1}; HRMS (ESI): m/z calculated for $C_{29}H_{22}ClN_3O_3S_2K$ $[M+K]^+$: 598.0423, found: 598.0428.

***N*-(4-(4-Methoxyphenyl)thiazol-2-yl)-2-((4-methylphenyl)sulfonamido)-5-(trifluoromethyl)benzamide (29)**

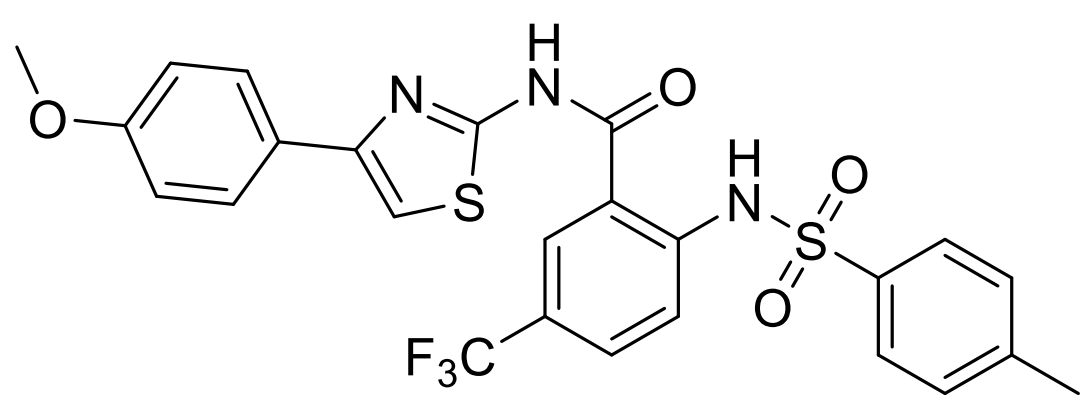

According to General Procedure B, amide **29** (75.9 mg, 0.14 mmol, 29%) was obtained from the carboxylic acid **25a** (170 mg, 0.47 mmol) and commercially-sourced and 4-(4-methoxyphenyl)thiazol-2-amine (96.9 mg, 0.75 mmol), followed by reverse phase column chromatography (12 g Sfär C18 Duo cartridge; 12 mL/min; 100%$_{v/v}$ (H_2O + 0.1%$_{v/v}$ FA) (2 CV), followed by a gradient (8 CV): 0%$_{v/v}$→40%$_{v/v}$ and (20 CV): 40%$_{v/v}$→100%$_{v/v}$ (acetonitrile + 0.1%$_{v/v}$ FA) in (H_2O + 0.1%$_{v/v}$ FA)) and subsequent purification using HPLC (phenyl-hexyl HPLC column (250 mm length, 19 mm internal diameter, 5 µm particle size); 10 mL/min, 25%$_{v/v}$→80%$_{v/v}$ (100 min) acetonitrile in (H_2O

+ 0.1%$_{v/v}$ FA)).

Yellow solid; m.p.: 120–122 °C; ^{1}H NMR (600 MHz, 300 K, acetone-d_6): δ = 8.45 (q, J = 1.1 Hz, 1H), 7.92 – 7.84 (m, 4H), 7.79 – 7.74 (m, 2H), 7.44 (s, 1H), 7.32 – 7.26 (m, 2H), 7.04 – 6.98 (m, 2H), 3.85 (s, 3H), 2.30 ppm (s, 3H); ^{13}C NMR (150 MHz, 300 K, acetone-d_6): δ = 165.7, 160.9, 158.3, 147.0, 145.4, 141.5, 137.2, 130.8 (3C), 128.2 (2C), 128.1 (2C), 127.9, 127.3, 125.2, 124.9 (q, J = 271.1 Hz), 121.6 (2C), 115.0 (2C), 107.4, 55.7, 21.4 ppm; ^{19}F NMR (565 MHz, 300 K, acetone-d_6): δ = -63.7 ppm (s, 3F); IR (film): ṽ = 3649, 2993, 1649, 1549, 1520, 1433, 1318, 1287, 1251, 1159, 1122, 1089, 1030 cm^{-1}; HRMS (ESI): m/z calculated for $C_{25}H_{21}F_3N_3O_4S_2$ $[M+H]^+$: 548.0920, found: 548.0908.

2-((4-Methylphenyl)sulfonamido)-*N*-(4-(naphthalen-1-yl)thiazol-2-yl)-5-(trifluoromethyl)benzamide (30)

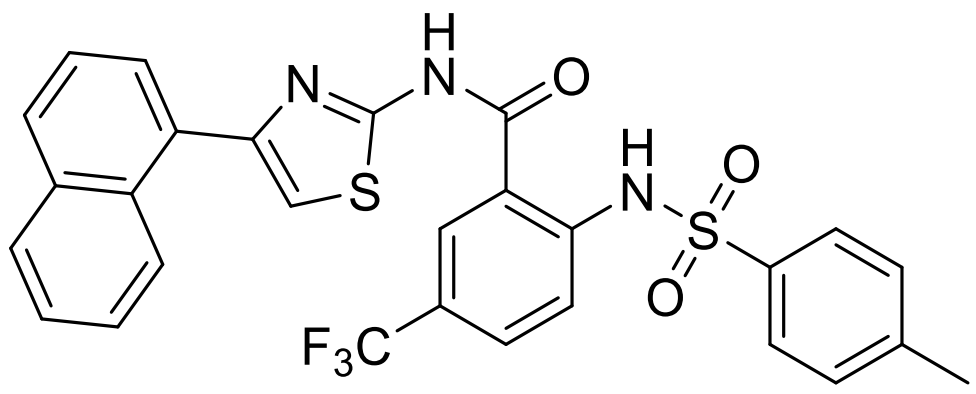

According to General Procedure B, amide **30** (49.3 mg, 0.087 mmol, 18%) was obtained from the carboxylic acid **25a** (170 mg, 0.47 mmol) and commercially-sourced 4-(naphthalen-1-yl)thiazol-2-amine (106 mg, 0.75 mmol), followed by reverse phase column chromatography (12 g Sfär C18 Duo cartridge; 12 mL/min; 100%$_{v/v}$ (H_2O + 0.1%$_{v/v}$ FA) (2 CV), followed by a gradient (8 CV): 0%$_{v/v}$→40%$_{v/v}$ and (20 CV): 40%$_{v/v}$→100%$_{v/v}$ (acetonitrile + 0.1%$_{v/v}$ FA) in (H_2O + 0.1%$_{v/v}$ FA)) and subsequent purification using flash column chromatography (10 g Sfär Silica Duo cartridge; 40 mL/min; 100%$_{v/v}$ cyclohexane (2 CV), followed by a linear gradient (25 CV): 0%$_{v/v}$→50%$_{v/v}$ ethyl acetate in cyclohexane).

Yellow solid; m.p.: 146–148 °C; ^{1}H NMR (600 MHz, 300 K, MeOD-d_4): δ = 8.24 (d, J = 2.1 Hz, 1H), 8.23 – 8.17 (m, 1H), 7.95 – 7.89 (m, 2H), 7.77 – 7.70 (m, 2H), 7.69 – 7.63 (m, 3H,), 7.55 – 7.47 (m, 3H), 7.28 (s, 1H), 7.24 – 7.19 (m, 2H), 2.27 ppm (s, 3H); ^{13}C NMR (150 MHz, 300 K, MeOD-d_4): δ = 169.1, 160.4, 145.9, 144.1, 143.2, 137.3, 135.4, 132.7, 132.3, 130.9 (2C), 130.5 (q, J = 3.5 Hz), 130.4, 129.5, 128.7, 128.3 (2C), 128.1, 127.6, 127.2, 126.8, 126.6, 126.3, 125.1 (q, J = 271.3 Hz), 123.9, 123.1, 113.0, 21.5 ppm; note that the ^{13}C NMR signal at 160.4 ppm was assigned using HMBC. ^{19}F NMR (565 MHz, 300 K, MeOD-d_4): δ = -64.8 ppm (s, 3F); IR (film): ṽ = 3034, 2929, 1925, 1707, 1592, 1546, 1401, 1344, 1315, 1162, 1122, 1089 cm^{-1}; HRMS (ESI): m/z calculated for $C_{28}H_{21}F_3N_3O_3S_2$ $[M+H]^+$: 568.0971, found: 568.0973.

2-((4-Methoxyphenyl)sulfonamido)-5-(trifluoromethyl)benzoic acid (31a)

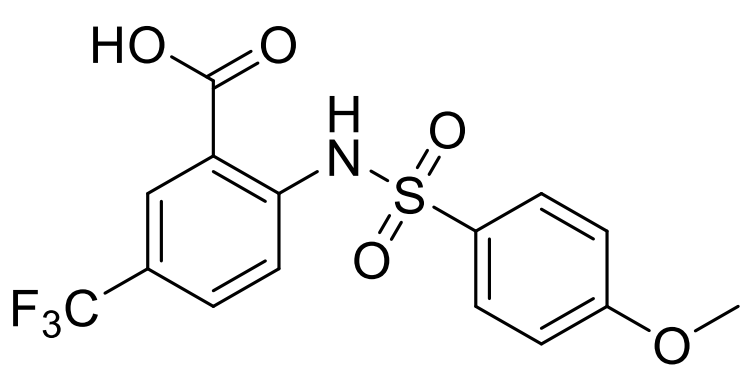

According to General Procedure A, sulfonamide **31a** (647 mg, 1.7 mmol, 57%) was obtained from commercially-sourced 2-amino-5-(trifluoromethyl)benzoic acid (615 mg, 3.0 mmol) and 4-methoxybenzenesulfonyl chloride (1.23 g, 6.0 mmol), followed by reverse phase column chromatography (12 g Sfär C18 Duo cartridge; 12 mL/min; 100%$_{v/v}$ (H_2O + 0.1%$_{v/v}$ FA) (2 CV), followed by a gradient (30 CV): 0%$_{v/v}$→100%$_{v/v}$ (acetonitrile + 0.1%$_{v/v}$ FA) in (H_2O + 0.1%$_{v/v}$ FA)).

Yellow solid; m.p.: 178–180 °C; ^{1}H NMR (400 MHz, 300 K, MeOD-d_4): δ = 8.20 (d, J = 2.2 Hz, 1H), 7.83 – 7.77 (m, 3H), 7.73 (dd, J = 8.8, 2.2 Hz, 1H), 7.02 – 6.96 (m, 2H), 3.80 ppm (s, 3H); ^{13}C NMR (100 MHz, 300 K, MeOD-d_4): δ = 170.2, 165.2, 145.1, 131.8 (q, J = 3.6 Hz), 131.4, 130.6 (2C) 129.8 (q, J = 4.0 Hz), 125.5 (q, J = 33.4 Hz), 125.0 (q, J = 272.0 Hz) , 119.8, 117.4, 115.5 (2C), 56.2 ppm; ^{19}F NMR (375 MHz, 300 K, MeOD-d_4): δ = -64.7 ppm (s, 3F); IR (film): ṽ = 3748, 3090, 2950, 1676, 1595, 1498, 1436, 1343, 1269, 1180, 1161, 1152, 1090 cm^{-1}; HRMS (ESI): m/z calculated for $C_{15}H_{11}F_3NO_5S$ $[M-H]^-$: 374.0315, found: 374.0315.

2-((4-Methoxyphenyl)sulfonamido)-*N*-(4-(naphthalen-1-yl)thiazol-2-yl)-5-(trifluoromethyl)benzamide (31)

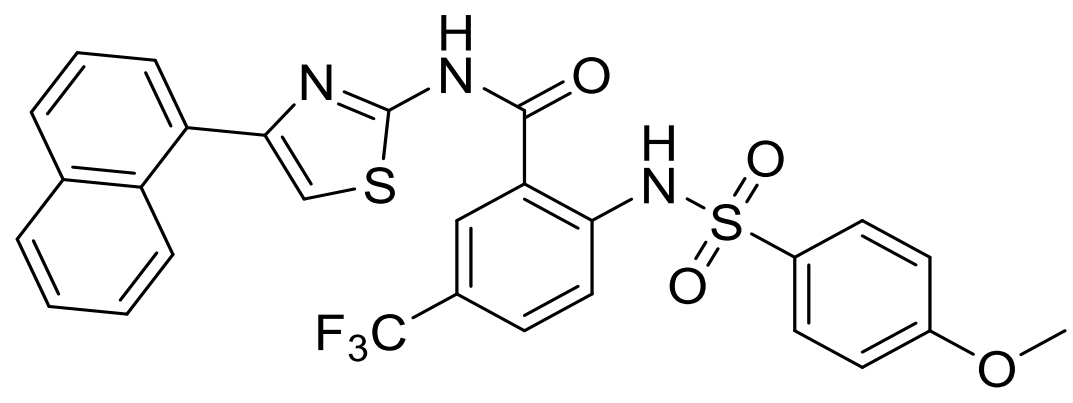

According to General Procedure B, amide **31** (60.4 mg, 0.10 mmol, 14%) was obtained from the carboxylic acid **31a** (281 mg, 0.75 mmol) and commercially-sourced 4-(naphthalen-1-yl)thiazol-2-amine (106 mg, 0.75 mmol), followed by reverse phase column chromatography (12 g Sfär C18 Duo cartridge; 12 mL/min; 100%$_{v/v}$ (H_2O + 0.1%$_{v/v}$ FA) (2 CV), followed by a gradient (8 CV): 0%$_{v/v}$→40%$_{v/v}$ and (20 CV): 40%$_{v/v}$→100%$_{v/v}$ (acetonitrile + 0.1%$_{v/v}$ FA) in (H_2O + 0.1%$_{v/v}$ FA)) and subsequent purification using HPLC (phenyl-hexyl HPLC column (250 mm length, 19 mm internal diameter, 5 µm particle size); 10 mL/min, 25%$_{v/v}$→80%$_{v/v}$ (100 min) acetonitrile in (H_2O + 0.1%$_{v/v}$ FA)).

White solid; m.p.: 135–137 °C; ^{1}H NMR (600 MHz, 300 K, acetone-d_6): δ = 8.48 (d, *J* = 2.1 Hz, 1H), 8.35 – 8.31 (m, 1H), 8.01 – 7.96 (m, 2H), 7.91 – 7.83 (m, 4H), 7.75 (dd, *J* = 7.1, 1.2 Hz, 1H), 7.60 – 7.51 (m, 3H), 7.45 (s, 1H), 7.04 – 6.99 (m, 2H), 3.79 ppm (s, 3H); ^{13}C NMR (150 MHz, 300 K, acetone-d_6): δ = 168.2, 164.4, 160.9, 147.7, 143.9, 134.9, 132.6, 132.2, 131.7, 130.7, 130.4 (2C), 129.9, 129.3, 128.5, 128.0, 127.3, 126.9, 126.6, 126.2, 125.1 (q, *J* = 33.4 Hz), 124.9 (q, *J* = 270.0 Hz), 121.5, 121.2, 115.4 (2C), 113.2, 56.1 ppm; ^{19}F NMR (565 MHz, 300 K, acetone-d_6): δ = -62.7 ppm (s, 3F); IR (film): ṽ = 3021, 2941, 2841, 1651, 1595, 1548, 1498, 1314, 1289, 1157, 1123, 1090, 1025 cm^{-1}; HRMS (ESI): m/z calculated for $C_{28}H_{21}F_3N_3O_4S_2$ [M+H]$^+$: 584.0920, found: 584.0932.

***N*-(4-(Naphthalen-1-yl)thiazol-2-yl)-2-(naphthalene-1-sulfonamido)-4-(trifluoromethyl)benzamide (32)**

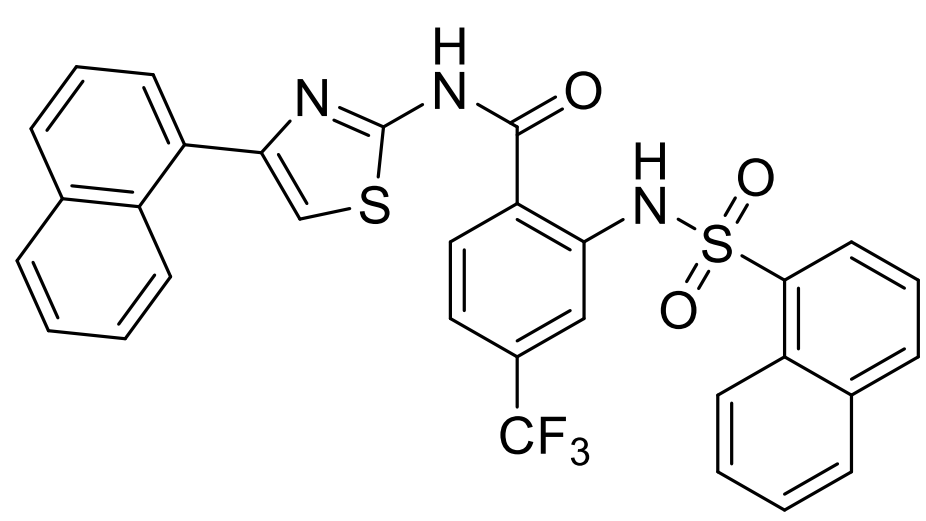

According to General Procedure B, amide **32** (122 mg, 0.20 mmol, 27%) was obtained from the carboxylic acid **20a** (297 mg, 0.75 mmol) and commercially-sourced 4-(naphthalen-1-yl)thiazol-2-amine (170 mg, 0.75 mmol), followed by reverse phase column chromatography (12 g Sfär C18 Duo cartridge; 12 mL/min; 100%$_{v/v}$ (H_2O + 0.1%$_{v/v}$ FA) (2 CV), followed by a gradient (8 CV): 0%$_{v/v}$→40%$_{v/v}$ and (20 CV): 40%$_{v/v}$→100%$_{v/v}$ (acetonitrile + 0.1%$_{v/v}$ FA) in (H_2O + 0.1%$_{v/v}$ FA)) and subsequent purification using flash column chromatography (10 g Sfär Silica Duo cartridge; 40 mL/min; 100%$_{v/v}$ cyclohexane (2 CV), followed by a linear gradient (25 CV): 0%$_{v/v}$→50%$_{v/v}$ ethyl acetate in cyclohexane).

White solid; m.p.: 135–137 °C; ^{1}H NMR (600 MHz, 300 K, acetone-d_6): δ = 11.96 (s, 1H), 11.44 (s, 1H), 8.72 (d, *J* = 8.6 Hz, 1H), 8.36 – 8.27 (m, 2H), 8.18 (d, *J* = 8.2 Hz, 1H), 8.14 (d, *J* = 8.2 Hz, 1H), 8.03 – 7.95 (m, 4H), 7.77 (dd, *J* = 7.2, 1.2 Hz, 1H), 7.63 – 7.55 (m, 5H), 7.53 (ddd, *J* = 8.0, 6.8, 1.1 Hz, 1H), 7.46 (s, 1H), 7.42 – 7.38 ppm (m, 1H); ^{13}C NMR (150 MHz, 300 K, acetone-d_6): δ = 167.8, 161.0, 147.4, 140.2, 136.09, 135.2, 134.9, 134.4, 134.2 (q, *J* = 32.6 Hz), 132.5, 132.2, 131.6, 131.5, 130.1, 130.0, 129.3, 129.3, 128.5 (2C), 127.9, 127.3, 127.0, 126.6, 126.2, 125.4, 125.0, 124.9, 122.5 (q, *J* = 272.3 Hz), 120.6, 117.6, 113.2 ppm; note that the ^{13}C NMR signals at 161.0 and 147.4 ppm were assigned from HMBC. ^{19}F NMR (565 MHz, 300 K, acetone-d_6): δ = -64.1 ppm (s, 3F); IR (film): ṽ = 3022, 2836, 1702, 1548, 1423, 1333, 1266, 1163, 1135 cm^{-1}; HRMS (ESI): m/z calculated for $C_{31}H_{21}F_3N_3O_3S_2$ [M+H]$^+$: 604.0971, found: 604.0971.

2-(Naphthalene-1-sulfonamido)-5-(trifluoromethyl)benzoic acid (33a)

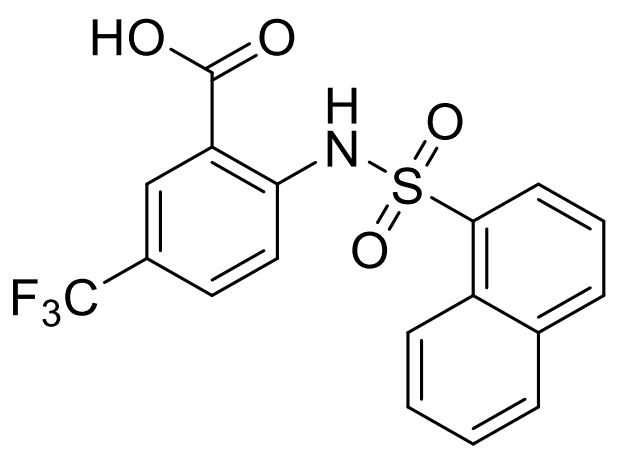

According to General Procedure A, sulfonamide **33a** (612 mg, 1.5 mmol, 52%) was obtained from commercially-sourced 2-amino-5-(trifluoromethyl)benzoic acid (615 mg, 3.0 mmol) and naphthalene-1-sulfonyl chloride (1.36 g, 6.0 mmol), followed by reverse phase column chromatography (12 g Sfär C18 Duo cartridge; 12 mL/min; 100%$_{v/v}$ (H_2O + 0.1%$_{v/v}$ FA) (2 CV), followed by a gradient (30 CV): 0%$_{v/v}$→100%$_{v/v}$ (acetonitrile + 0.1%$_{v/v}$ FA) in (H_2O + 0.1%$_{v/v}$ FA)).

Pale yellow solid; m.p.: 192–194 °C; ^{1}H NMR (400 MHz, 300 K, DMSO-*d₆*): δ = 12.50 – 12.46 (m, 1H), 8.97 (d, *J* = 8.6 Hz, 1H), 8.83 (dd, *J* = 7.4, 1.2 Hz, 1H), 8.71 (d, *J* = 8.3 Hz, 1H), 8.55 – 8.46 (m, 2H), 8.28 – 8.15 (m, 2H), 8.14 – 8.00 ppm (m, 3H); ^{13}C NMR (100 MHz, 300 K, DMSO-d_6): δ = 168.8, 143.1, 135.5, 133.9, 132.8, 131.1, 130.6, 129.5, 128.8, 128.3 (q, *J* = 3.9 Hz), 127.3, 126.9, 125.9 (q, *J* = 274.5 Hz), 124.6, 123.3, 122.7 (q, *J* = 33.2 Hz), 117.5, 116.0 ppm; ^{19}F NMR (470 MHz, 300 K, DMSO-d_6): δ = -61.1 ppm (s, 3F); IR (film): ṽ = 3213, 1677, 1571, 1508, 1402, 1402, 1295, 1255, 1162, 1125, 1094 cm^{-1}; HRMS (ESI): m/z calculated for $C_{18}H_{11}F_3NO_4S$ $[M-H]^-$: 394.0366, found: 394.0367.

***N*-(4-(Naphthalen-1-yl)thiazol-2-yl)-2-(naphthalene-1-sulfonamido)-5-(trifluoromethyl)benzamide (33)**

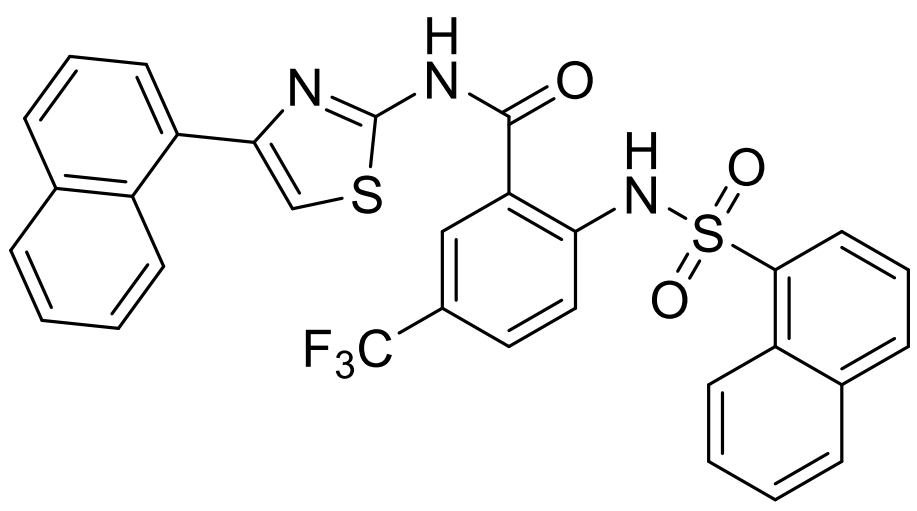

According to General Procedure B, amide **33** (212 mg, 0.35 mmol, 47%) was obtained from the carboxylic acid **33a** (297 mg, 0.75 mmol) and commercially-sourced 4-(naphthalen-1-yl)thiazol-2-amine (158 mg, 0.75 mmol), followed by reverse phase column chromatography (12 g Sfär C18 Duo cartridge; 12 mL/min; 100%$_{v/v}$ (H_2O + 0.1%$_{v/v}$ FA) (2 CV), followed by a gradient (8 CV): 0%$_{v/v}$→40%$_{v/v}$ and (20 CV): 40%$_{v/v}$→100%$_{v/v}$ (acetonitrile + 0.1%$_{v/v}$ FA) in (H_2O + 0.1%$_{v/v}$ FA)) and subsequent purification using flash column chromatography (10 g Sfär Silica Duo cartridge; 40 mL/min; 100%$_{v/v}$ cyclohexane (2 CV), followed by a linear gradient (25 CV): 0%$_{v/v}$→50%$_{v/v}$ ethyl acetate in cyclohexane).

White solid; m.p.: 168–170 °C; ^{1}H NMR (600 MHz, 300 K, acetone-d_6): δ = 12.14 (s, 1H), 11.85 (s, 1H), 8.74 (d, *J* = 8.6 Hz, 1H), 8.38 (dd, *J* = 7.4, 1.2 Hz, 1H), 8.37 (d, *J* = 2.3 Hz, 1H), 8.35 – 8.29 (m, 1H), 8.20 (dt, *J* = 8.5, 1.1 Hz, 1H), 8.00 (td, *J* = 7.1, 2.2 Hz, 3H), 7.88 (d, *J* = 8.8 Hz, 1H), 7.81 – 7.75 (m, 2H), 7.69 – 7.55 (m, 6H), 7.47 ppm (s, 1H); ^{13}C NMR (150 MHz, 300 K, acetone-d_6): δ = 168.5, 161.5, 147.0, 143.2, 136.1, 135.3, 134.9, 134.6, 132.2 (2C), 131.6, 130.7 (q, *J* = 3.7 Hz), 130.2, 130.0, 129.4, 129.3, 128.5, 128.5, 128.0 (q, *J* = 4.2 Hz), 128.0, 127.4, 127.0, 126.6, 126.2, 125.1, 125.0 (q, *J* = 36.0 Hz), 124.9, 123.0 (q, *J* = 271.1 Hz), 121.3, 120.4, 113.1 ppm; note that the ^{13}C NMR signals at 161.5 and 147.0 ppm were assigned using HMBC. ^{19}F NMR (565 MHz, 300 K, acetone-d_6): δ = -62.8 ppm (s, 3F); IR (film): ṽ = 3493, 2998, 1707, 1650, 1590, 1344, 1315, 1285, 1164, 1124, 1092 cm^{-1}; HRMS (ESI): m/z calculated for $C_{31}H_{20}F_3N_3O_3S_2Na$ $[M+Na]^+$: 626.0790, found: 626.0816.

2-((4-(*tert*-Butyl)phenyl)sulfonamido)-*N*-(4-phenylthiazol-2-yl)-4-(trifluoromethyl)benzamide (34)

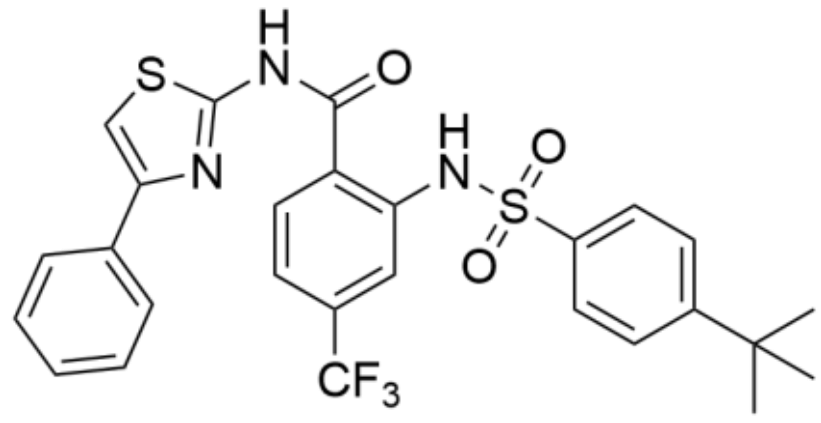

According to General Procedure B, amide **34** (72.3 mg, 0.13 mmol, 26%) was obtained from the carboxylic acid **16a** (200 mg, 0.5 mmol) and commercially-sourced 4-phenylthiazol-2-amine (88.1 mg, 0.50 mmol), followed by reverse phase column chromatography (12 g Sfär C18 Duo cartridge; 12 mL/min; 100%$_{v/v}$ (H_2O + 0.1%$_{v/v}$ FA) (2 CV), followed by a gradient (8 CV): 0%$_{v/v}$→40%$_{v/v}$ and (20 CV): 40%$_{v/v}$→100%$_{v/v}$ (acetonitrile + 0.1%$_{v/v}$ FA) in (H_2O + 0.1%$_{v/v}$ FA)) and subsequent purification using flash column chromatography (10 g Sfär Silica Duo cartridge; 40 mL/min; 100%$_{v/v}$ cyclohexane (2 CV), followed by a linear gradient (25 CV): 0%$_{v/v}$→50%$_{v/v}$ ethyl acetate in cyclohexane).

White solid; m.p.: >200 °C (decomposition); ^{1}H NMR (600 MHz, 300 K, MeOD-*d$_4$*): δ = 7.96 (s, 1H), 7.91 (d, *J* = 7.7 Hz, 2H), 7.88 (d, *J* = 1.8 Hz, 1H), 7.62 – 7.52 (m, 3H), 7.47 (s, 1H), 7.41 (t, *J* = 7.6 Hz, 2H), 7.38 – 7.31 (m, 3H), 1.13 ppm (s, 9H); ^{13}C NMR (150 MHz, 300 K, MeOD-*d$_4$*): δ = 166.8, 158.7, 151.6, 139.8, 136.9, 135.7, 135.1 (q, *J* = 33.1 Hz), 131.0, 129.7 (2C), 129.1, 128.9, 128.1 (2C), 127.3 (2C), 127.2 (3C), 124.7 (q, *J* = 272.0 Hz), 122.9 (2C), 109.4, 35.9, 31.2 ppm (3C); note that the ^{13}C NMR signals at 166.8, 151.6 and 139.8 ppm were assigned using HMBC. ^{19}F NMR (565 MHz, 300 K, MeOD-*d$_4$*): δ = -65.7 ppm (s, 3F); IR (film): ṽ = 2959, 1639, 1596, 1497, 1394, 1333, 1261, 1170, 1033 cm^{-1}; HRMS (ESI): m/z calculated for $C_{27}H_{25}F_3N_3O_3S_2$ [M+H]$^+$: 560.1284, found: 560.1303.

2-((4-Methylphenyl)sulfonamido)-*N*-(4-phenylthiazol-2-yl)benzamide (35)

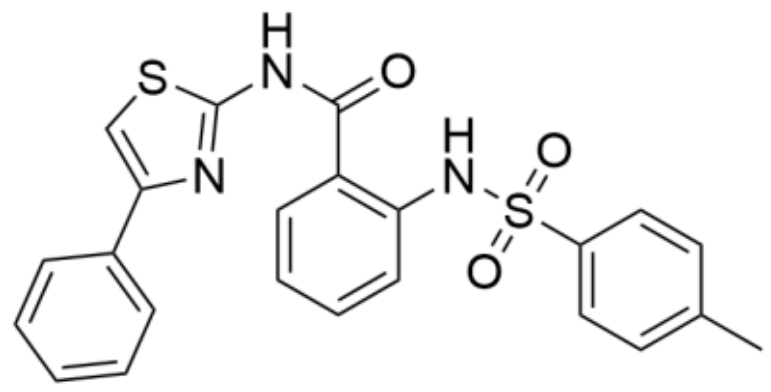

According to General Procedure B, amide **35** (88.5 mg, 0.20 mmol, 39%) was obtained from the carboxylic acid **21a** (147 mg, 0.50 mmol) and commercially-sourced 4-phenylthiazol-2-amine (88.1 mg, 0.50 mmol), followed by reverse phase column chromatography (12 g Sfär C18 Duo cartridge; 12 mL/min; 100%$_{v/v}$ (H_2O + 0.1%$_{v/v}$ FA) (2 CV), followed by a gradient (8 CV): 0%$_{v/v}$→40%$_{v/v}$ and (20 CV): 40%$_{v/v}$→100%$_{v/v}$ (acetonitrile + 0.1%$_{v/v}$ FA) in (H_2O + 0.1%$_{v/v}$ FA)) and subsequent purification using flash column chromatography (10 g Sfär Silica Duo cartridge; 40 mL/min; 100%$_{v/v}$ cyclohexane (2 CV), followed by a linear gradient (25 CV): 0%$_{v/v}$→50%$_{v/v}$ ethyl acetate in cyclohexane).

White solid, m.p.: 164–166 °C; ^{1}H NMR (400 MHz, 300 K, MeOD-*d$_4$*): δ = 7.97 – 7.88 (m, 2H), 7.71 (dd, *J* = 7.9, 1.5 Hz, 1H), 7.64 (dd, *J* = 8.2, 1.3 Hz, 1H), 7.56 (ddd, *J* = 8.3, 7.4, 1.5 Hz, 1H), 7.49 – 7.36 (m, 5H), 7.36 – 7.25 (m, 2H), 7.03 (d, *J* = 8.1 Hz, 2H), 2.15 ppm (s, 3H); ^{13}C NMR (100 MHz, 300 K, MeOD-*d$_4$*): δ = 168.0, 159.5, 151.1, 145.6, 138.8, 136.8, 135.8, 134.0, 130.7 (2C), 129.7 (2C), 129.6, 129.0, 128.2 (2C), 127.1 (2C), 126.6, 126.3, 125.8, 109.2, 21.5 ppm; IR (film): ṽ = 2982, 1641, 1582, 1333, 1333, 1161, 1090 cm^{-1}; HRMS (ESI): m/z calculated for $C_{23}H_{20}N_3O_3S_2$ [M+H]$^+$: 450.0941, found: 450.0954.

4-((4-Methylphenyl)sulfonamido)-*N*-(4-phenylthiazol-2-yl)benzamide (36)

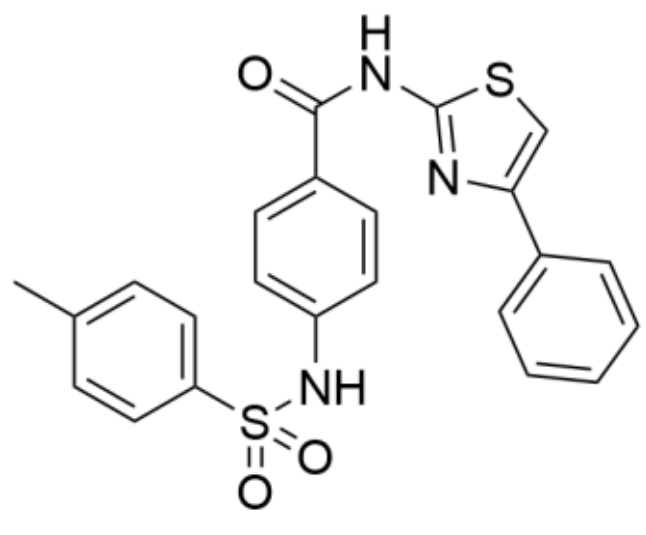

According to General Procedure B, amide **36** (89.6 mg, 0.20 mmol, 40%) was obtained from the carboxylic acid **24a** (147 mg, 0.50 mmol) and commercially-sourced 4-phenylthiazol-2-amine (88.1 mg, 0.50 mmol), followed by reverse phase column chromatography (12 g Sfär C18 Duo cartridge; 12 mL/min; 100%$_{v/v}$ (H_2O + 0.1%$_{v/v}$ FA) (2 CV), followed by a gradient (8 CV): 0%$_{v/v}$→40%$_{v/v}$ and (20 CV): 40%$_{v/v}$→100%$_{v/v}$ (acetonitrile + 0.1%$_{v/v}$ FA) in (H_2O + 0.1%$_{v/v}$ FA)).

White solid; m.p.: 125–127 °C; ^{1}H NMR (400 MHz, 300 K, MeOD-d_4): δ = 7.95 – 7.86 (m, 4H), 7.78 – 7.69 (m, 2H), 7.40 – 7.33 (m, 3H), 7.33 – 7.20 (m, 5H), 2.35 ppm (s, 3H); ^{13}C NMR (100 MHz, 300 K, MeOD-d_4): δ = 166.7, 159.9, 151.4, 145.4, 143.6, 138.0, 136.0, 130.8 (2C), 130.3 (2C), 129.6 (2C), 128.8, 128.7, 128.3 (2C), 127.1 (2C), 120.0 (2C), 108.9, 21.4 ppm; IR (film): ṽ = 3612, 1667, 1607, 1541, 1481, 1445, 1330, 1280, 1159, 1090 cm^{-1}; HRMS (ESI): m/z calculated for $C_{23}H_{18}N_3O_3S_2$ [M-H]$^-$: 448.0795, found: 448.0791.

2-((4-Methylphenyl)sulfonamido)-*N*-(4-phenylthiazol-2-yl)-5-(trifluoromethyl)benzamide (37)

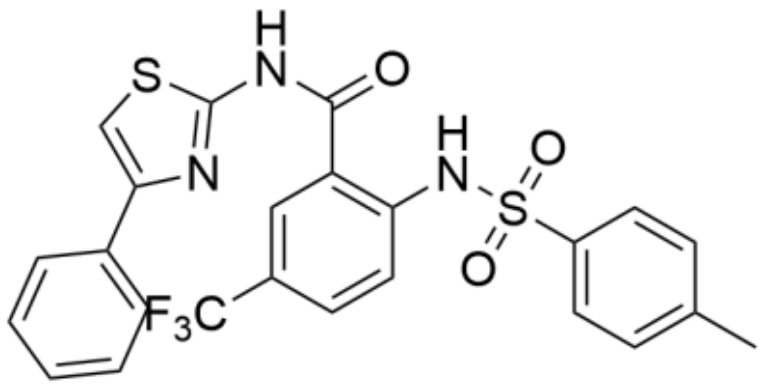

According to General Procedure B, amide **37** (41 mg, 0.079 mmol, 11%) was obtained from the carboxylic acid **25a** (270 mg, 0.75 mmol) and commercially-sourced 4-phenylthiazol-2-amine (132 mg, 0.75 mmol), followed by reverse phase column chromatography (12 g Sfär C18 Duo cartridge; 12 mL/min; 100%$_{v/v}$ (H_2O + 0.1%$_{v/v}$ FA) (2 CV), followed by a gradient (8 CV): 0%$_{v/v}$→40%$_{v/v}$ and (20 CV): 40%$_{v/v}$→100%$_{v/v}$ (acetonitrile + 0.1%$_{v/v}$ FA) in (H_2O + 0.1%$_{v/v}$ FA)) and subsequent purification using flash column chromatography (10 g Sfär Silica Duo cartridge; 40 mL/min; 100%$_{v/v}$ cyclohexane (2 CV), followed by a linear gradient (25 CV): 0%$_{v/v}$→50%$_{v/v}$ ethyl acetate in cyclohexane).

White solid; m.p.: 107–109 °C; ^{1}H NMR (600 MHz, 300 K, acetone-d_6): δ = 8.44 (q, J = 1.2 Hz, 1H), 7.97 – 7.92 (m, 2H), 7.90 (d, J = 1.3 Hz, 2H), 7.79 – 7.74 (m, 2H), 7.63 (s, 1H), 7.48 – 7.42 (m, 2H), 7.38 – 7.33 (m, 1H), 7.32 – 7.26 (m, 2H), 2.29 ppm (s, 3H); ^{13}C NMR (150 MHz, 300 K, acetone-d_6): δ = 167.3, 159.6, 149.7, 145.4, 143.4, 137.1, 134.8, 130.9 (q, J = 3.9 Hz), 130.8 (2C), 129.6 (2C), 129.0, 128.1 (2C), 127.8 (q, J = 3.8 Hz), 126.8 (2C), 125.3, 124.8 (q, J = 271.3 Hz), 121.7, 121.5, 109.4, 21.4 ppm; ^{19}F NMR (565 MHz, 300 K, acetone-d_6): δ = -62.8 ppm (s, 3F); IR (film): ṽ = 3658, 2991, 1639, 1602, 1550, 1446, 1308, 1162 cm^{-1}; HRMS (ESI): m/z calculated for $C_{24}H_{19}F_3N_3O_3S_2$ [M+H]$^+$: 518.0815, found: 518.0809.

7. References

[1] J.-H. Zhang, T. D. Y. Chung, K. R. Oldenburg, "A simple statistical parameter for use in evaluation and validation of high throughput screening assays", *J. Biomol. Screen.* **1999**, *4*, 67–73.

[2] L. Brewitz, H. T. Henry Chan, P. Lukacik, C. Strain-Damerell, M. A. Walsh, F. Duarte, C. J. Schofield, "Mass spectrometric assays monitoring the deubiquitinase activity of the SARS-CoV-2 papain-like protease inform on the basis of substrate selectivity and have utility for substrate identification", *Bioorg. Med. Chem.* **2023**, *95*, 117498.

[3] P. M. Wydorski, J. Osipiuk, B. T. Lanham, C. Tesar, M. Endres, E. Engle, R. Jedrzejczak, V. Mullapudi, K. Michalska, K. Fidelis, D. Fushman, A. Joachimiak, L. A. Joachimiak, "Dual domain recognition determines SARS-CoV-2 PLpro selectivity for human ISG15 and K48-linked di-ubiquitin", *Nat. Commun.* **2023**, *14*, 2366.

[4] Q. Yue, J. Hua-Juan, Y. Yu-Shun, H. Xiao-Qin, Z. Xue-Wen, "Review of the crystallized structures of the SARS-CoV-2 papain-like protease", *J. Mol. Struct.* **2025**, *1333*, 141730.

[5] S. Ullrich, C. Nitsche, "SARS-CoV-2 papain-like protease: structure, function and inhibition", *ChemBioChem* **2022**, *23*, e202200327.

[6] K. Ratia, S. Pegan, J. Takayama, K. Sleeman, M. Coughlin, S. Baliji, R. Chaudhuri, W. Fu, B. S. Prabhakar, M. E. Johnson, S. C. Baker, A. K. Ghosh, A. D. Mesecar, "A noncovalent class of papain-like protease/deubiquitinase inhibitors blocks SARS virus replication", *Proc. Natl. Acad. Sci. USA* **2008**, *105*, 16119–16124.

[7] J. Osipiuk, S.-A. Azizi, S. Dvorkin, M. Endres, R. Jedrzejczak, K. A. Jones, S. Kang, R. S. Kathayat, Y. Kim, V. G. Lisnyak, S. L. Maki, V. Nicolaescu, C. A. Taylor, C. Tesar, Y.-A. Zhang, Z. Zhou, G. Randall, K. Michalska, S. A. Snyder, B. C. Dickinson, A. Joachimiak, "Structure of papain-like protease from SARS-CoV-2 and its complexes with non-covalent inhibitors", *Nat. Commun.* **2021**, *12*, 743.

[8] C. Ma, M. D. Sacco, Z. Xia, G. Lambrinidis, J. A. Townsend, Y. Hu, X. Meng, T. Szeto, M. Ba, X. Zhang, M. Gongora, F. Zhang, M. T. Marty, Y. Xiang, A. Kolocouris, Y. Chen, J. Wang, "Discovery of SARS-CoV-2 papain-like protease inhibitors through a combination of high-throughput screening and a FlipGFP-based reporter assay", *ACS Cent. Sci.* **2021**, *7*, 1245–1260.

[9] L. Brewitz, J. J. A. G. Kamps, P. Lukacik, C. Strain-Damerell, Y. Zhao, A. Tumber, T. R. Malla, A. M. Orville, M. A. Walsh, C. J. Schofield, "Mass spectrometric assays reveal discrepancies in inhibition profiles for the SARS-CoV-2 papain-like protease", *ChemMedChem* **2022**, *17*, e202200016.

[10] L. A. Armstrong, S. M. Lange, V. Dee Cesare, S. P. Matthews, R. S. Nirujogi, I. Cole, A. Hope, F. Cunningham, R. Toth, R. Mukherjee, D. Bojkova, F. Gruber, D. Gray, P. G. Wyatt, J. Cinatl, I. Dikic, P. Davies, Y. Kulathu, "Biochemical characterization of protease activity of Nsp3 from SARS-CoV-2 and its inhibition by nanobodies", *PLoS One* **2021**, *16*, e0253364.

[11] B. T. Freitas, I. A. Durie, J. Murray, J. E. Longo, H. C. Miller, D. Crich, R. J. Hogan, R. A. Tripp, S. D. Pegan, "Characterization and noncovalent inhibition of the deubiquitinase and deISGylase activity of SARS-CoV-2 papain-like protease", *ACS Infect. Dis.* **2020**, *6*, 2099–2109.

[12] Z. Fu, B. Huang, J. Tang, S. Liu, M. Liu, Y. Ye, Z. Liu, Y. Xiong, W. Zhu, D. Cao, J. Li, X. Niu, H. Zhou, Y. J. Zhao, G. Zhang, H. Huang, "The complex structure of GRL0617 and SARS-CoV-2 PLpro reveals a hot spot for antiviral drug discovery", *Nat. Commun.* **2021**, *12*, 488.

[13] Z. Shen, K. Ratia, L. Cooper, D. Kong, H. Lee, Y. Kwon, Y. Li, S. Alqarni, F. Huang, O. Dubrovskyi, L. Rong, G. R. J. Thatcher, R. Xiong, "Design of SARS-CoV-2 PLpro inhibitors for COVID-19 antiviral therapy leveraging binding cooperativity", *J. Med. Chem.* **2022**, *65*, 2940–2955.

[14] Y. Zhao, X. Du, Y. Duan, X. Pan, Y. Sun, T. You, L. Han, Z. Jin, W. Shang, J. Yu, H. Guo, Q. Liu, Y. Wu, C. Peng, J. Wang, C. Zhu, X. Yang, K. Yang, Y. Lei, L. W. Guddat, W. Xu, G. Xiao, L. Sun, L. Zhang, Z. Rao, H. Yang, "High-throughput screening identifies established drugs as SARS-CoV-2 PLpro inhibitors", *Protein Cell* **2021**, *12*, 877–888.

[15] Y. Xu, K. Chen, J. Pan, Y. Lei, D. Zhang, L. Fang, J. Tang, X. Chen, Y. Ma, Y. Zheng, B. Zhang, Y. Zhou, J. Zhan, W. Xu, "Repurposing clinically approved drugs for COVID-19 treatment targeting SARS-CoV-2 papain-like protease", *Int. J. Biol. Macromol.* **2021**, *188*, 137–146.

[16] M. A. Redhead, C. D. Owen, L. Brewitz, A. H. Collette, P. Lukacik, C. Strain-Damerell, S. W. Robinson, P. M. Collins, P. Schäfer, M. Swindells, C. J. Radoux, I. N. Hopkins, D. Fearon, A. Douangamath, F. von Delft, T. R. Malla, L. Vangeel, T. Vercruysse, J. Thibaut, P. Leyssen, T.-T. Nguyen, M. Hull, A. Tumber, D. J. Hallett, C. J. Schofield, D. I. Stuart, A. L. Hopkins, M. A. Walsh, "Bispecific repurposed medicines targeting the viral and immunological arms of COVID-19", *Sci. Rep.* **2021**, *11*, 13208.

[17] M. Gil-Moles, U. Basu, R. Büssing, H. Hoffmeister, S. Türck, A. Varchmin, I. Ott, "Gold metallodrugs to target coronavirus proteins: inhibitory effects on the Spike-ACE2 interaction and on PLpro protease activity by auranofin and gold organometallics", *Chem. Eur. J.* **2020**, *26*, 15140–15144.

[18] E. Weglarz-Tomczak, J. M. Tomczak, M. Talma, M. Burda-Grabowska, M. Giurg, S. Brul, "Identification of ebselen and its analogues as potent covalent inhibitors of papain-like protease from SARS-CoV-2", *Sci. Rep.* **2021**, *11*, 3640.

[19] M. Zmudzinski, W. Rut, K. Olech, J. Granda, M. Giurg, M. Burda-Grabowska, L. Zhang, X. Sun, Z. Lv, D. Nayak, M. Kesik-Brodacka, S. K. Olsen, R. Hilgenfeld, M. Drag, "Ebselen derivatives are very potent dual inhibitors of SARS-CoV-2 proteases - PLpro and Mpro in in vitro studies", *bioRxiv* **2020**, DOI: 10.1101/2020.08.30.273979.

[20] T. R. Malla, A. Tumber, T. John, L. Brewitz, C. Strain-Damerell, C. D. Owen, P. Lukacik, H. T. H. Chan, P. Maheswaran, E. Salah, F. Duarte, H. Yang, Z. Rao, M. A. Walsh, C. J. Schofield, "Mass spectrometry reveals potential of β-lactams as SARS-CoV-2 Mpro inhibitors", *Chem. Commun.* **2021**, *57*, 1430–1433.

[21] T. R. Malla, L. Brewitz, D.-G. Muntean, H. Aslam, C. D. Owen, E. Salah, A. Tumber, P. Lukacik, C. Strain-Damerell, H. Mikolajek, M. A. Walsh, C. J. Schofield, "Penicillin derivatives inhibit the SARS-CoV-2 main protease by reaction with its nucleophilic cysteine", *J. Med. Chem.* **2022**, *65*, 7682–7696.

[22] L. Brewitz, L. Dumjahn, Y. Zhao, C. D. Owen, S. M. Laidlaw, T. R. Malla, D. Nguyen, P. Lukacik, E. Salah, A. D. Crawshaw, A. J. Warren, J. Trincao, C. Strain-Damerell, M. W. Carroll, M. A. Walsh, C. J. Schofield, "Alkyne derivatives of SARS-CoV-2 main protease inhibitors including nirmatrelvir inhibit by reacting covalently with the nucleophilic cysteine", *J. Med. Chem.* **2023**, *66*, 2663–2680.

[23] W. Rut, Z. Lv, M. Zmudzinski, S. Patchett, D. Nayak, S. J. Snipas, F. El Oualid, T. T. Huang, M. Bekes, M. Drag, S. K. Olsen, "Activity profiling and crystal structures of inhibitor-bound SARS-CoV-2 papain-like protease: A framework for anti–COVID-19 drug design", *Sci. Adv.* **2020**, *6*, eabd4596.

[24] G. Madhavi Sastry, M. Adzhigirey, T. Day, R. Annabhimoju, W. Sherman, "Protein and ligand preparation: parameters, protocols, and influence on virtual screening enrichments", *J. Comput. Aided Mol. Des.* **2013**, *27*, 221–234.

[25] Schrödinger Release 2024-2: Maestro, Schrödinger, LLC, New York, NY, **2024**.

[26] R. A. Friesner, R. B. Murphy, M. P. Repasky, L. L. Frye, J. R. Greenwood, T. A. Halgren, P. C. Sanschagrin, D. T. Mainz, "Extra Precision Glide: docking and scoring incorporating a model of hydrophobic enclosure for protein−ligand complexes", *J. Med. Chem.* **2006**, *49*, 6177–6196.

[27] J. Wang, R. M. Wolf, J. W. Caldwell, P. A. Kollman, D. A. Case, "Development and testing of a general amber force field", *J. Comput. Chem.* **2004**, *25*, 1157–1174.

[28] J. Wang, W. Wang, P. A. Kollman, D. A. Case, "Automatic atom type and bond type perception in molecular mechanical calculations", *J. Mol. Graph. Model.* **2006**, *25*, 247–260.

[29] M. J. Frisch, G. W. Trucks, H. B. Schlegel, G. E. Scuseria, M. A. Robb, J. R. Cheeseman, G. Scalmani, V. Barone, G. A. Petersson, H. Nakatsuji, X. Li, M. Caricato, A. V. Marenich, J. Bloino, B. G. Janesko, R. Gomperts, B. Mennucci, H. P. Hratchian, J. V. Ortiz, A. F. Izmaylov, J. L. Sonnenberg, Williams, F. Ding, F. Lipparini, F. Egidi, J. Goings, B. Peng, A. Petrone, T. Henderson, D. Ranasinghe, V. G. Zakrzewski, J. Gao, N. Rega, G. Zheng, W. Liang, M. Hada, M. Ehara, K. Toyota, R. Fukuda, J. Hasegawa, M. Ishida, T. Nakajima, Y. Honda, O. Kitao, H. Nakai, T. Vreven, K. Throssell, J. A. Montgomery Jr., J. E. Peralta, F. Ogliaro, M. J. Bearpark, J. J. Heyd, E. N. Brothers, K. N. Kudin, V. N. Staroverov, T. A. Keith, R. Kobayashi, J. Normand, K. Raghavachari, A. P. Rendell, J. C. Burant, S. S. Iyengar, J. Tomasi, M. Cossi, J. M. Millam, M. Klene, C. Adamo, R. Cammi, J. W. Ochterski, R. L. Martin, K. Morokuma, O. Farkas, J. B. Foresman, D. J. Fox in *Gaussian 16 Revision C.01*, Gaussian, Inc., Wallingford, CT, **2016**.

[30] C. I. Bayly, P. Cieplak, W. Cornell, P. A. Kollman, "A well-behaved electrostatic potential based method using charge restraints for deriving atomic charges: the RESP model", *J. Phys. Chem.* **1993**, *97*, 10269–10280.

[31] M. B. Peters, Y. Yang, B. Wang, L. Füsti-Molnár, M. N. Weaver, K. M. Merz, Jr., "Structural survey of zinc-

containing proteins and development of the Zinc AMBER Force Field (ZAFF)", *J. Chem. Theory Comput.* **2010**, *6*, 2935–2947.

[32] J. A. Maier, C. Martinez, K. Kasavajhala, L. Wickstrom, K. E. Hauser, C. Simmerling, "ff14SB: Improving the accuracy of protein side chain and backbone parameters from ff99SB", *J. Chem. Theory Comput.* **2015**, *11*, 3696–3713.

[33] H. M. A. D.A. Case, K. Belfon, I.Y. Ben-Shalom, J.T. Berryman, S.R. Brozell, D.S. Cerutti, T.E. Cheatham, III, G.A. Cisneros, V.W.D. Cruzeiro, T.A. Darden, N. Forouzesh, M. Ghazimirsaeed, G. Giambaşu, T. Giese, M.K. Gilson, H. Gohlke, A.W. Goetz, J. Harris, Z. Huang, S. Izadi, S.A. Izmailov, K. Kasavajhala, M.C. Kaymak, A. Kovalenko, T. Kurtzman, T.S. Lee, P. Li, Z. Li, C. Lin, J. Liu, T. Luchko, R. Luo, M. Machado, M. Manathunga, K.M. Merz, Y. Miao, O. Mikhailovskii, G. Monard, H. Nguyen, K.A. O'Hearn, A. Onufriev, F. Pan, S. Pantano, A. Rahnamoun, D.R. Roe, A. Roitberg, C. Sagui, S. Schott-Verdugo, A. Shajan, J. Shen, C.L. Simmerling, N.R. Skrynnikov, J. Smith, J. Swails, R.C. Walker, J. Wang, J. Wang, X. Wu, Y. Wu, Y. Xiong, Y. Xue, D.M. York, C. Zhao, Q. Zhu, and P.A. Kollman in *Amber 2024*, University of California, San Francisco, **2024**.

[34] D. A. Case, H. M. Aktulga, K. Belfon, D. S. Cerutti, G. A. Cisneros, V. W. D. Cruzeiro, N. Forouzesh, T. J. Giese, A. W. Götz, H. Gohlke, S. Izadi, K. Kasavajhala, M. C. Kaymak, E. King, T. Kurtzman, T.-S. Lee, P. Li, J. Liu, T. Luchko, R. Luo, M. Manathunga, M. R. Machado, H. M. Nguyen, K. A. O'Hearn, A. V. Onufriev, F. Pan, S. Pantano, R. Qi, A. Rahnamoun, A. Risheh, S. Schott-Verdugo, A. Shajan, J. Swails, J. Wang, H. Wei, X. Wu, Y. Wu, S. Zhang, S. Zhao, Q. Zhu, T. E. Cheatham, III, D. R. Roe, A. Roitberg, C. Simmerling, D. M. York, M. C. Nagan, K. M. Merz, Jr., "AmberTools", *J. Chem. Inf. Model.* **2023**, *63*, 6183–6191.

[35] W. L. Jorgensen, J. Chandrasekhar, J. D. Madura, R. W. Impey, M. L. Klein, "Comparison of simple potential functions for simulating liquid water", *J. Chem. Phys.* **1983**, *79*, 926–935.

[36] H. J. C. Berendsen, J. P. M. Postma, W. F. van Gunsteren, A. DiNola, J. R. Haak, "Molecular dynamics with coupling to an external bath", *J. Chem. Phys.* **1984**, *81*, 3684–3690.

[37] J.-P. Ryckaert, G. Ciccotti, H. J. C. Berendsen, "Numerical integration of the cartesian equations of motion of a system with constraints: molecular dynamics of n-alkanes", *J. Comput. Phys.* **1977**, *23*, 327–341.

[38] D. R. Roe, T. E. Cheatham, III, "PTRAJ and CPPTRAJ: Software for processing and analysis of molecular dynamics trajectory data", *J. Chem. Theory Comput.* **2013**, *9*, 3084–3095.

[39] B. R. Miller, III, T. D. McGee, Jr., J. M. Swails, N. Homeyer, H. Gohlke, A. E. Roitberg, "MMPBSA.py: An efficient program for end-state free energy calculations", *J. Chem. Theory Comput.* **2012**, *8*, 3314–3321.

[40] J. Weiser, P. S. Shenkin, W. C. Still, "Approximate atomic surfaces from linear combinations of pairwise overlaps (LCPO)", *J. Comput. Chem.* **1999**, *20*, 217–230.

[41] M. I. Davis, R. Pragani, J. T. Fox, M. Shen, K. Parmar, E. F. Gaudiano, L. Liu, C. Tanega, L. McGee, M. D. Hall, C. McKnight, P. Shinn, H. Nelson, D. Chattopadhyay, A. D. D'Andrea, D. S. Auld, L. J. DeLucas, Z. Li, M. B. Boxer, A. Simeonov, "Small molecule inhibition of the ubiquitin-specific protease USP2 accelerates cyclin D1 degradation and leads to cell cycle arrest in colorectal cancer and mantle cell lymphoma models", *J. Biol. Chem.* **2016**, *291*, 24628–24640.

[42] L. A. Carpino, "1-Hydroxy-7-azabenzotriazole. An efficient peptide coupling additive", *J. Am. Chem. Soc.* **1993**, *115*, 4397–4398.

[43] B. U. Emenike, A. T. Liu, E. P. Naveo, J. D. Roberts, "Substituent effects on energetics of peptide-carboxylate hydrogen bonds as studied by ^{1}H NMR spectroscopy: implications for enzyme catalysis", *J. Org. Chem.* **2013**, *78*, 11765–11771.

[44] Y. Son, J. Su Yang, S. Chul Shin, S. Kyoung Park, Y. Kim, J. Park, J. Yu, "Structural optimization and biological evaluation of ML364 based derivatives as USP2a inhibitors", *Bioorg. Chem.* **2024**, *145*, 107222.

[45] Y. Tian, K. Liu, D. Wu, L. Wu, Q. Xu, W. Wei, Z. Li, Q. Du, J. Bian, "The discovery of potent USP2/USP8 dual-target inhibitors for the treatment of breast cancer via structure guided optimization of ML364", *Eur. J. Med. Chem.* **2024**, *268*, 116275.

[46] X. Deng, N. S. Mani, "A facile, environmentally benign sulfonamide synthesis in water", *Green Chem.* **2006**, *8*, 835–838.

[47] D. Lee, S. Chang, "Direct C-H amidation of benzoic acids to introduce meta- and para-amino groups by tandem decarboxylation", *Chem. Eur. J.* **2015**, *21*, 5364–5368.

8. ^{1}H, ^{13}C and ^{19}F NMR spectra of synthesised ML364 derivatives

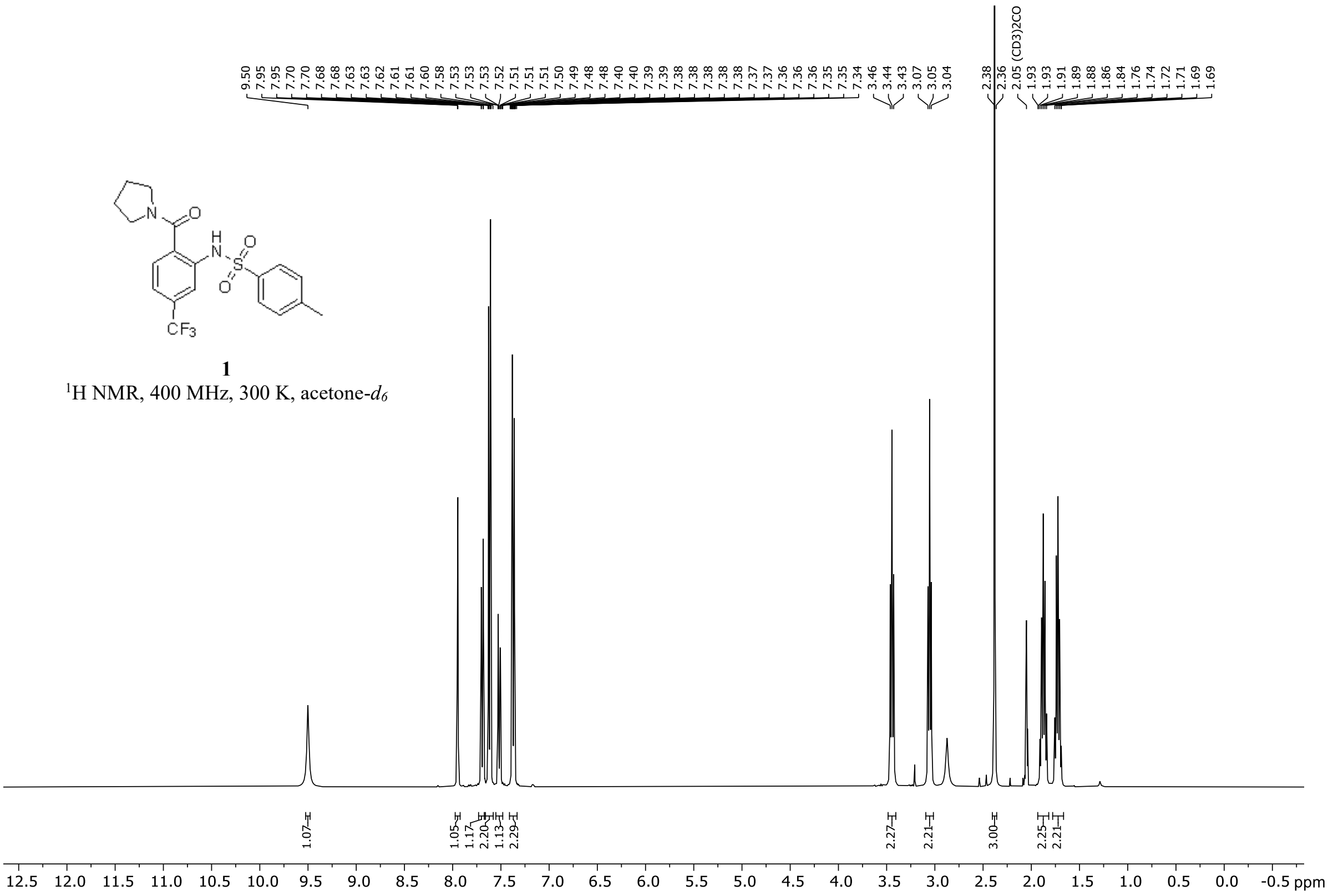

1
^{1}H NMR, 400 MHz, 300 K, acetone-d_6

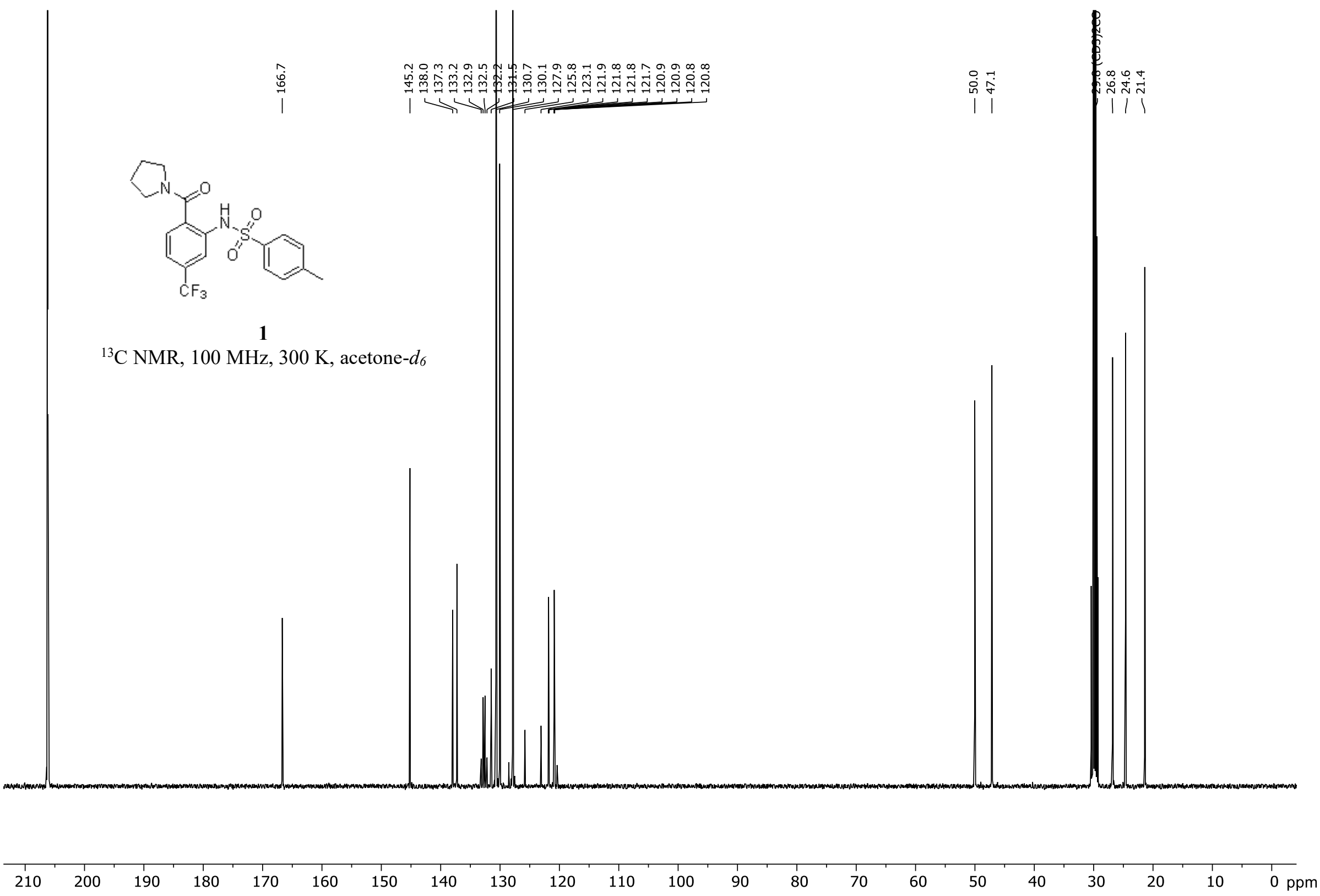

1
^{13}C NMR, 100 MHz, 300 K, acetone-d_6

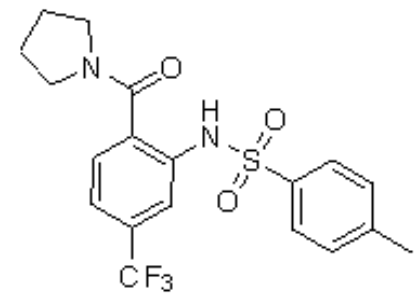

1

^{19}F NMR, 375 MHz, 300 K, acetone-d_6

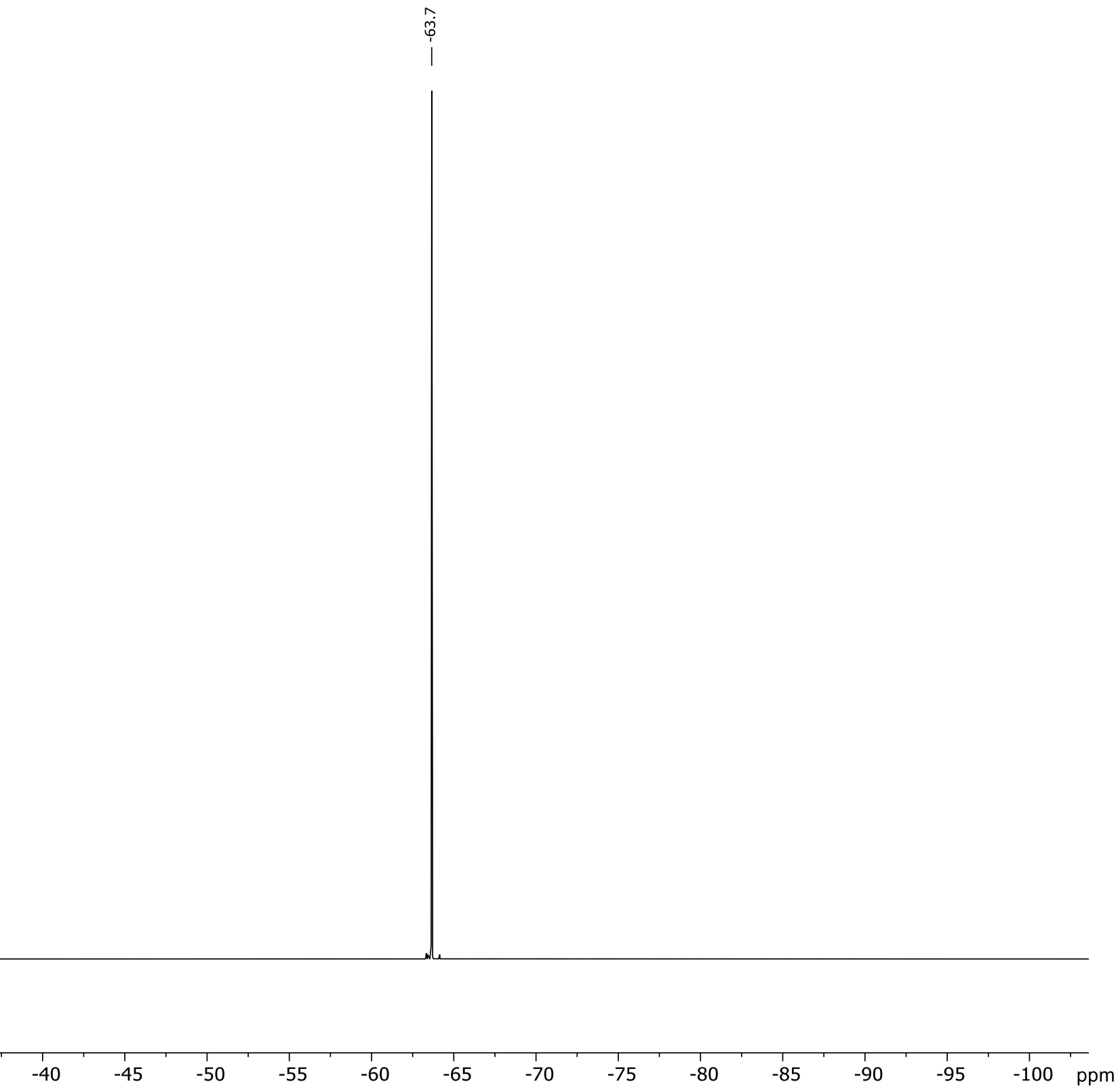

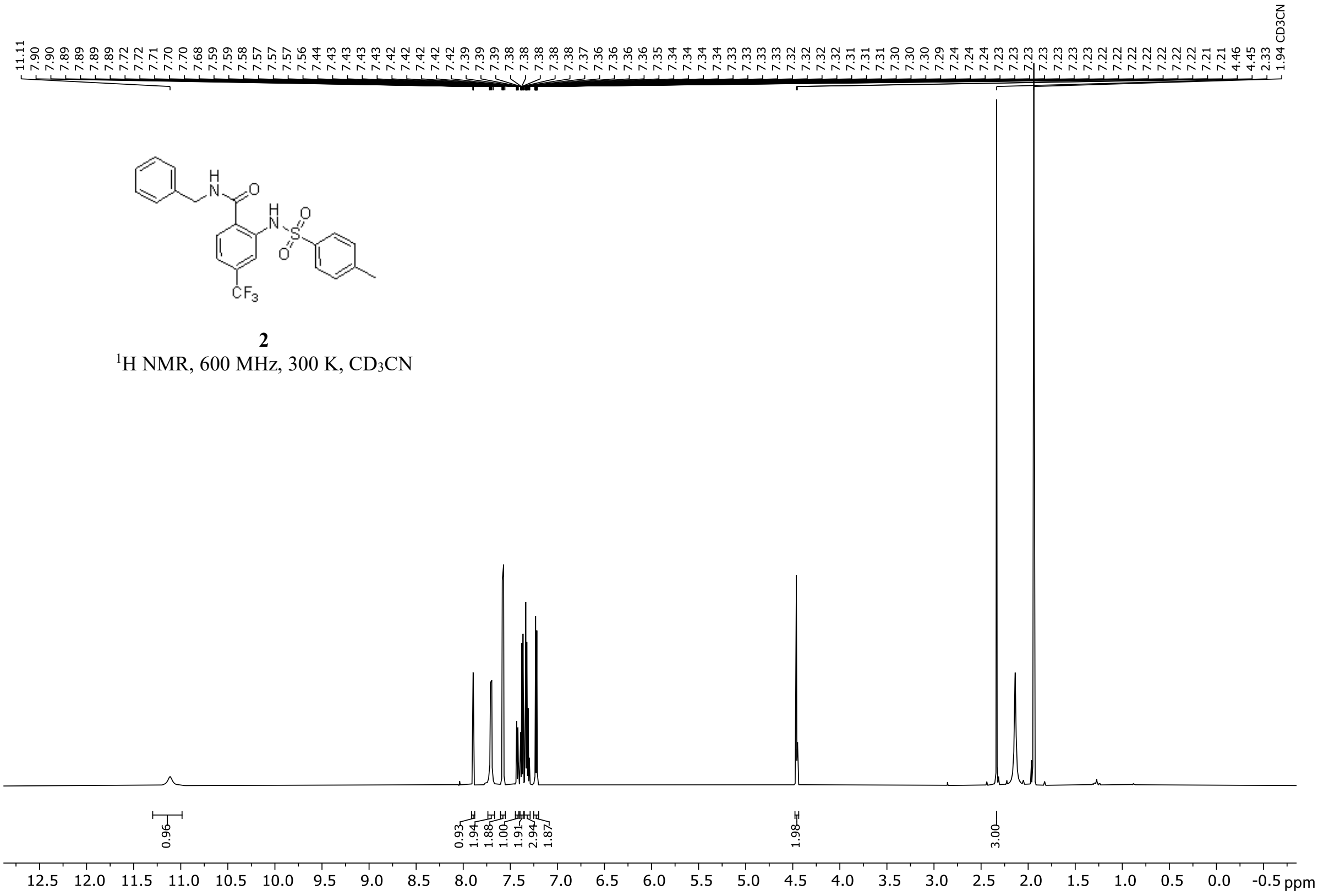

2

^{1}H NMR, 600 MHz, 300 K, CD_3CN

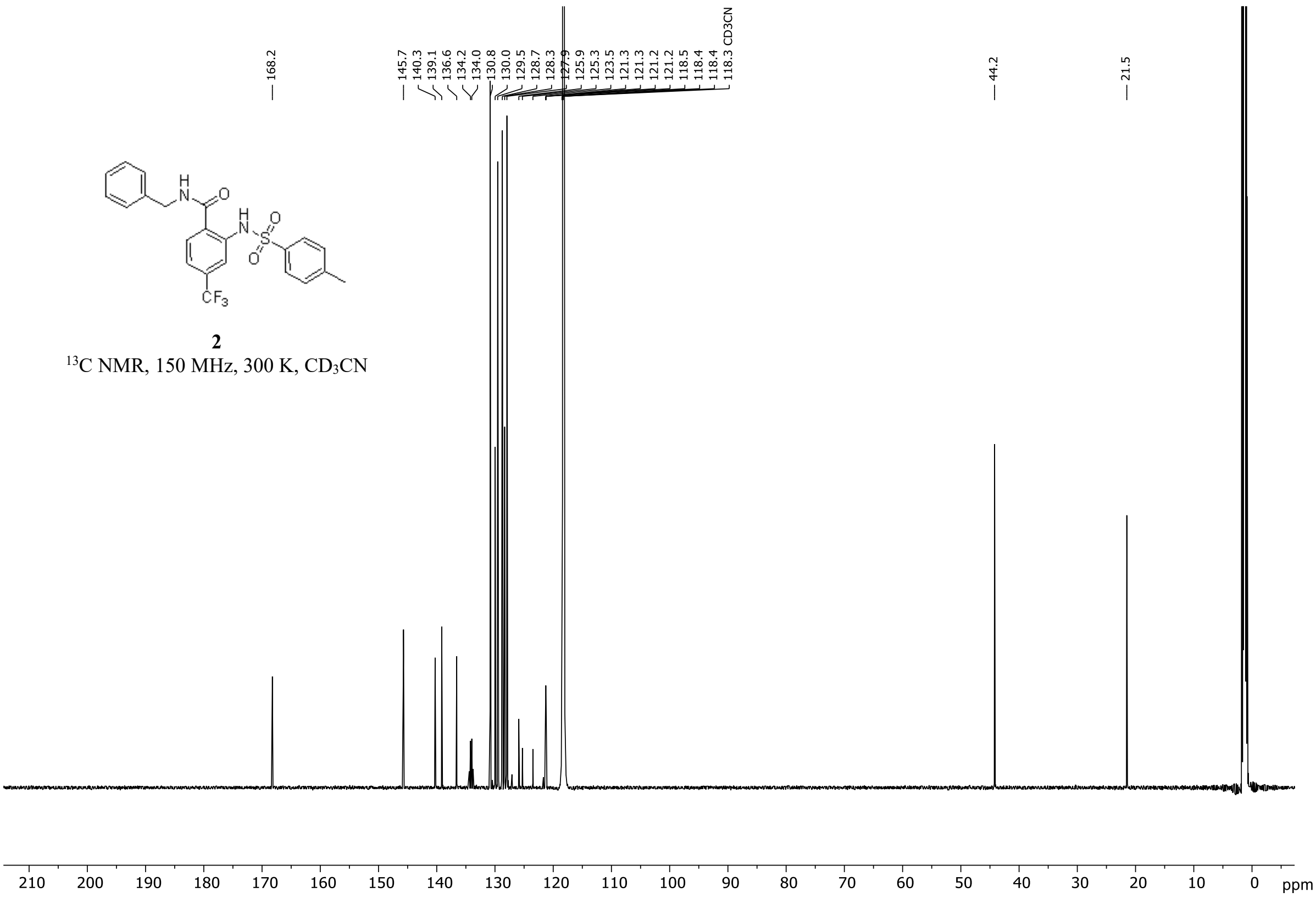

2

^{13}C NMR, 150 MHz, 300 K, CD_3CN

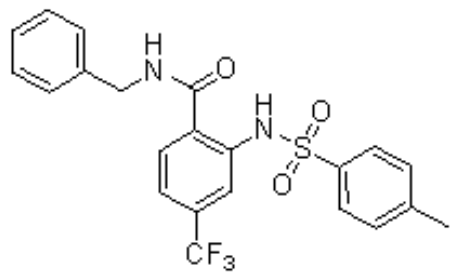

2

^{19}F NMR, 565 MHz, 300 K, CD_3CN

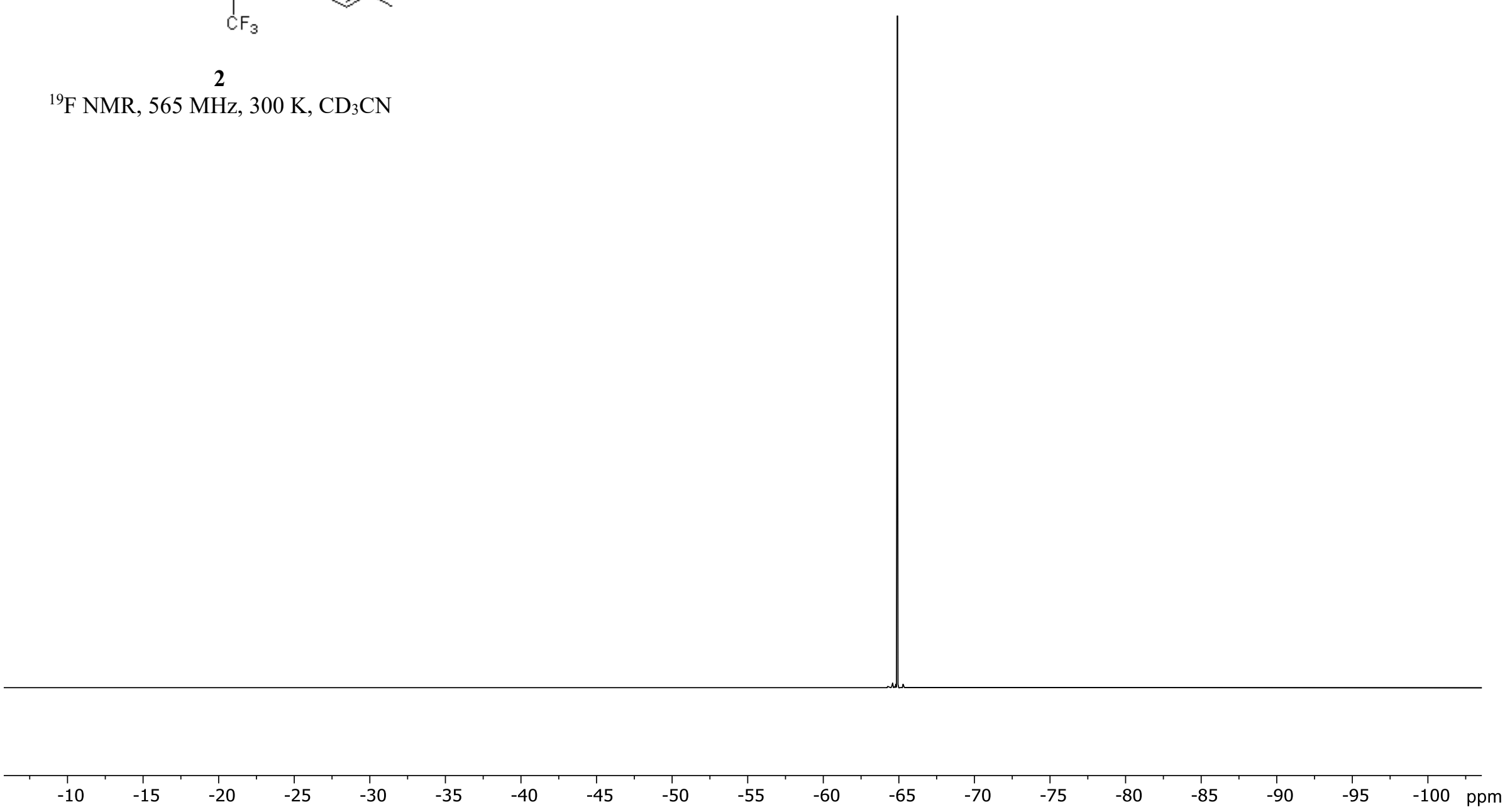

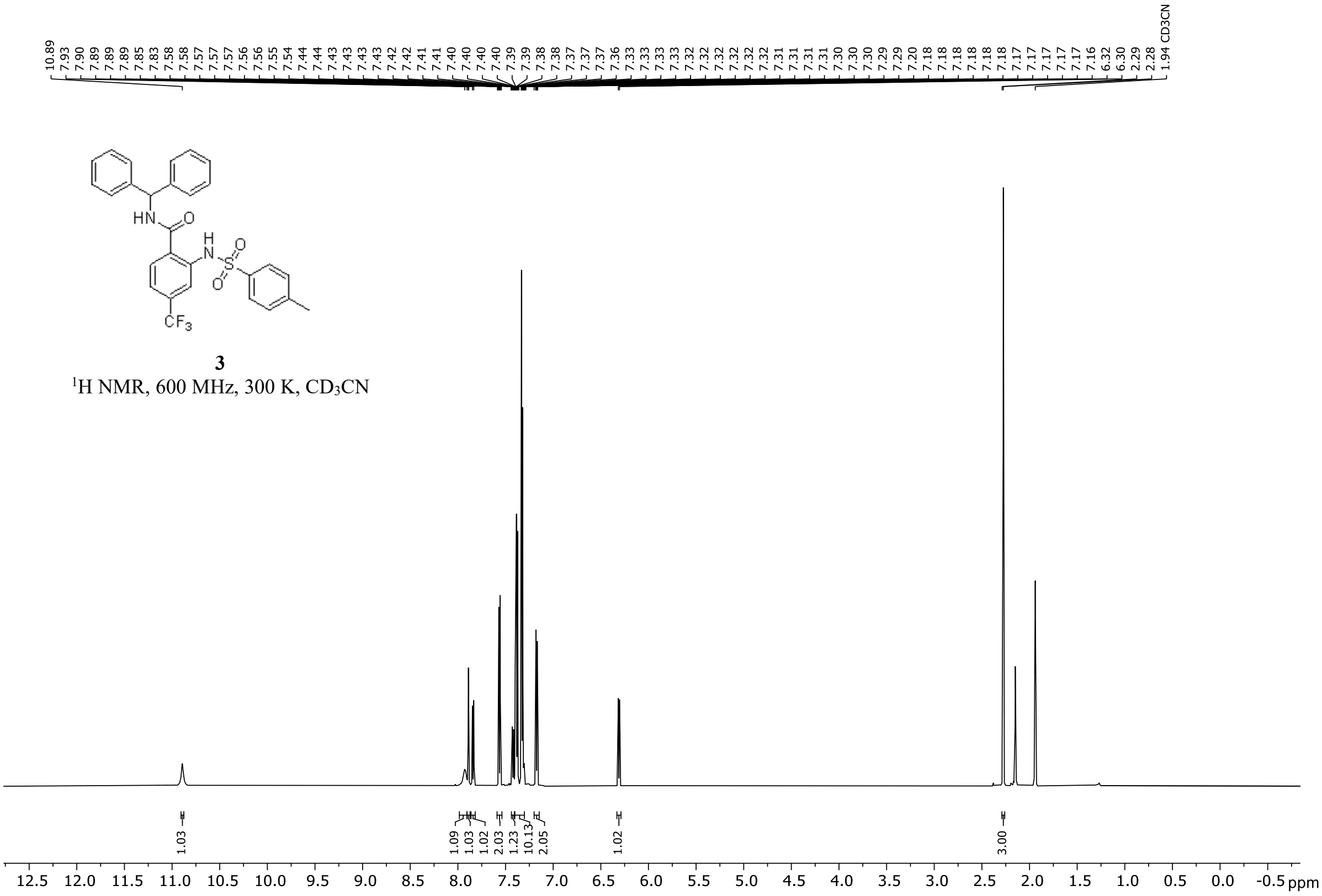

3
^{1}H NMR, 600 MHz, 300 K, CD_3CN

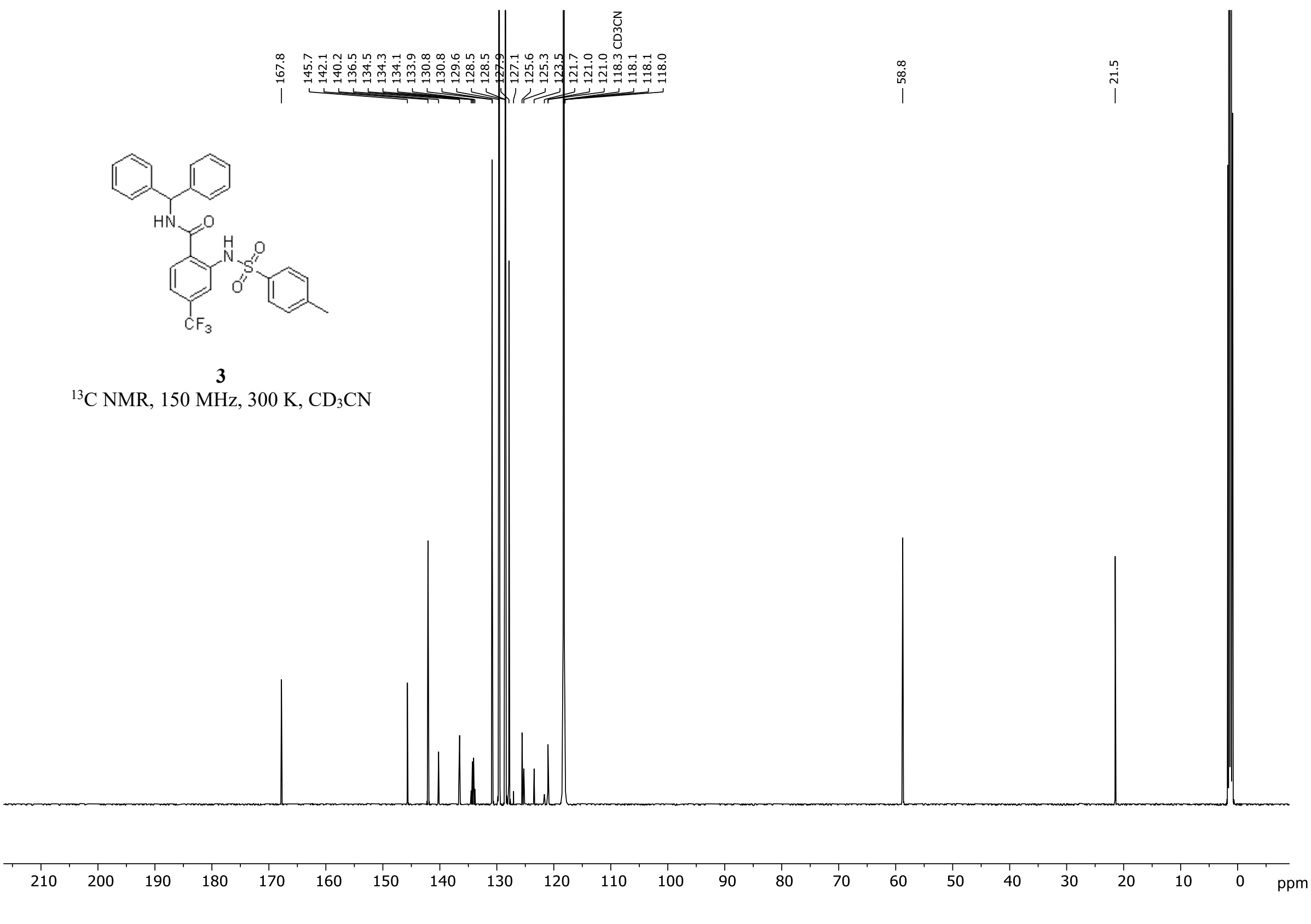

3
^{13}C NMR, 150 MHz, 300 K, CD_3CN

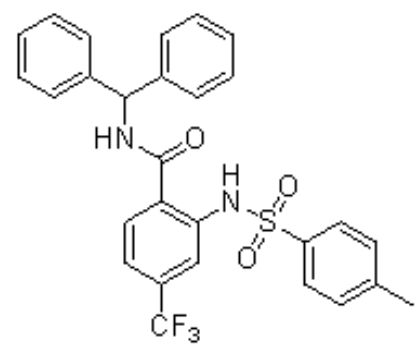

3
^{19}F NMR, 565 MHz, 300 K, CD_3CN

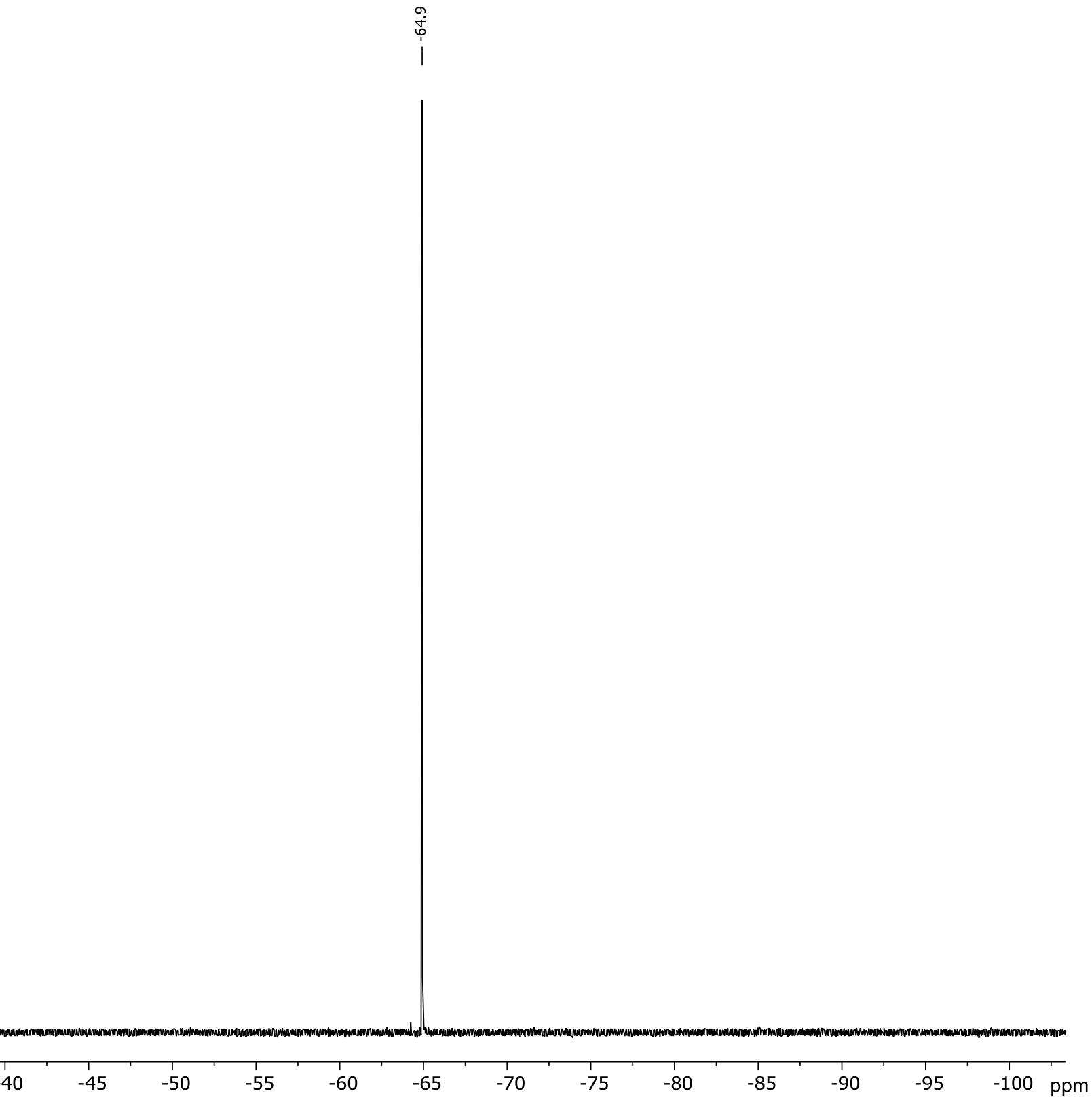

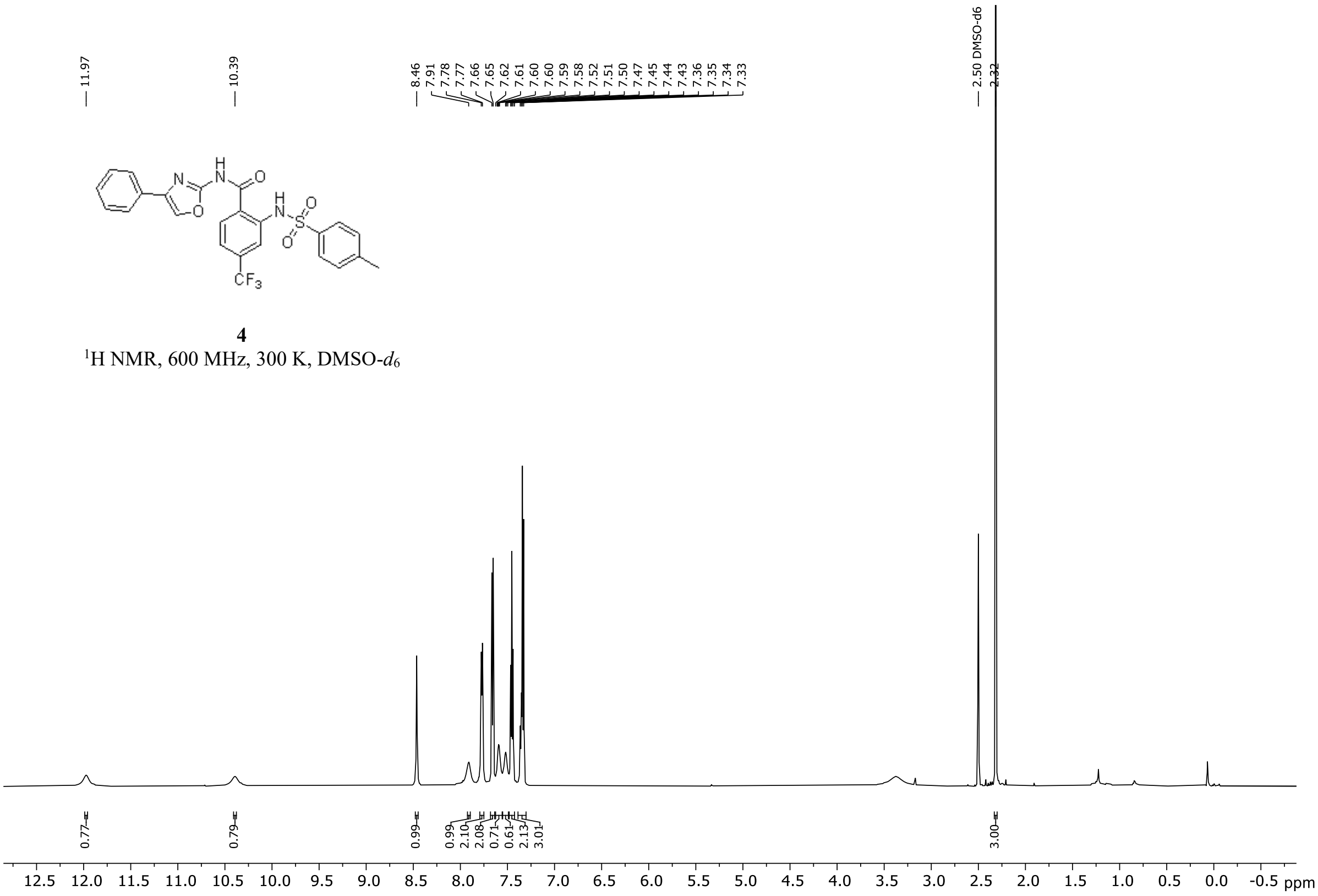

4

^{1}H NMR, 600 MHz, 300 K, DMSO-d_6

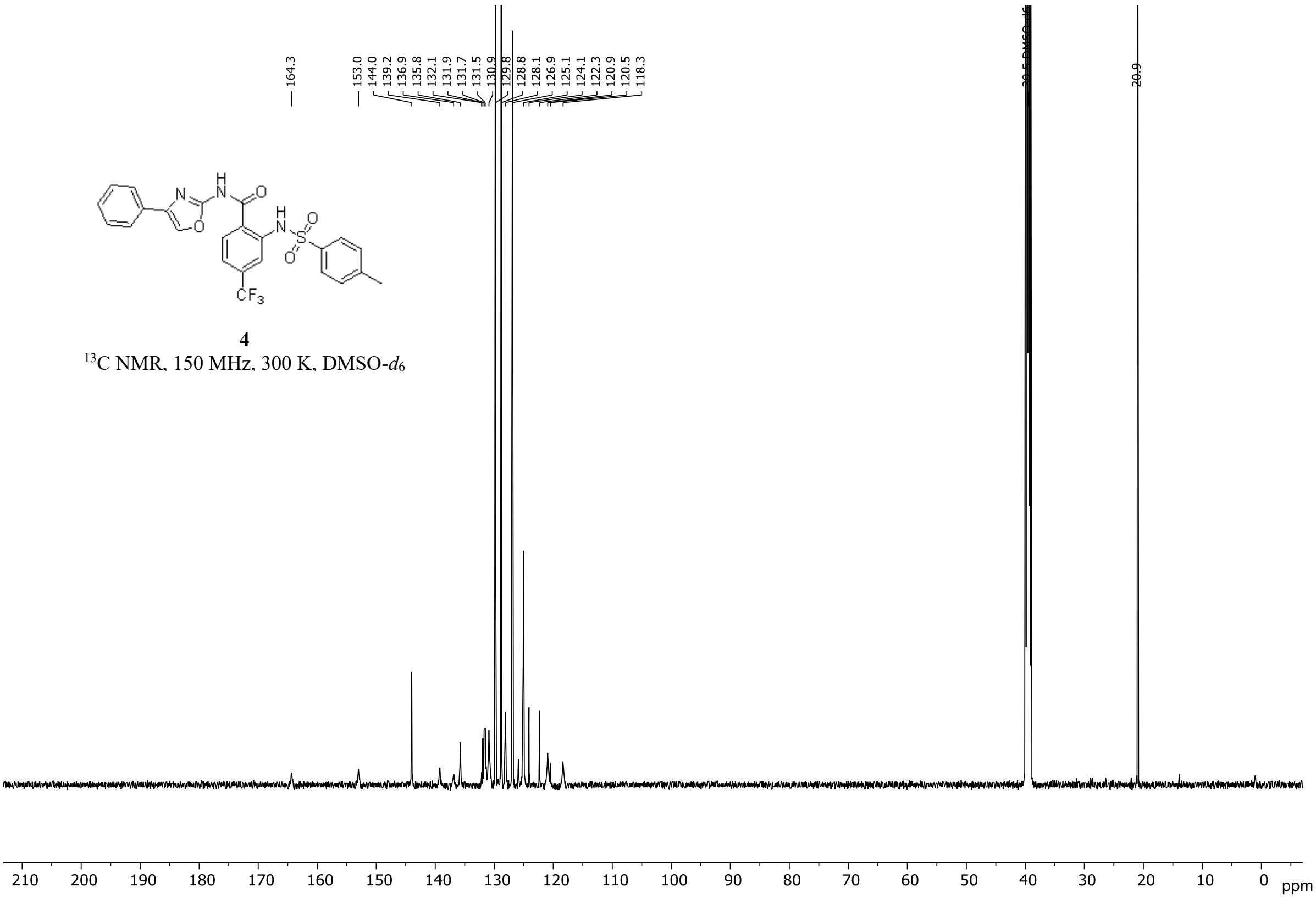

4

^{13}C NMR, 150 MHz, 300 K, DMSO-d_6

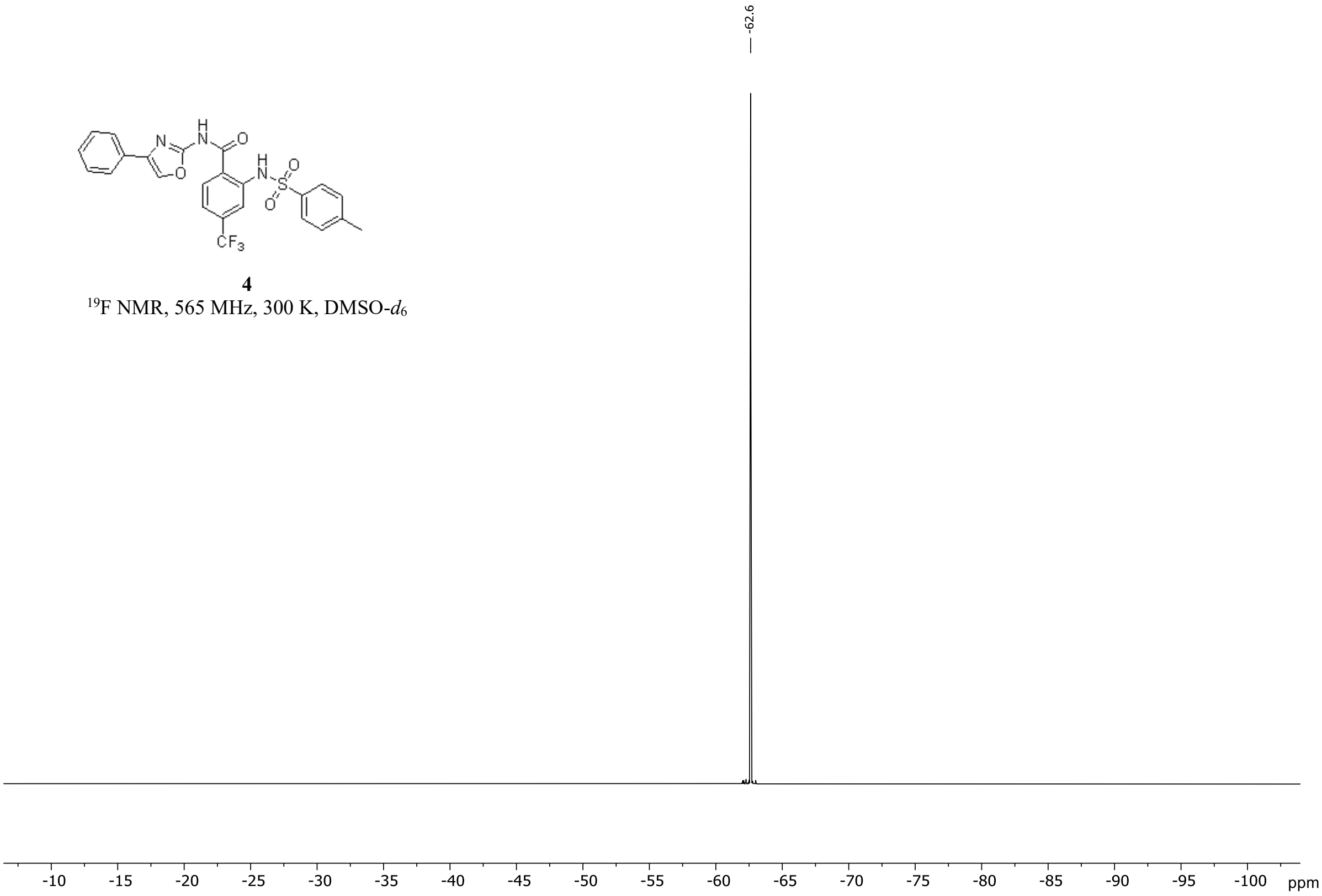

4

^{19}F NMR, 565 MHz, 300 K, DMSO-d_6

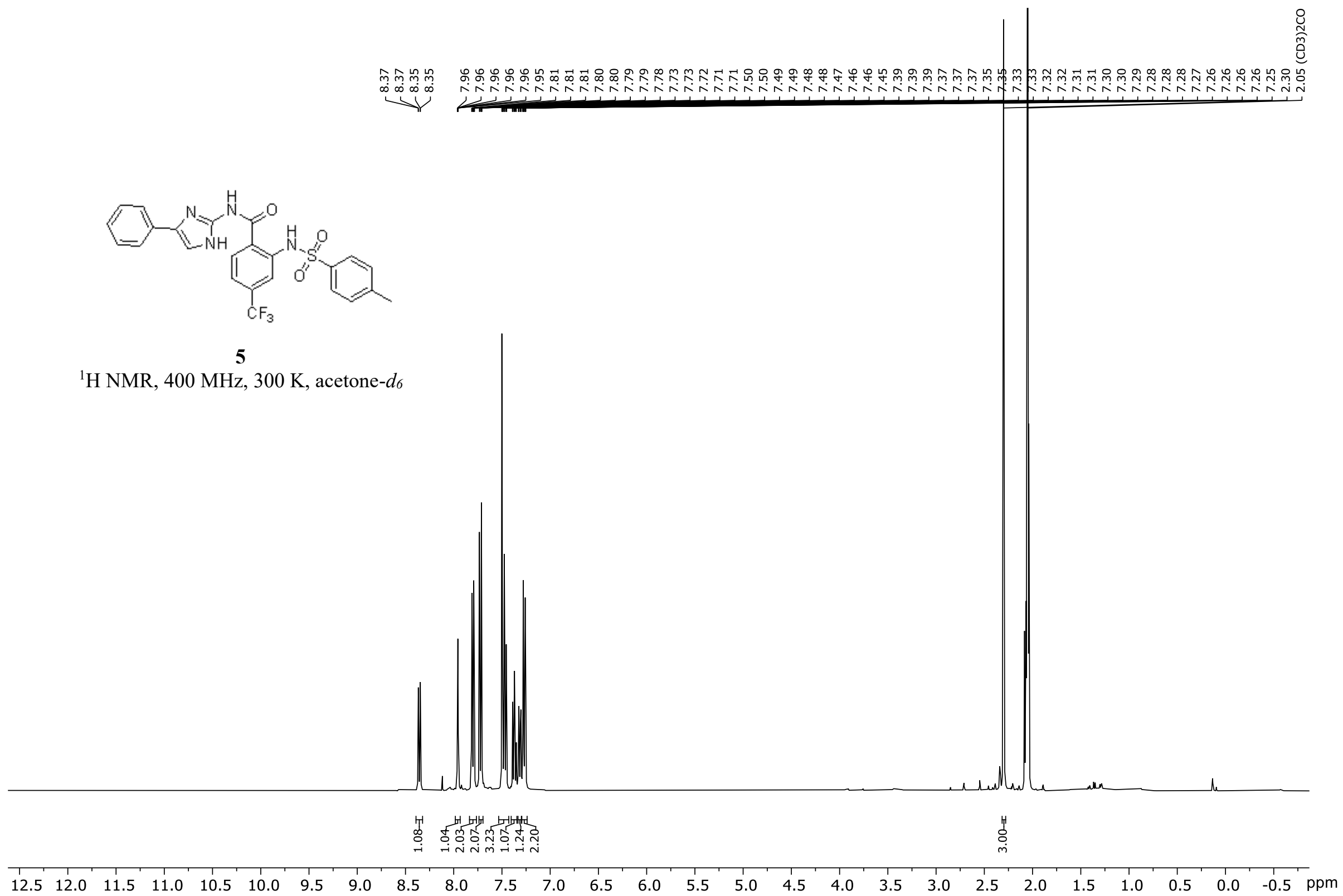

5

^{1}H NMR, 400 MHz, 300 K, acetone-d_6

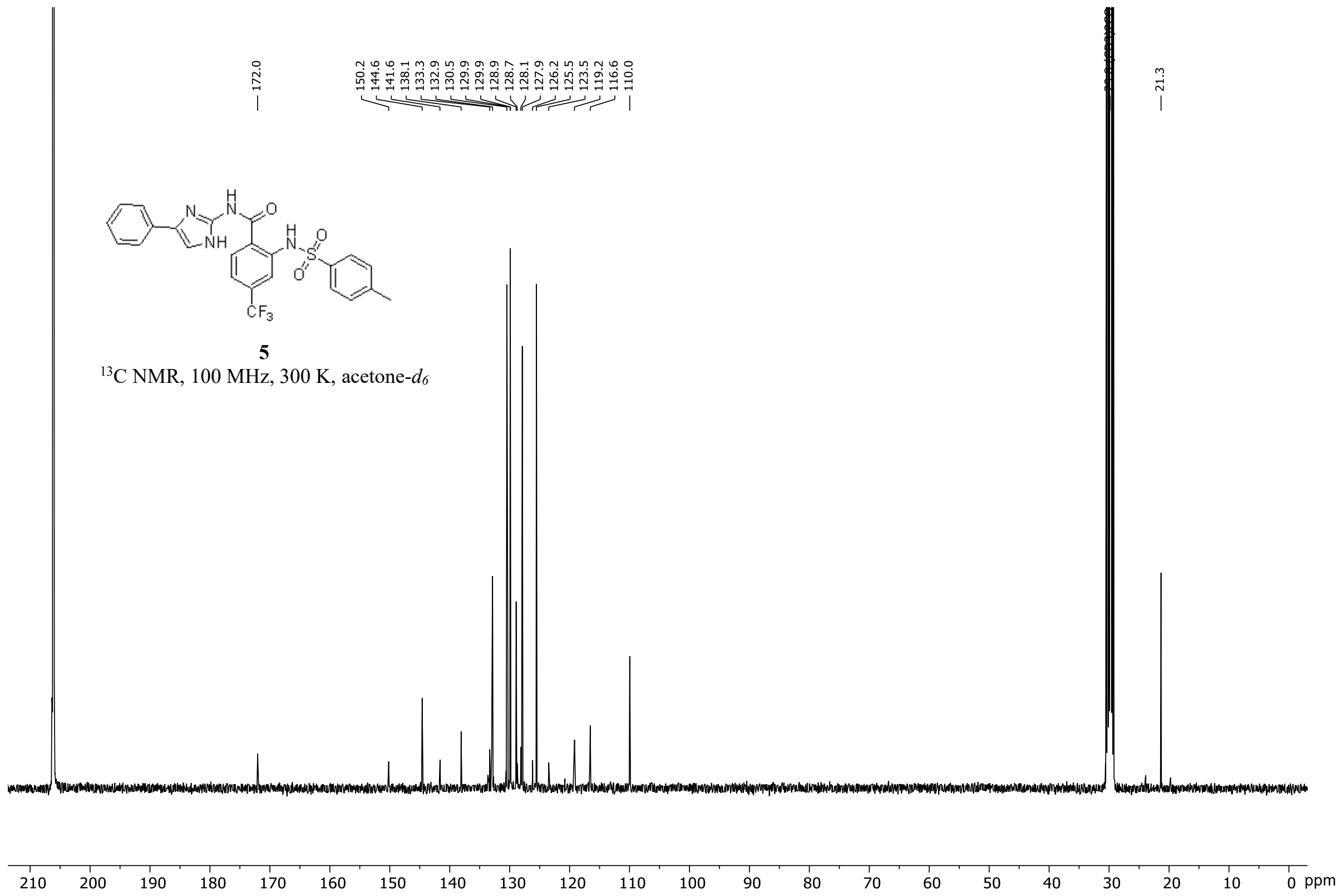

5

^{13}C NMR, 100 MHz, 300 K, acetone-d_6

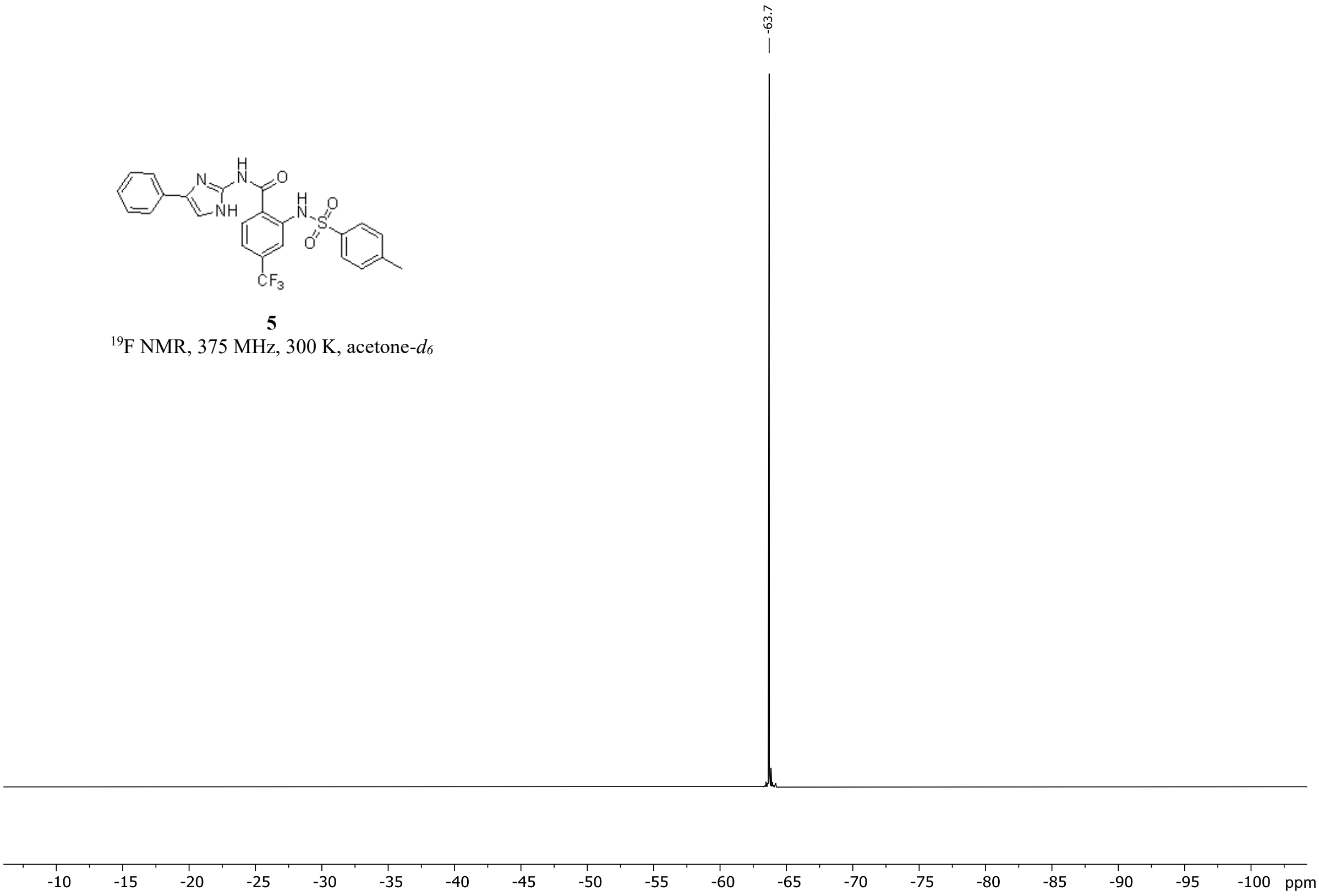

5

^{19}F NMR, 375 MHz, 300 K, acetone-d_6

6
^{1}H NMR, 400 MHz, 300 K, $CDCl_3$

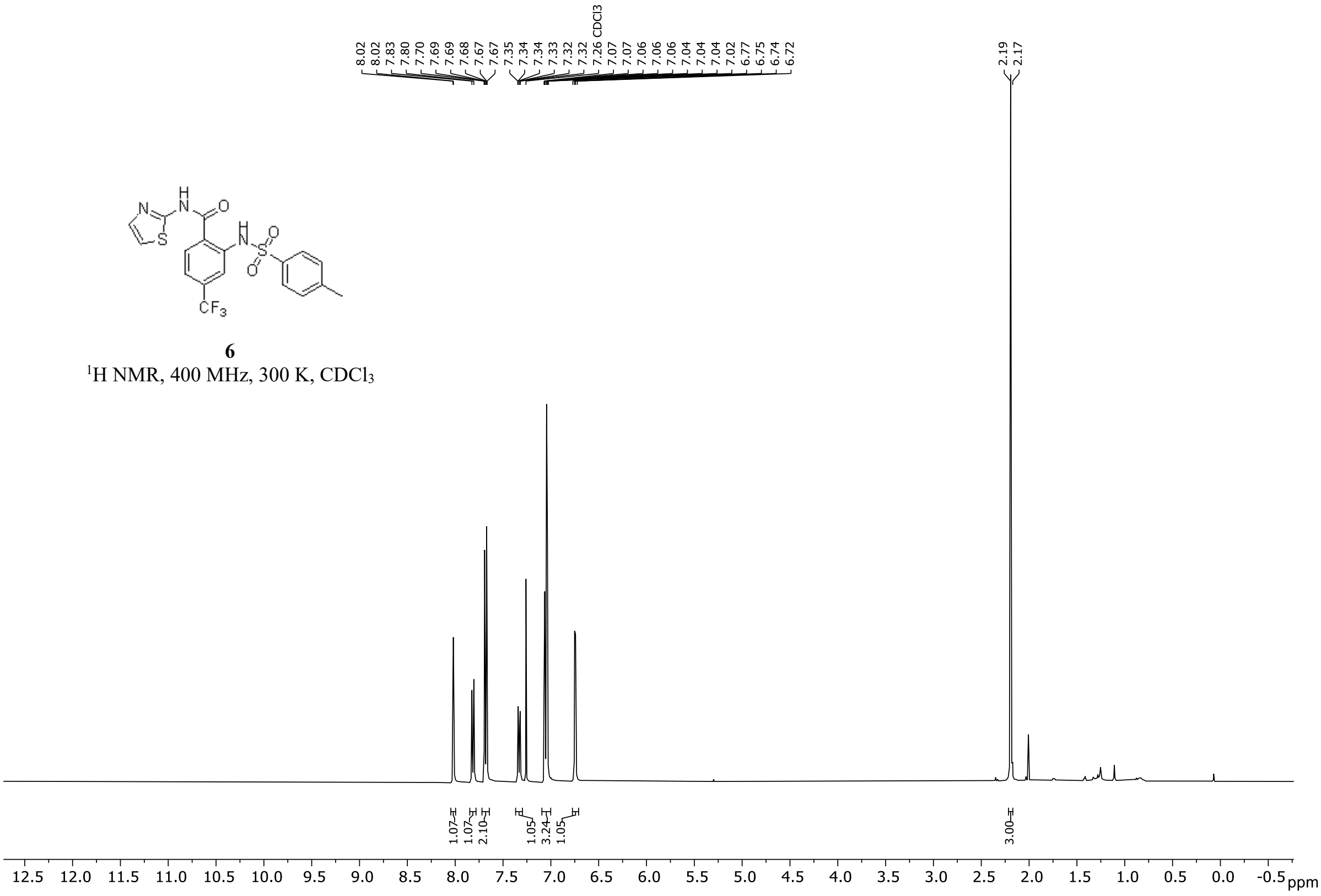

6
^{13}C NMR, 100 MHz, 300 K, $CDCl_3$

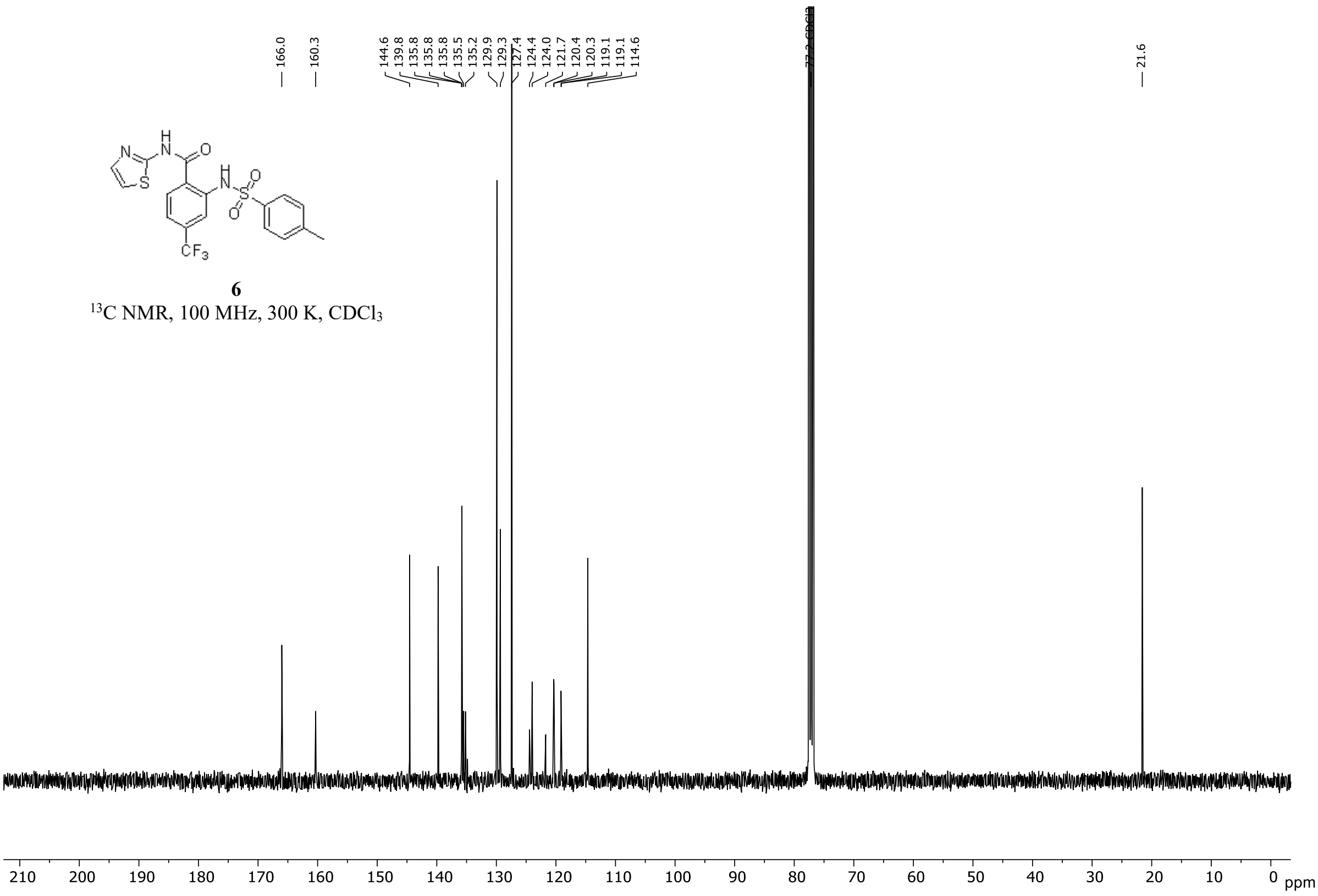

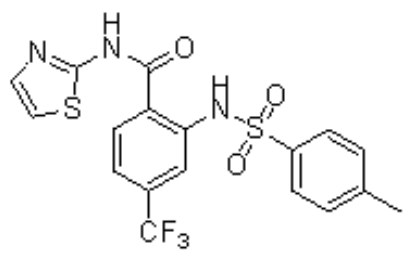

6
^{19}F NMR, 375 MHz, 300 K, $CDCl_3$

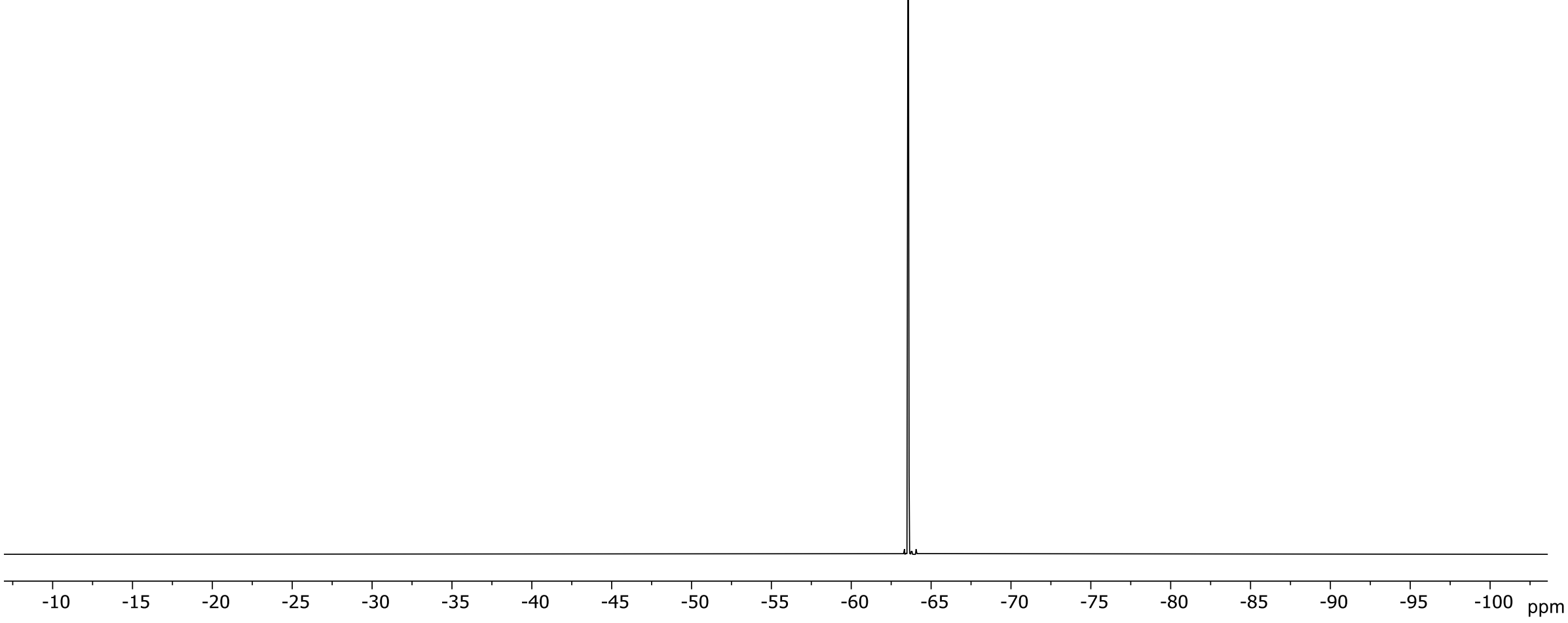

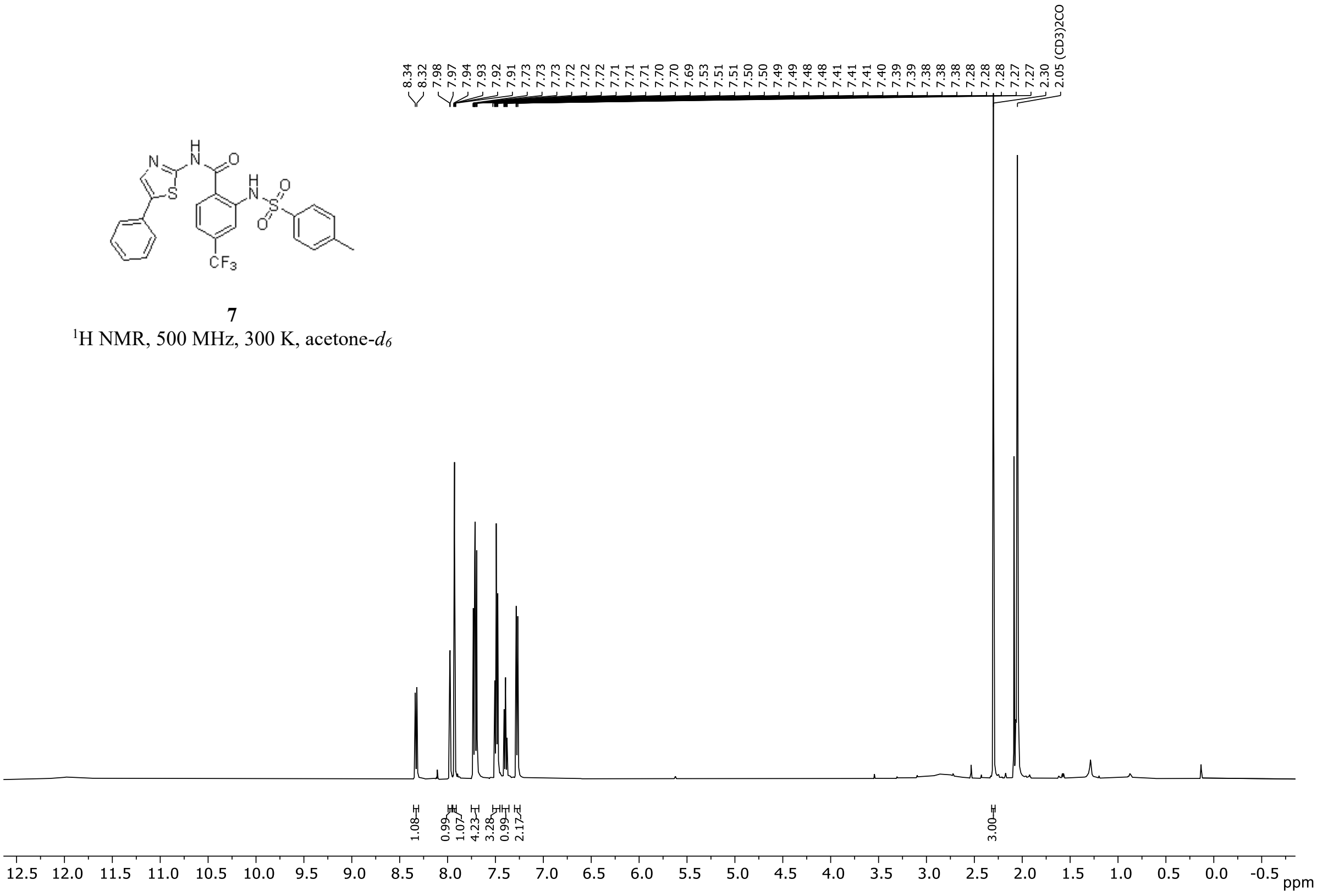

7
^{1}H NMR, 500 MHz, 300 K, acetone-d_6

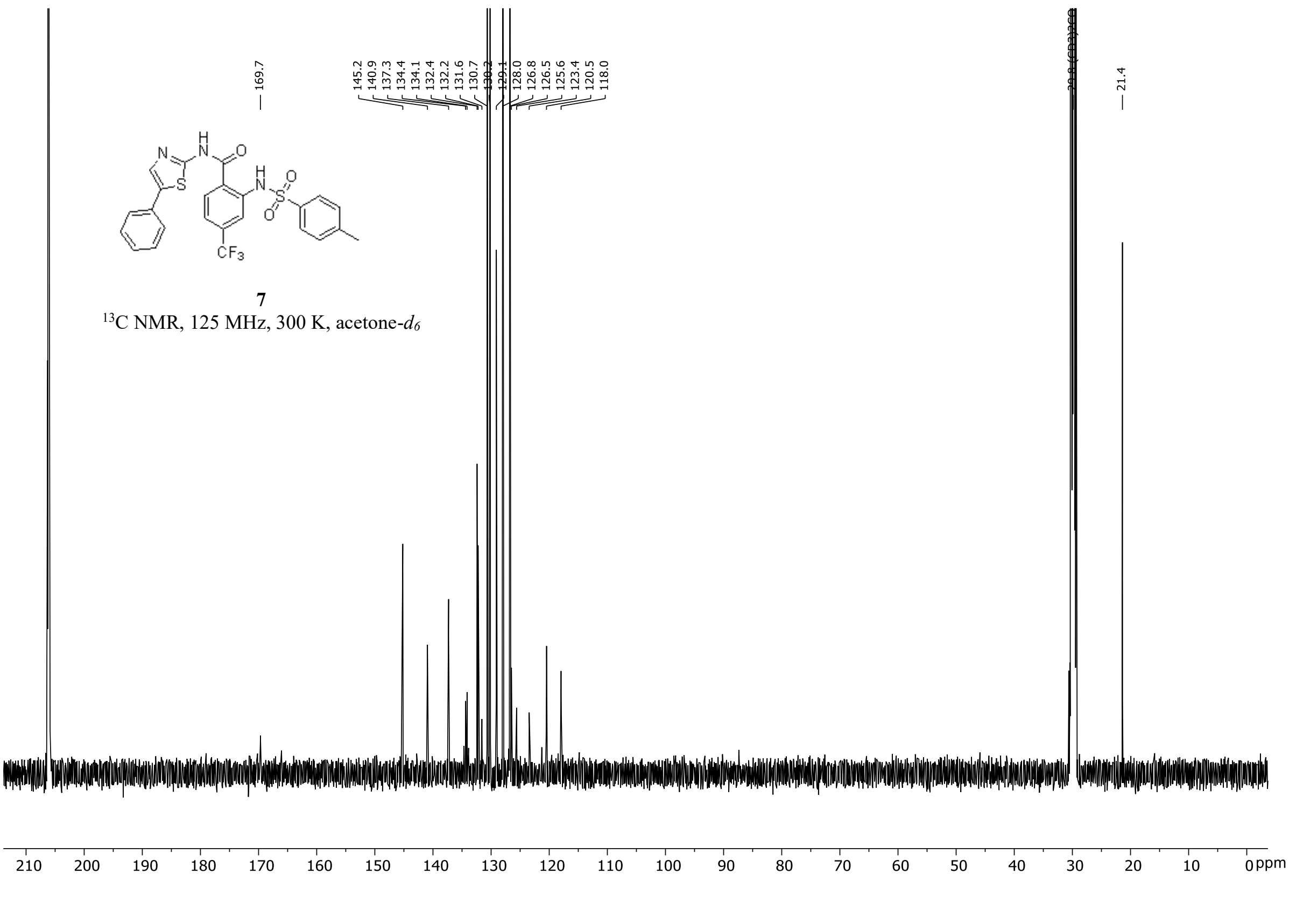

7
^{13}C NMR, 125 MHz, 300 K, acetone-d_6

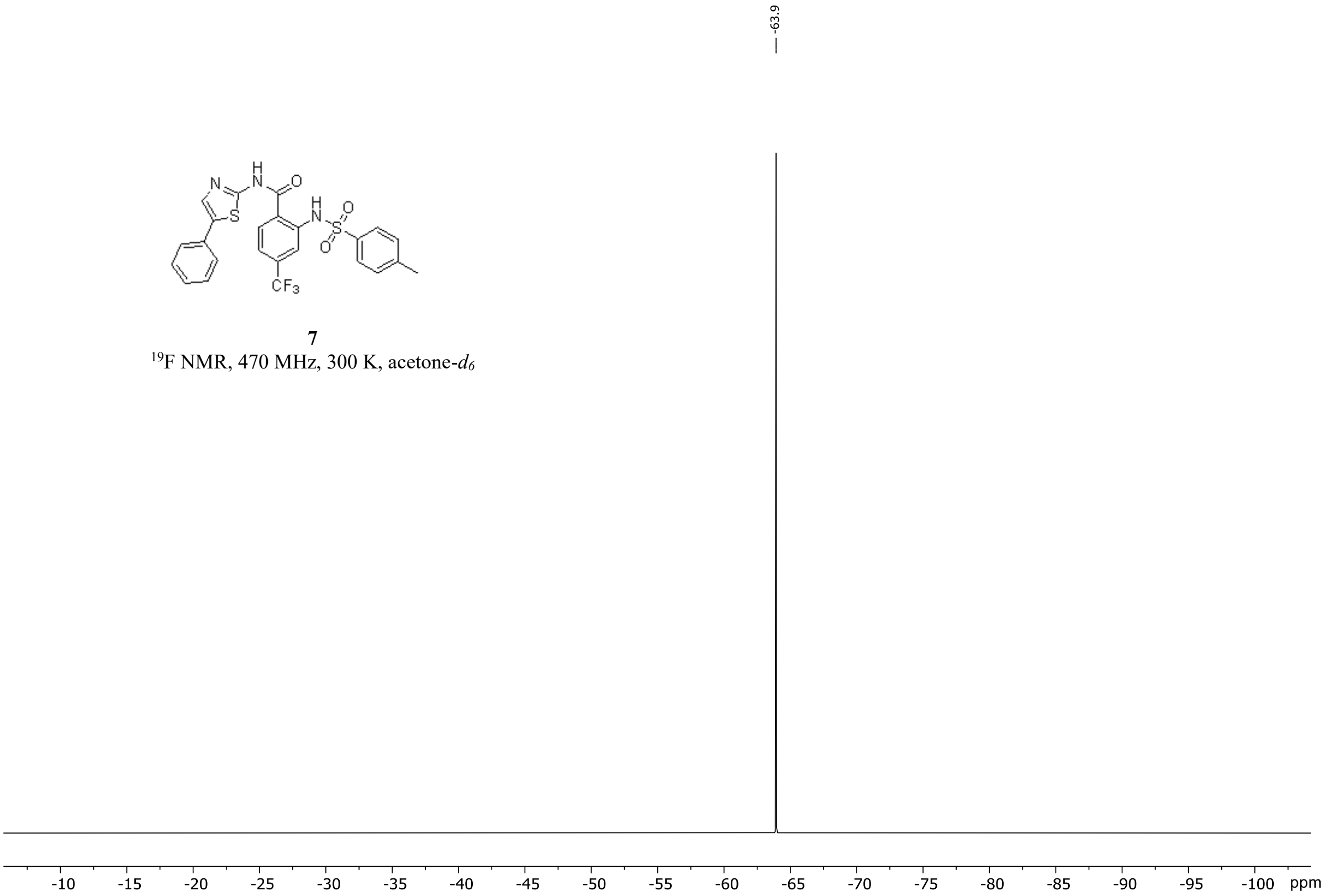

7

^{19}F NMR, 470 MHz, 300 K, acetone-d_6

8
^1H NMR, 600 MHz, 300 K, acetone-d_6

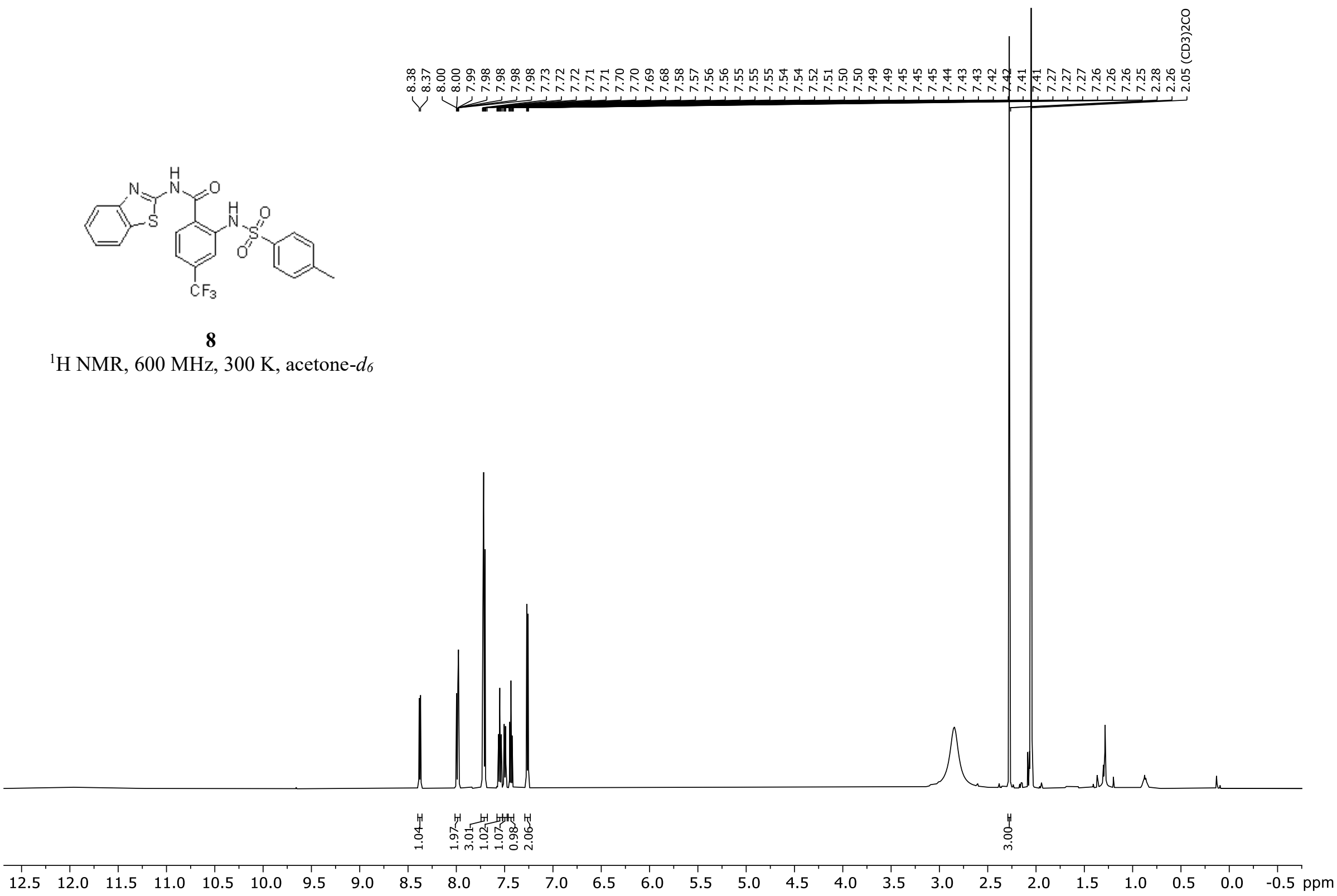

8
^{13}C NMR, 150 MHz, 300 K, acetone-d_6

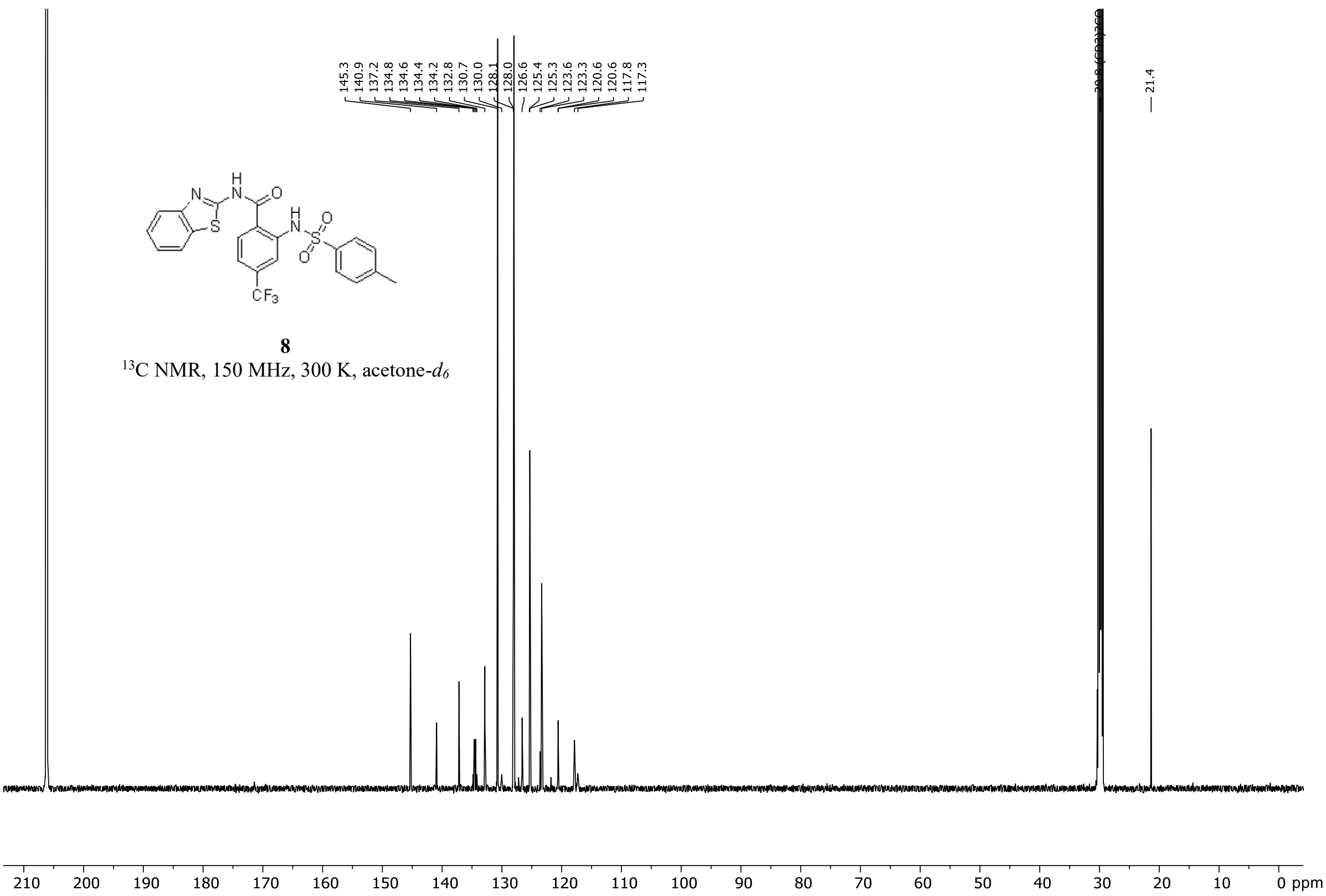

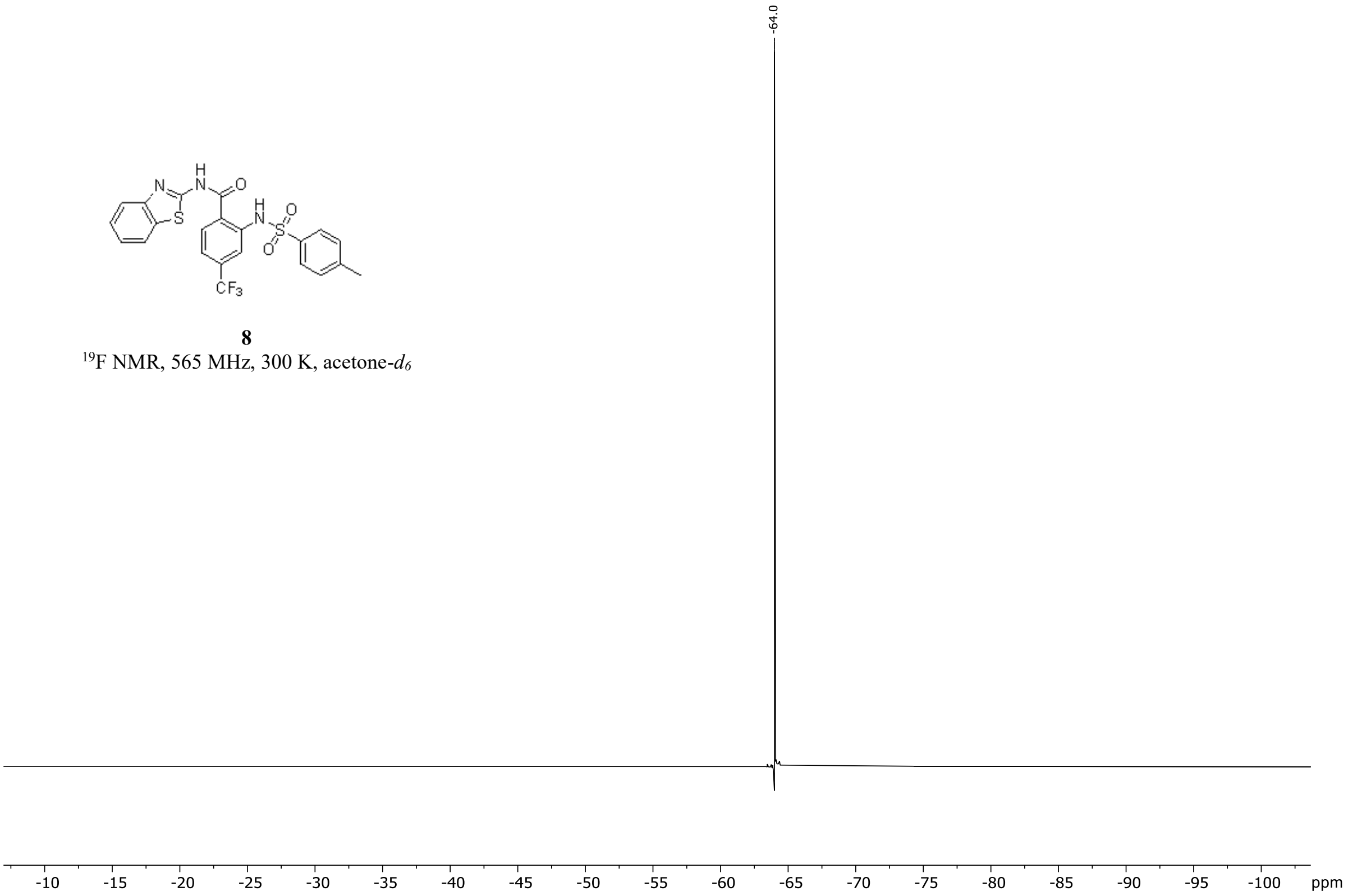

8

^{19}F NMR, 565 MHz, 300 K, acetone-d_6

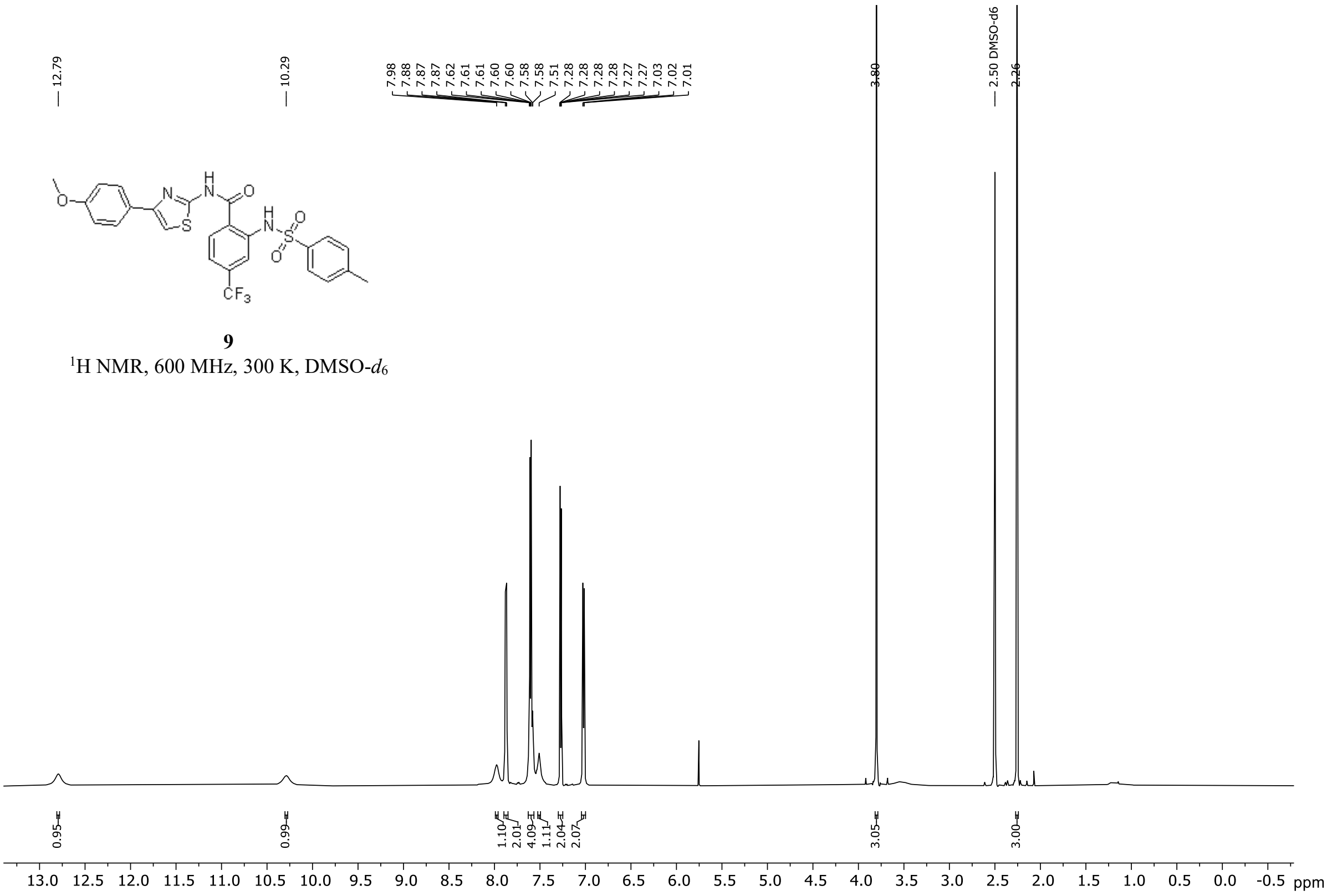

9

^{1}H NMR, 600 MHz, 300 K, DMSO-d_6

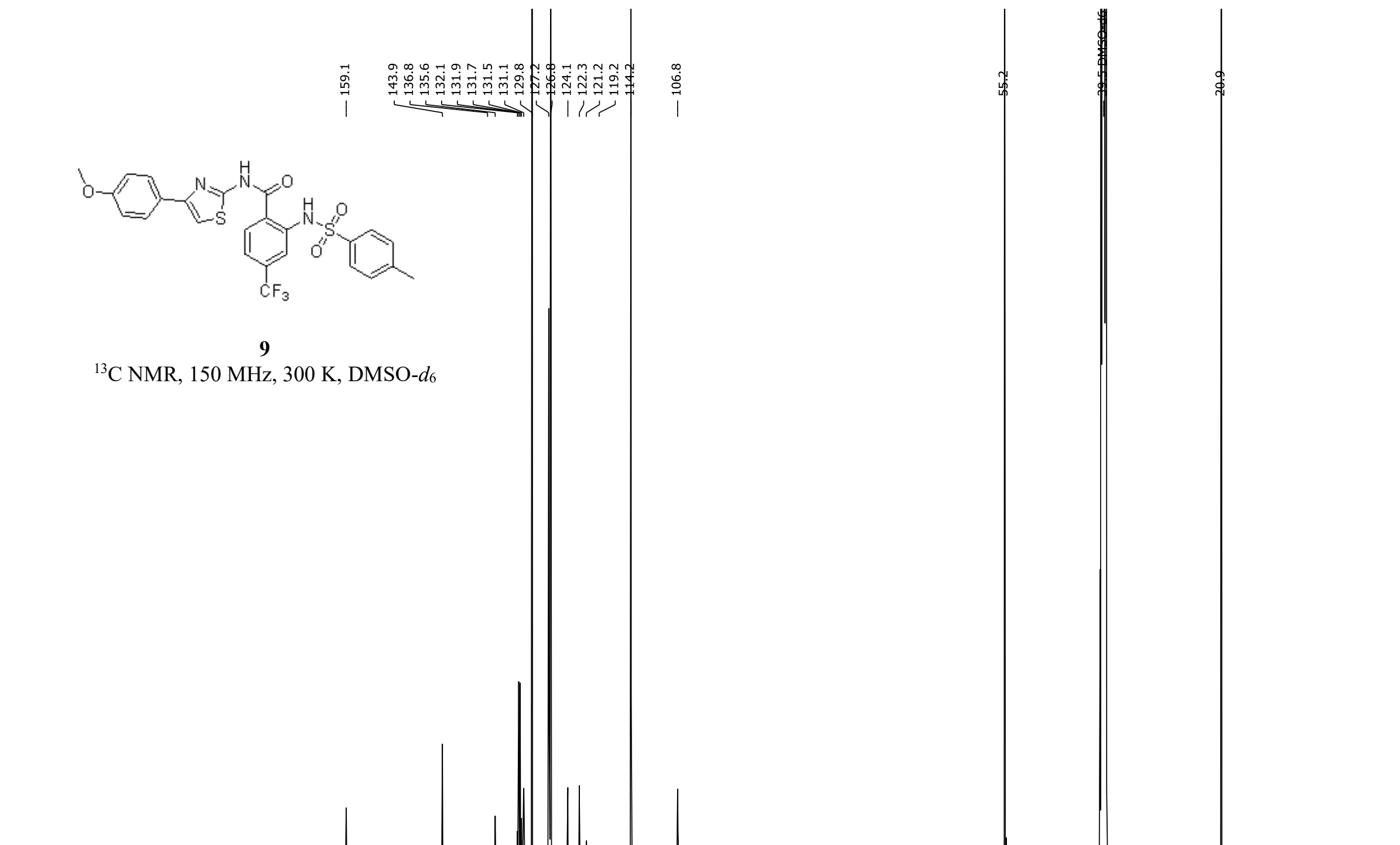

9

^{13}C NMR, 150 MHz, 300 K, DMSO-d_6

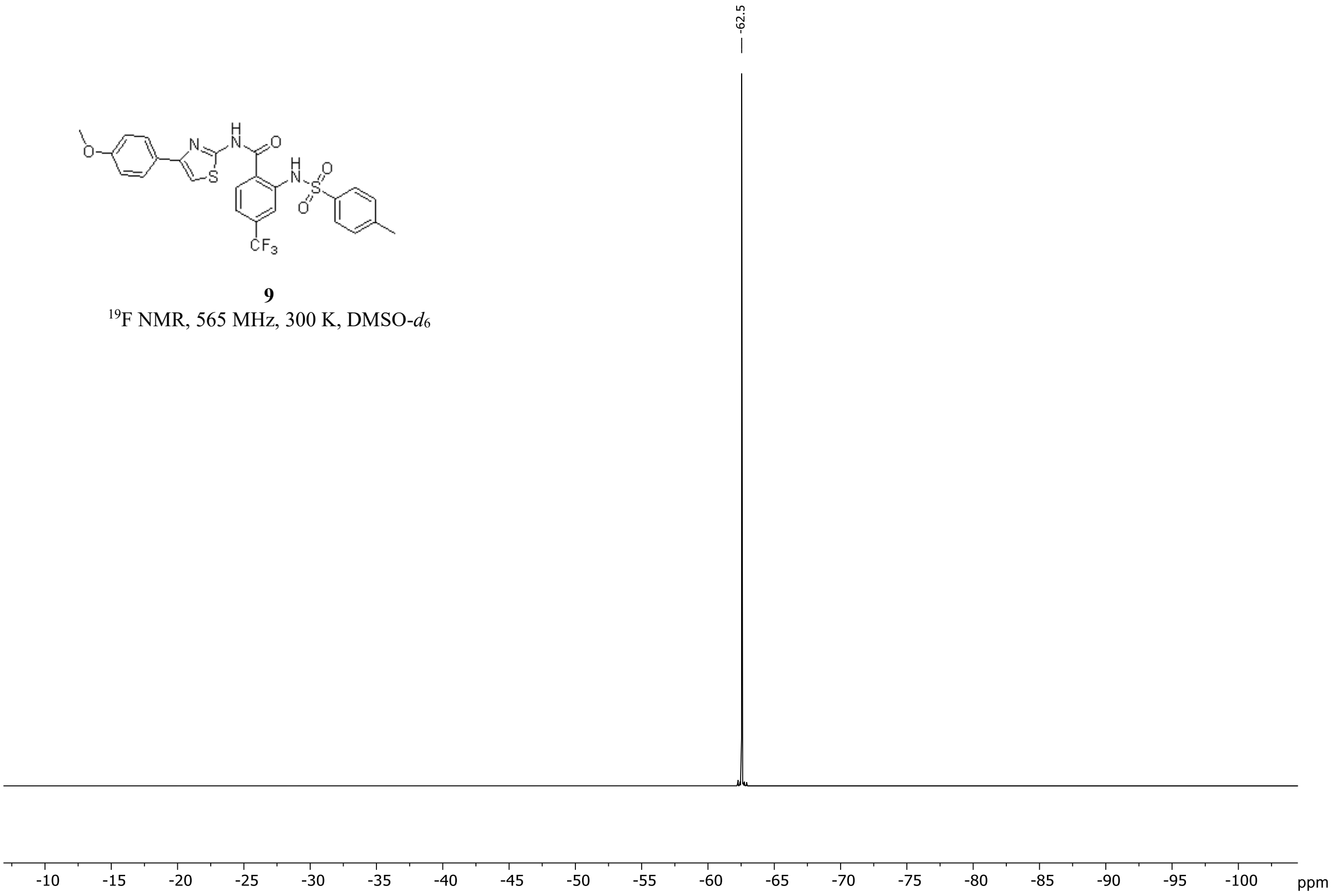

9

^{19}F NMR, 565 MHz, 300 K, DMSO-d_6

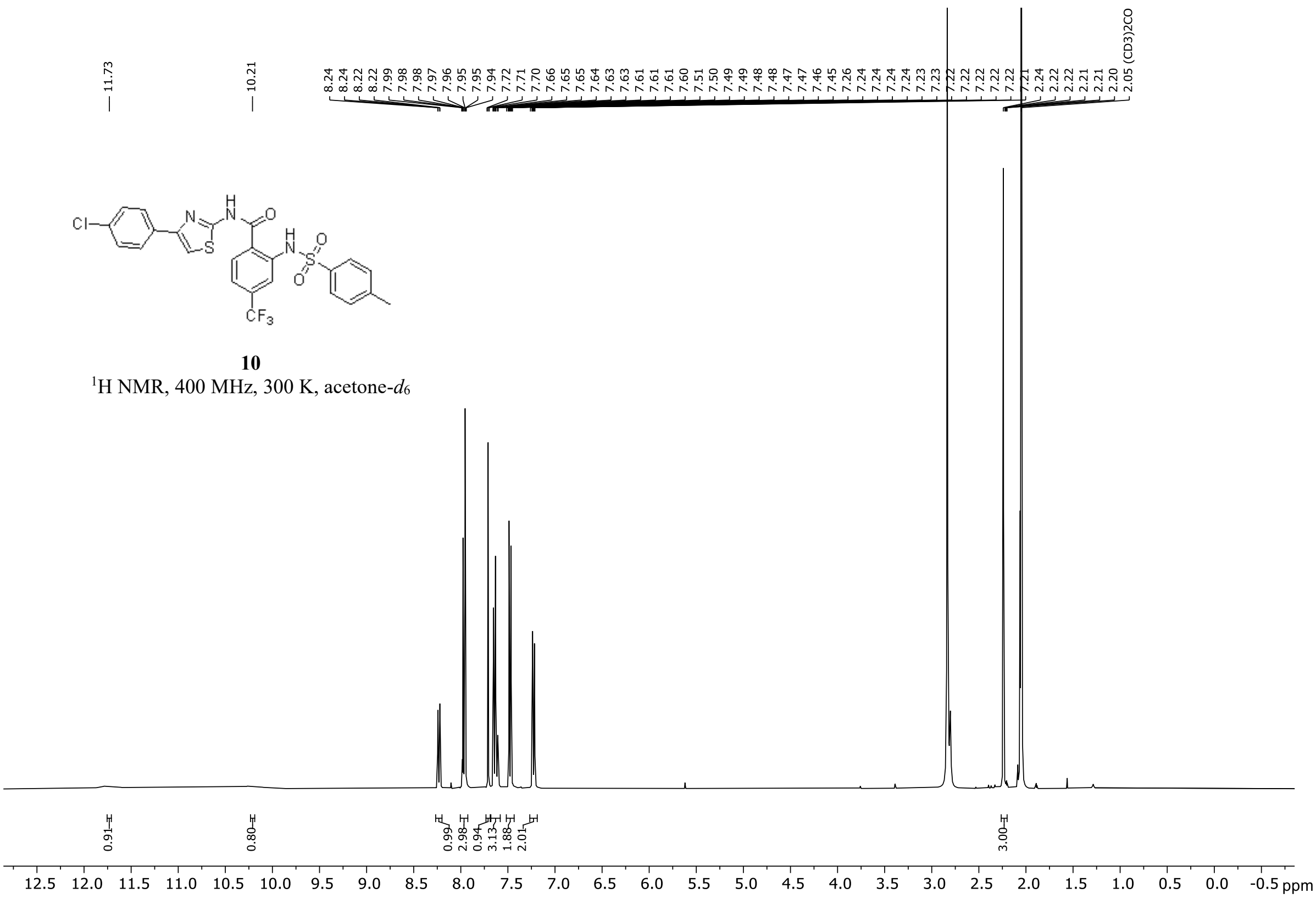

10
^{1}H NMR, 400 MHz, 300 K, acetone-d_6

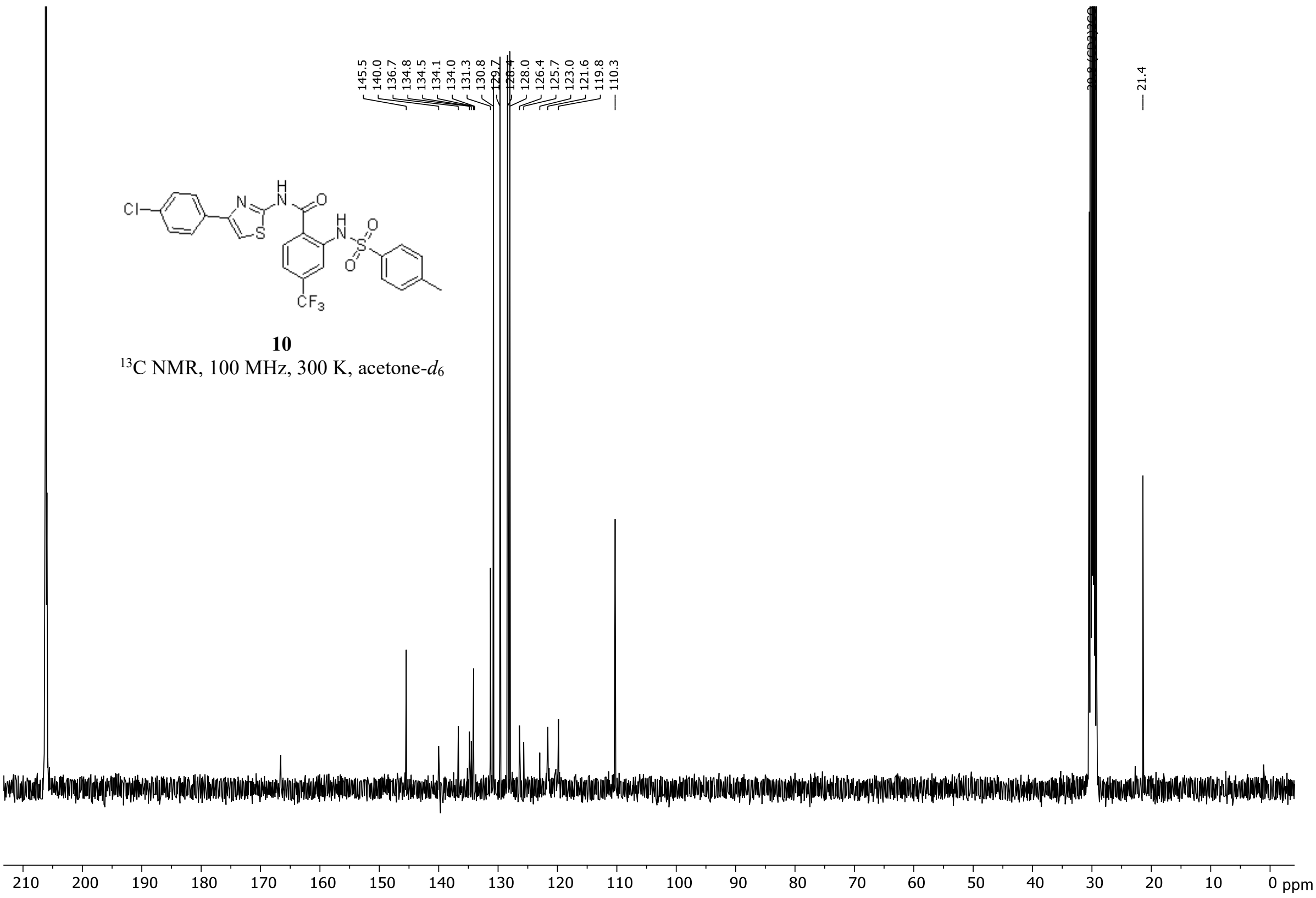

10
^{13}C NMR, 100 MHz, 300 K, acetone-d_6

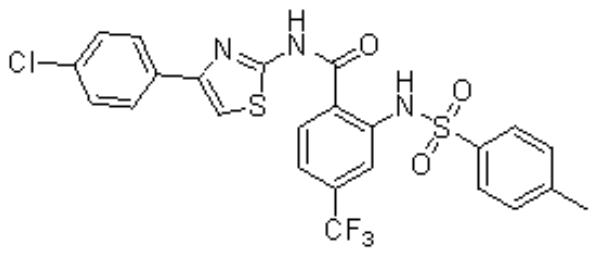

10

^{19}F NMR, 375 MHz, 300 K, acetone-d_6

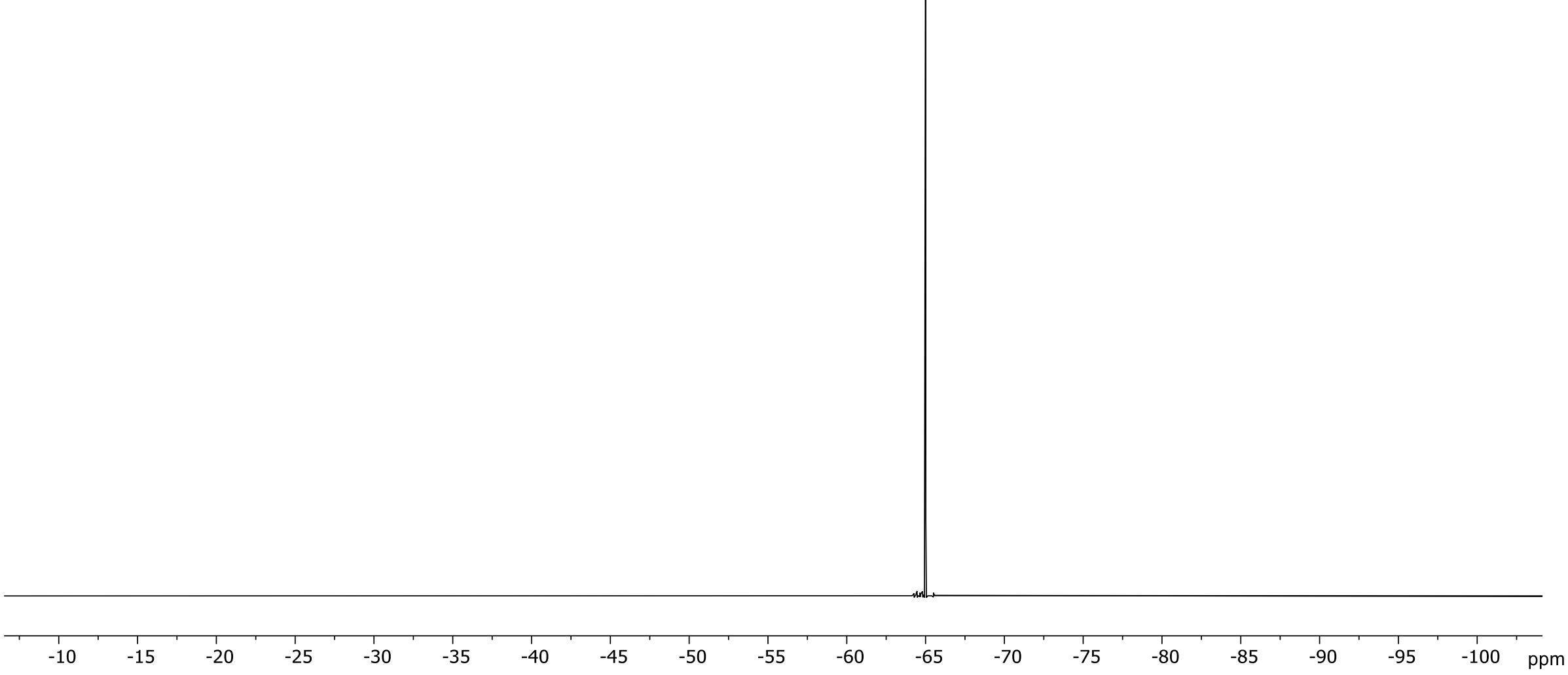

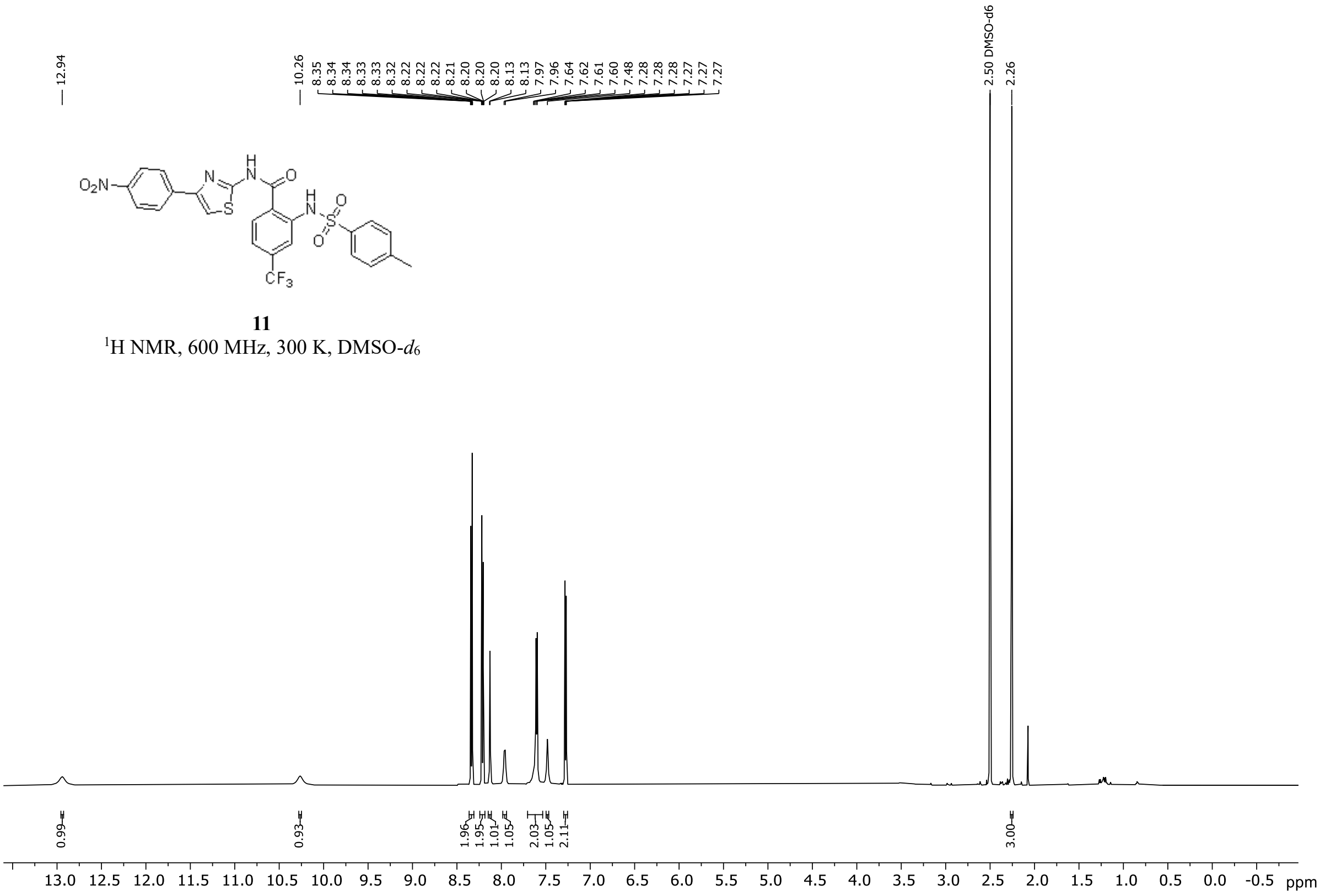

11

^{1}H NMR, 600 MHz, 300 K, DMSO-d_6

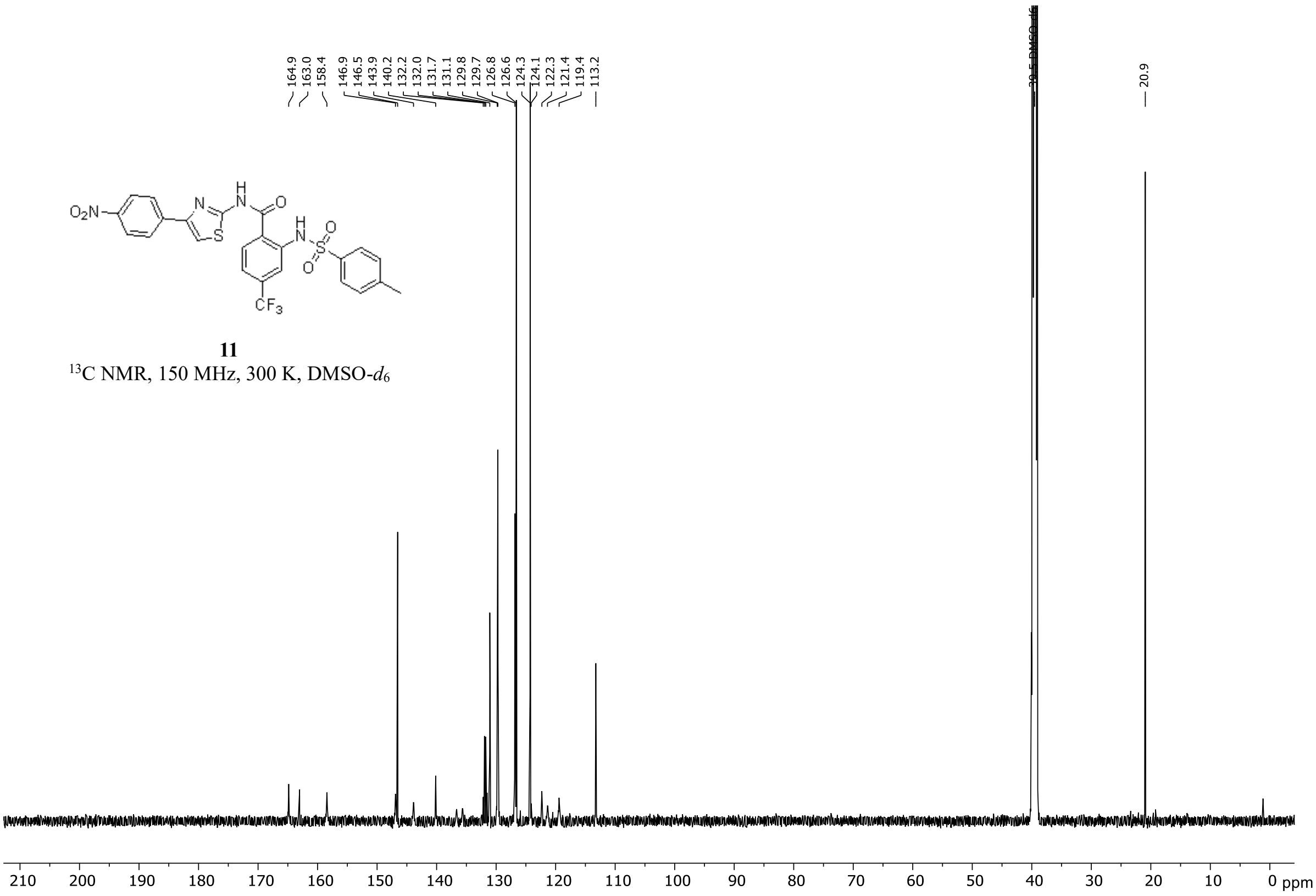

11

^{13}C NMR, 150 MHz, 300 K, DMSO-d_6

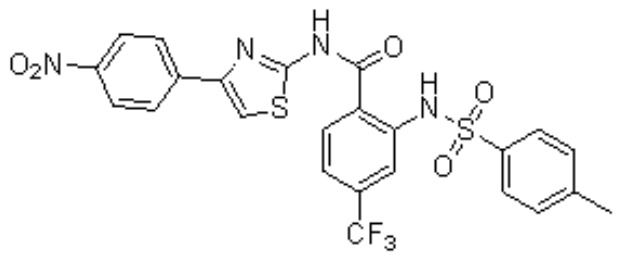

11
^{19}F NMR, 565 MHz, 300 K, DMSO-d_6

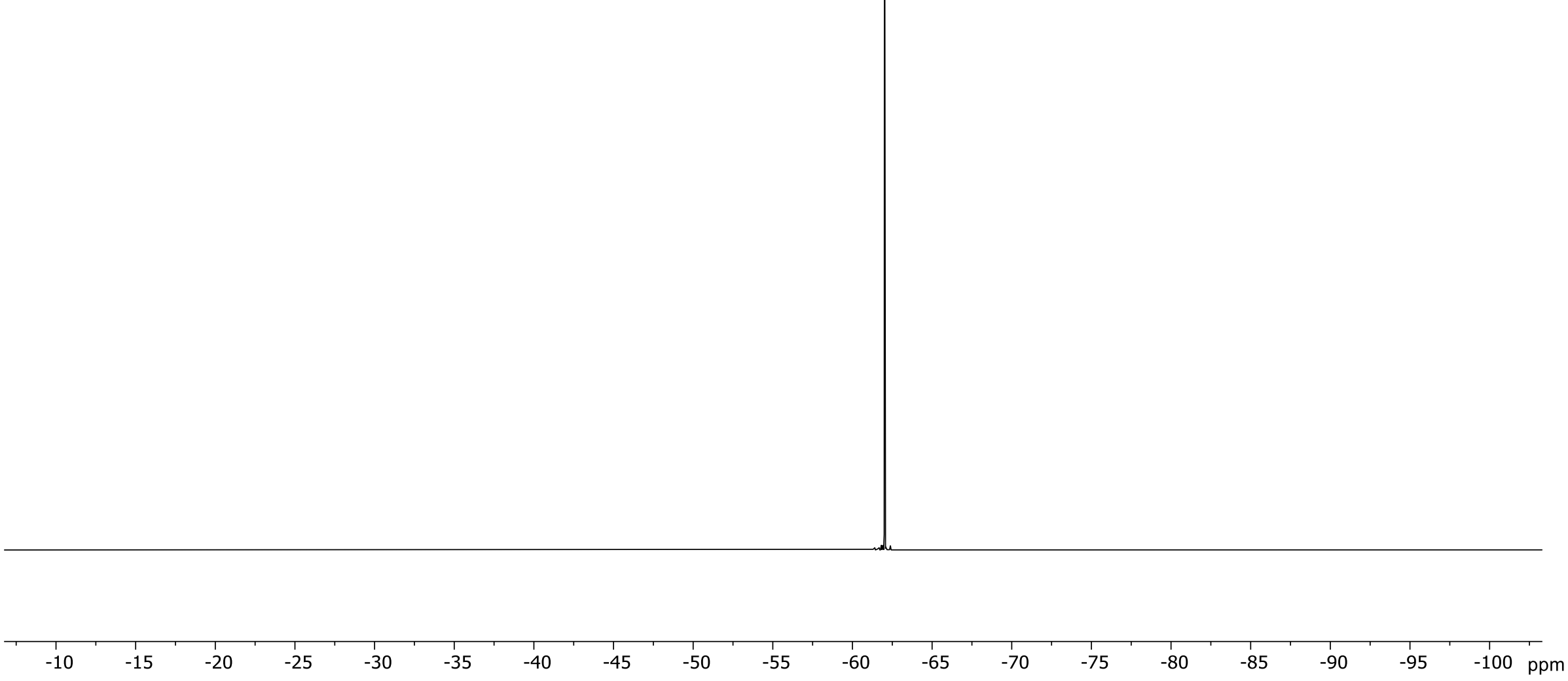

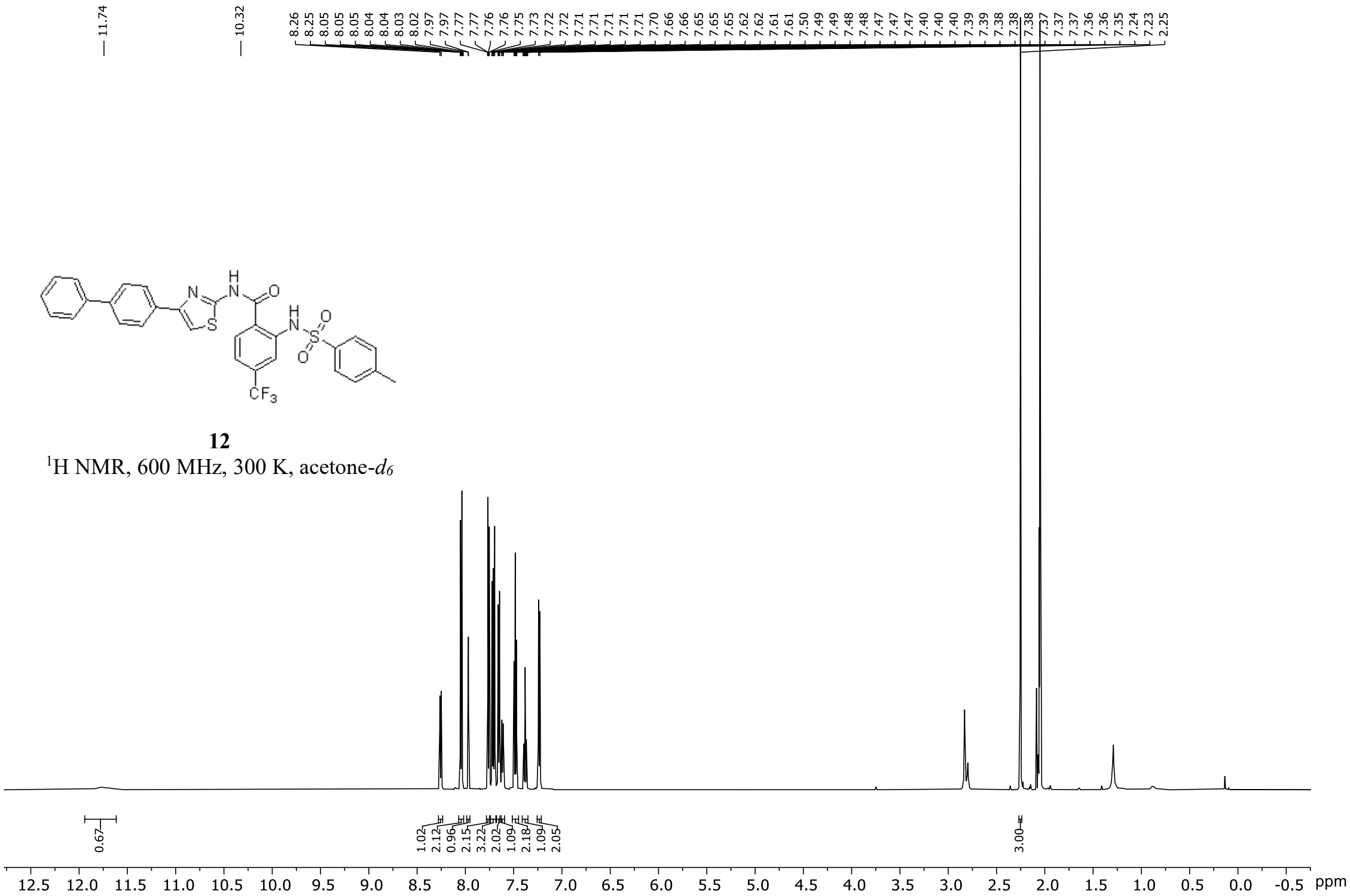

12
^{1}H NMR, 600 MHz, 300 K, acetone-d_6

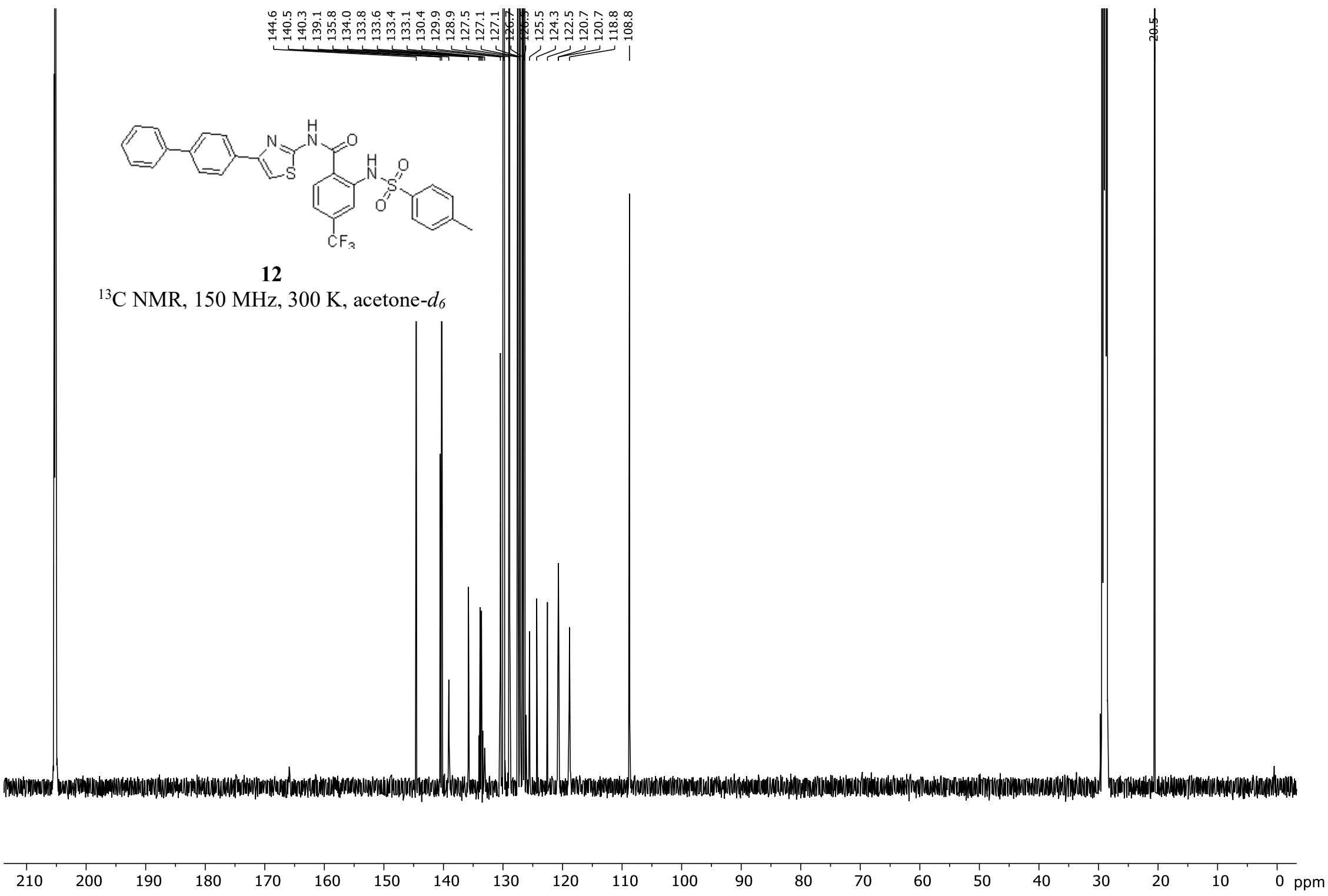

12
^{13}C NMR, 150 MHz, 300 K, acetone-d_6

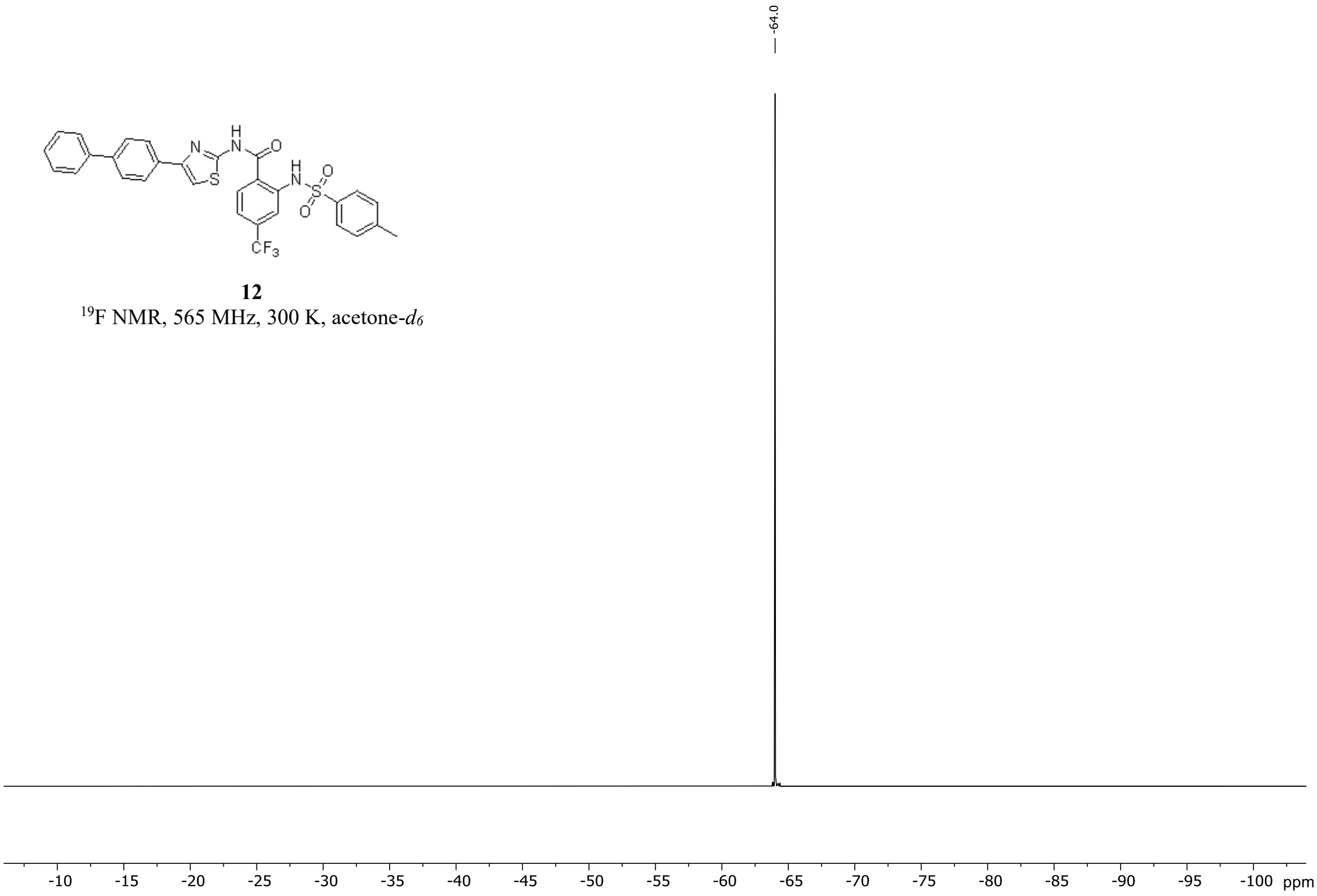

12
^{19}F NMR, 565 MHz, 300 K, acetone-d_6

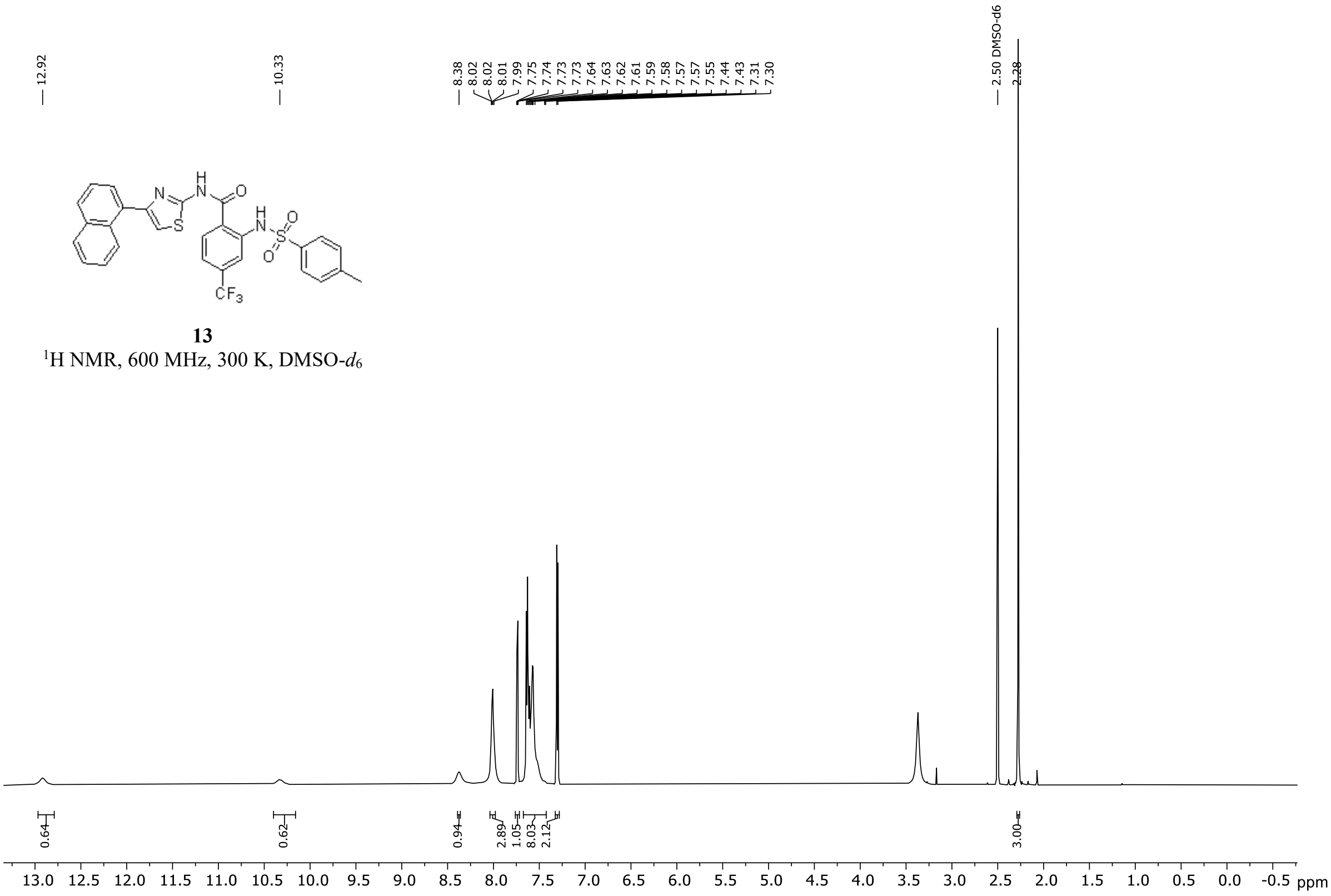

13
^{1}H NMR, 600 MHz, 300 K, DMSO-d_6

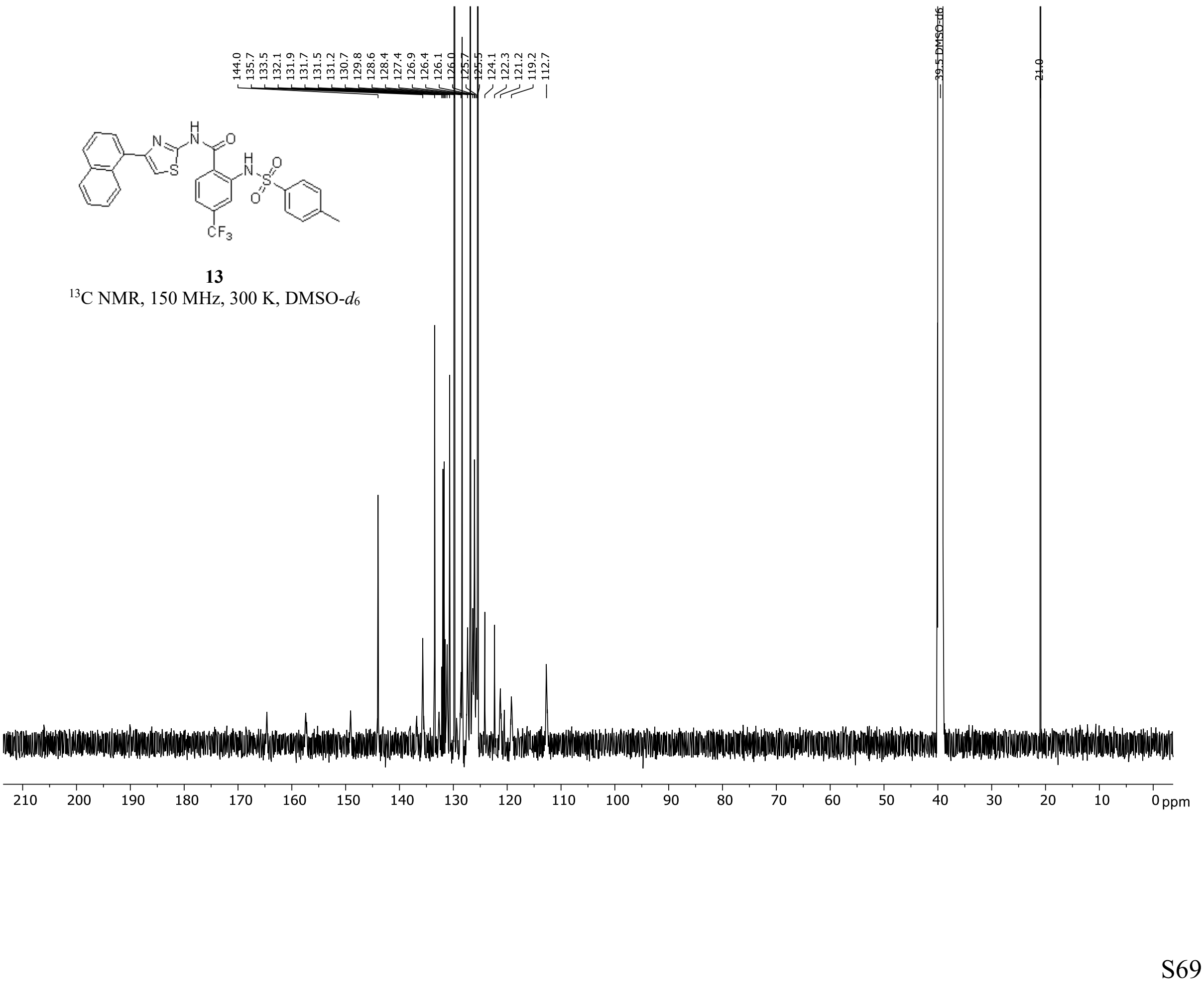

13
^{13}C NMR, 150 MHz, 300 K, DMSO-d_6

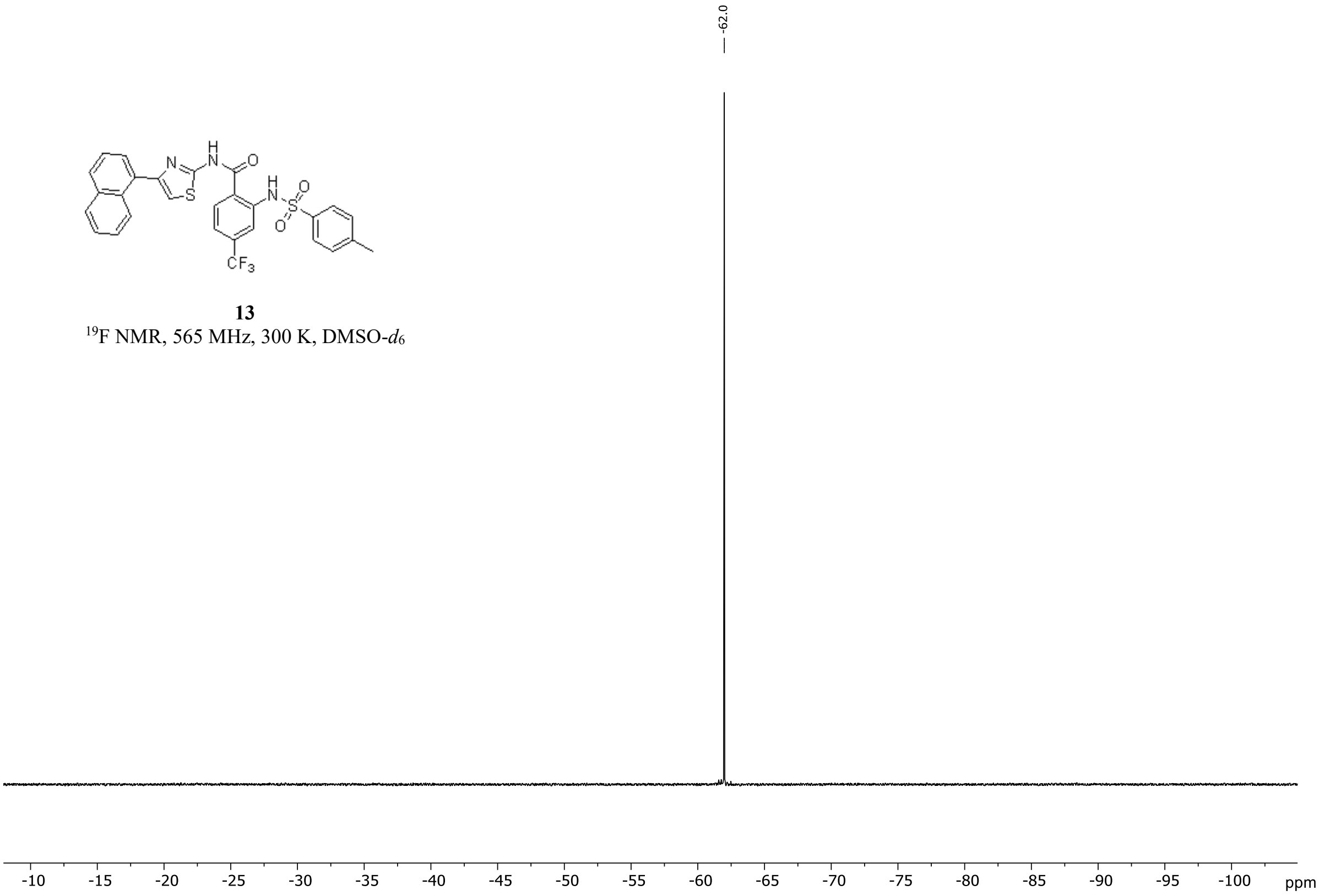

13

^{19}F NMR, 565 MHz, 300 K, DMSO-d_6

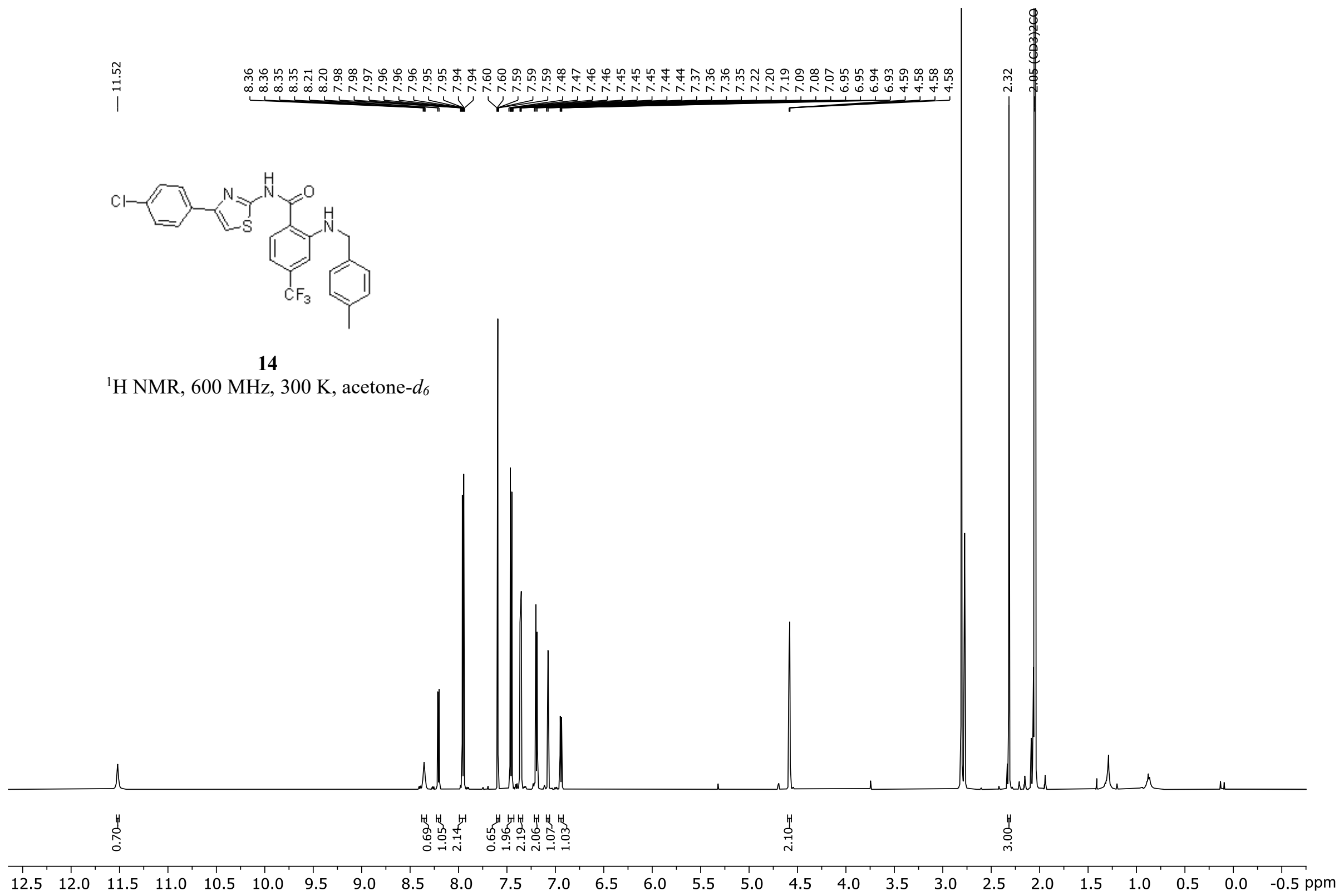

14

^{1}H NMR, 600 MHz, 300 K, acetone-d_6

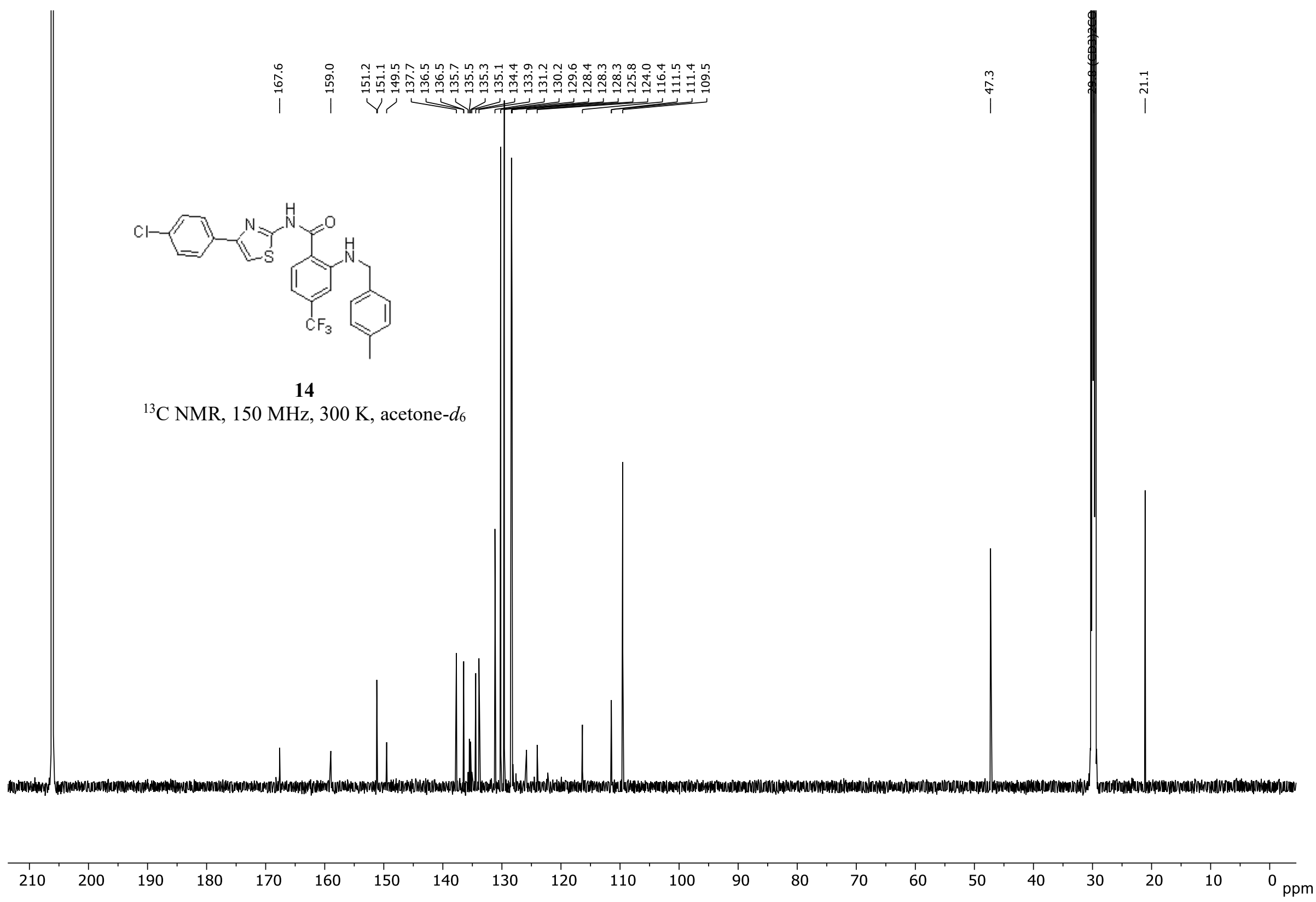

14

^{13}C NMR, 150 MHz, 300 K, acetone-d_6

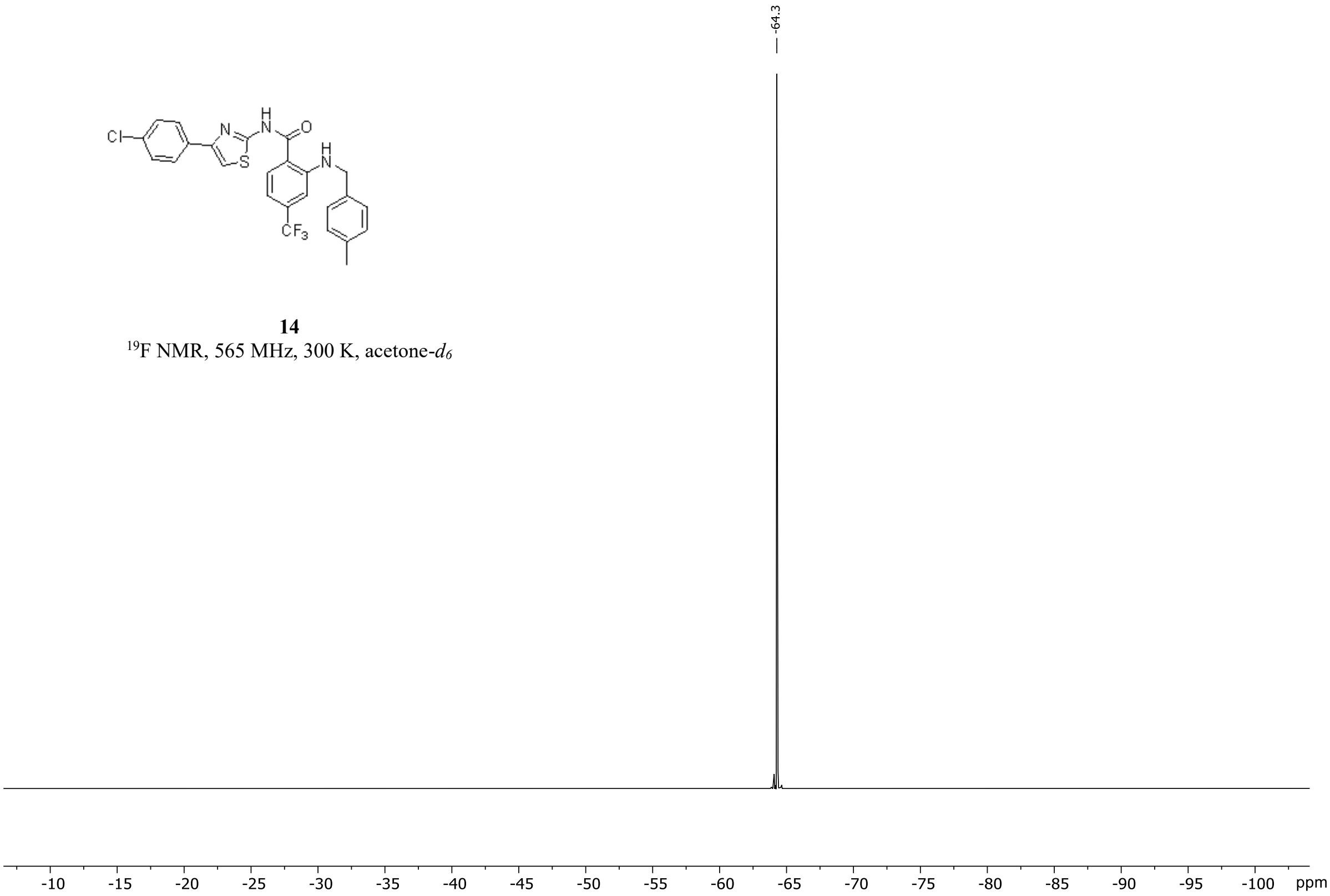

14

^{19}F NMR, 565 MHz, 300 K, acetone-d_6

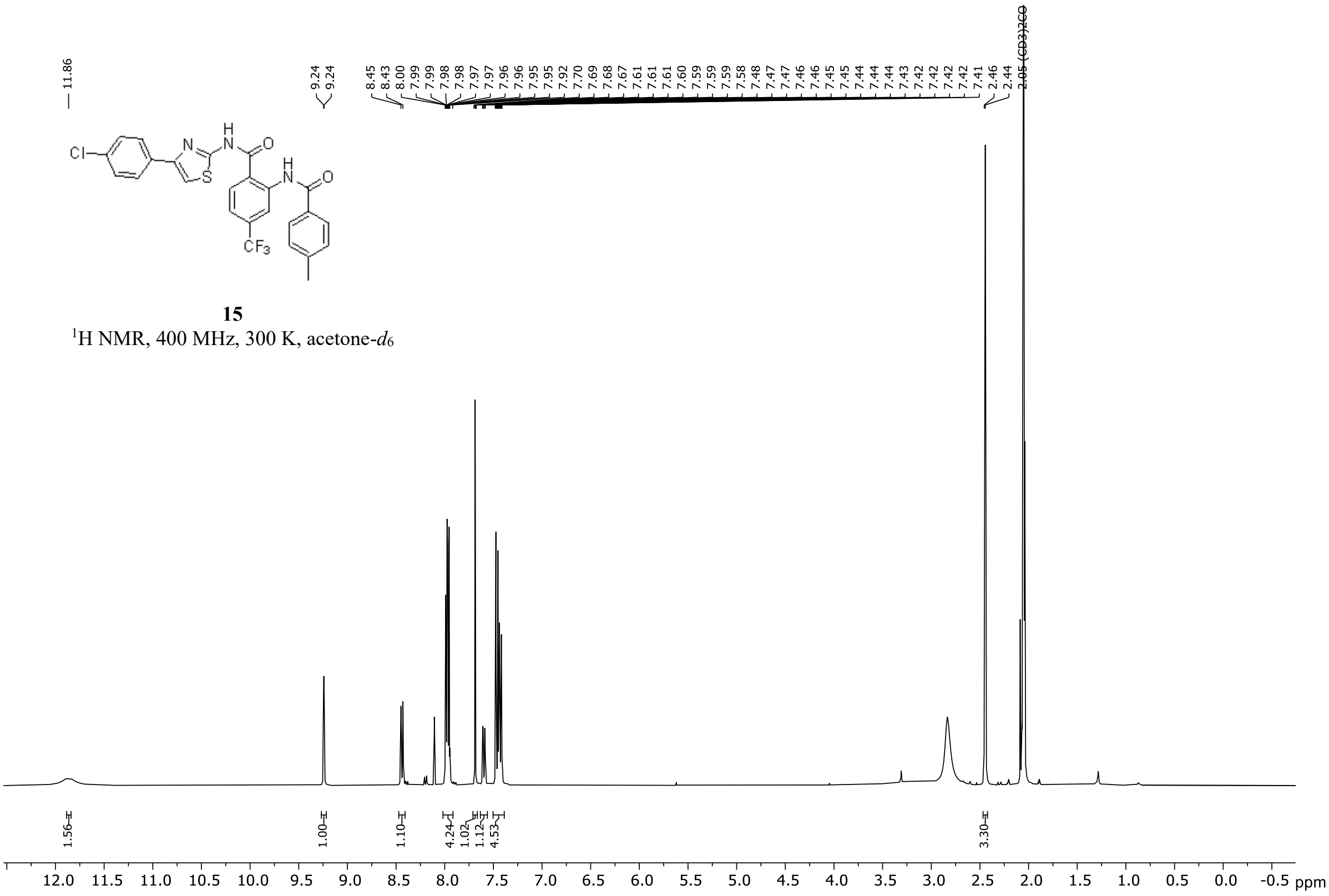

15

^{1}H NMR, 400 MHz, 300 K, acetone-d_6

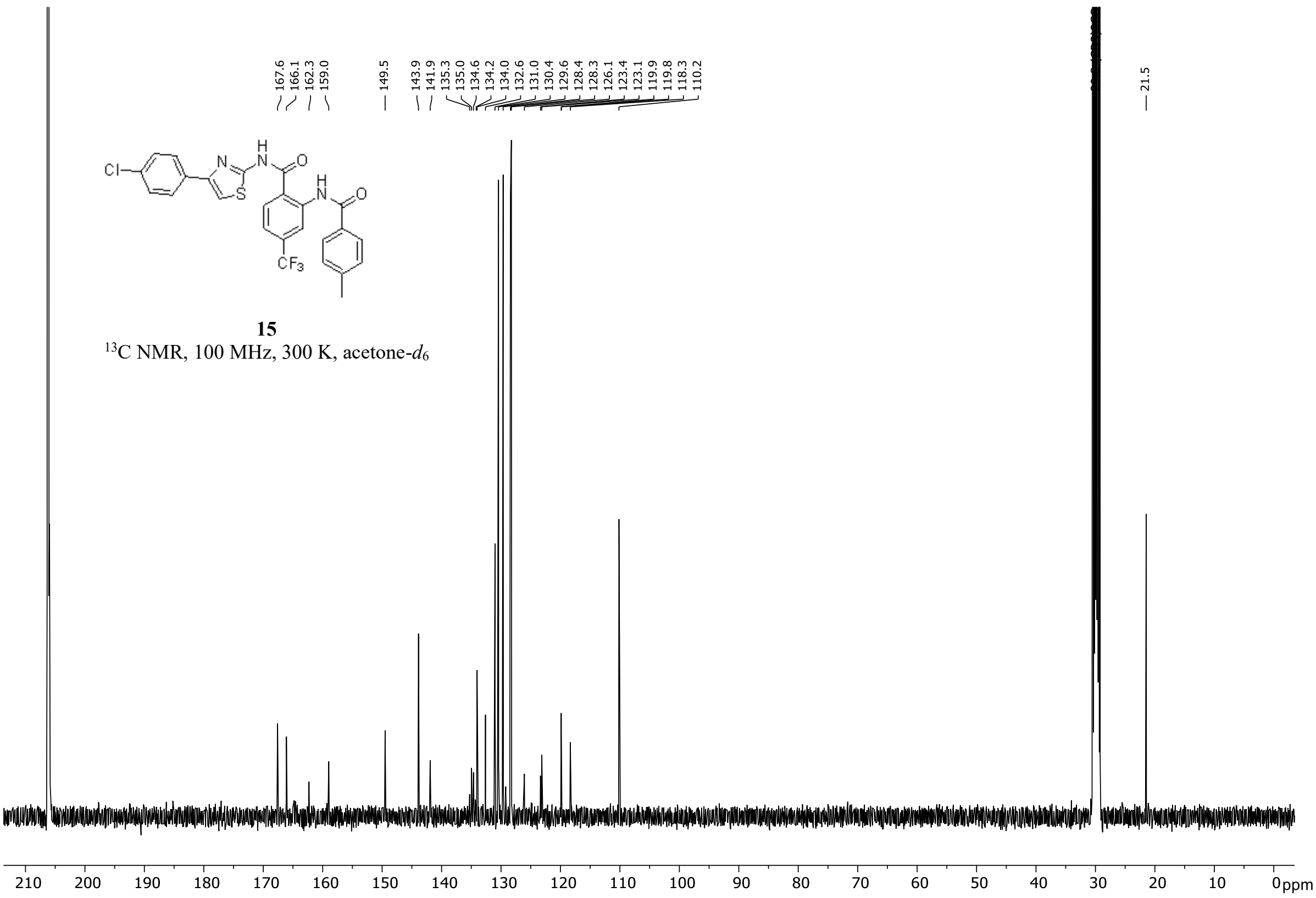

15

^{13}C NMR, 100 MHz, 300 K, acetone-d_6

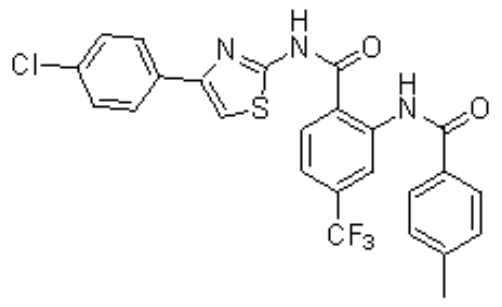

15
^{19}F NMR, 375 MHz, 300 K, acetone-d_6

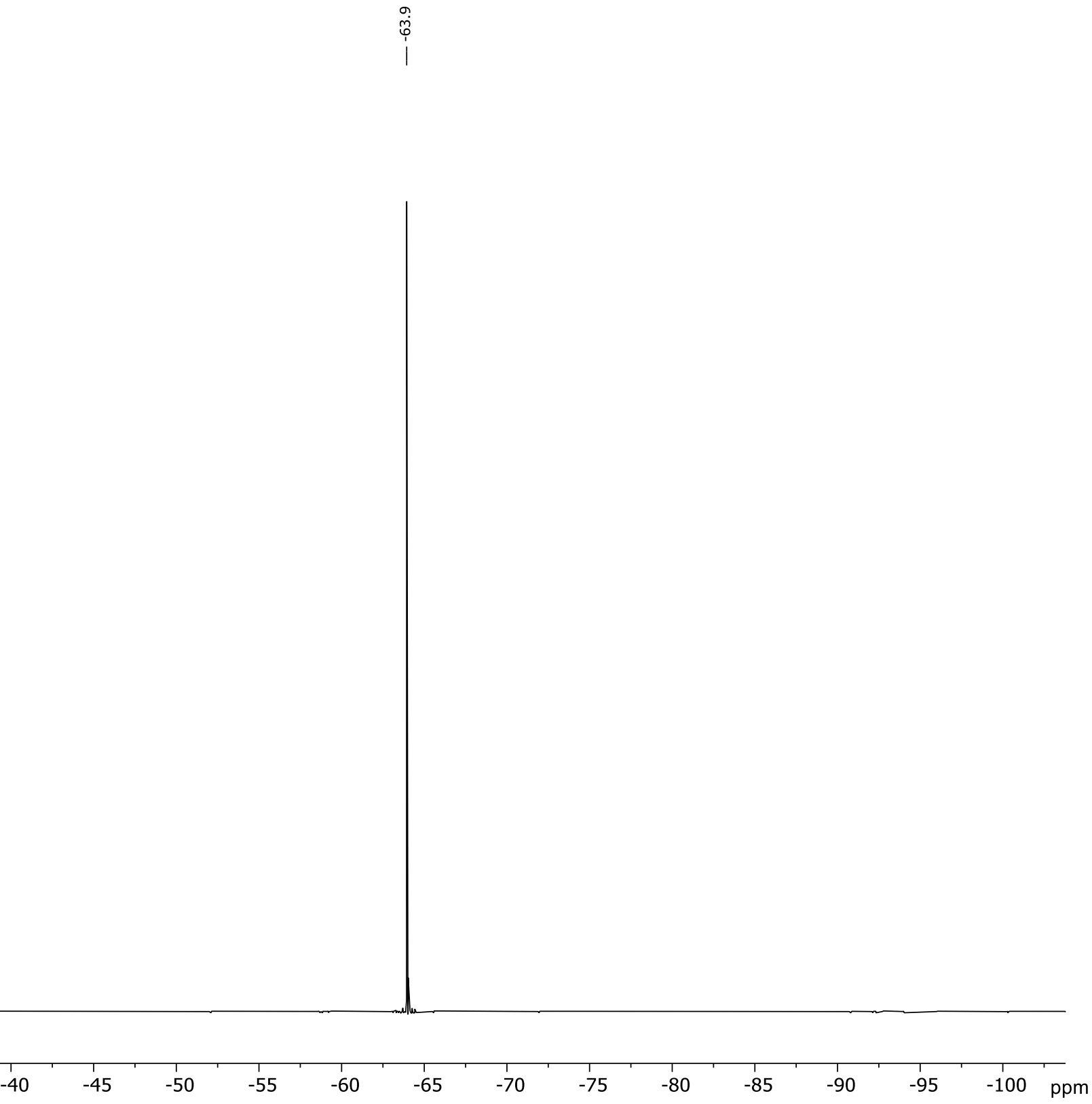

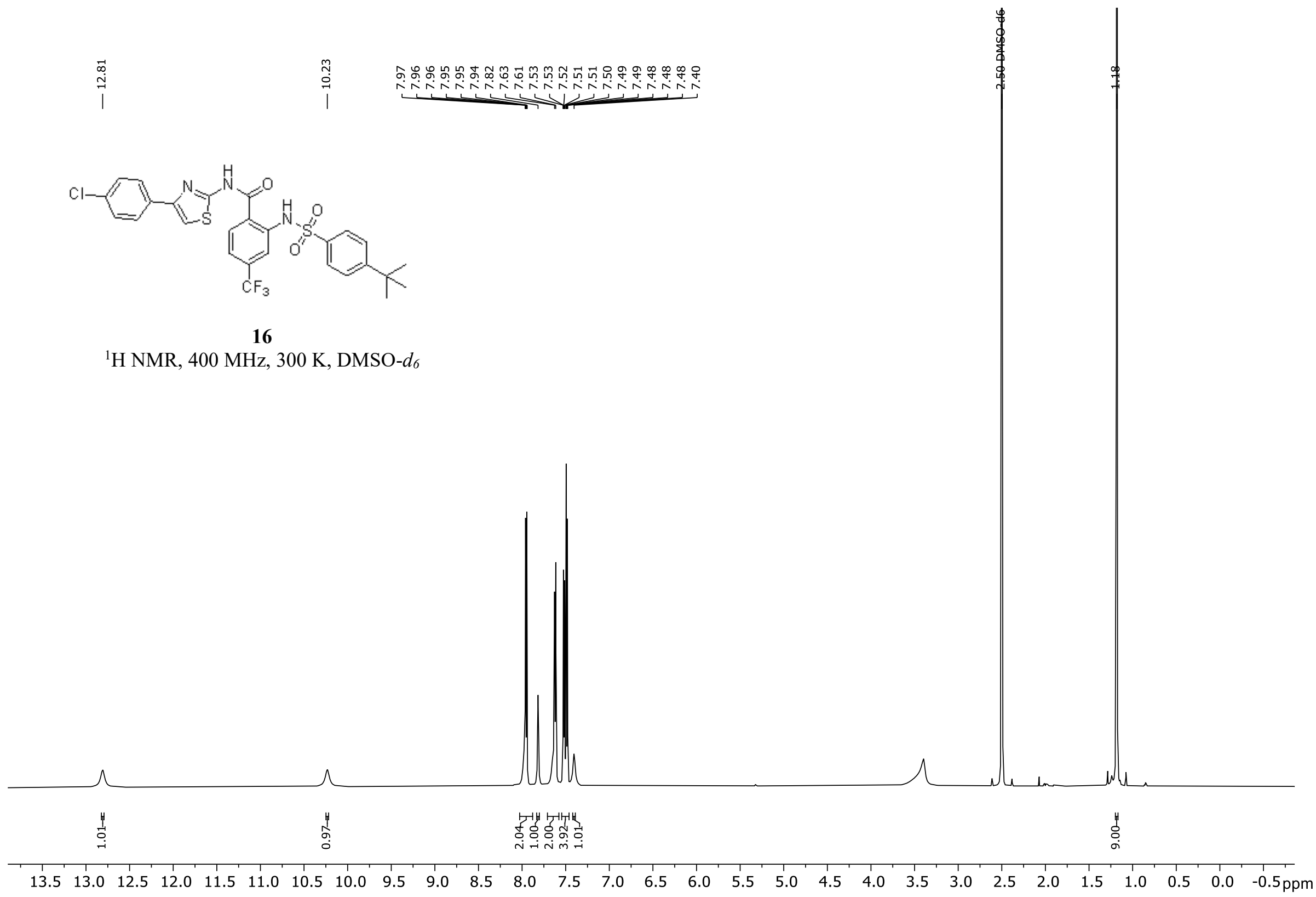

16

^{1}H NMR, 400 MHz, 300 K, DMSO-d_6

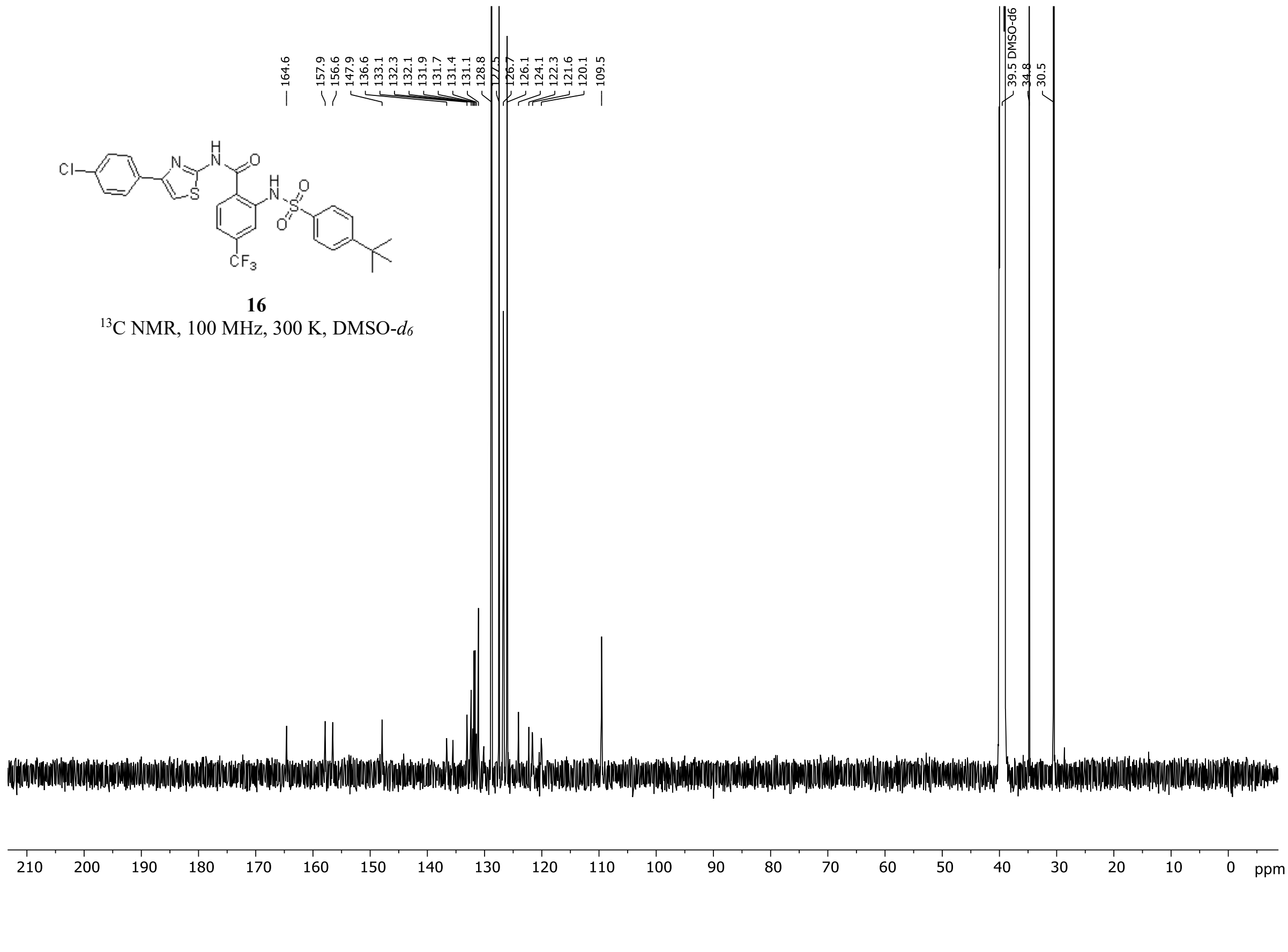

16

^{13}C NMR, 100 MHz, 300 K, DMSO-d_6

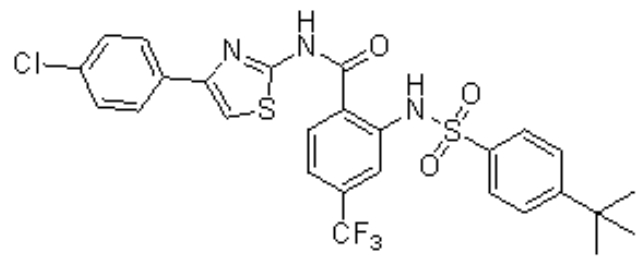

16
^{19}F NMR, 375 MHz, 300 K, DMSO-d_6

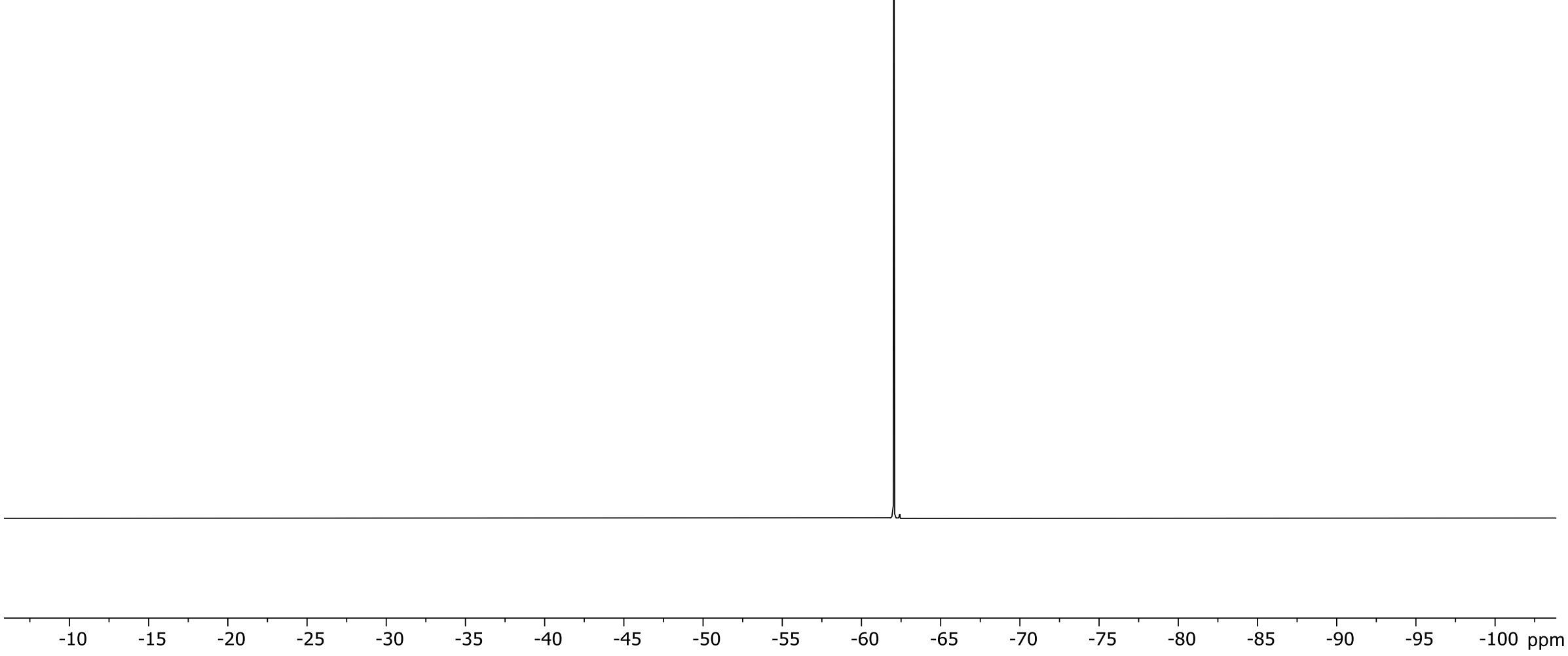

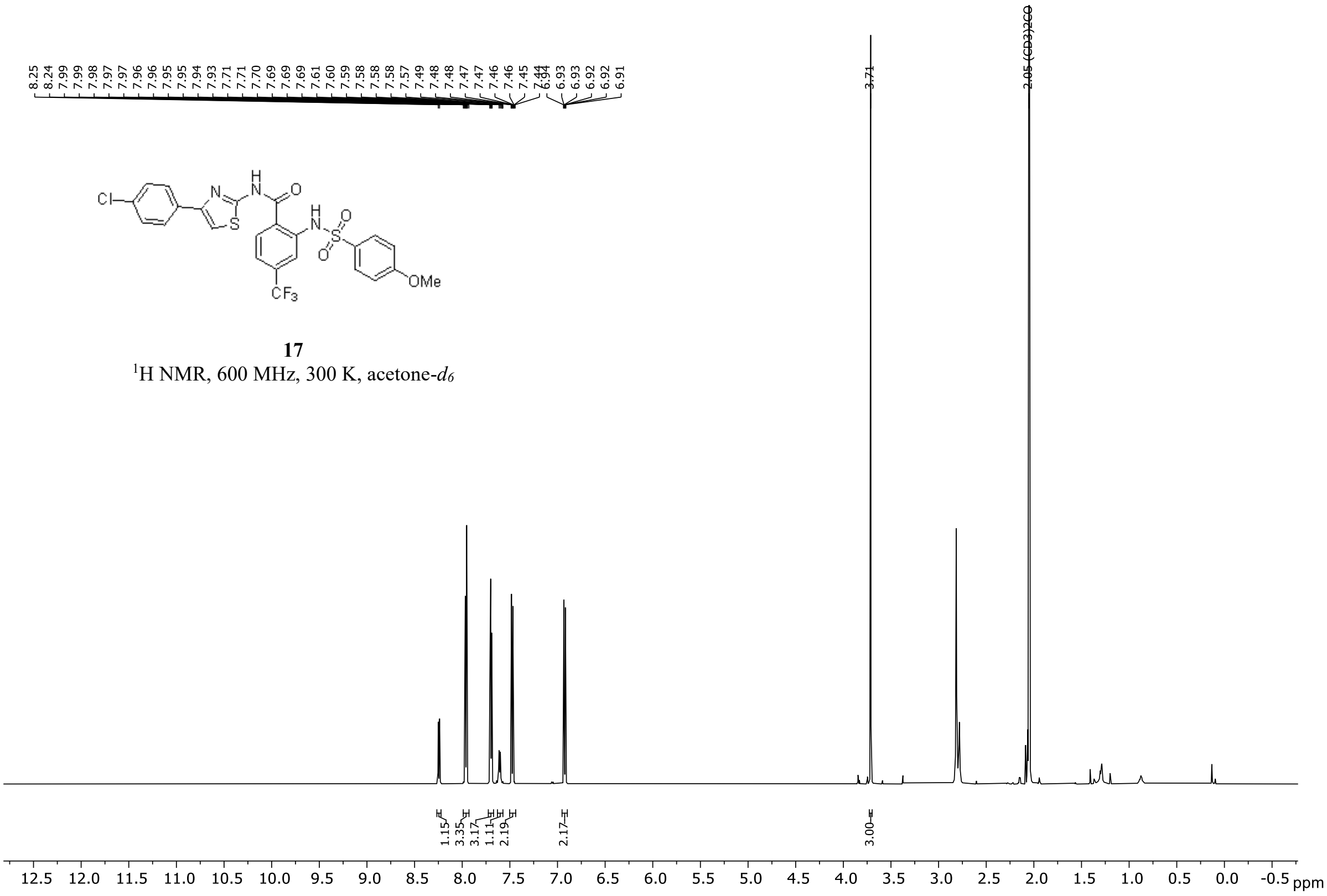

17
^{1}H NMR, 600 MHz, 300 K, acetone-d_6

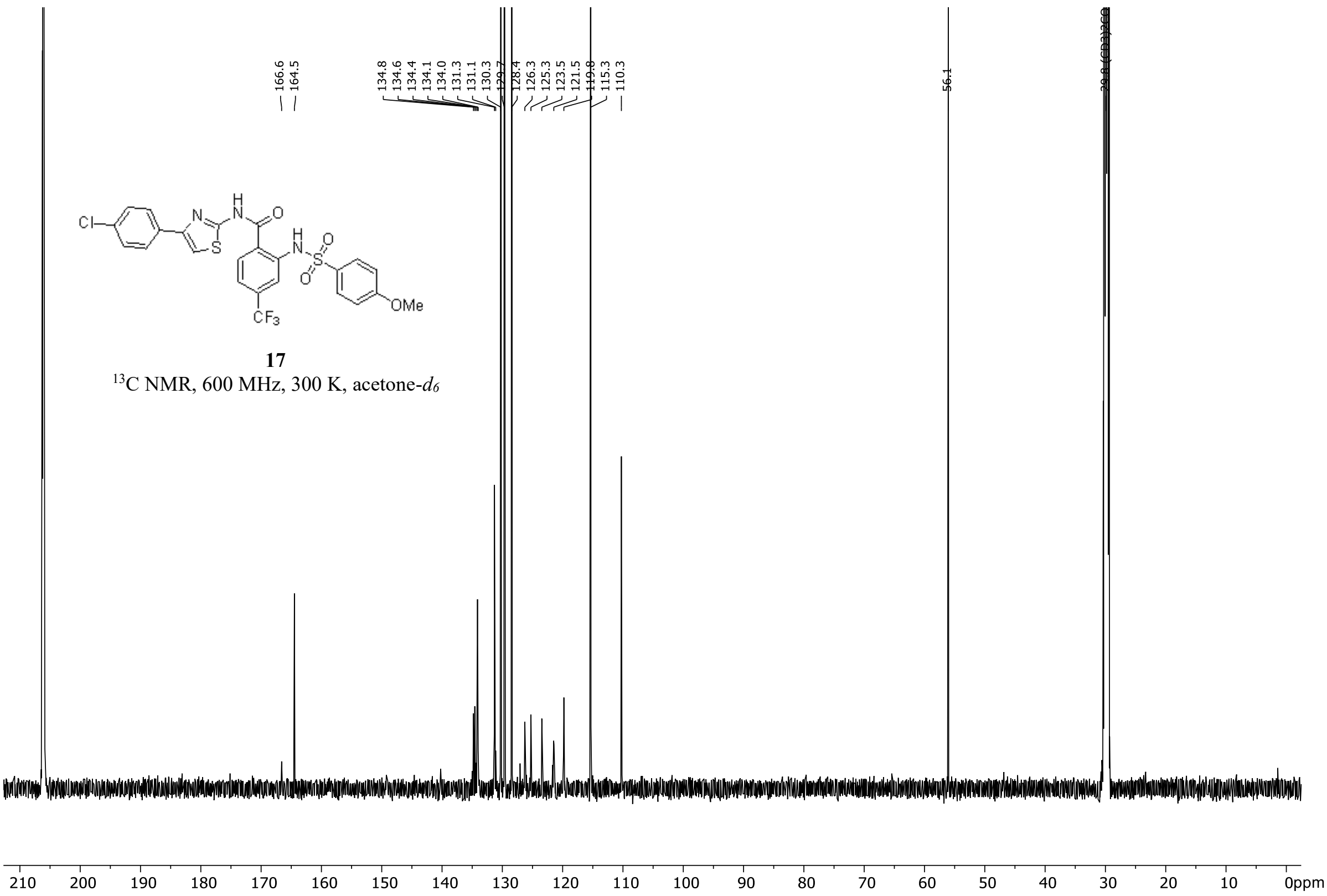

17
^{13}C NMR, 600 MHz, 300 K, acetone-d_6

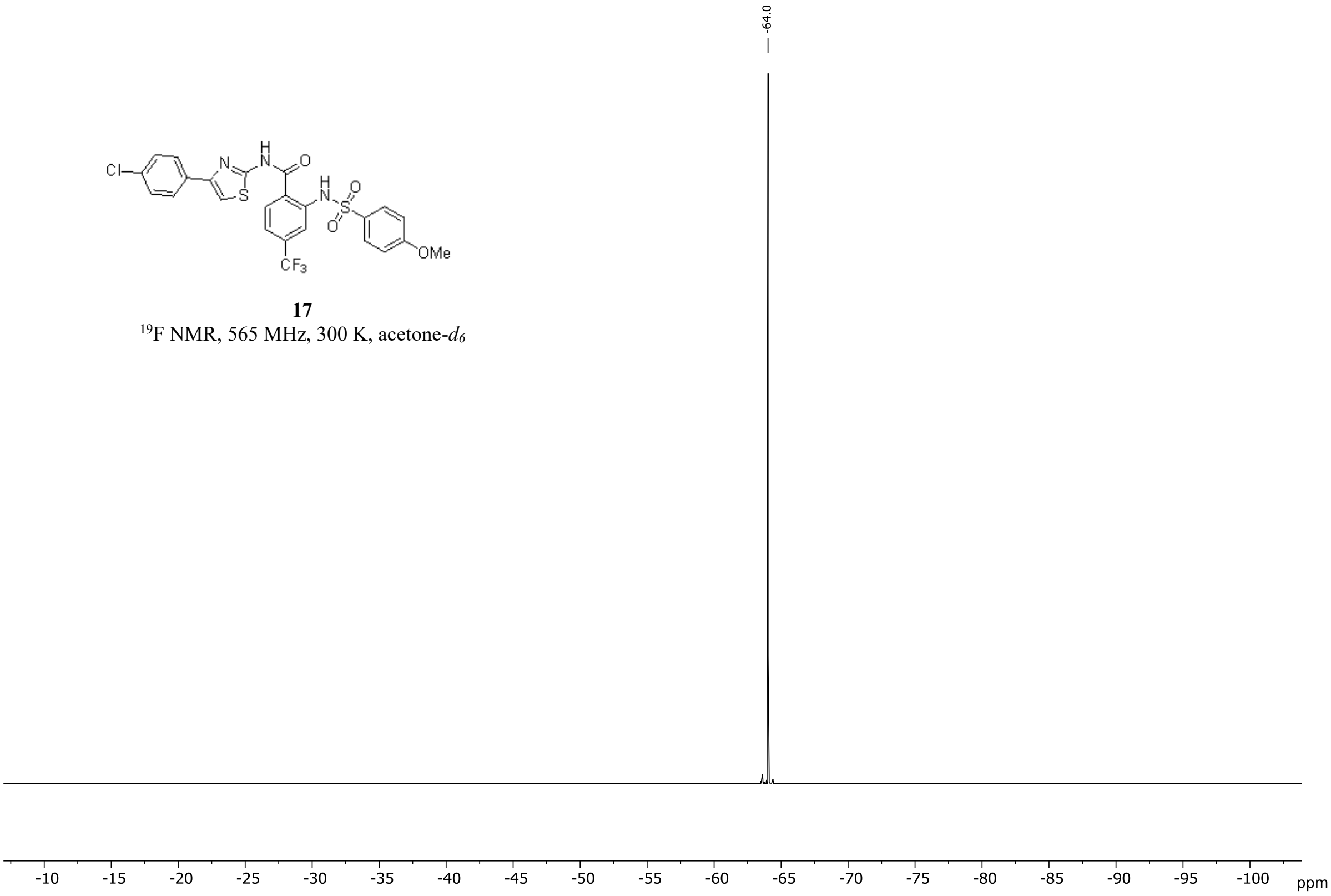

17

^{19}F NMR, 565 MHz, 300 K, acetone-d_6

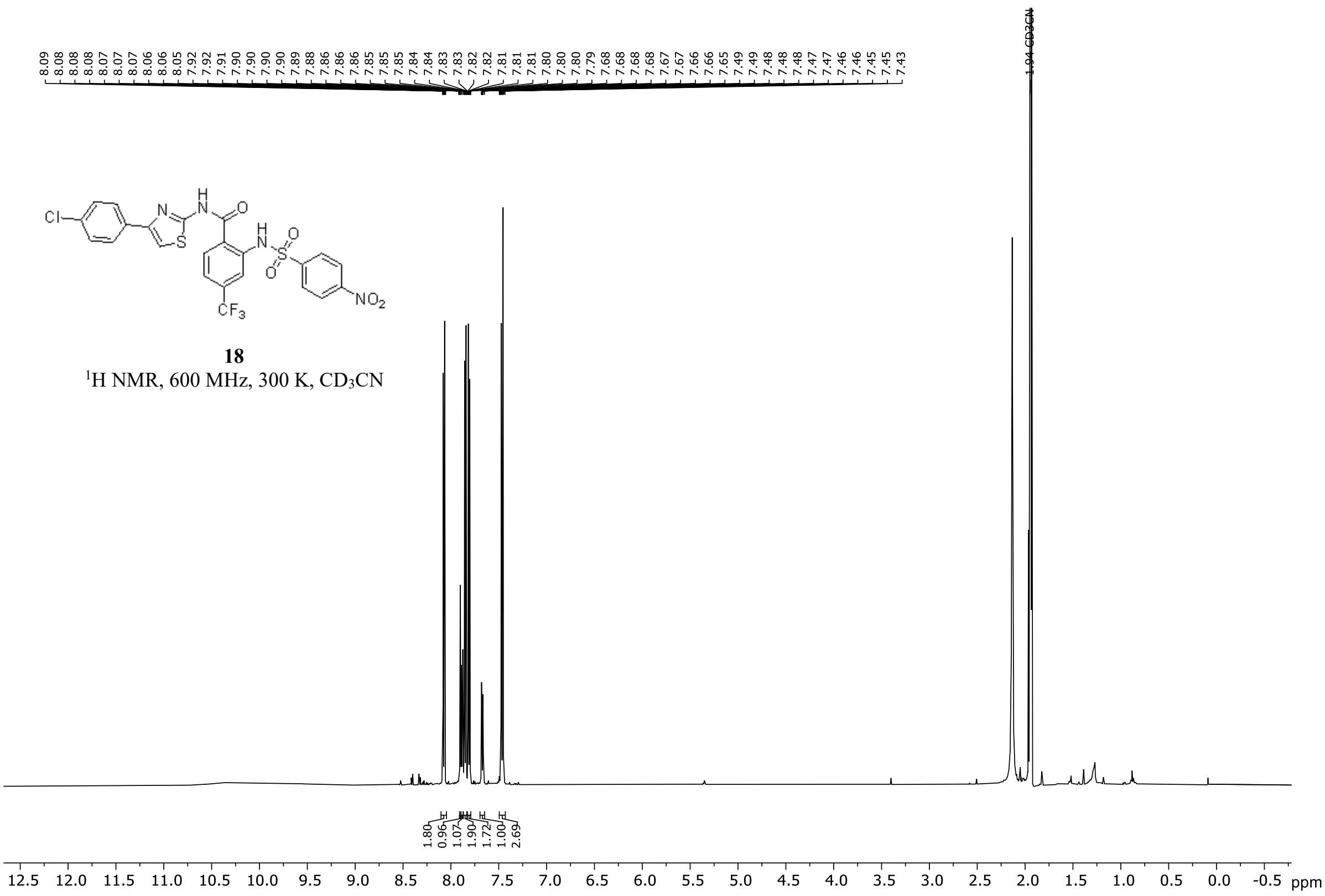

18
^{1}H NMR, 600 MHz, 300 K, CD_3CN

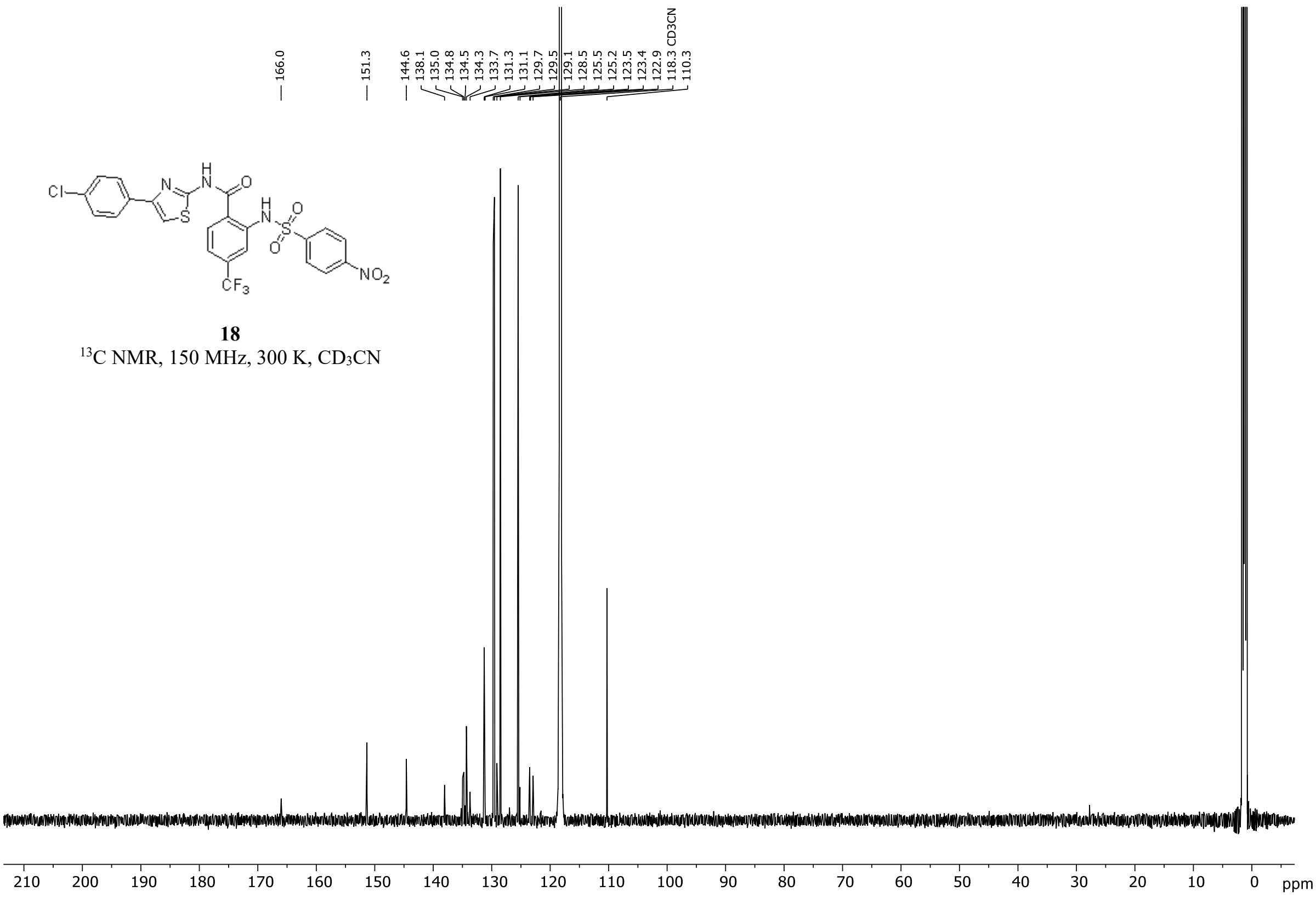

18
^{13}C NMR, 150 MHz, 300 K, CD_3CN

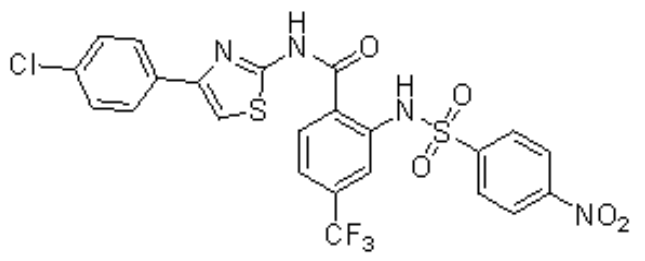

18

^{19}F NMR, 565 MHz, 300 K, CD_3CN

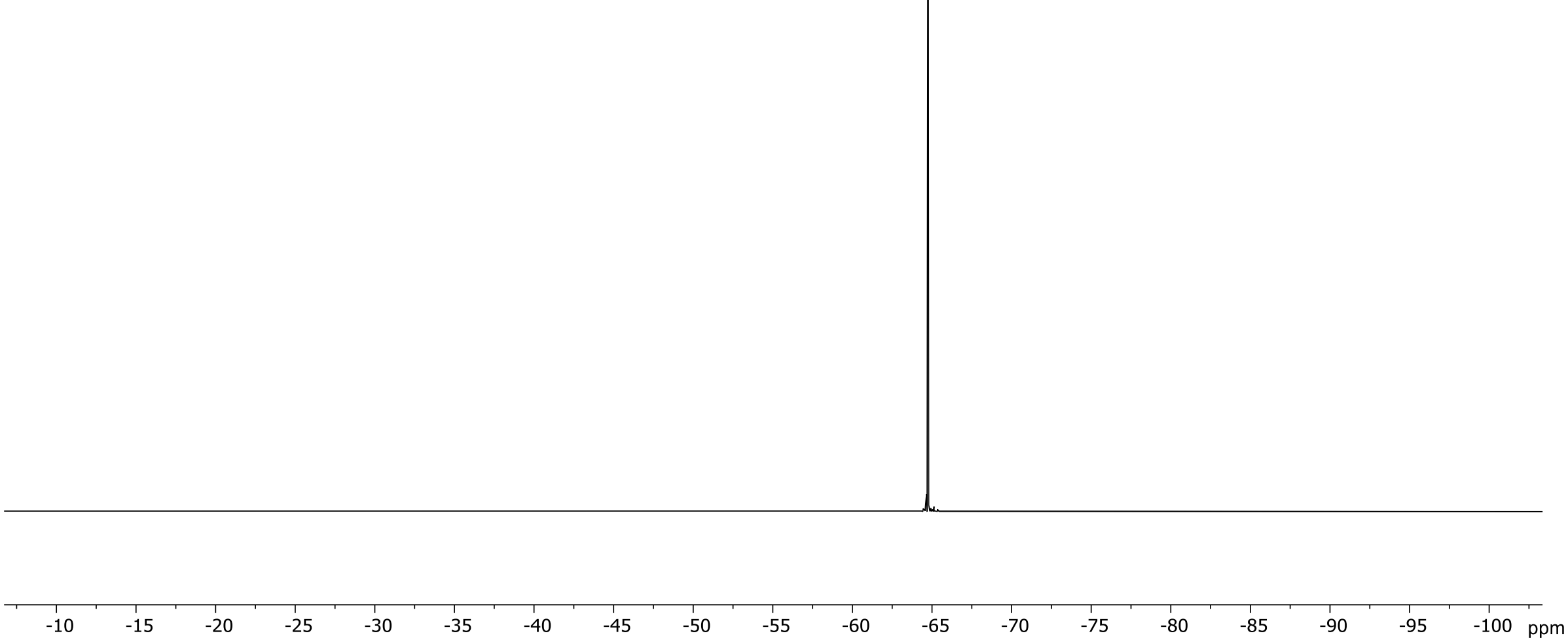

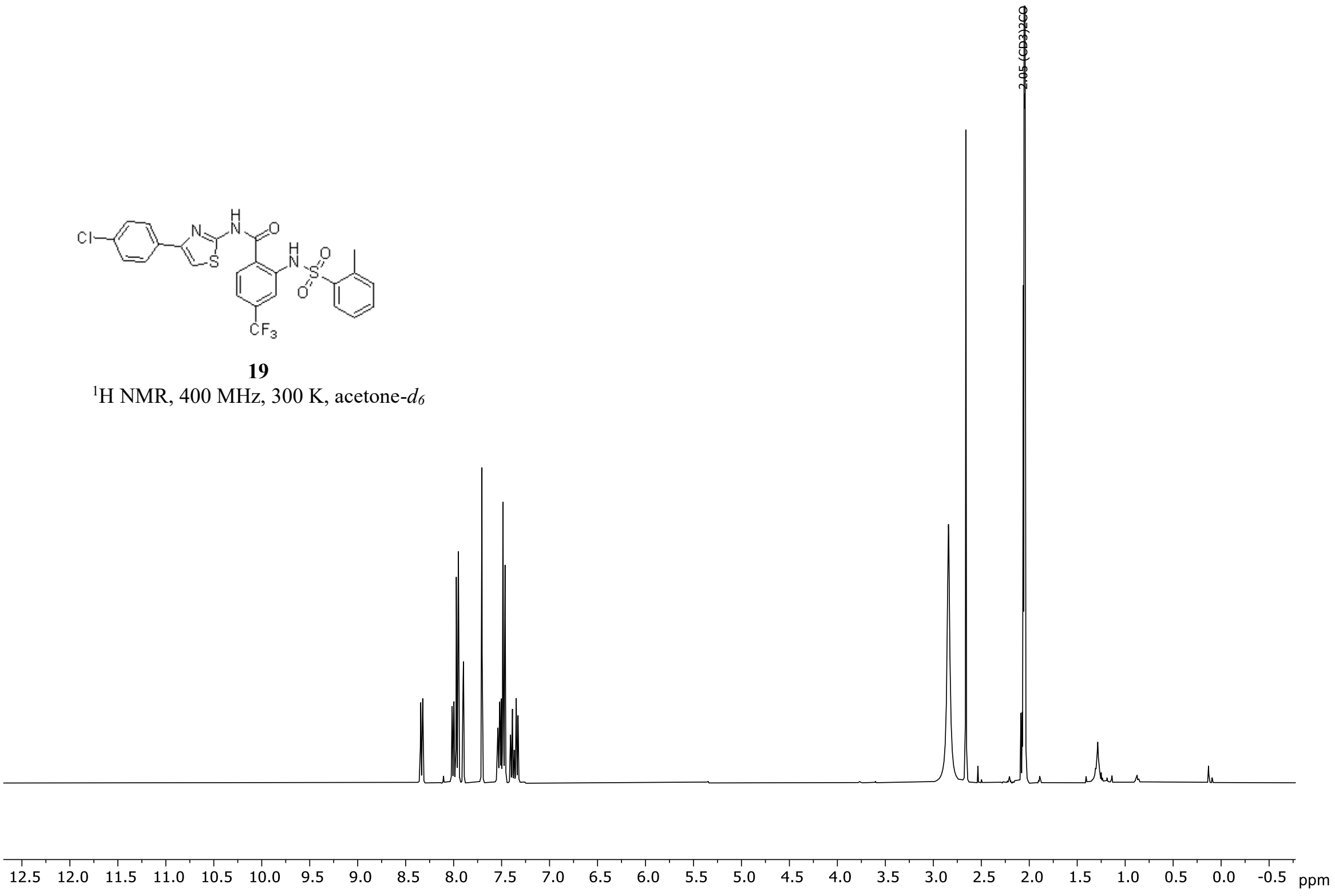

19
^{1}H NMR, 400 MHz, 300 K, acetone-d_6

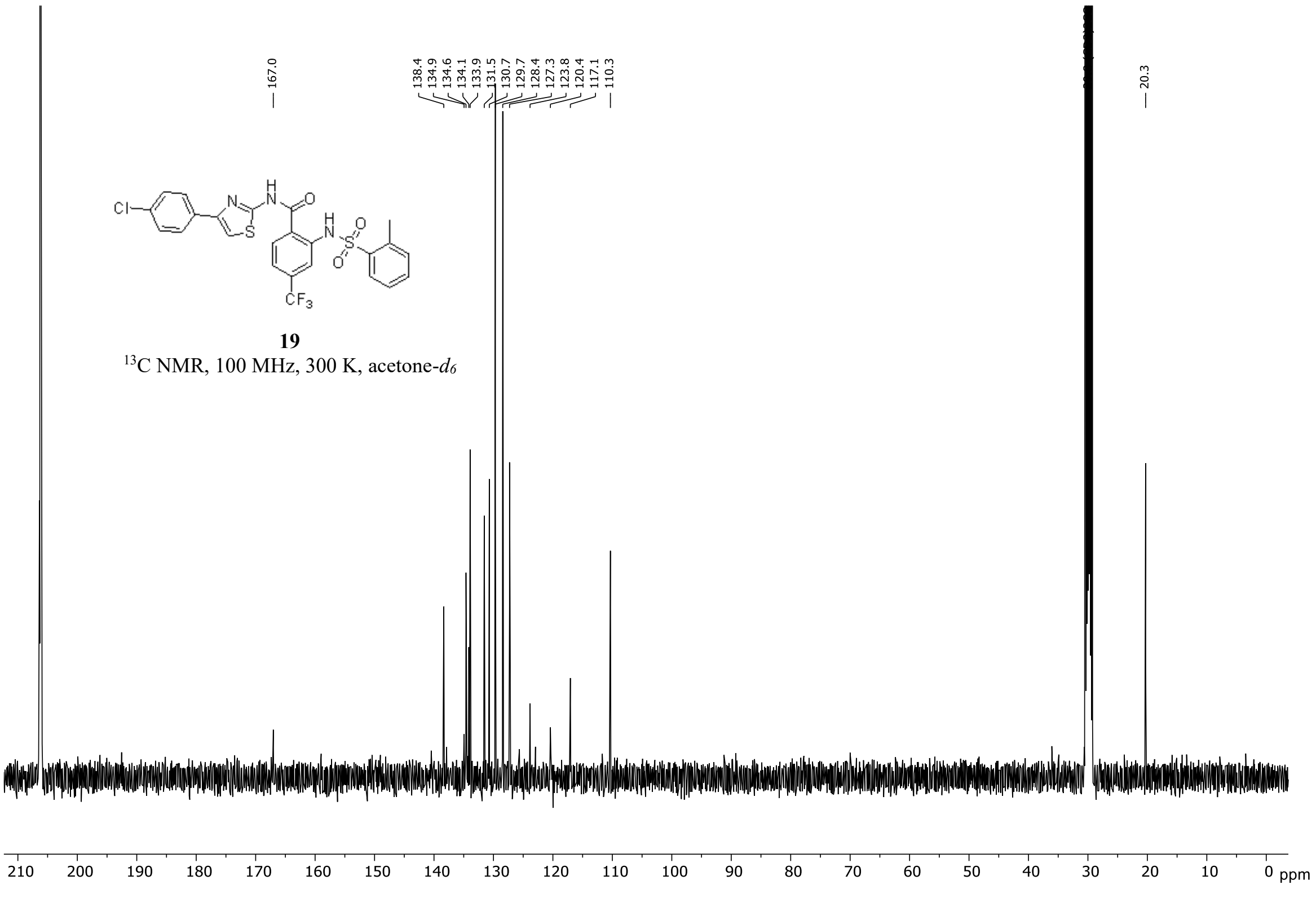

19
^{13}C NMR, 100 MHz, 300 K, acetone-d_6

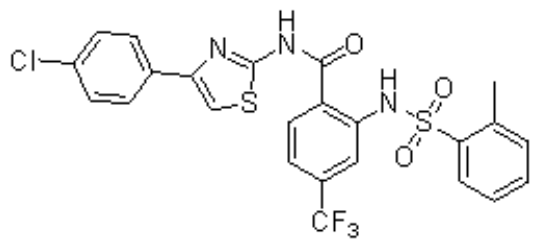

19

^{19}F NMR, 375 MHz, 300 K, acetone-d_6

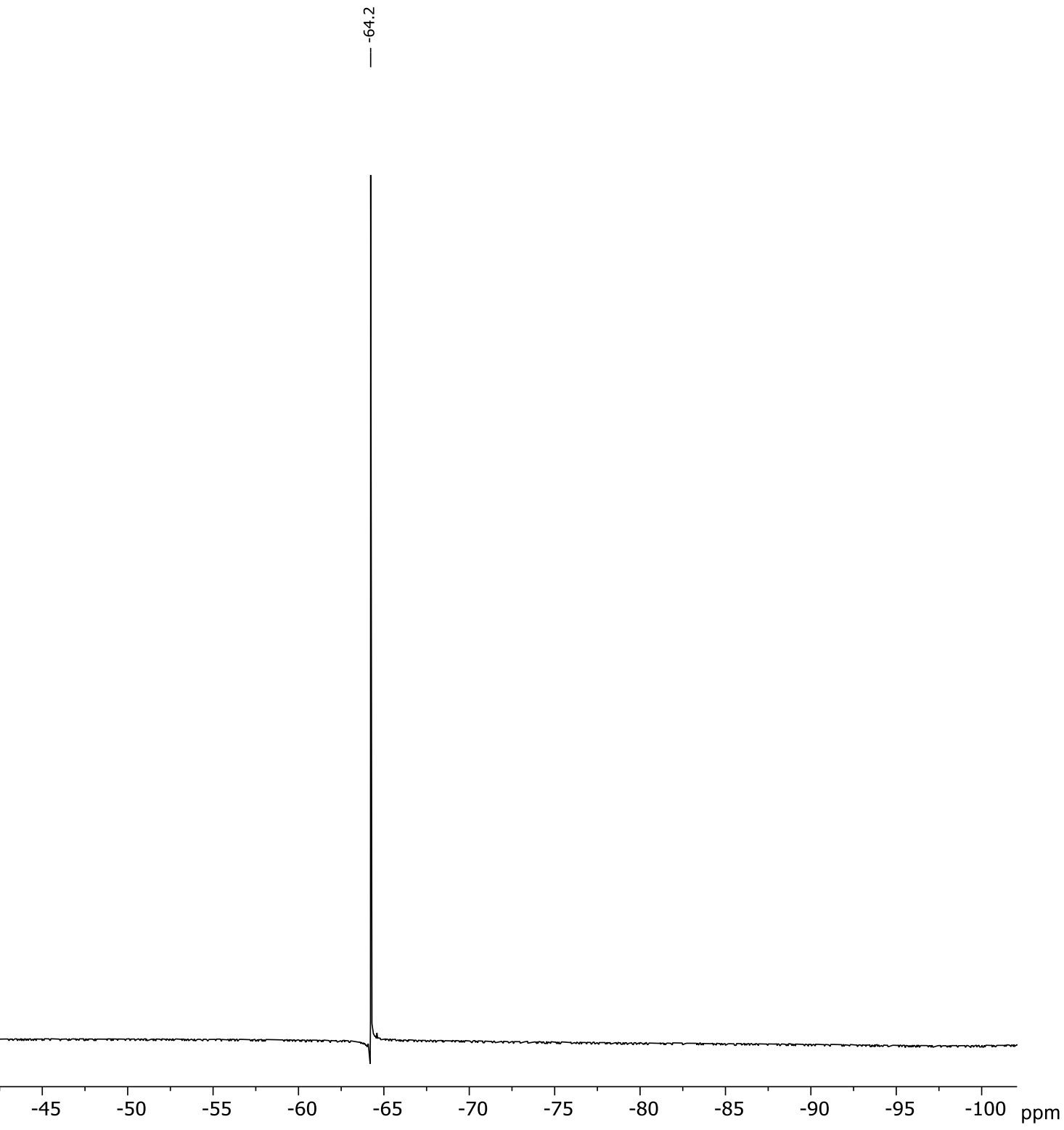

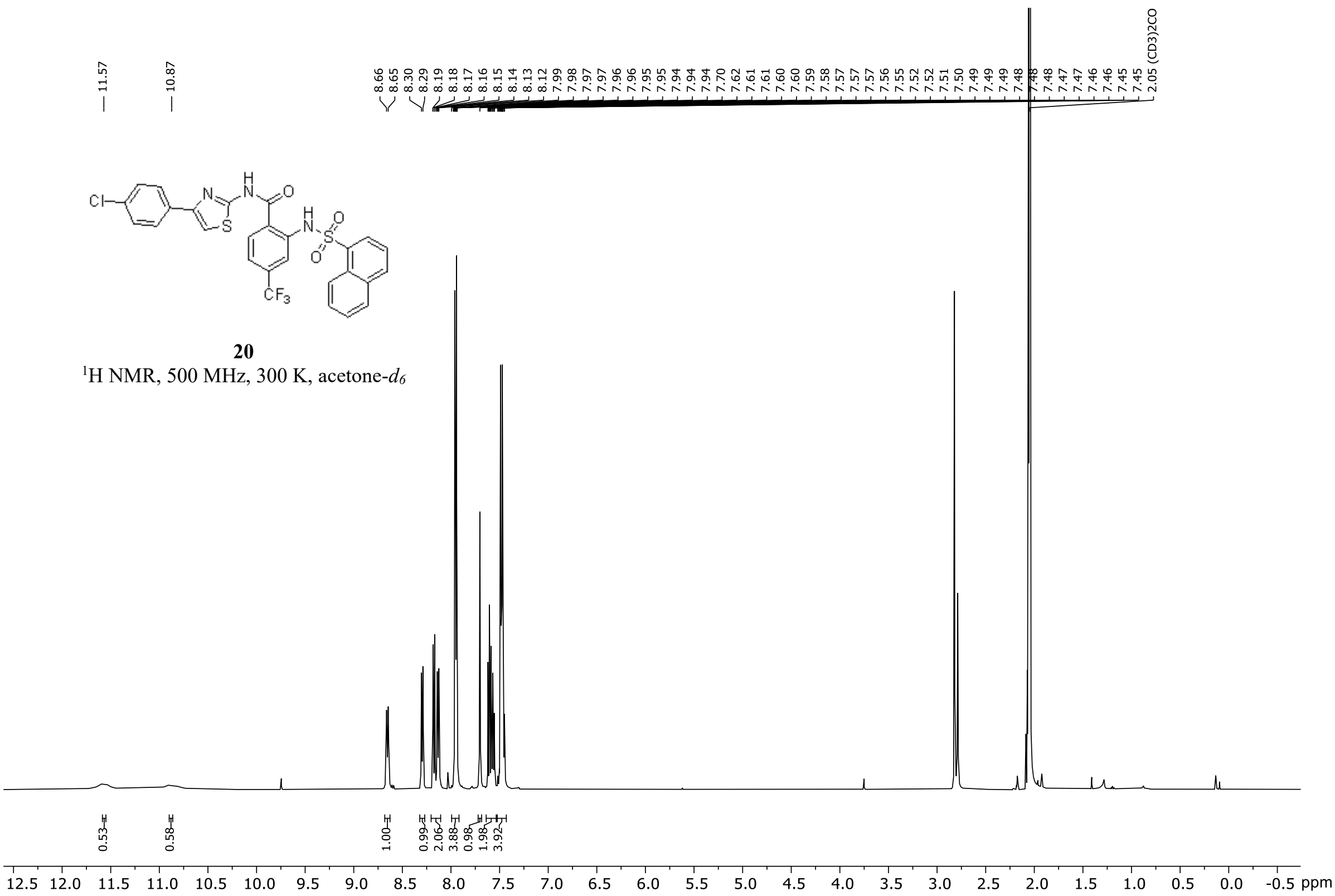

20
^{1}H NMR, 500 MHz, 300 K, acetone-d_6

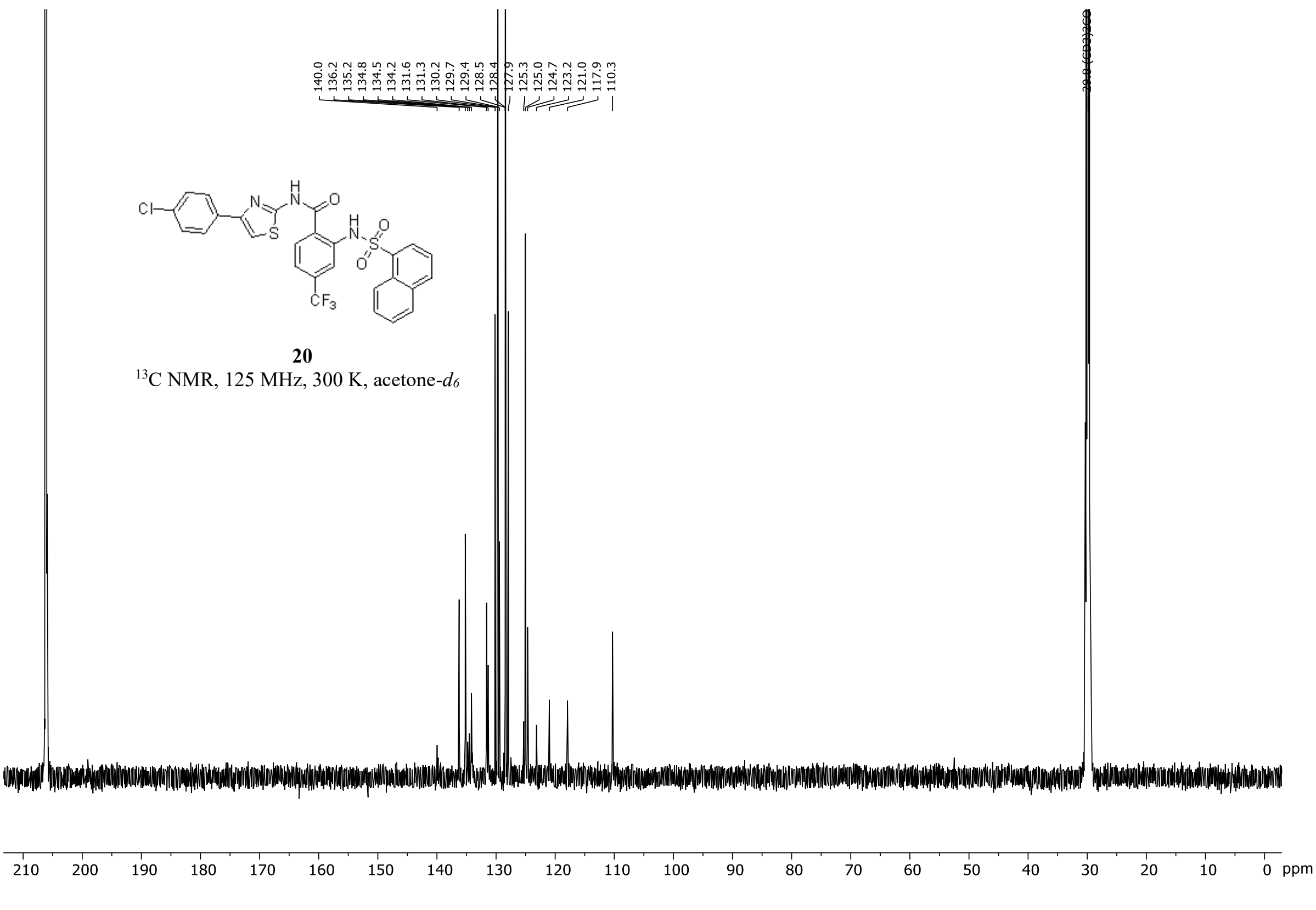

20
^{13}C NMR, 125 MHz, 300 K, acetone-d_6

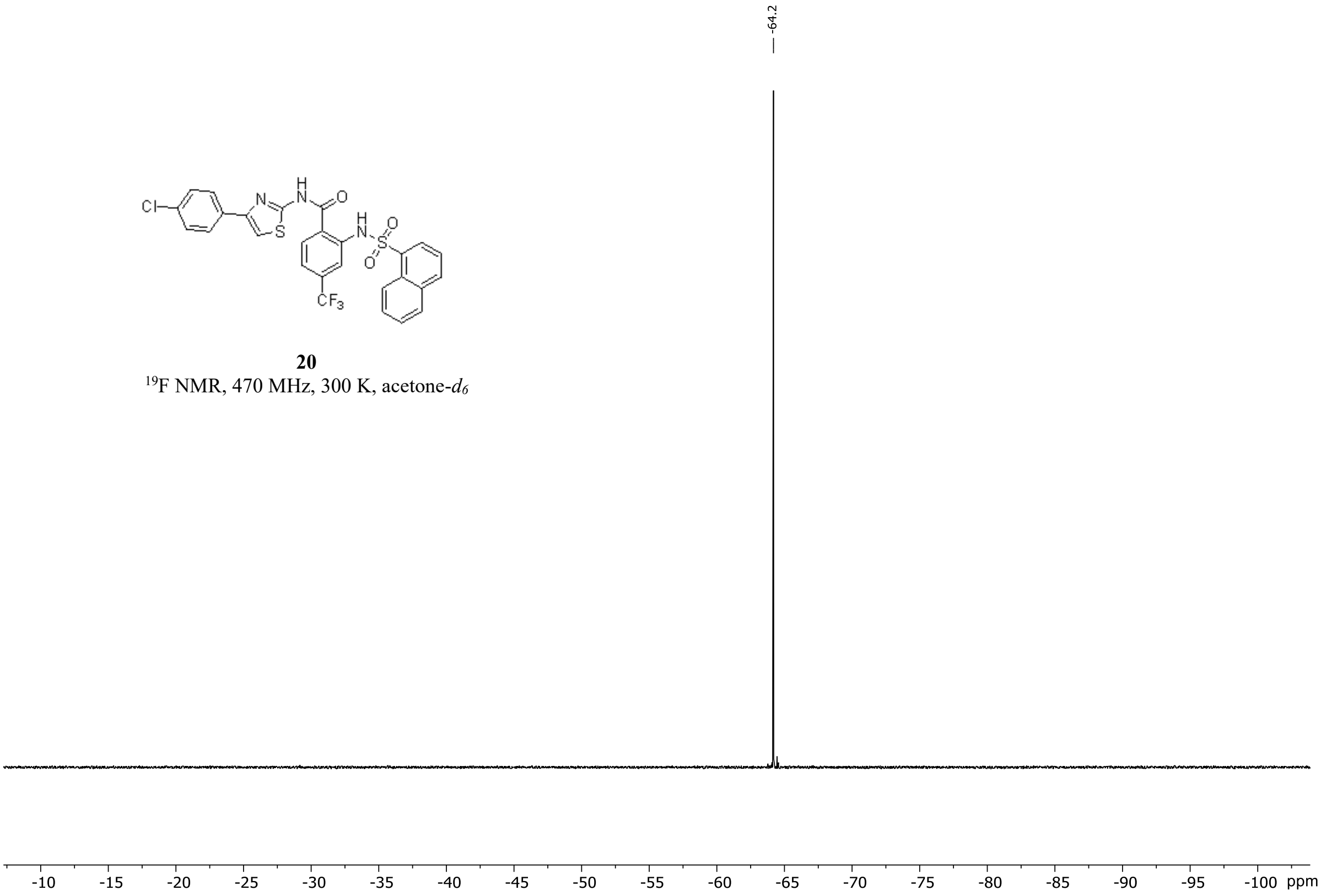

20

^{19}F NMR, 470 MHz, 300 K, acetone-d_6

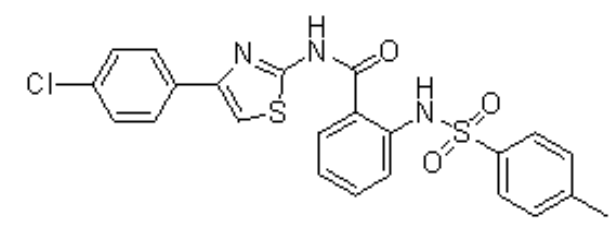

21
^{1}H NMR, 600 MHz, 300 K, CD_3CN

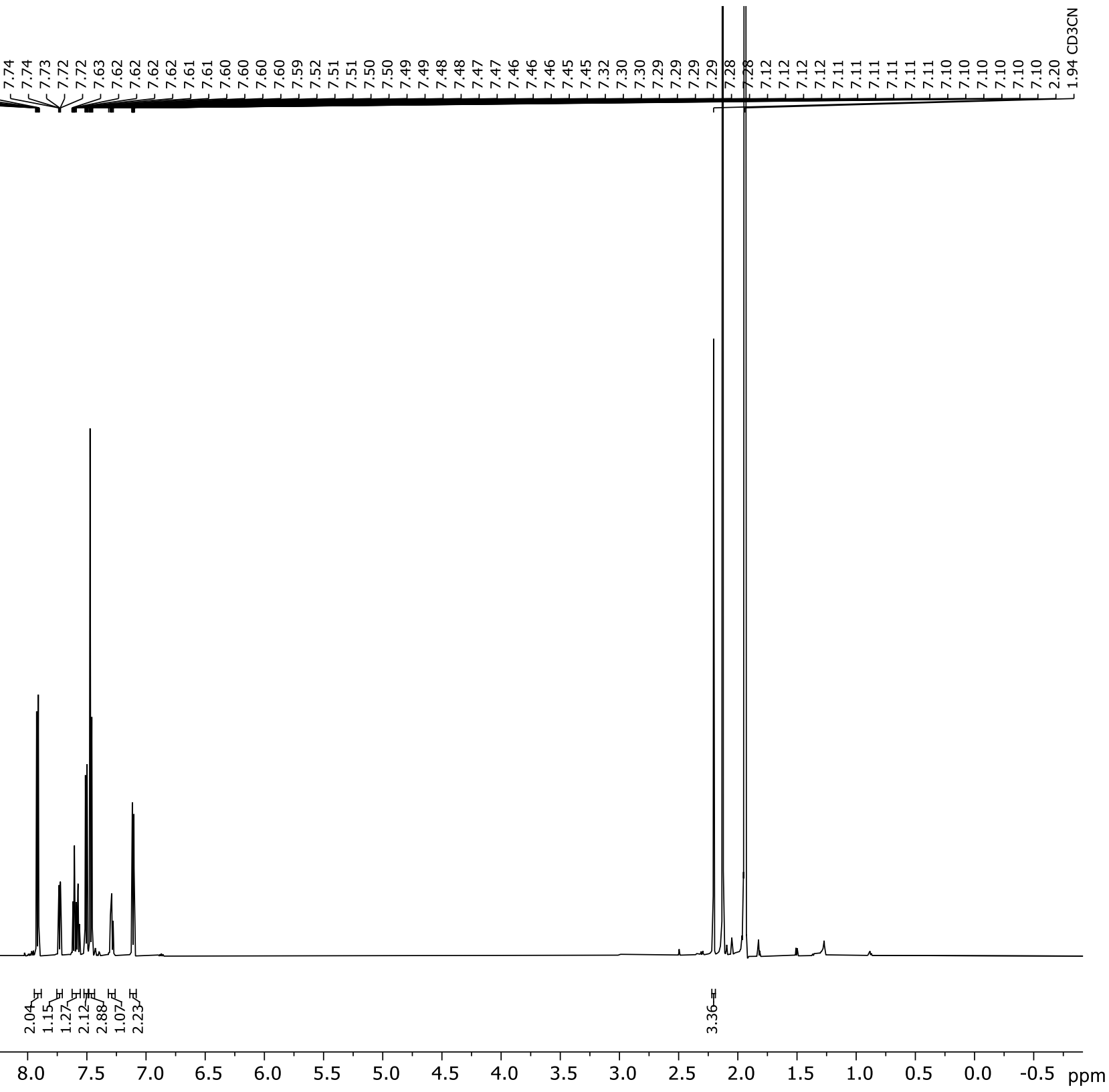

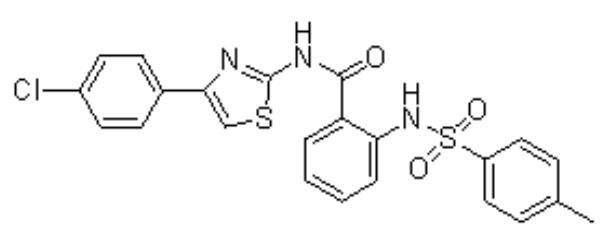

21
^{13}C NMR, 150 MHz, 300 K, CD_3CN

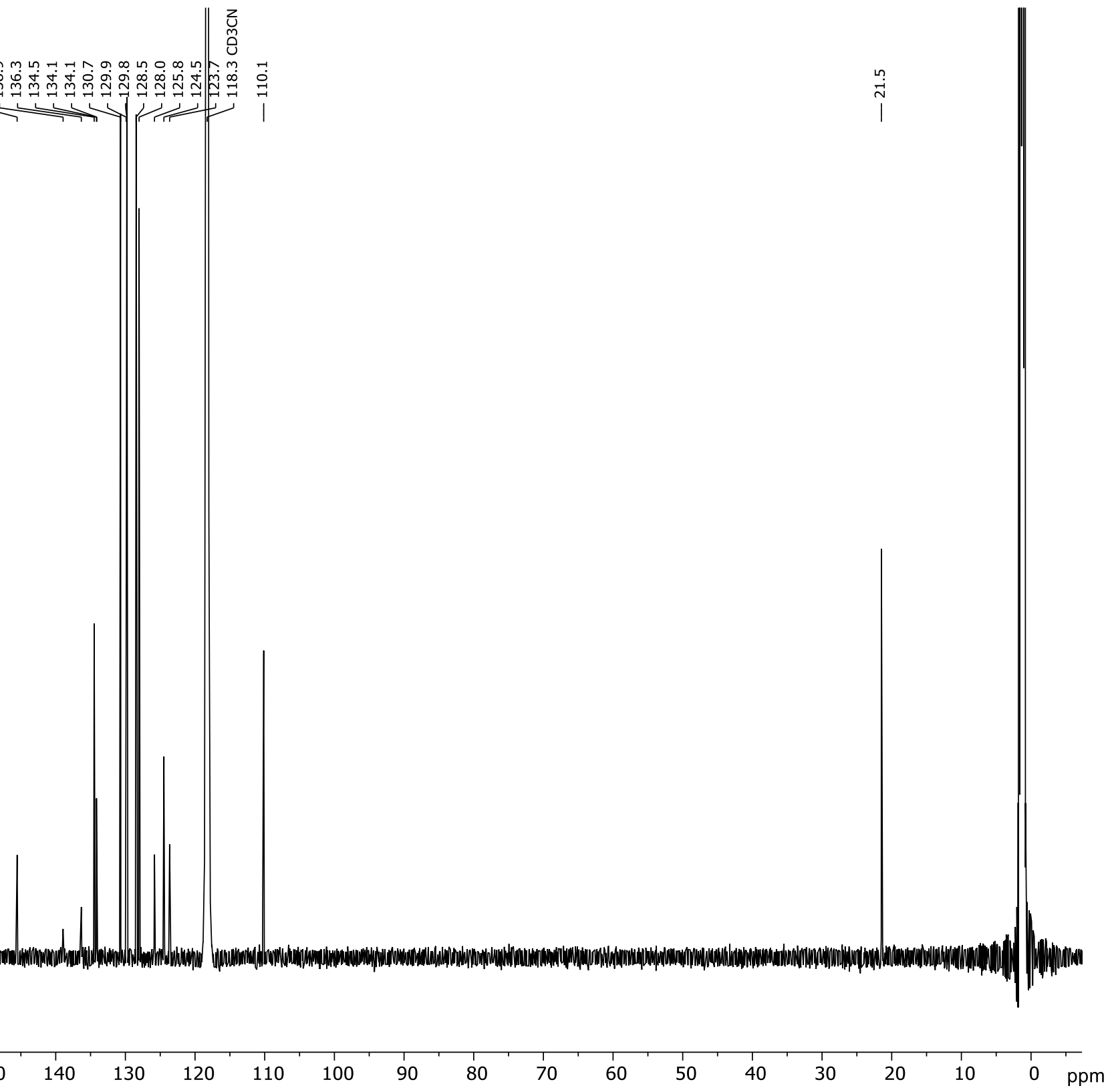

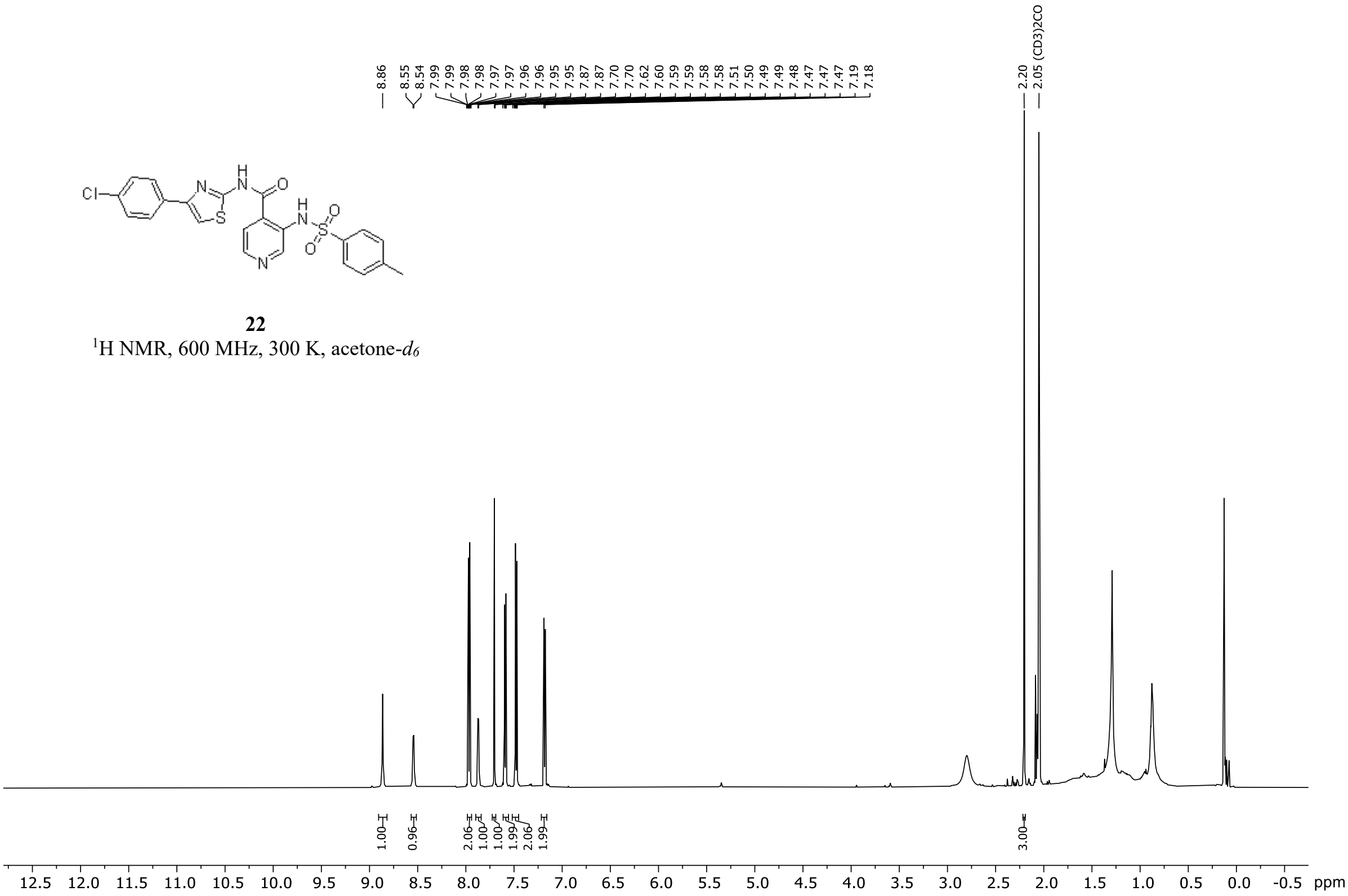

22

^{1}H NMR, 600 MHz, 300 K, acetone-d_6

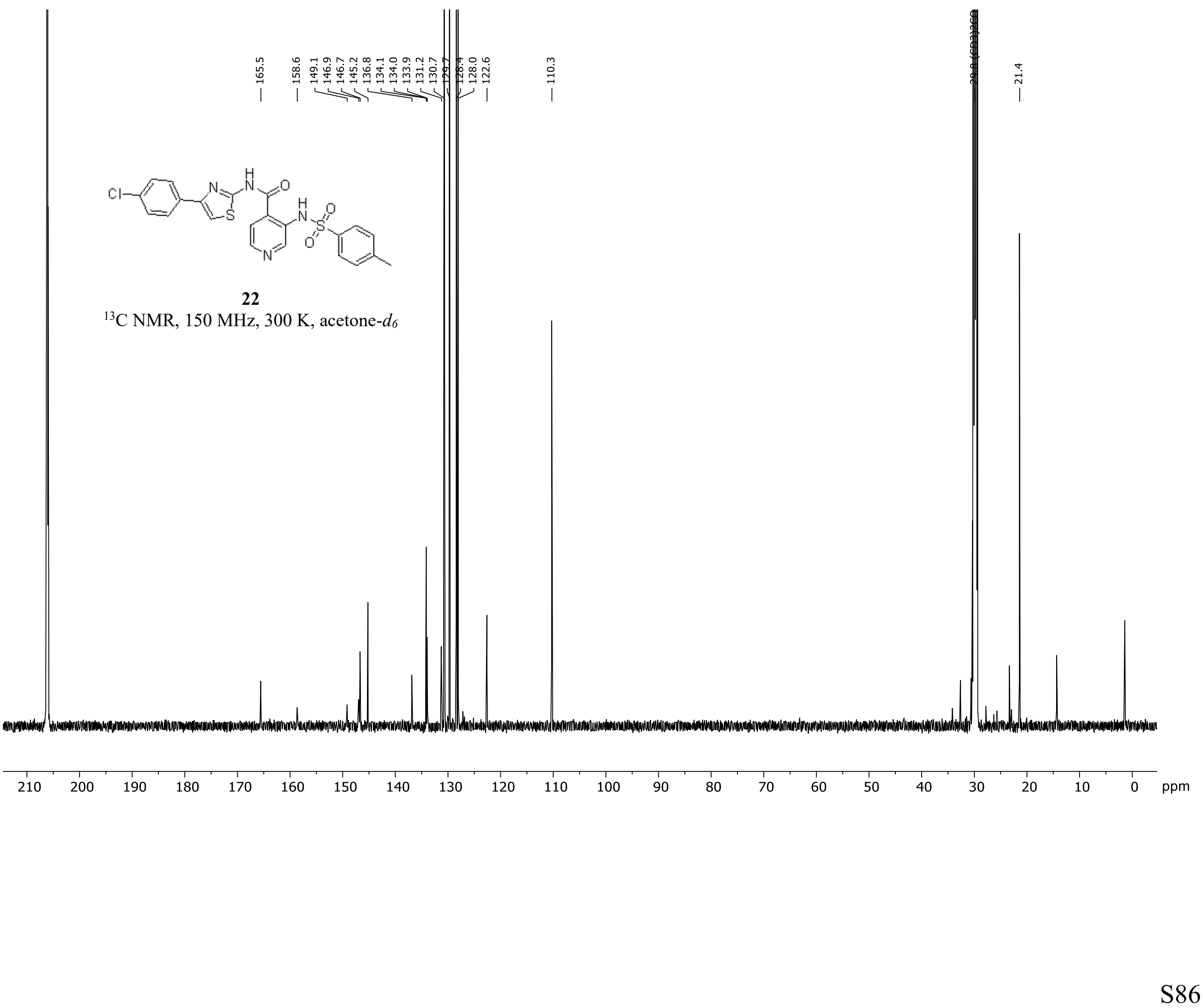

22

^{13}C NMR, 150 MHz, 300 K, acetone-d_6

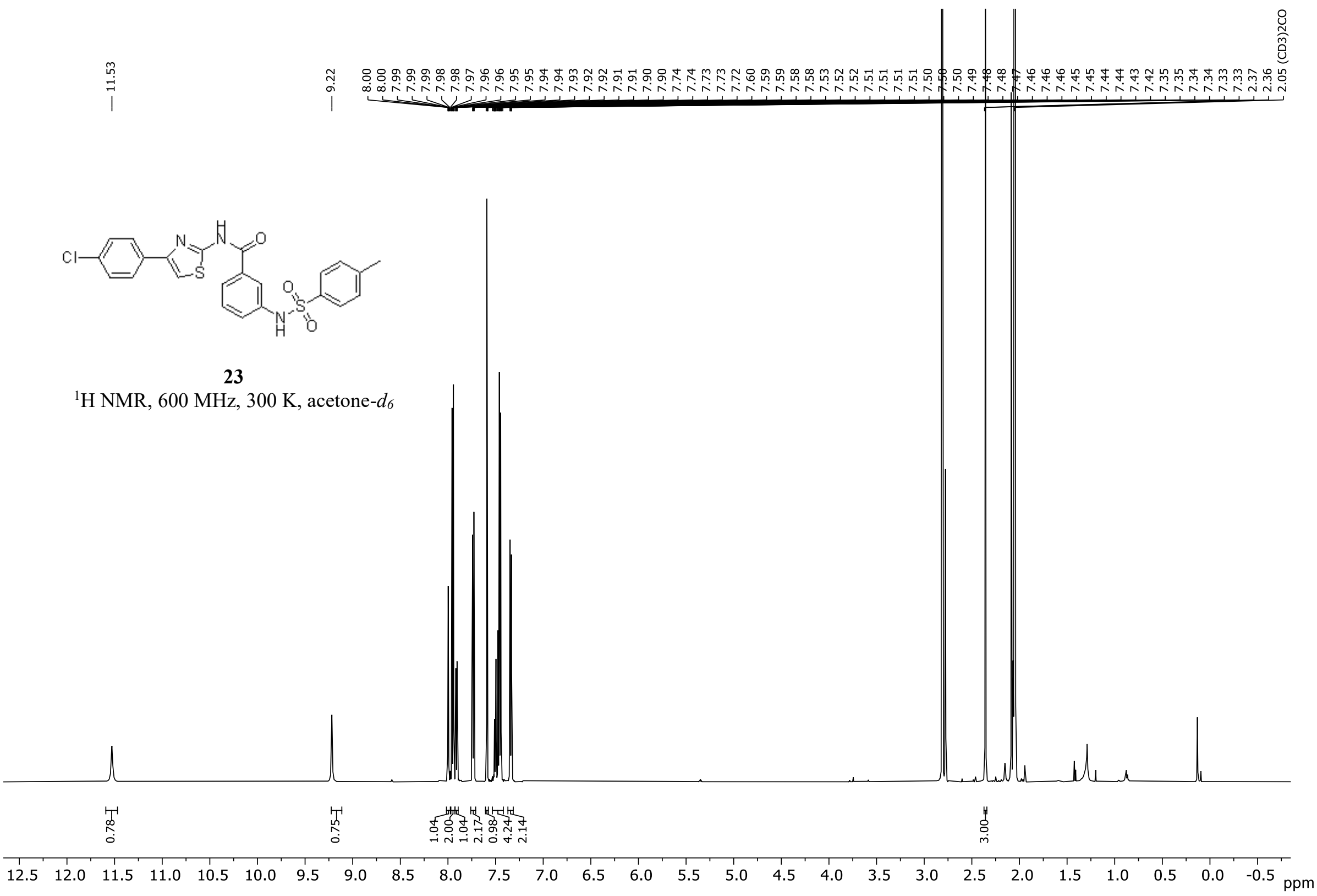

23
^{1}H NMR, 600 MHz, 300 K, acetone-d_6

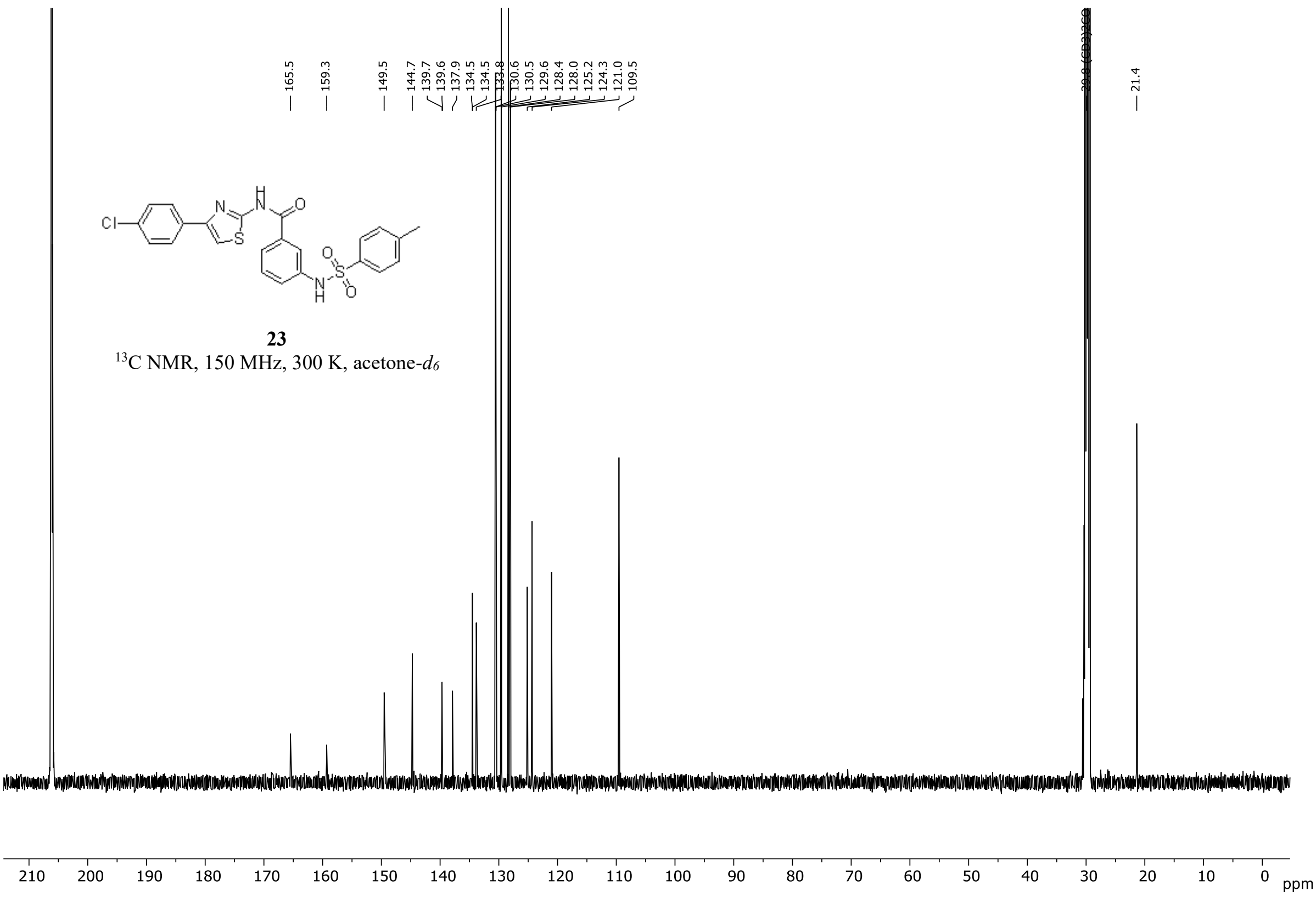

23
^{13}C NMR, 150 MHz, 300 K, acetone-d_6

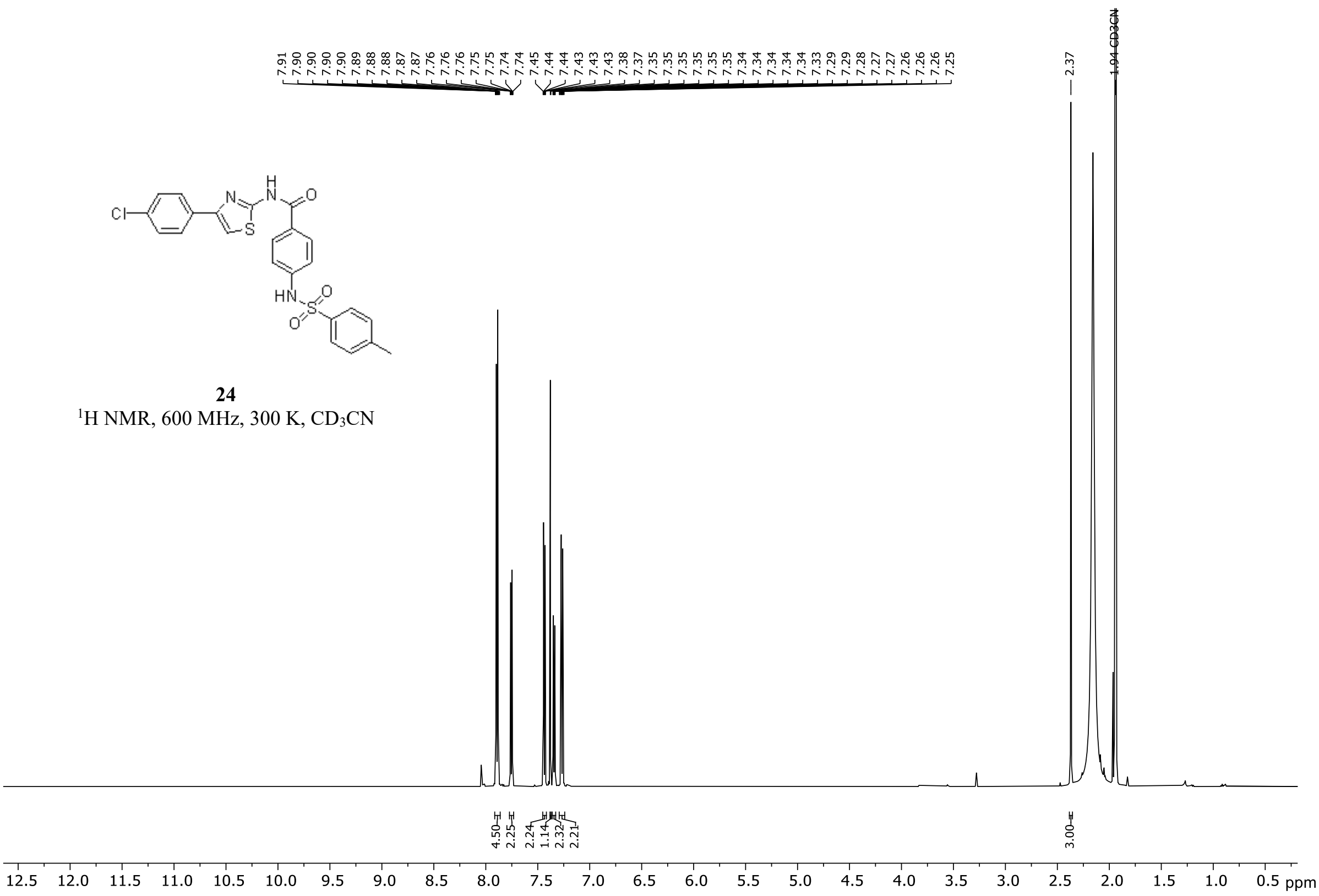

24
^{1}H NMR, 600 MHz, 300 K, CD_3CN

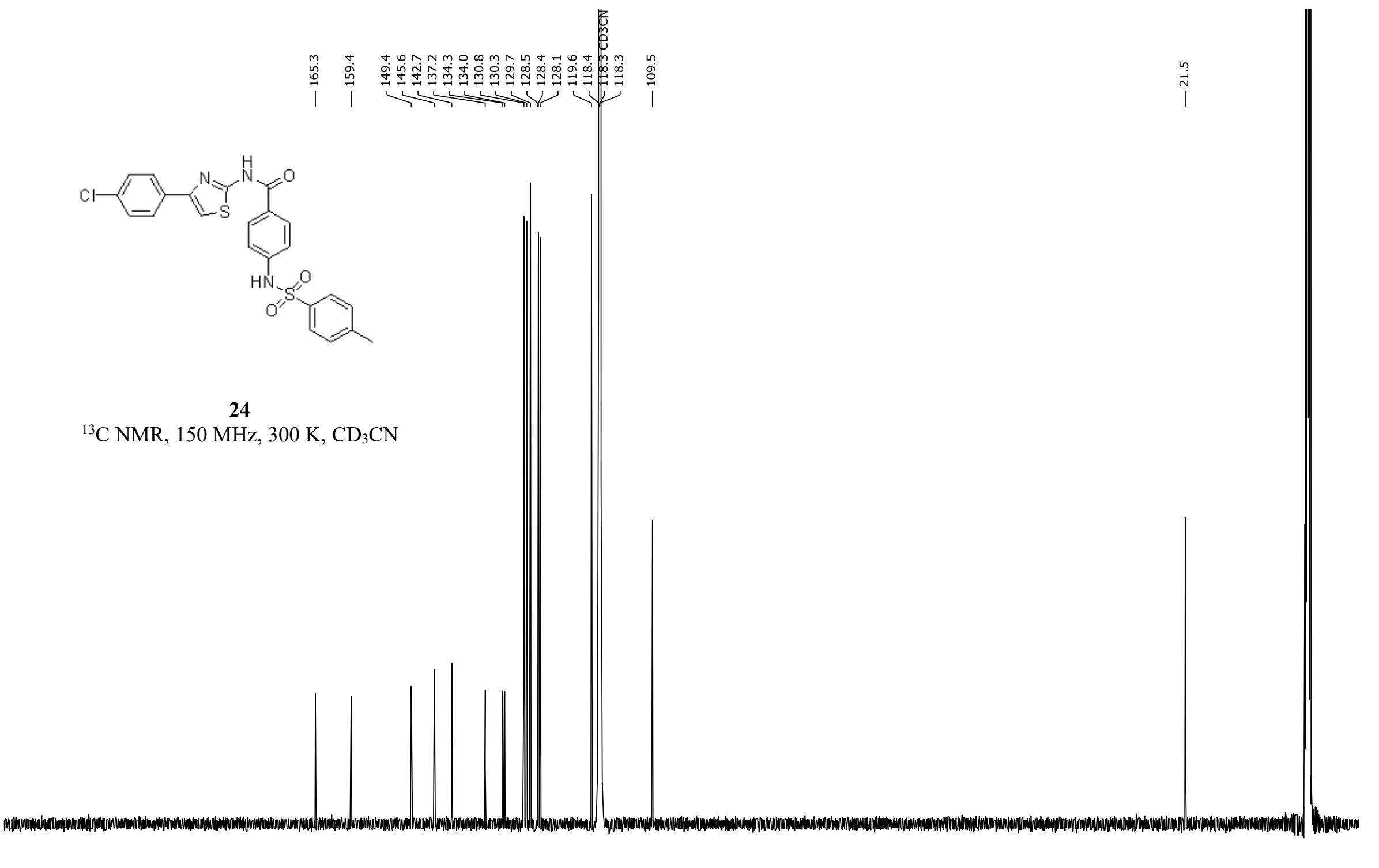

24
^{13}C NMR, 150 MHz, 300 K, CD_3CN

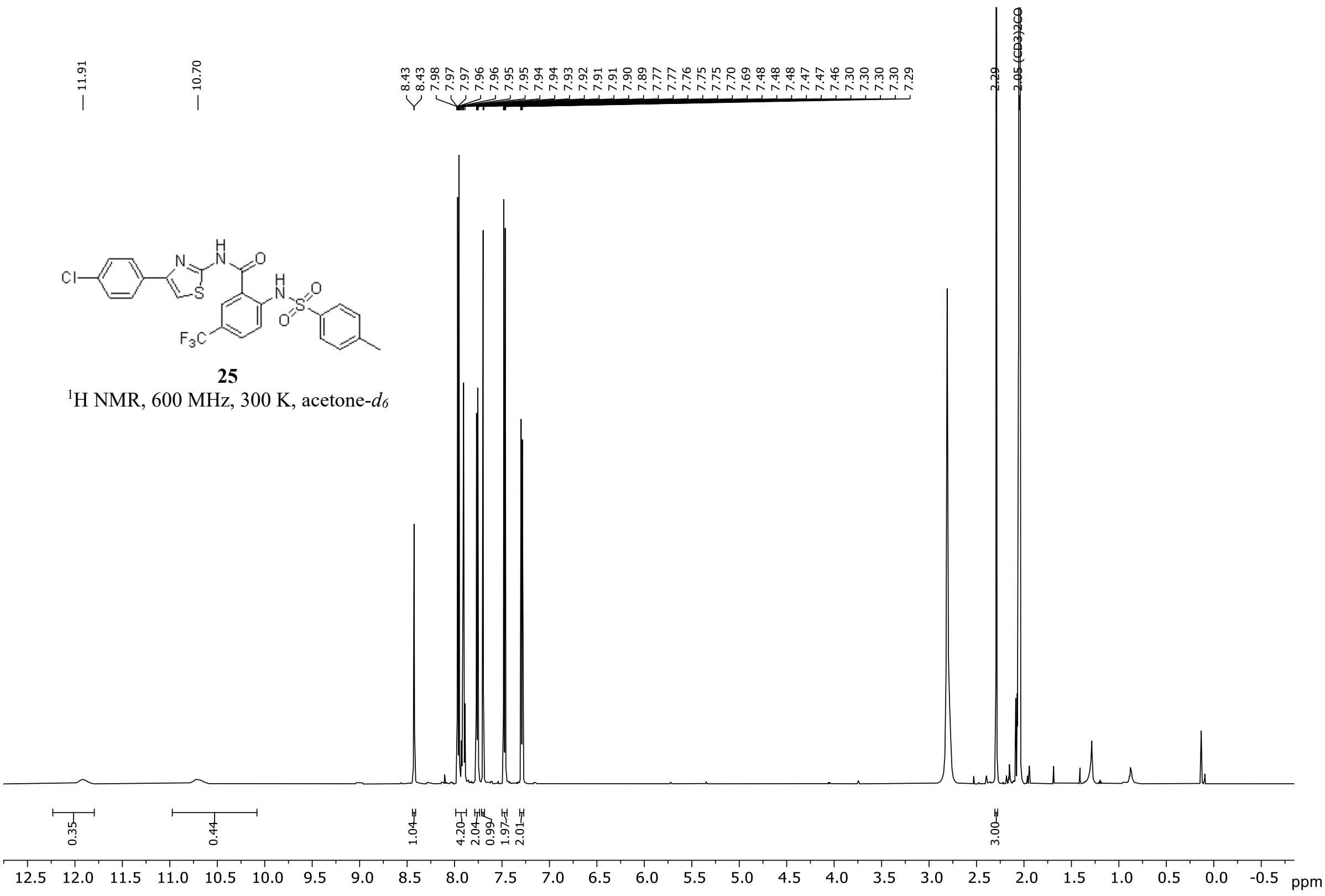

25

^{1}H NMR, 600 MHz, 300 K, acetone-d_6

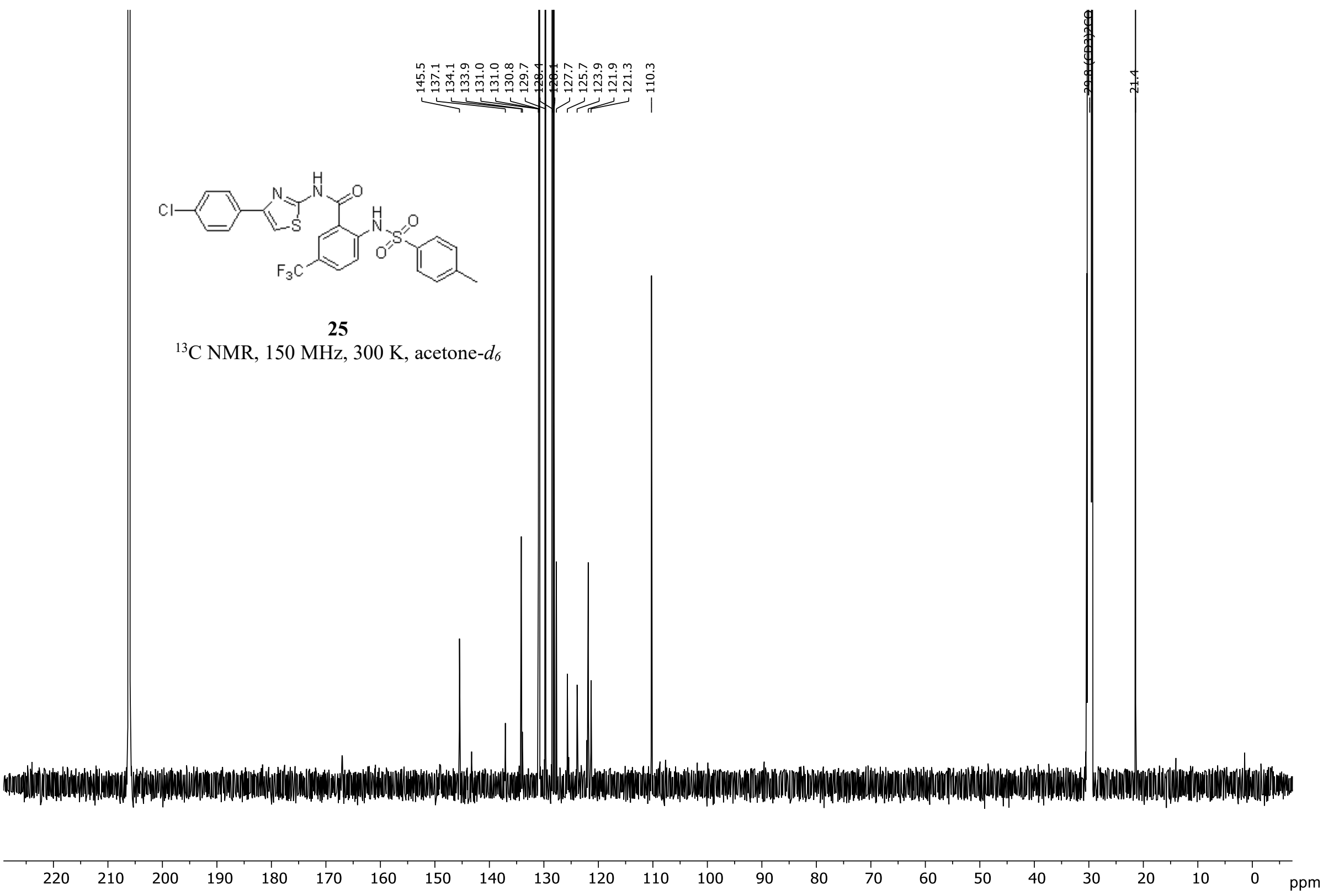

25

^{13}C NMR, 150 MHz, 300 K, acetone-d_6

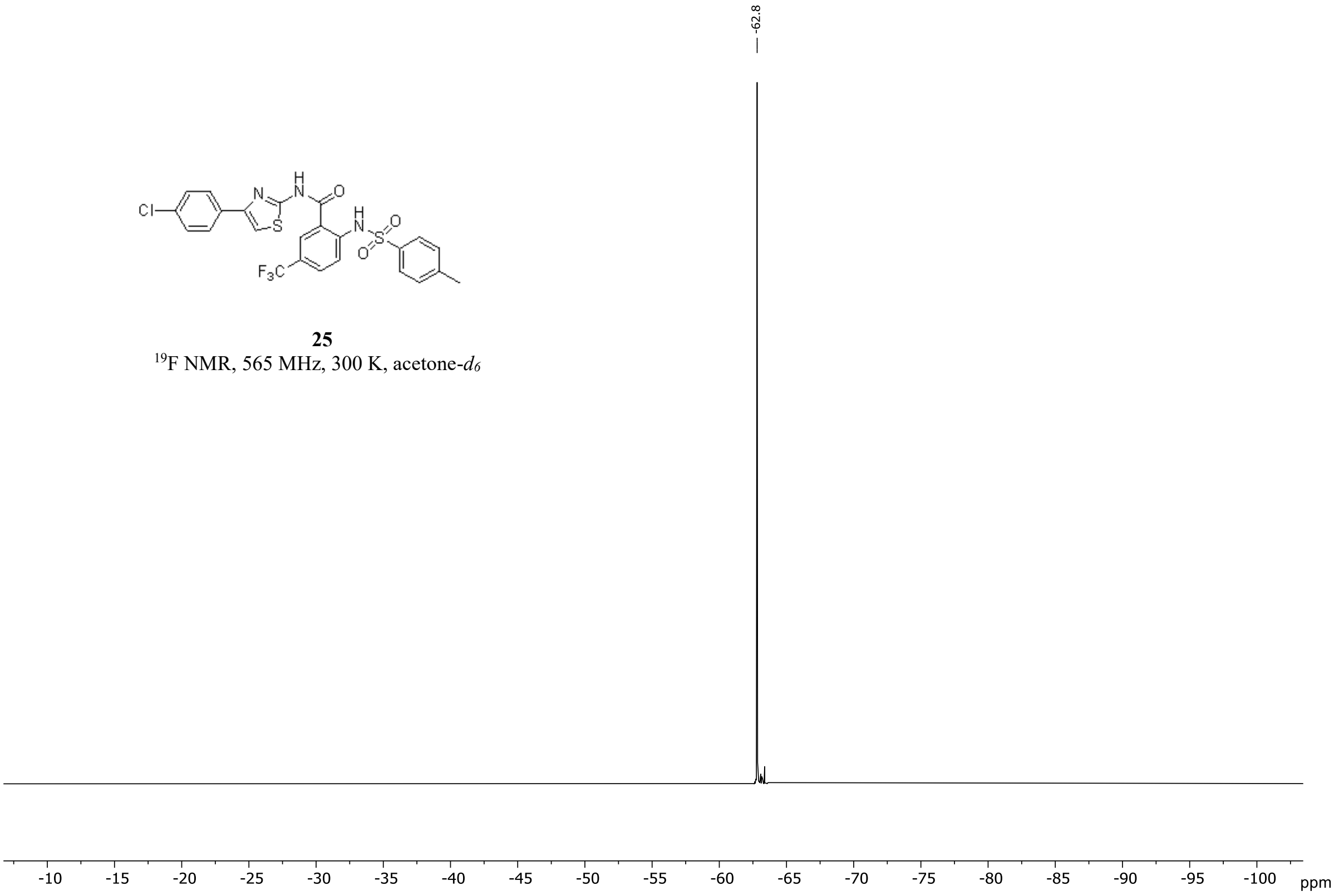

25
^{19}F NMR, 565 MHz, 300 K, acetone-*d*$_6$

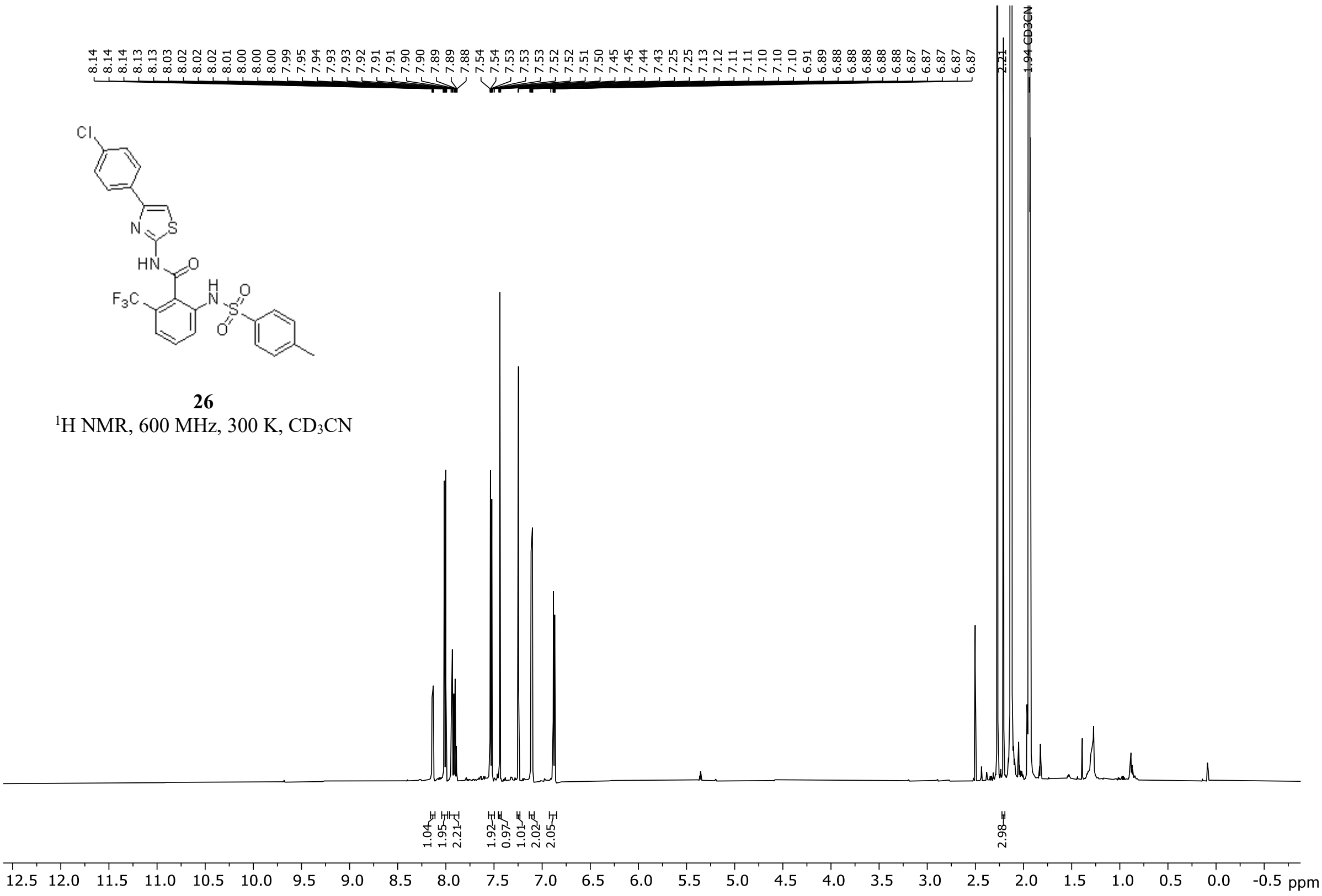

26

^{1}H NMR, 600 MHz, 300 K, CD_3CN

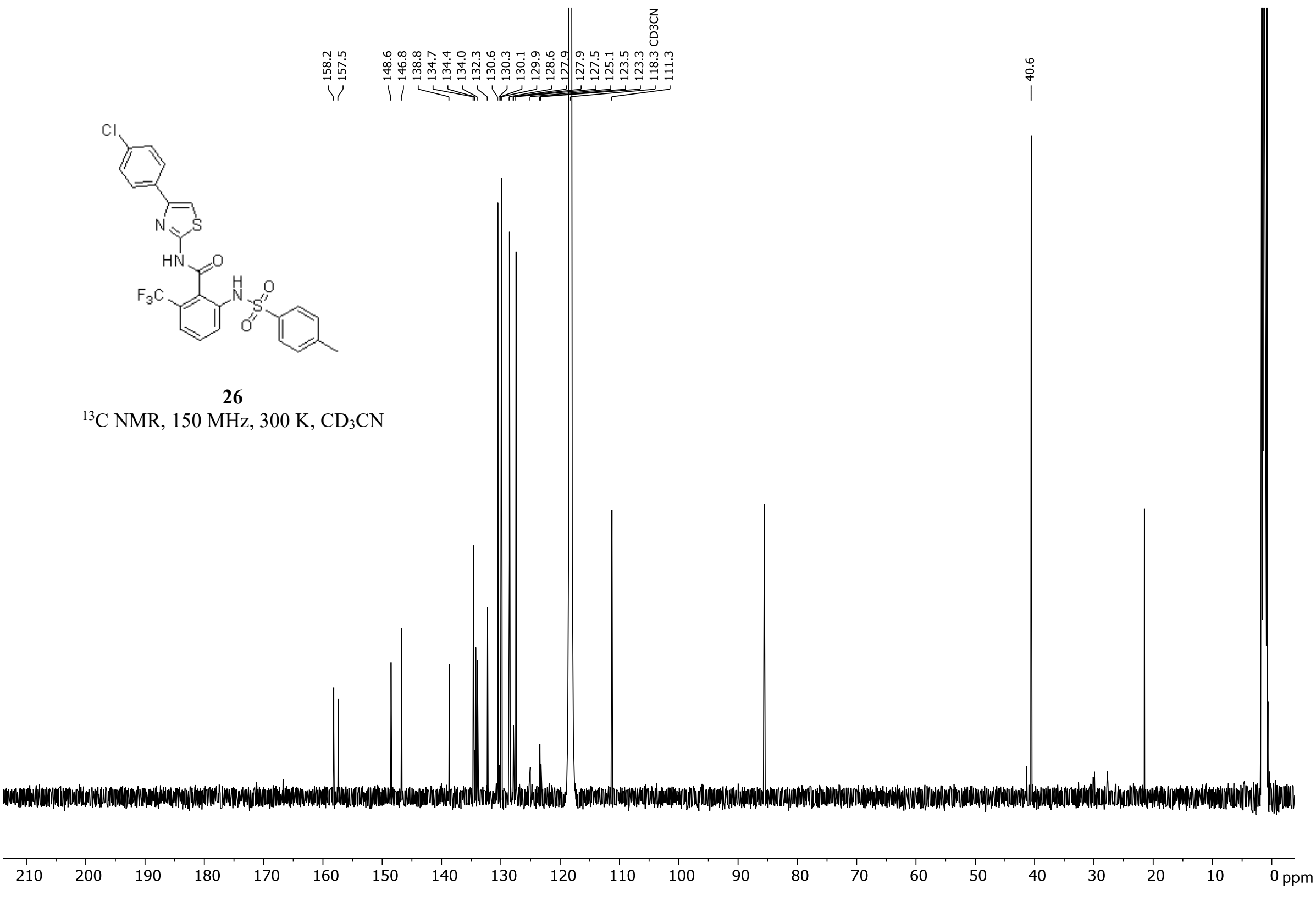

26

^{13}C NMR, 150 MHz, 300 K, CD_3CN

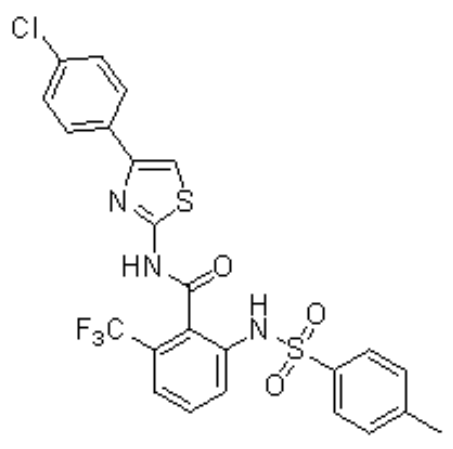

26

^{19}F NMR, 565 MHz, 300 K, CD_3CN

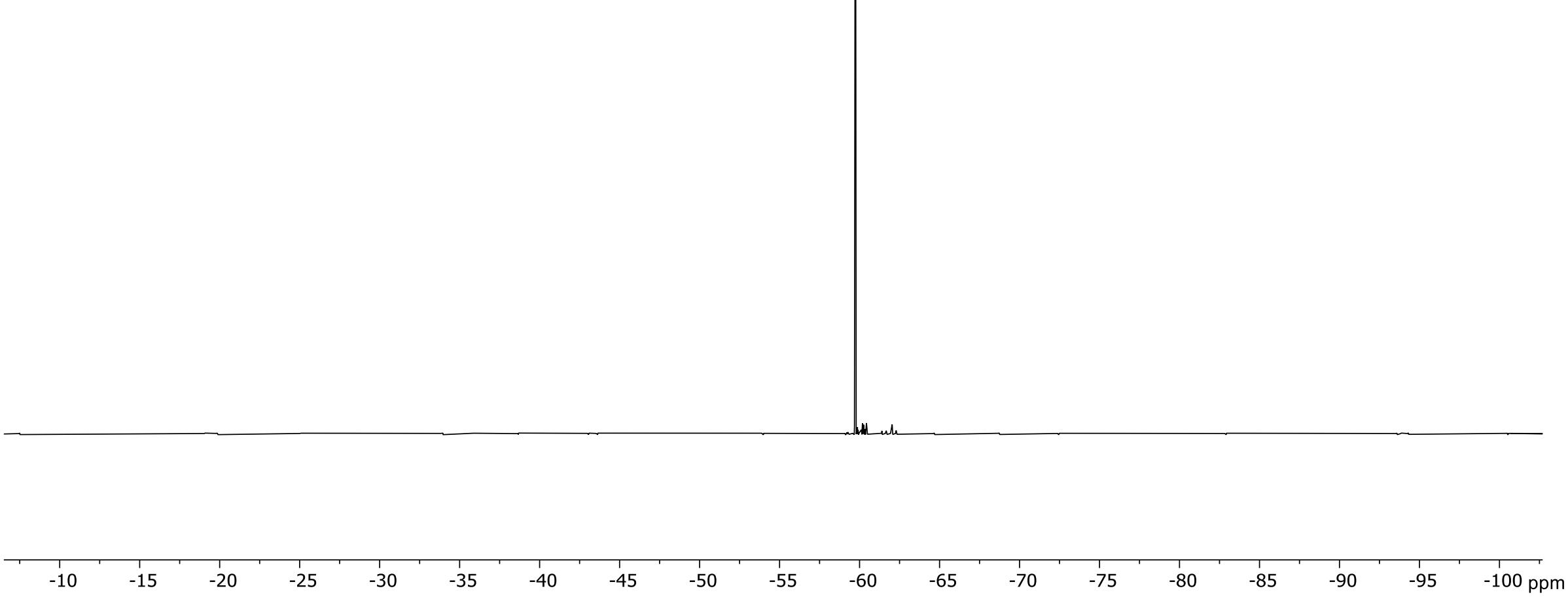

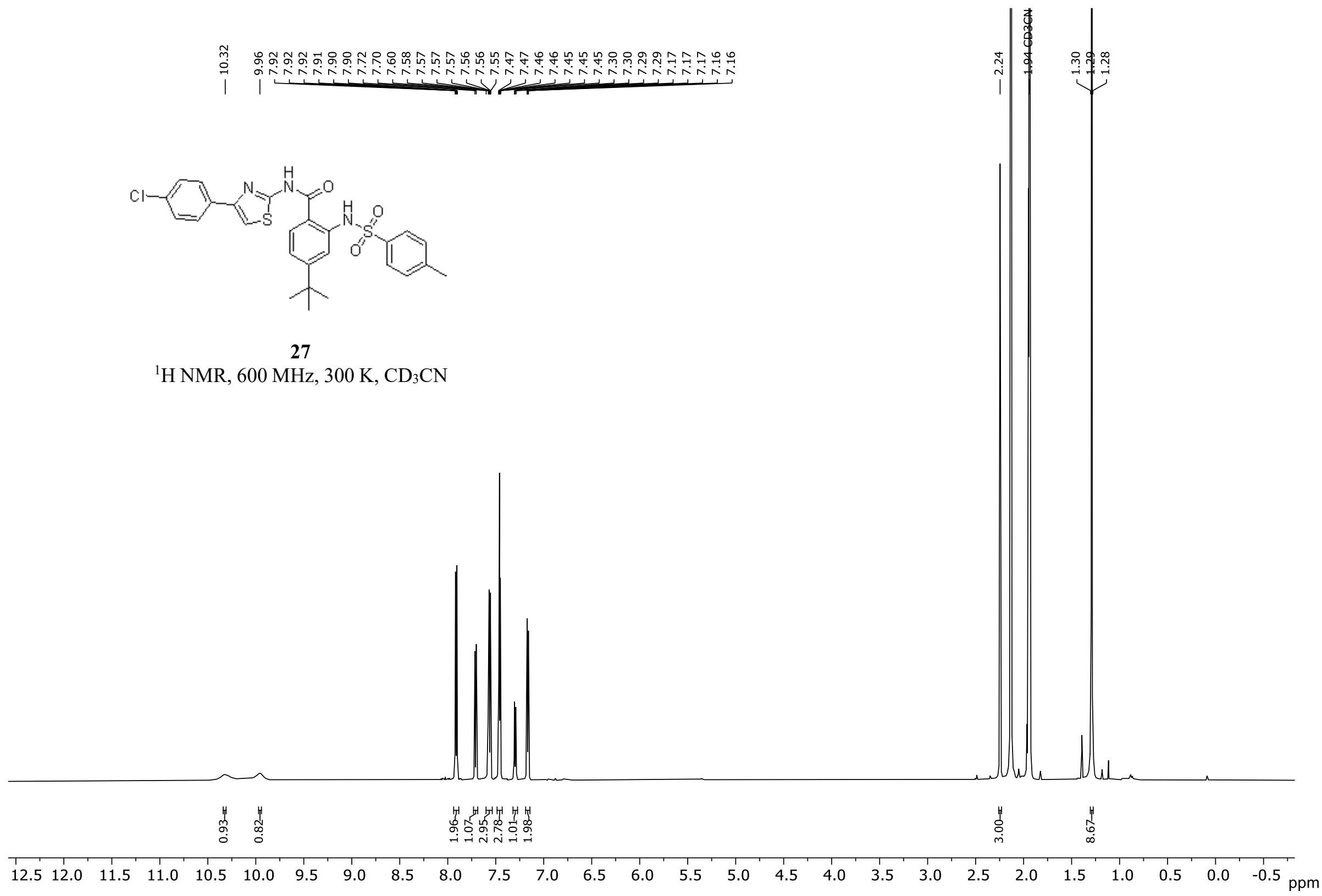

27

^{1}H NMR, 600 MHz, 300 K, CD_3CN

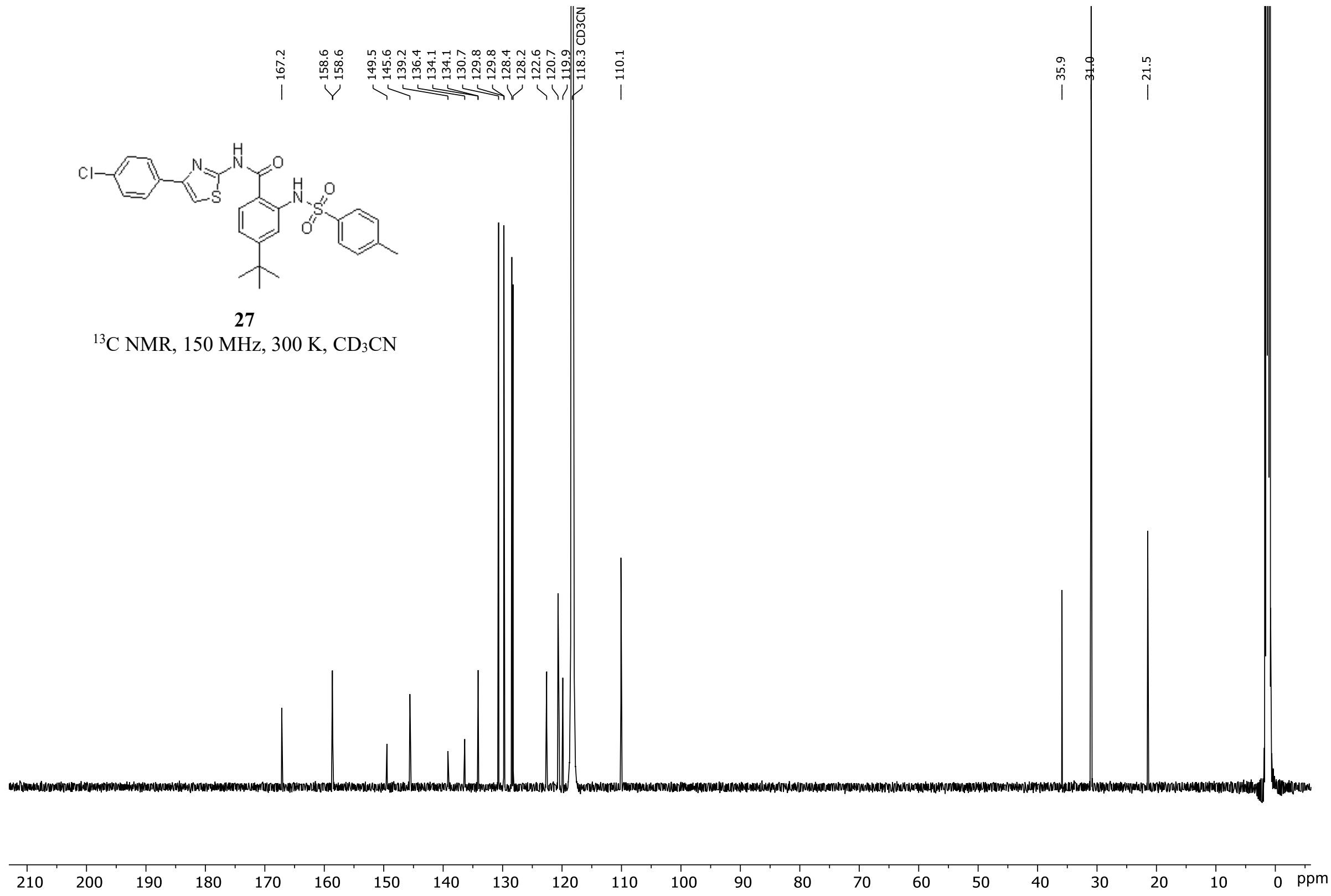

27

^{13}C NMR, 150 MHz, 300 K, CD_3CN

28
^{1}H NMR, 600 MHz, 300 K, DMSO-d_6

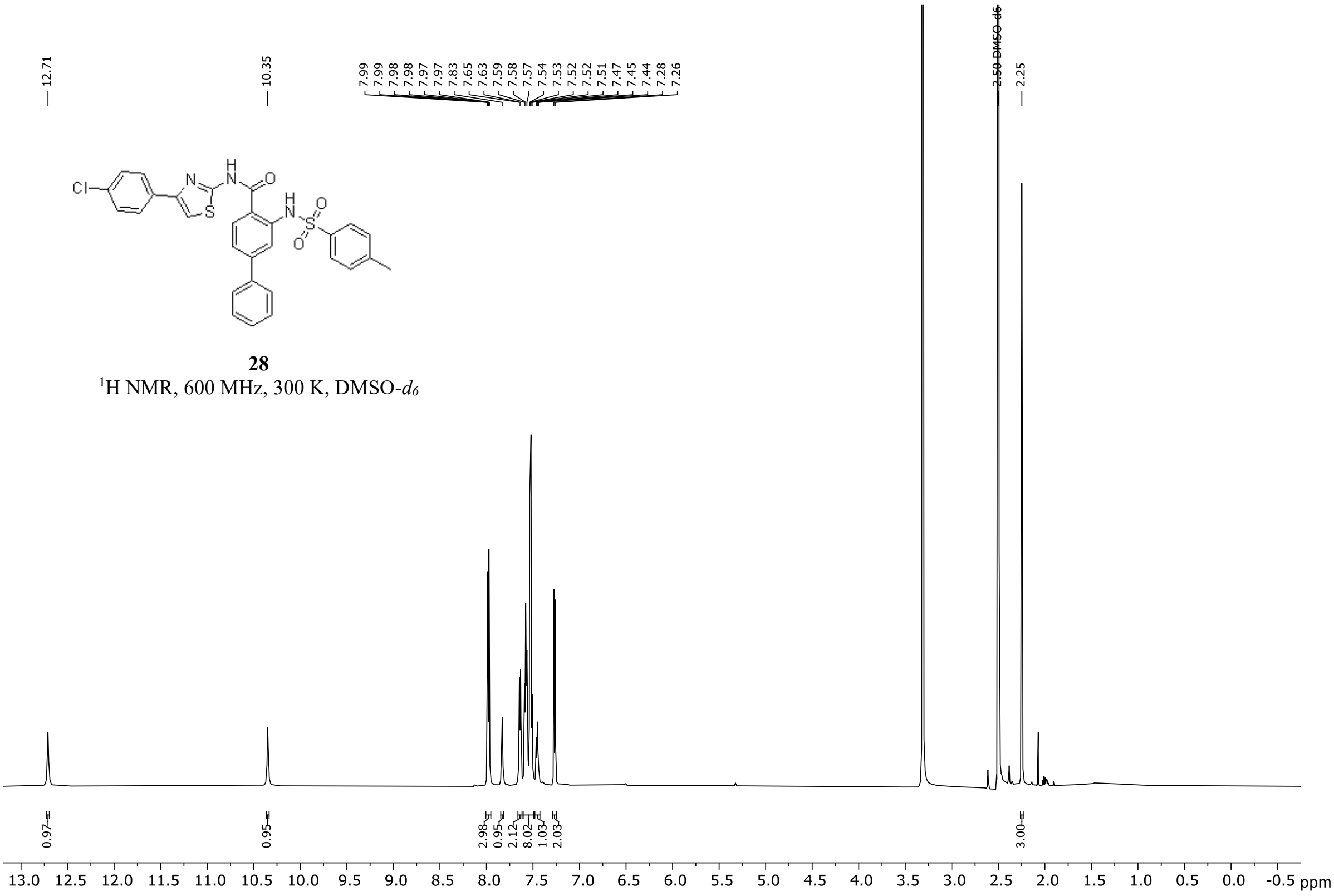

28
^{13}C NMR, 150 MHz, 300 K, DMSO-d_6

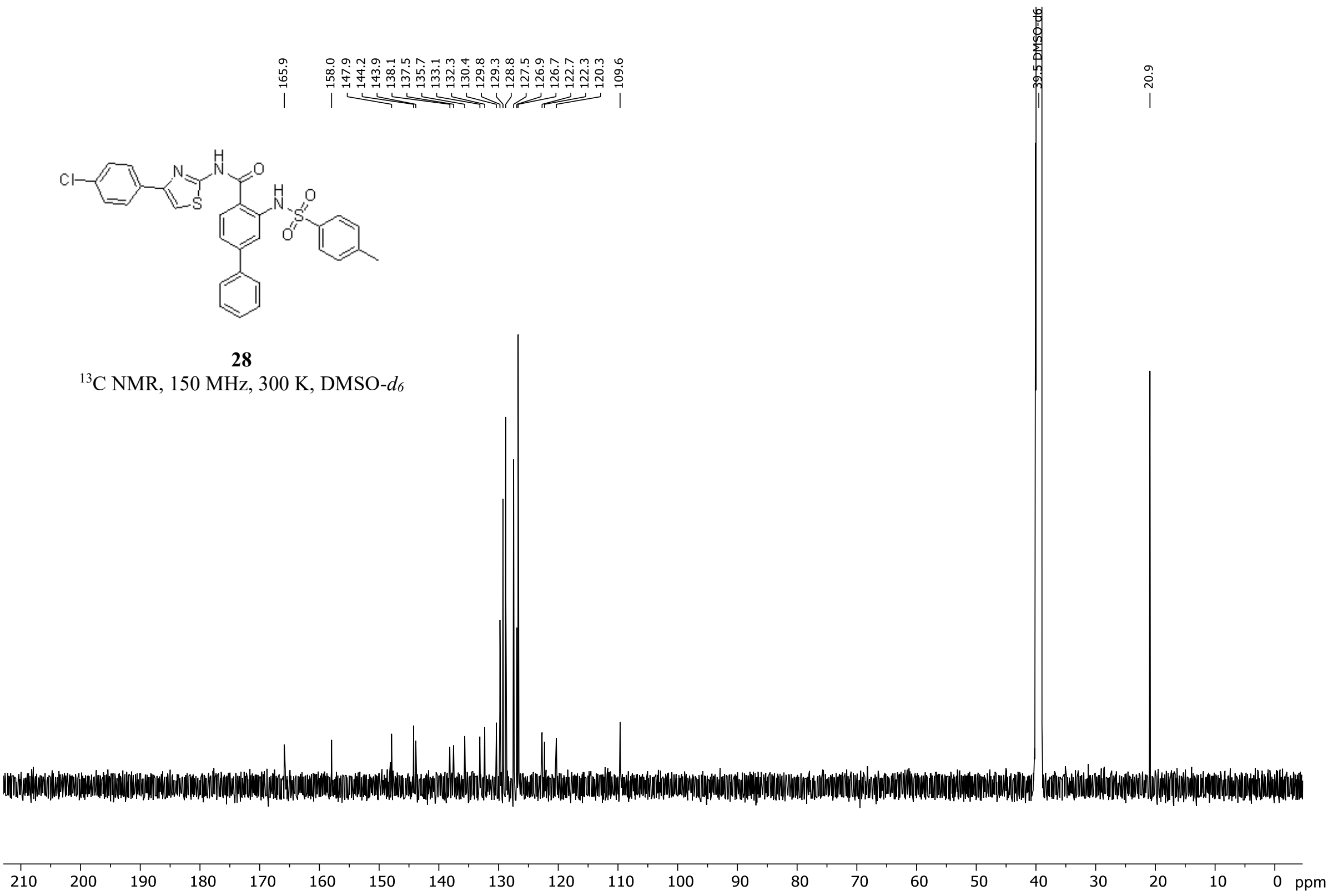

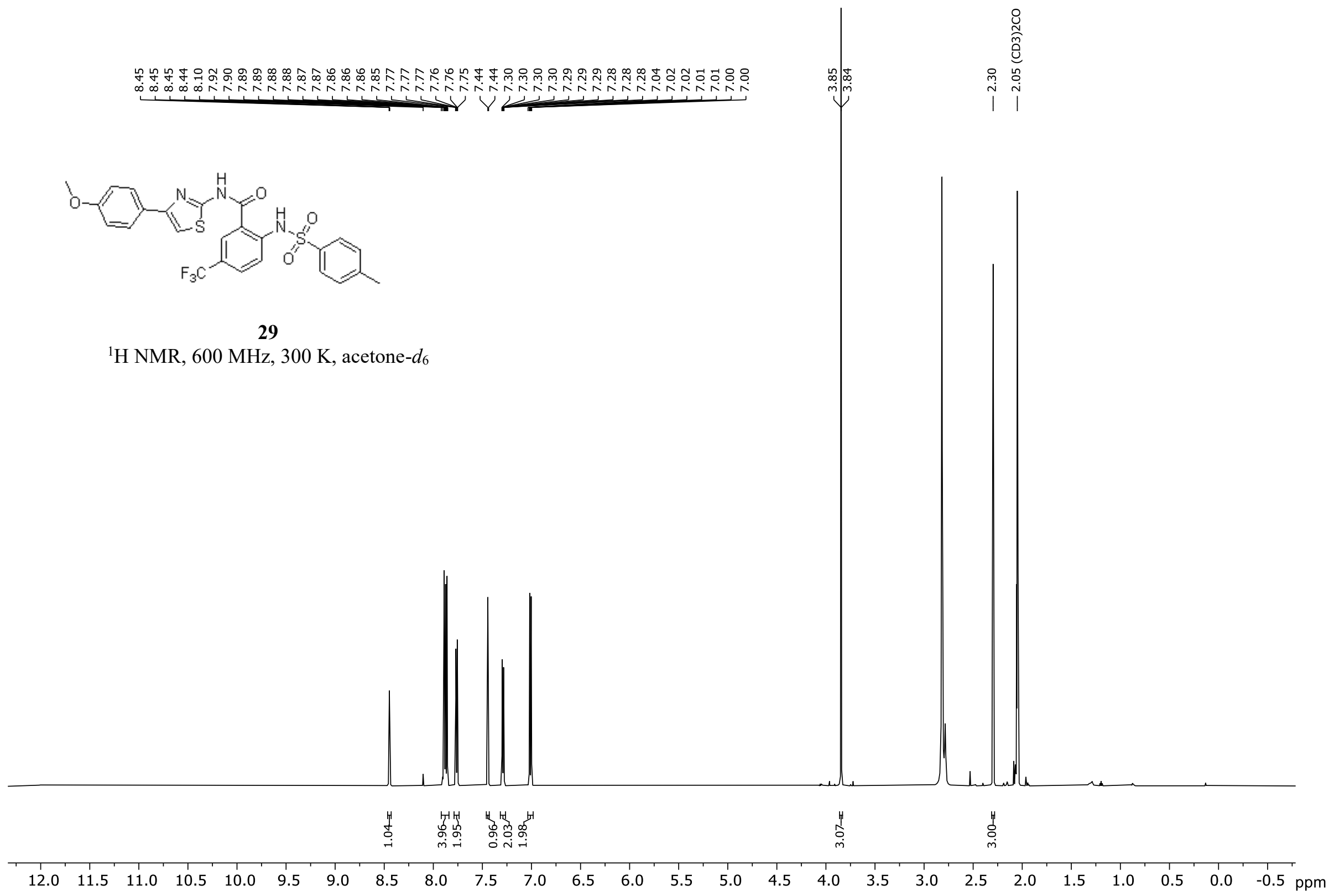

29

^{1}H NMR, 600 MHz, 300 K, acetone-d_6

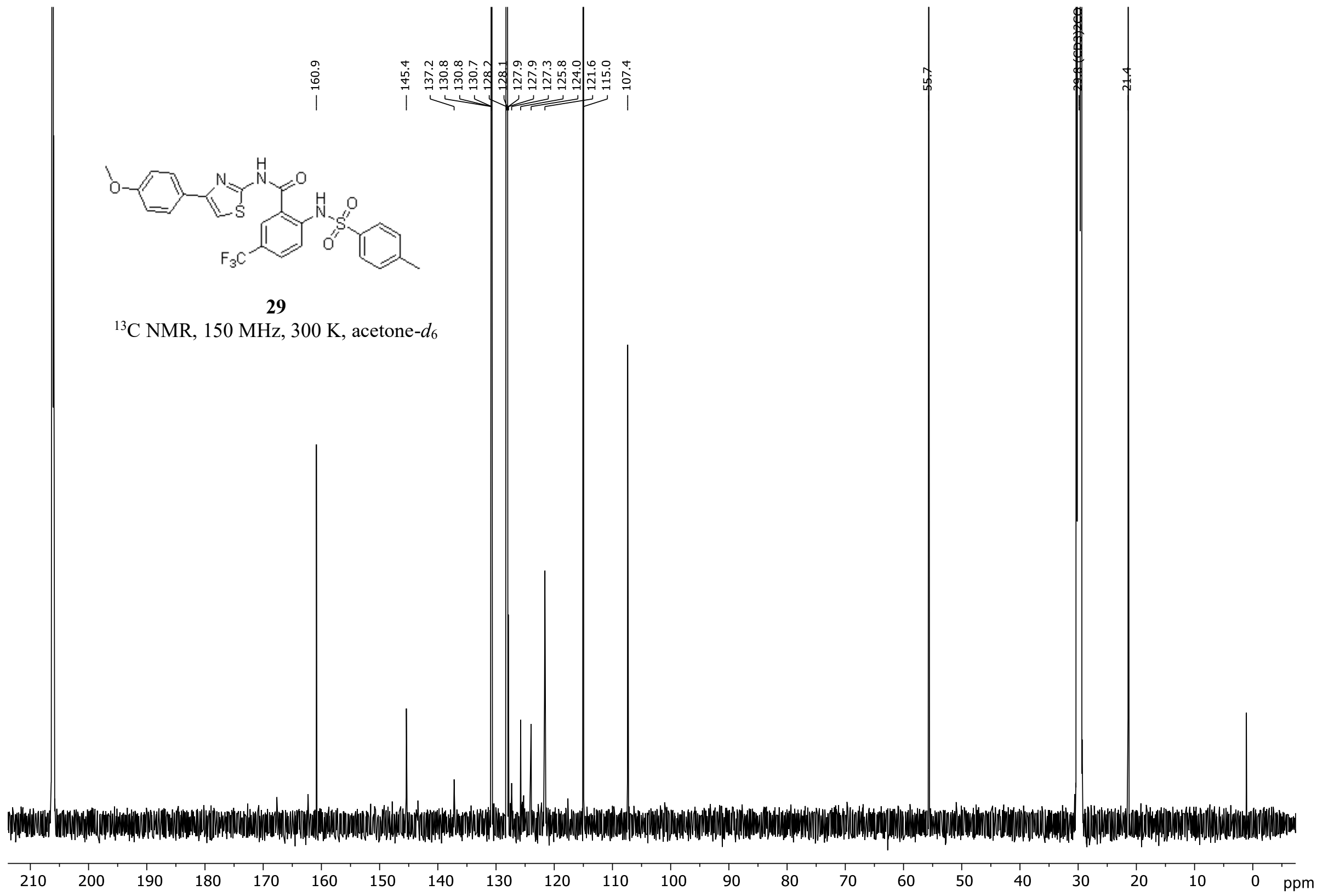

29

^{13}C NMR, 150 MHz, 300 K, acetone-d_6

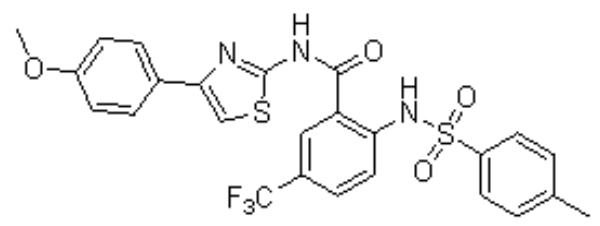

29
^{19}F NMR, 565 MHz, 300 K, acetone-d_6

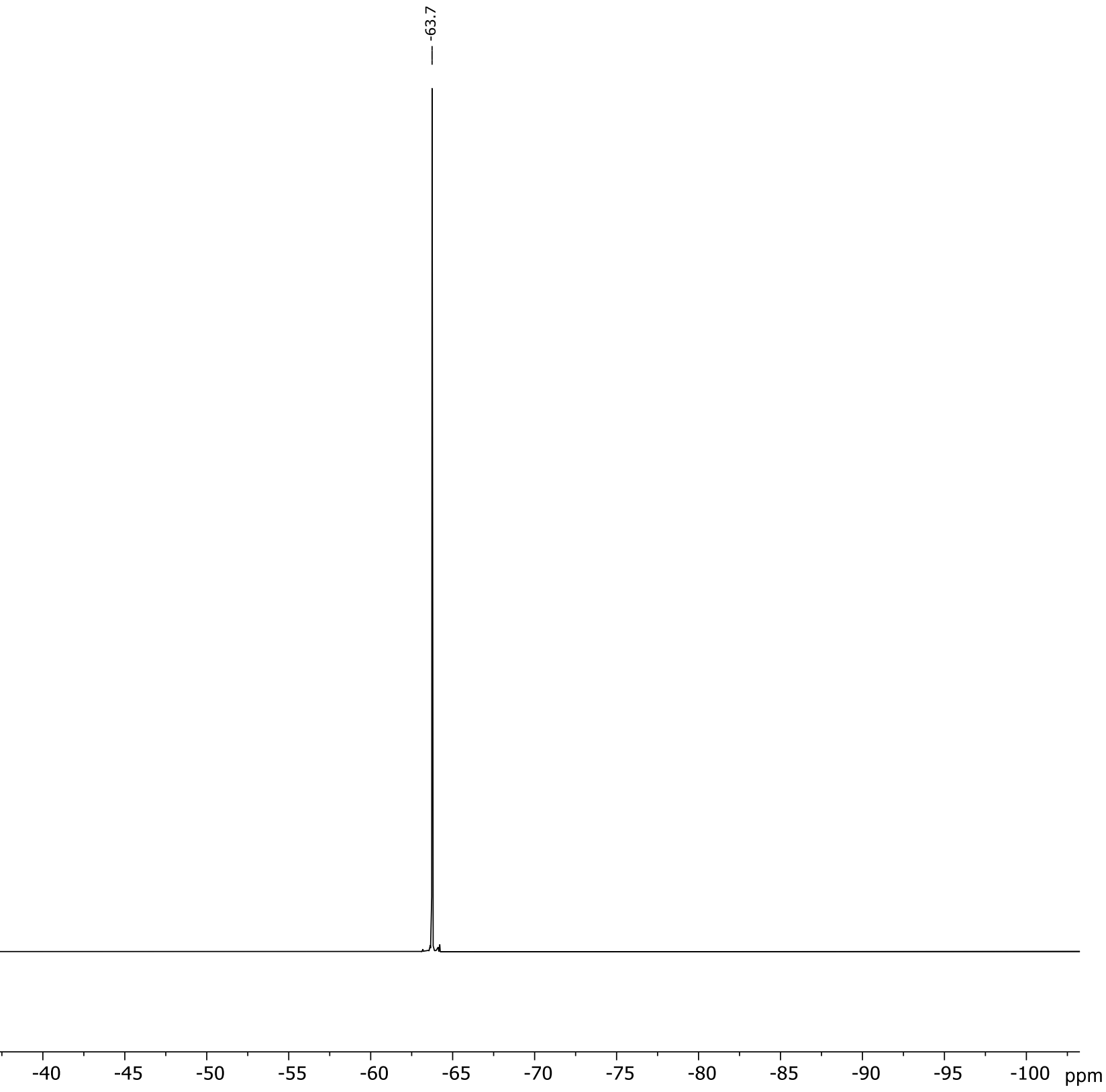

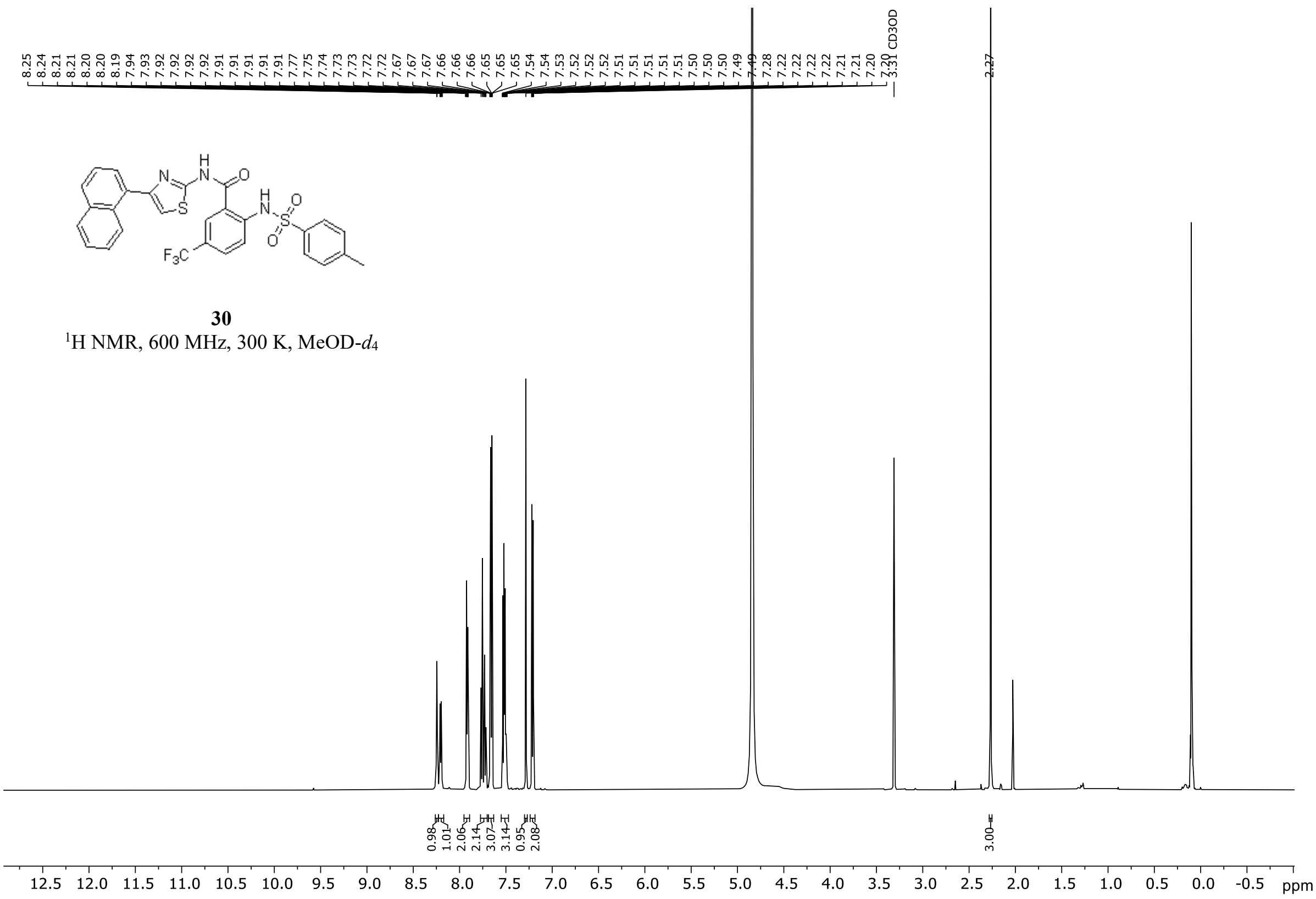

30
^{1}H NMR, 600 MHz, 300 K, MeOD-d_4

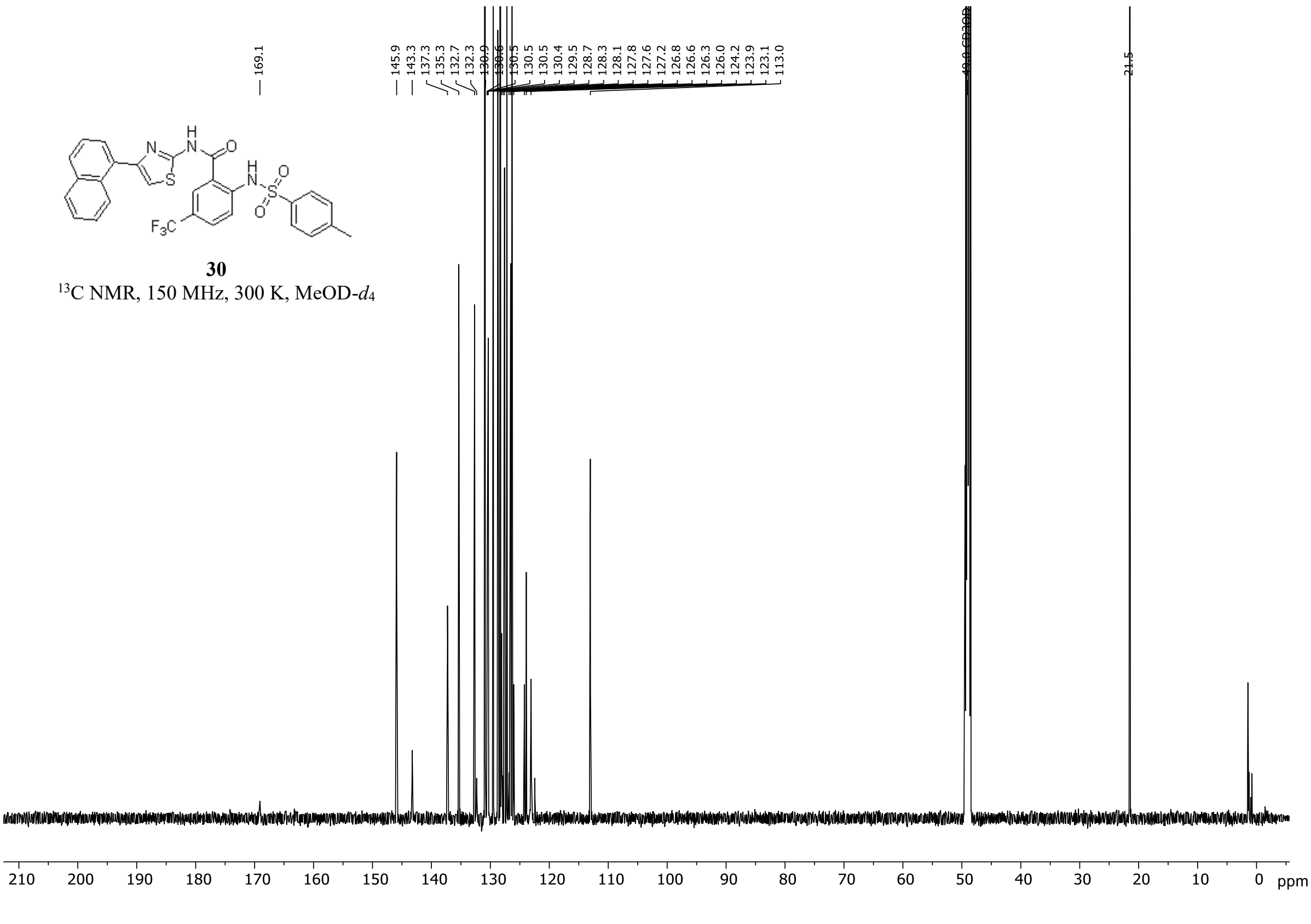

30
^{13}C NMR, 150 MHz, 300 K, MeOD-d_4

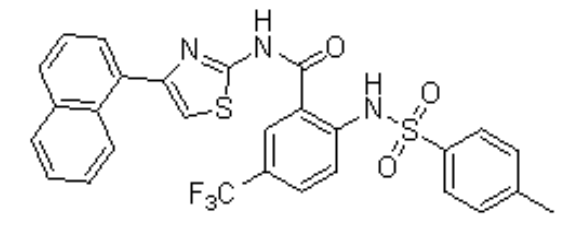

30
^{19}F NMR, 565 MHz, 300 K, MeOD-d_4

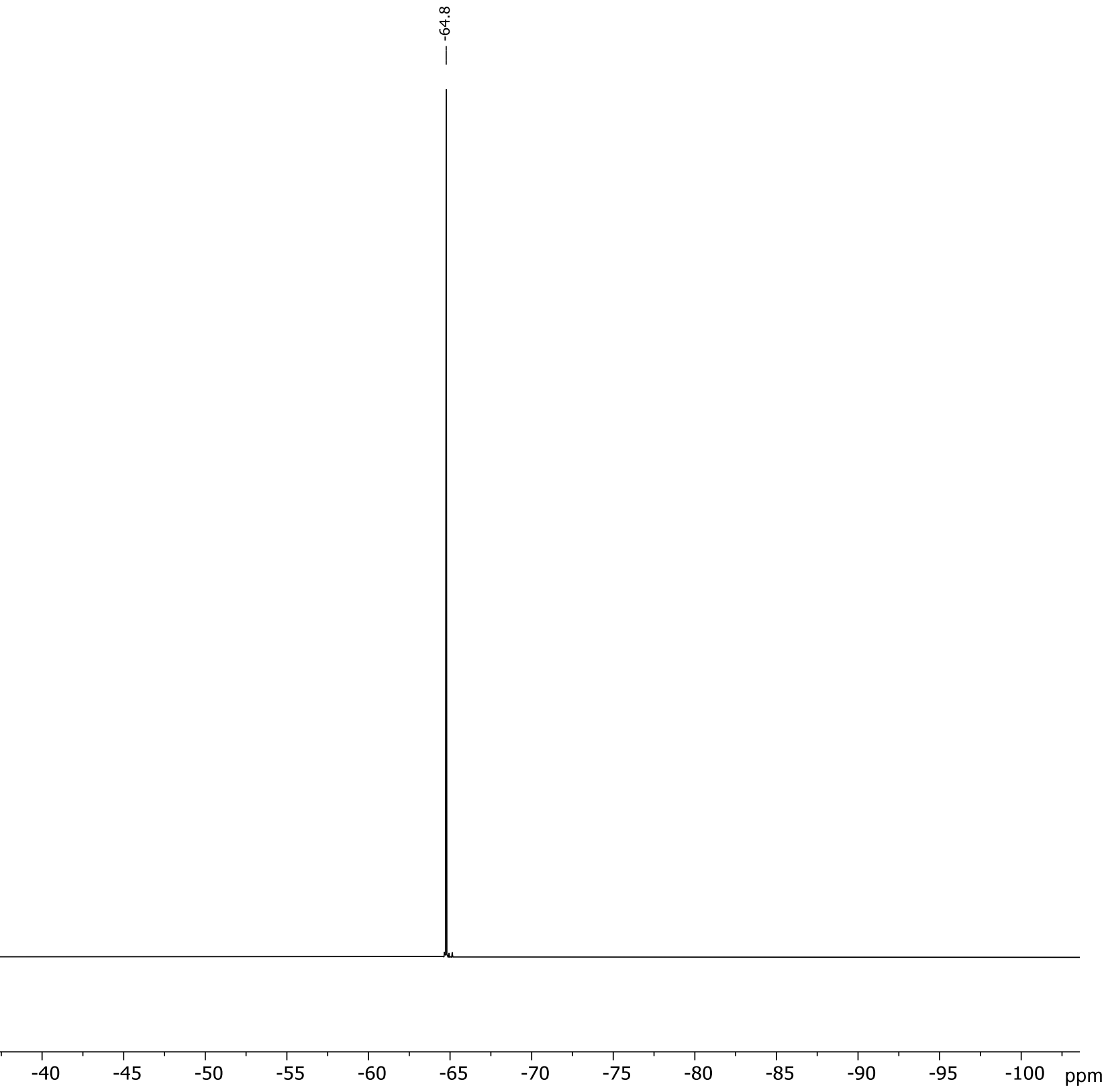

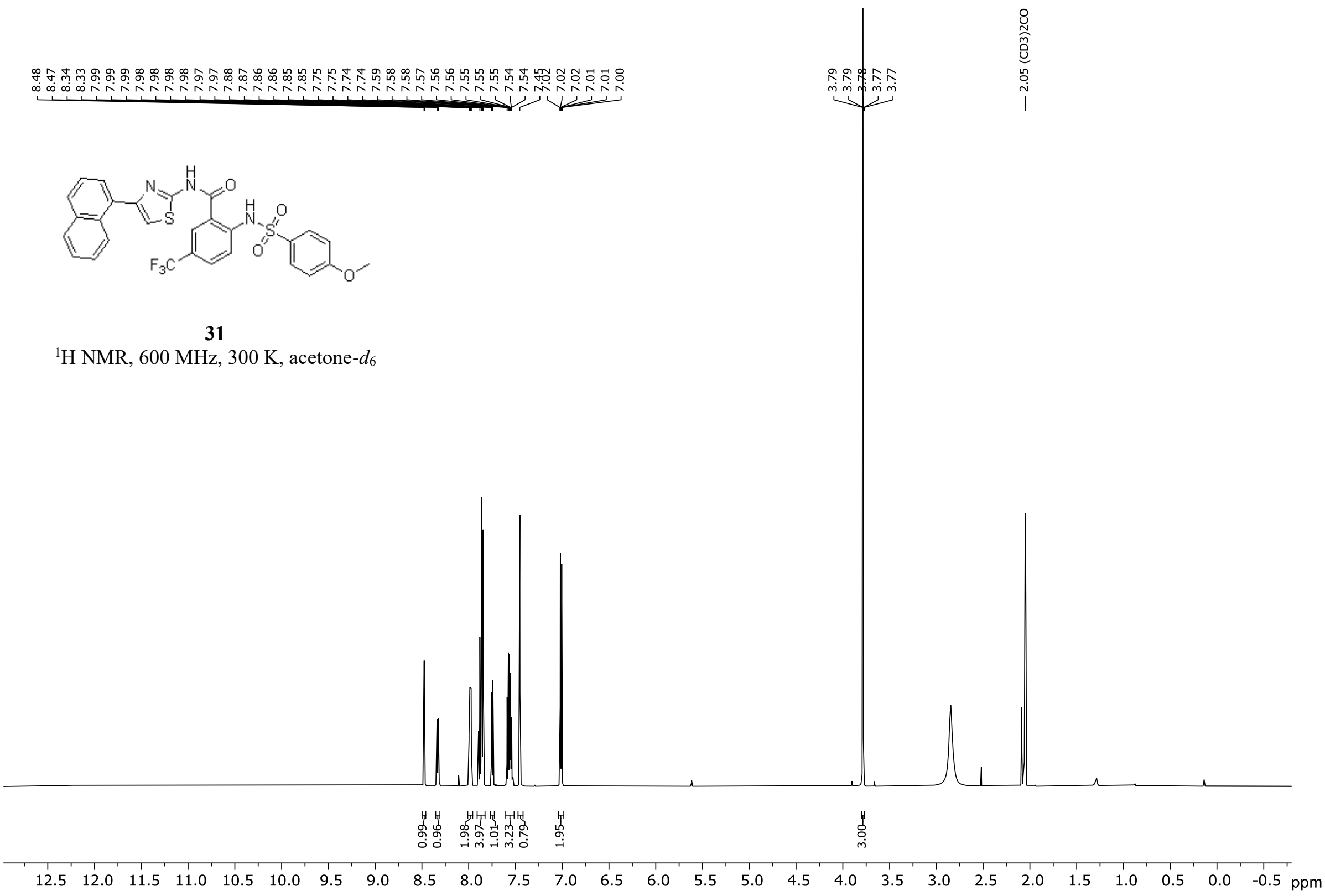

31
^{1}H NMR, 600 MHz, 300 K, acetone-d_6

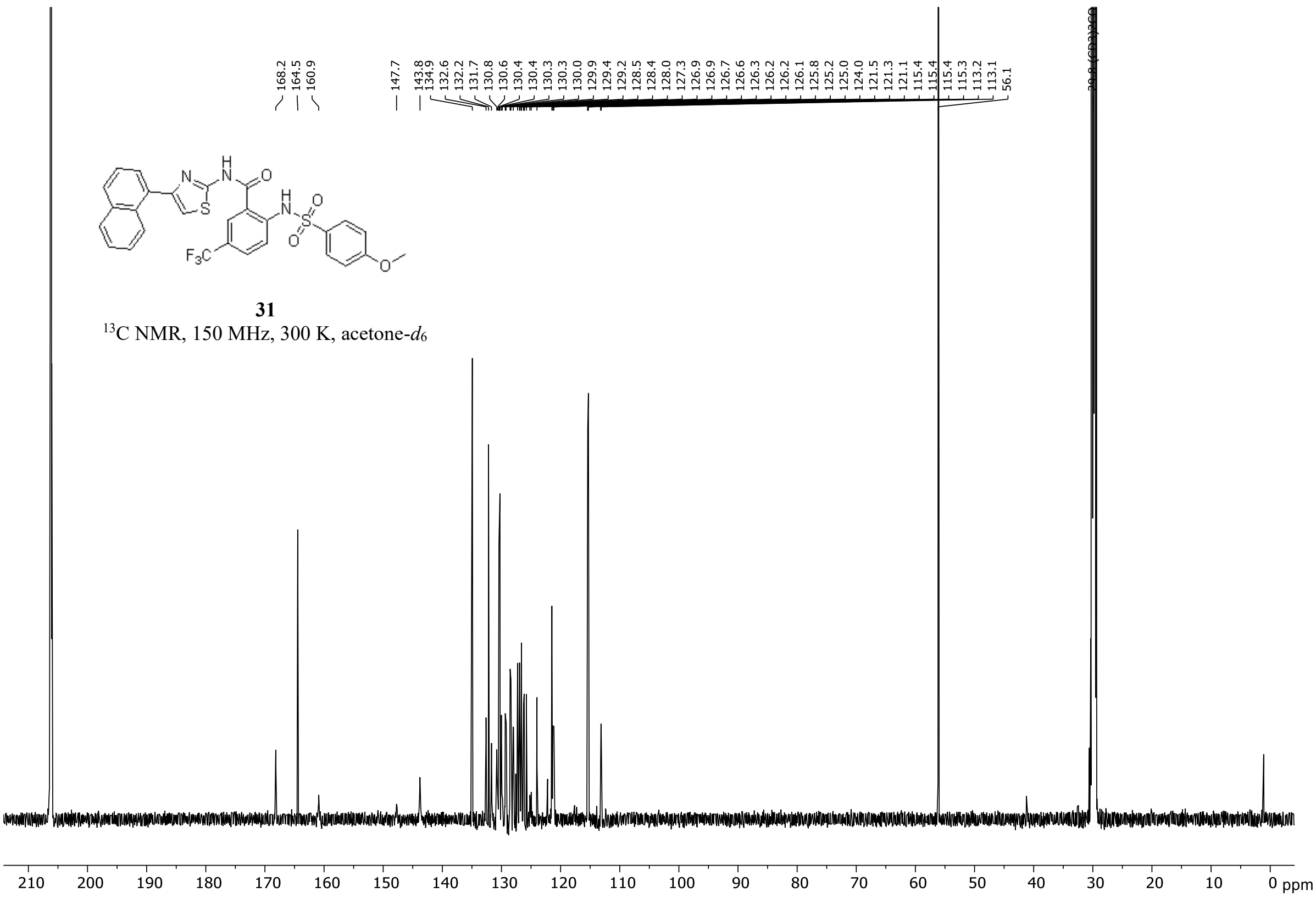

31
^{13}C NMR, 150 MHz, 300 K, acetone-d_6

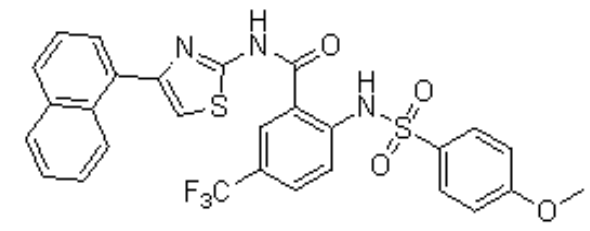

31

^{19}F NMR, 565 MHz, 300 K, acetone-d_6

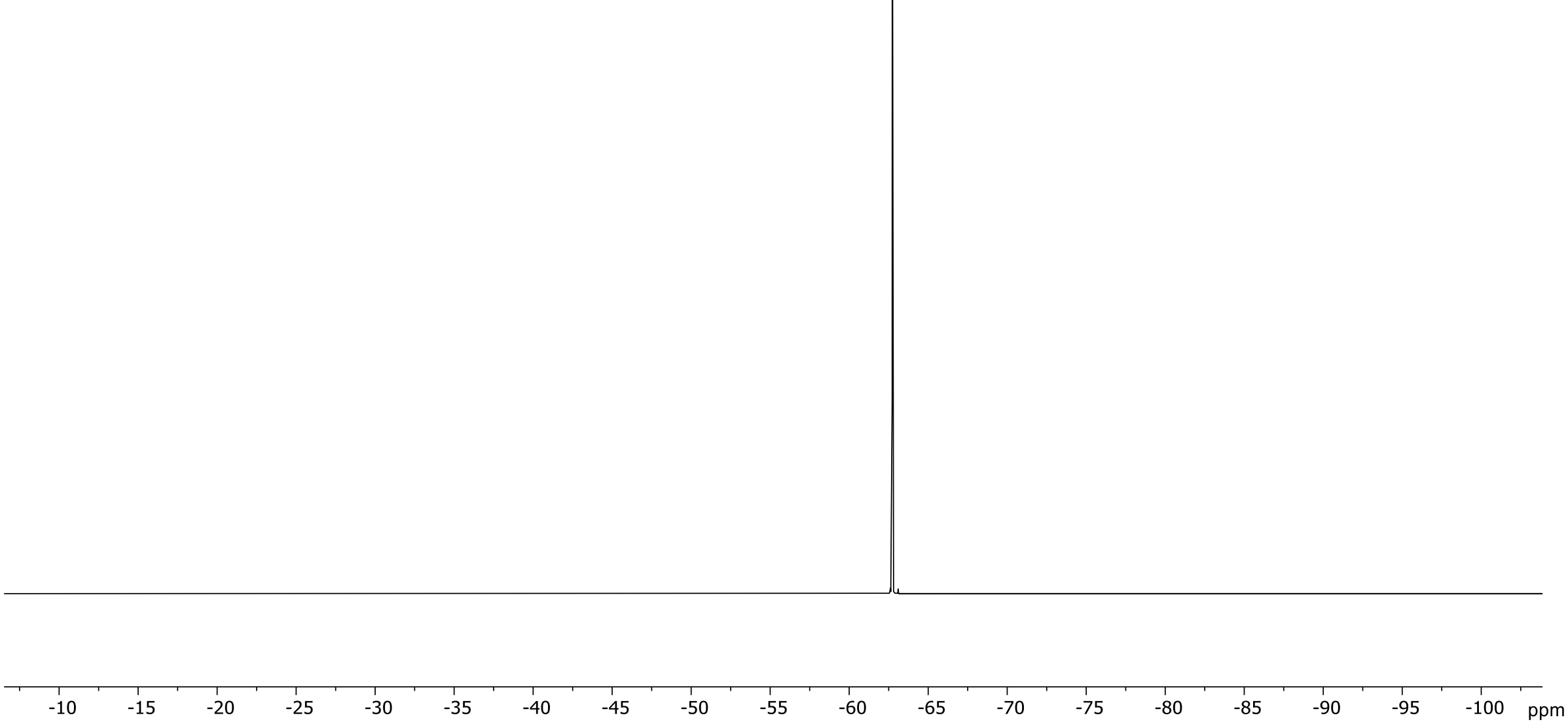

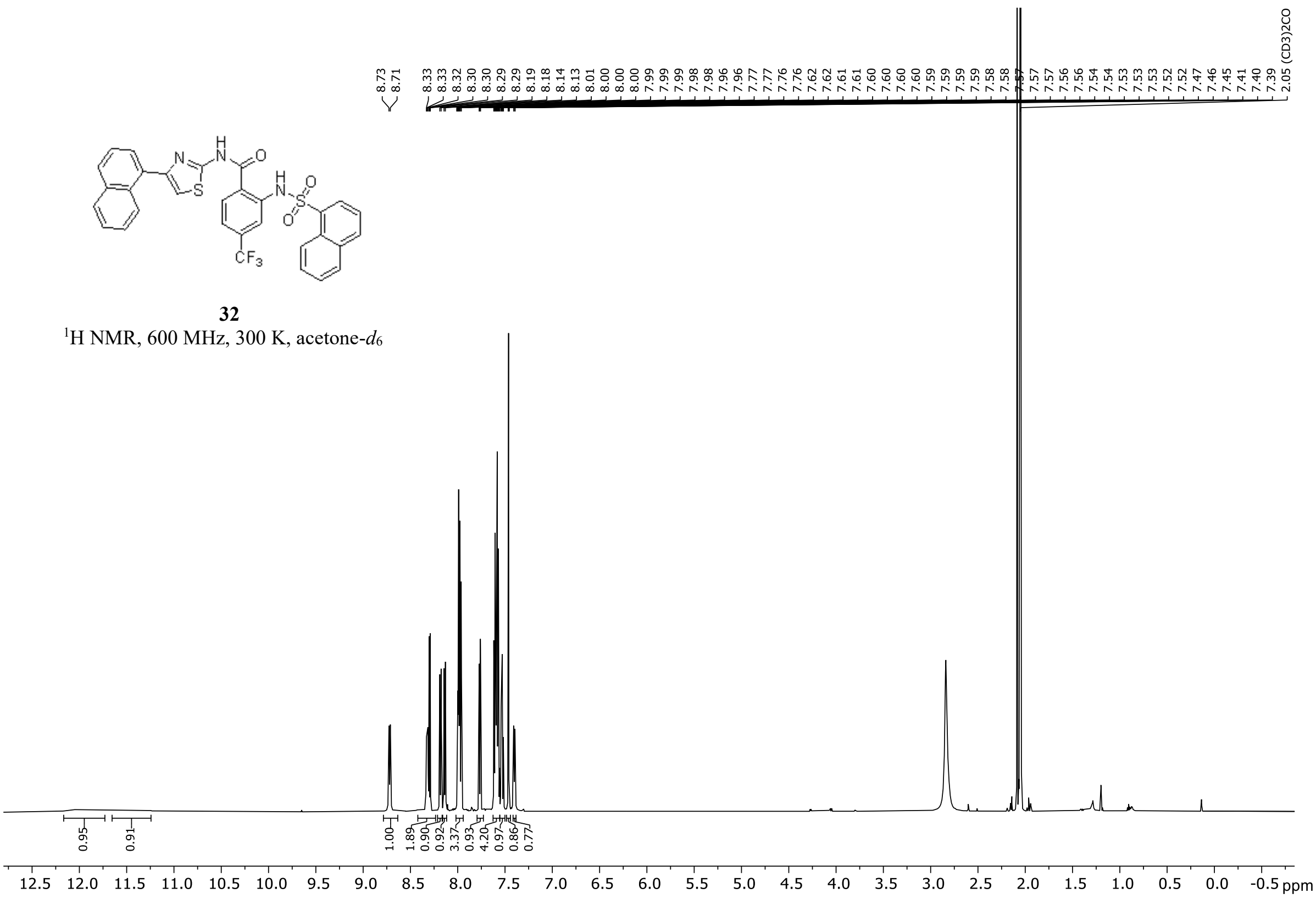

32

^{1}H NMR, 600 MHz, 300 K, acetone-d_6

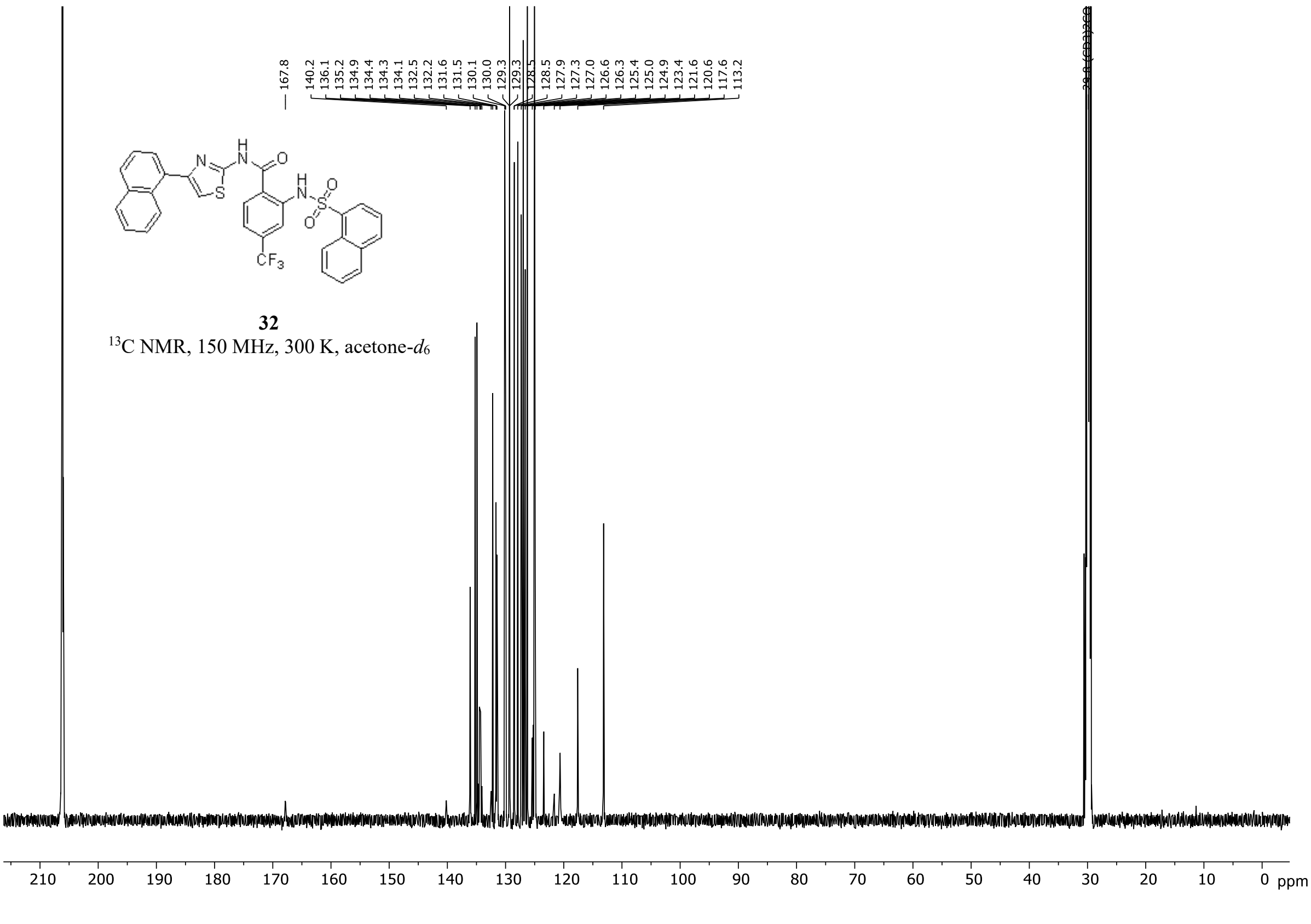

32

^{13}C NMR, 150 MHz, 300 K, acetone-d_6

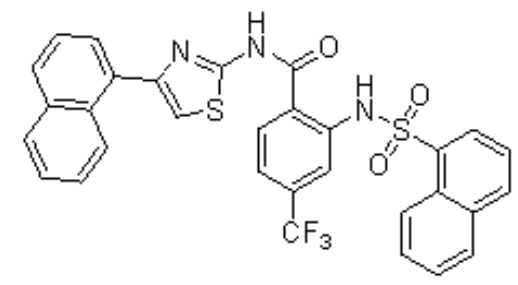

32

^{19}F NMR, 565 MHz, 300 K, acetone-d_6

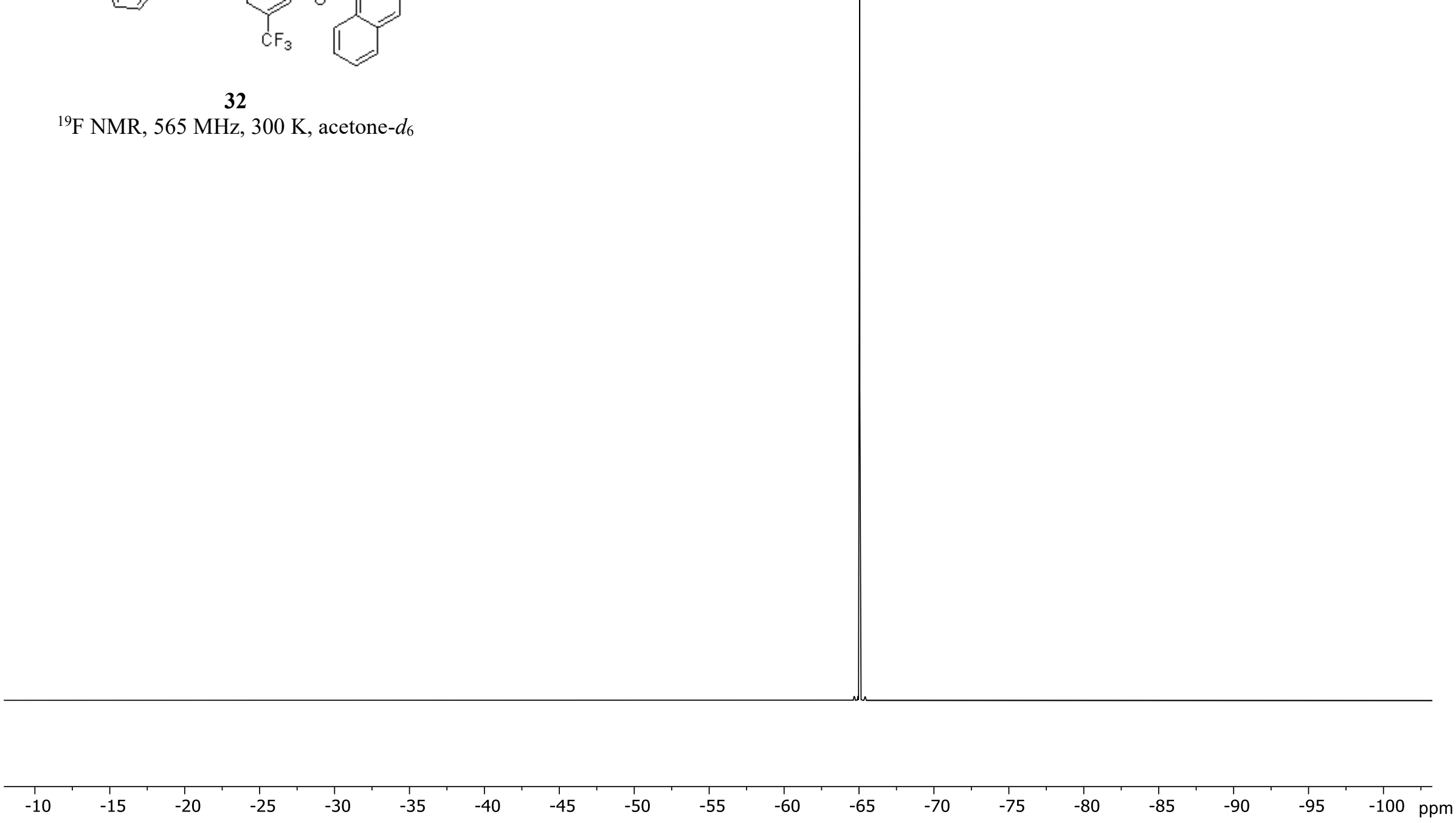

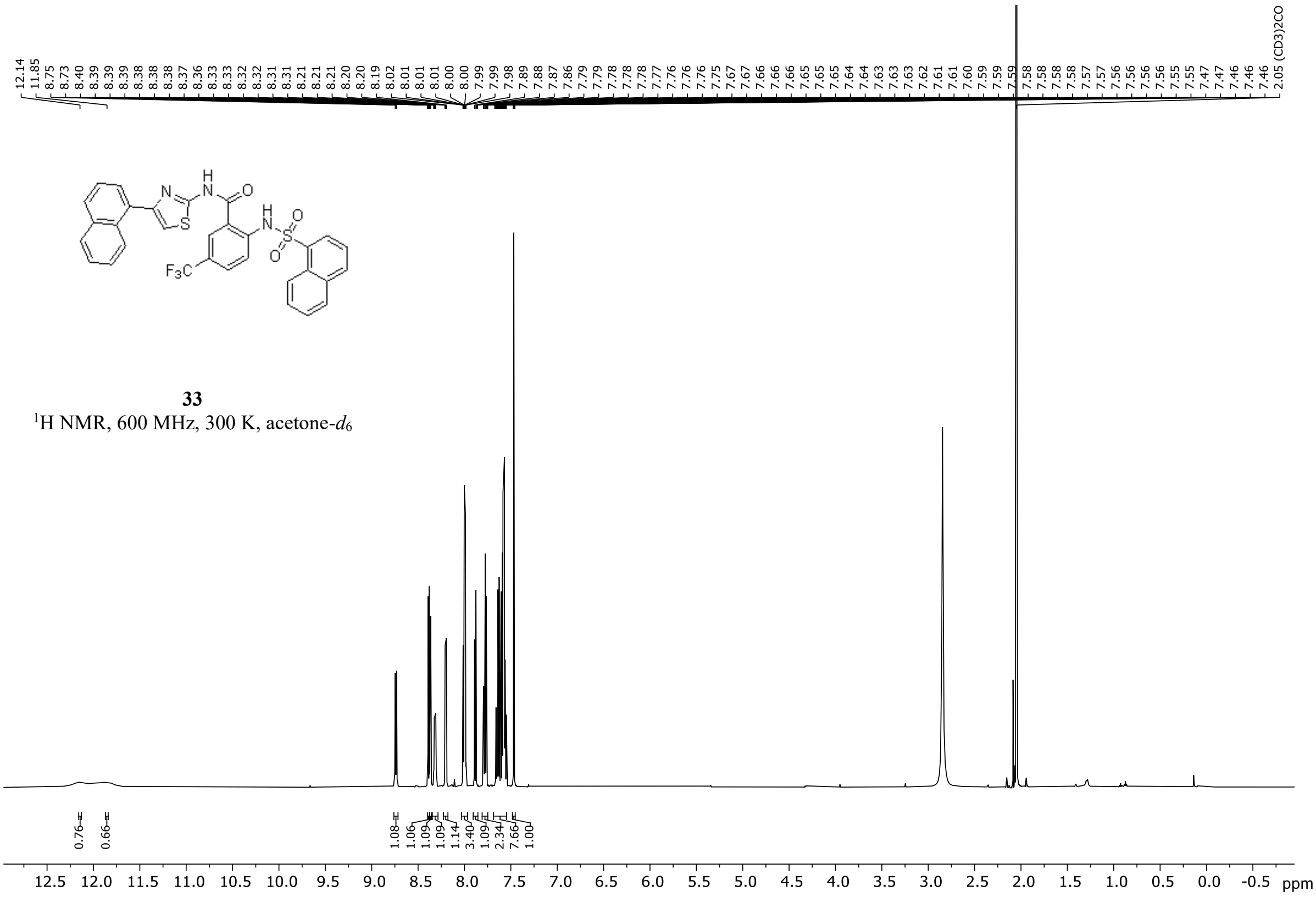

33

^{1}H NMR, 600 MHz, 300 K, acetone-d_6

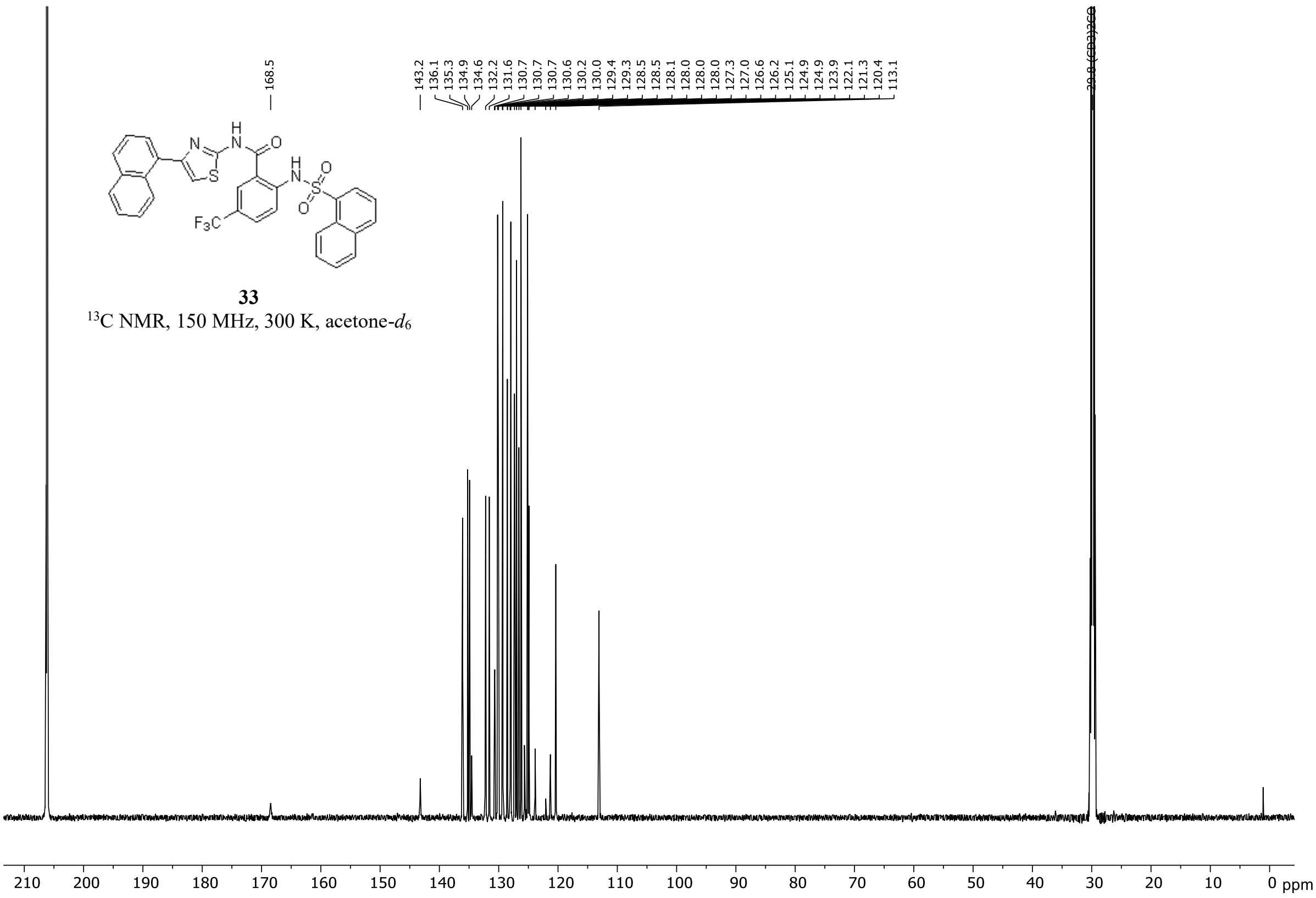

33

^{13}C NMR, 150 MHz, 300 K, acetone-d_6

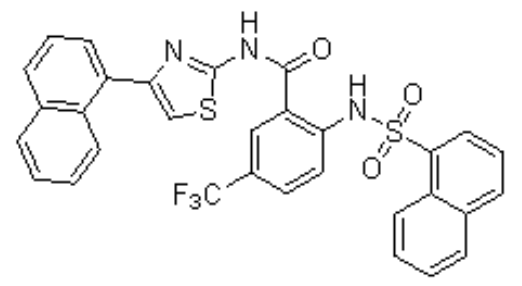

33

^{19}F NMR, 565 MHz, 300 K, acetone-d_6

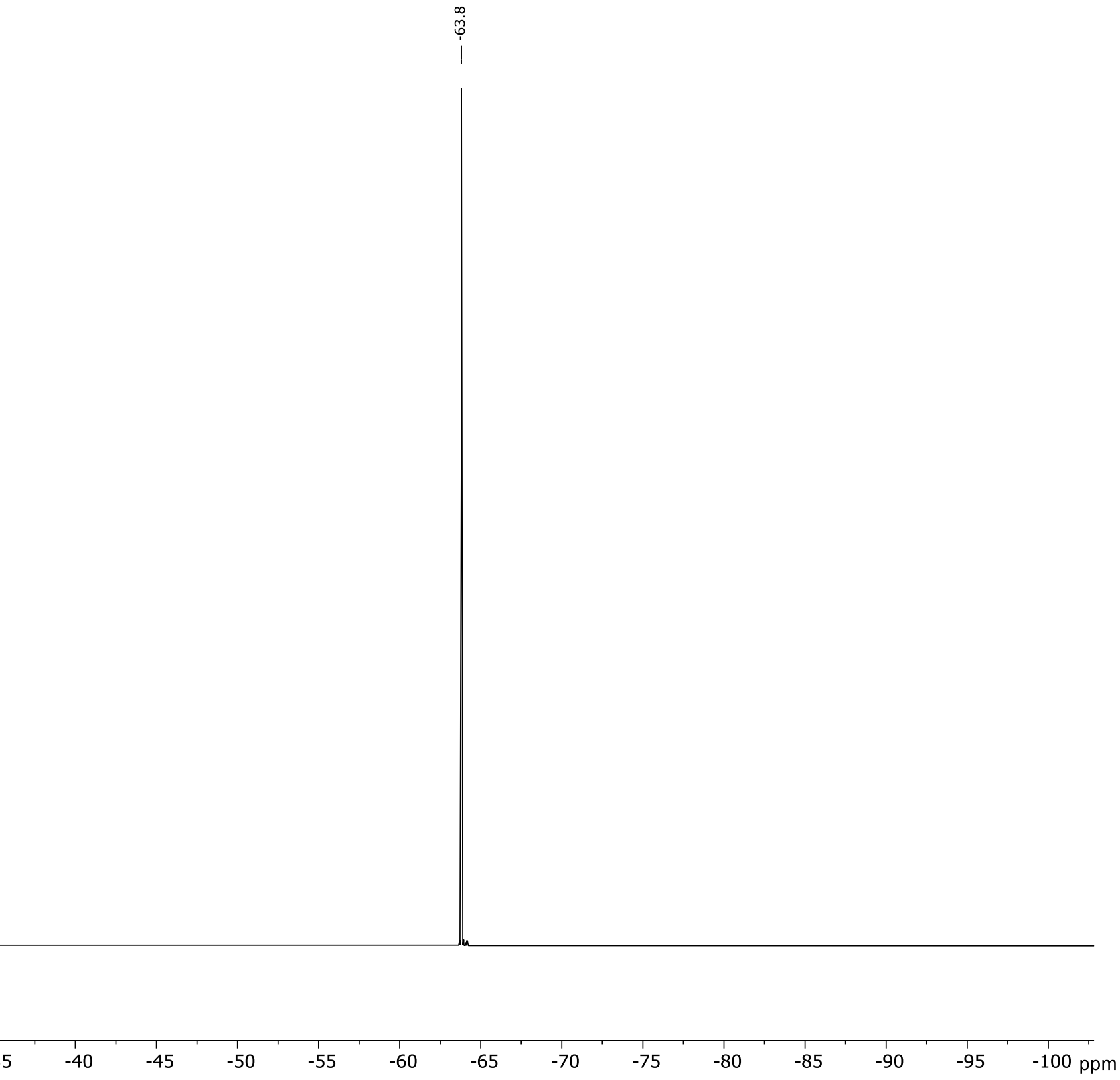

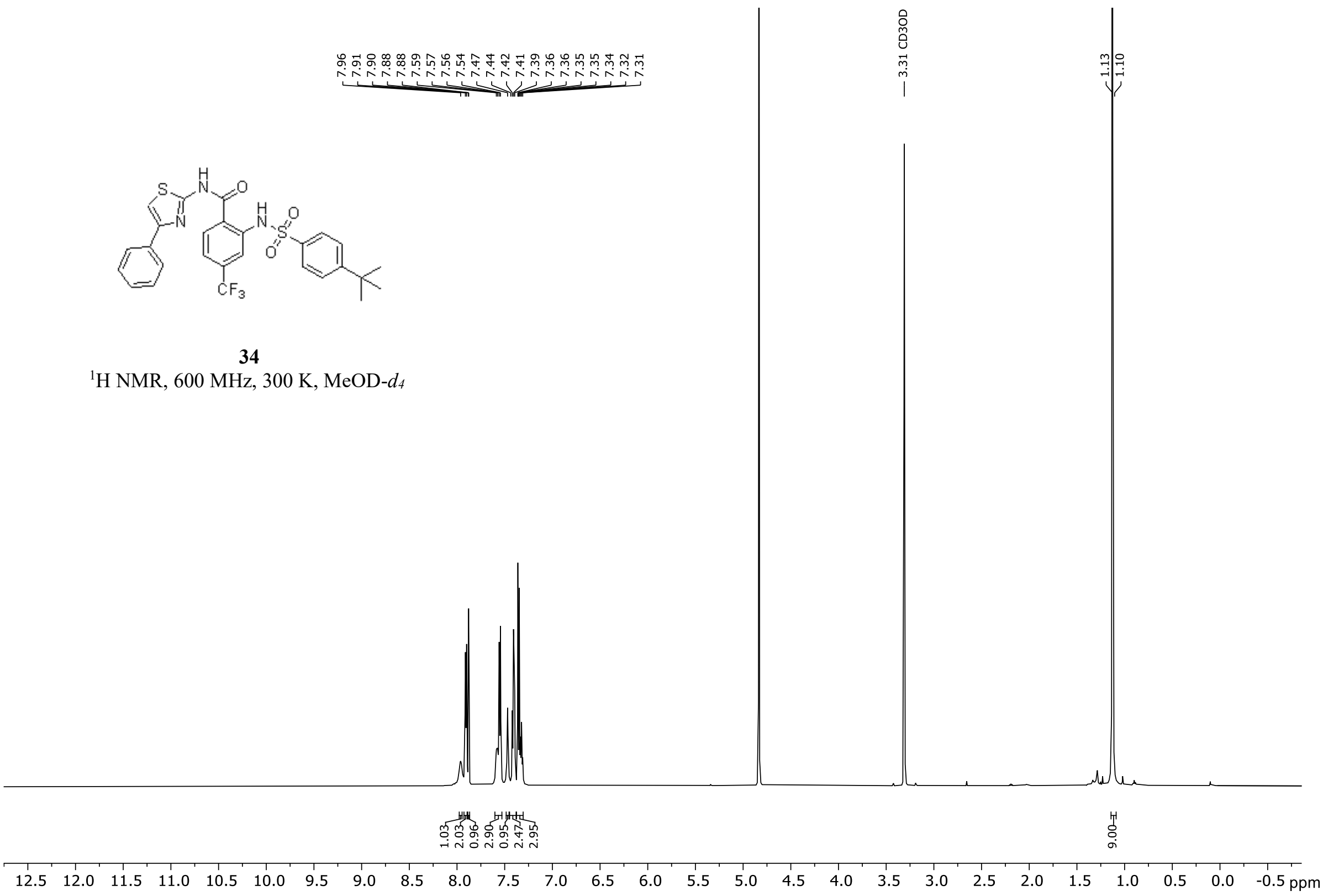

34

^{1}H NMR, 600 MHz, 300 K, MeOD-d_4

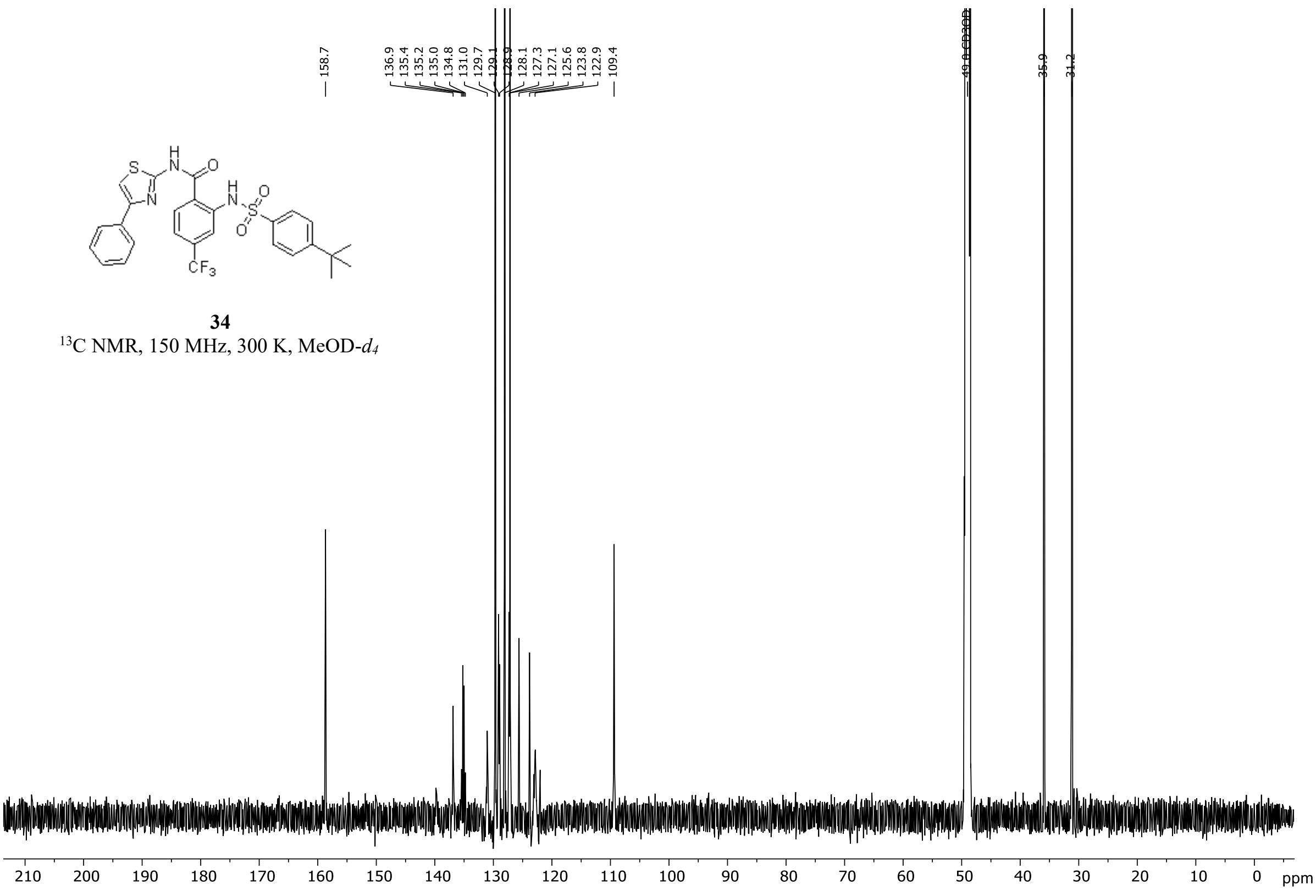

34

^{13}C NMR, 150 MHz, 300 K, MeOD-d_4

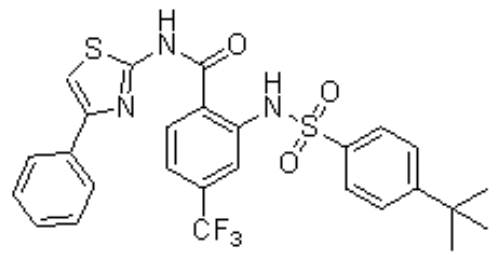

34
^{19}F NMR, 565 MHz, 300 K, MeOD-d_4

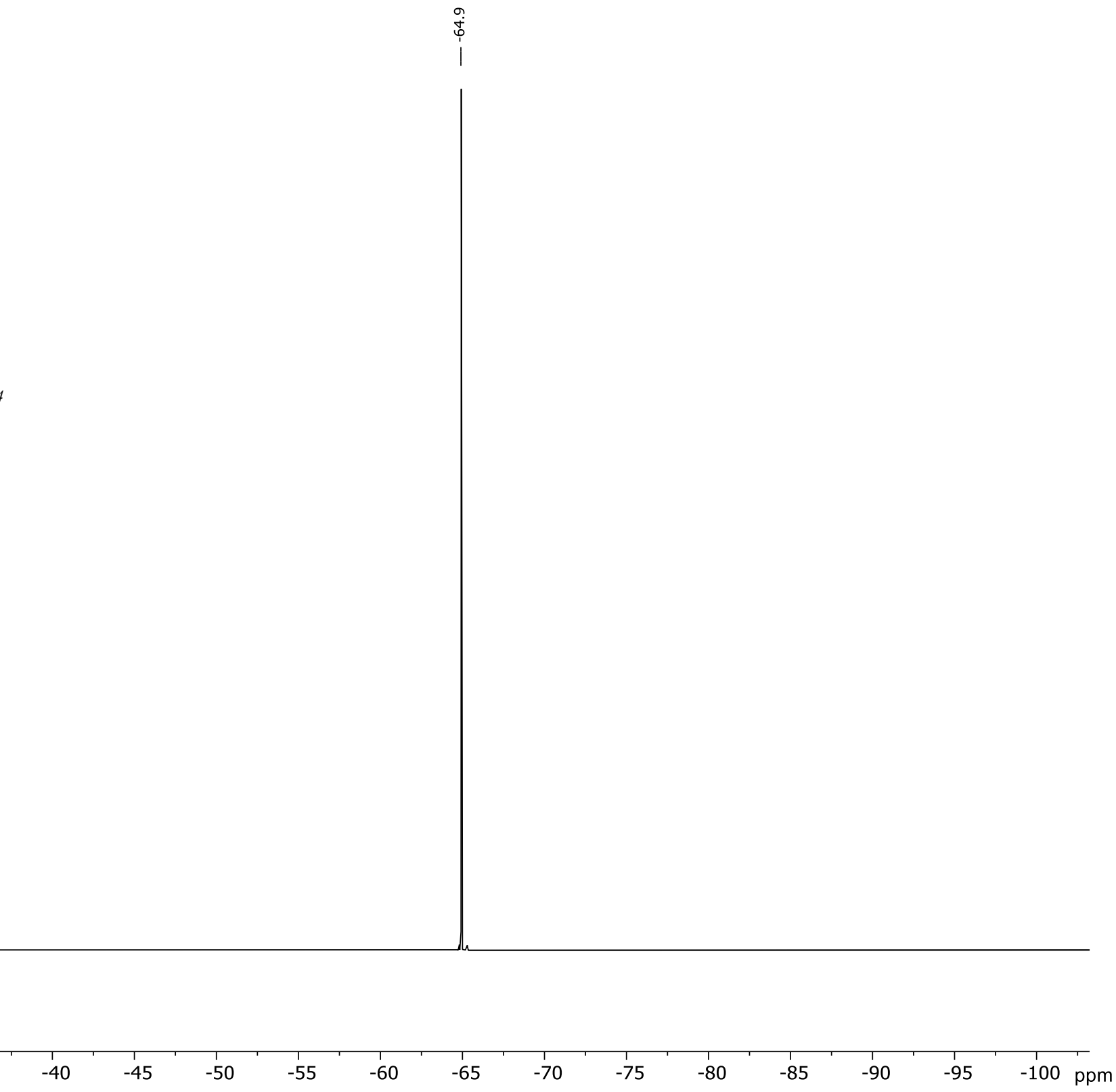

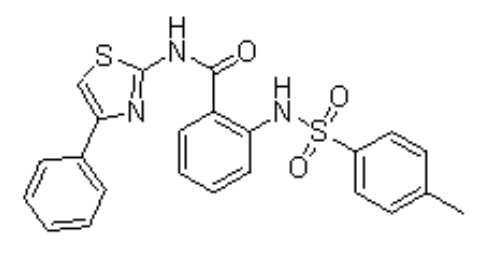

35
^{1}H NMR, 400 MHz, 300 K, MeOD-d_4

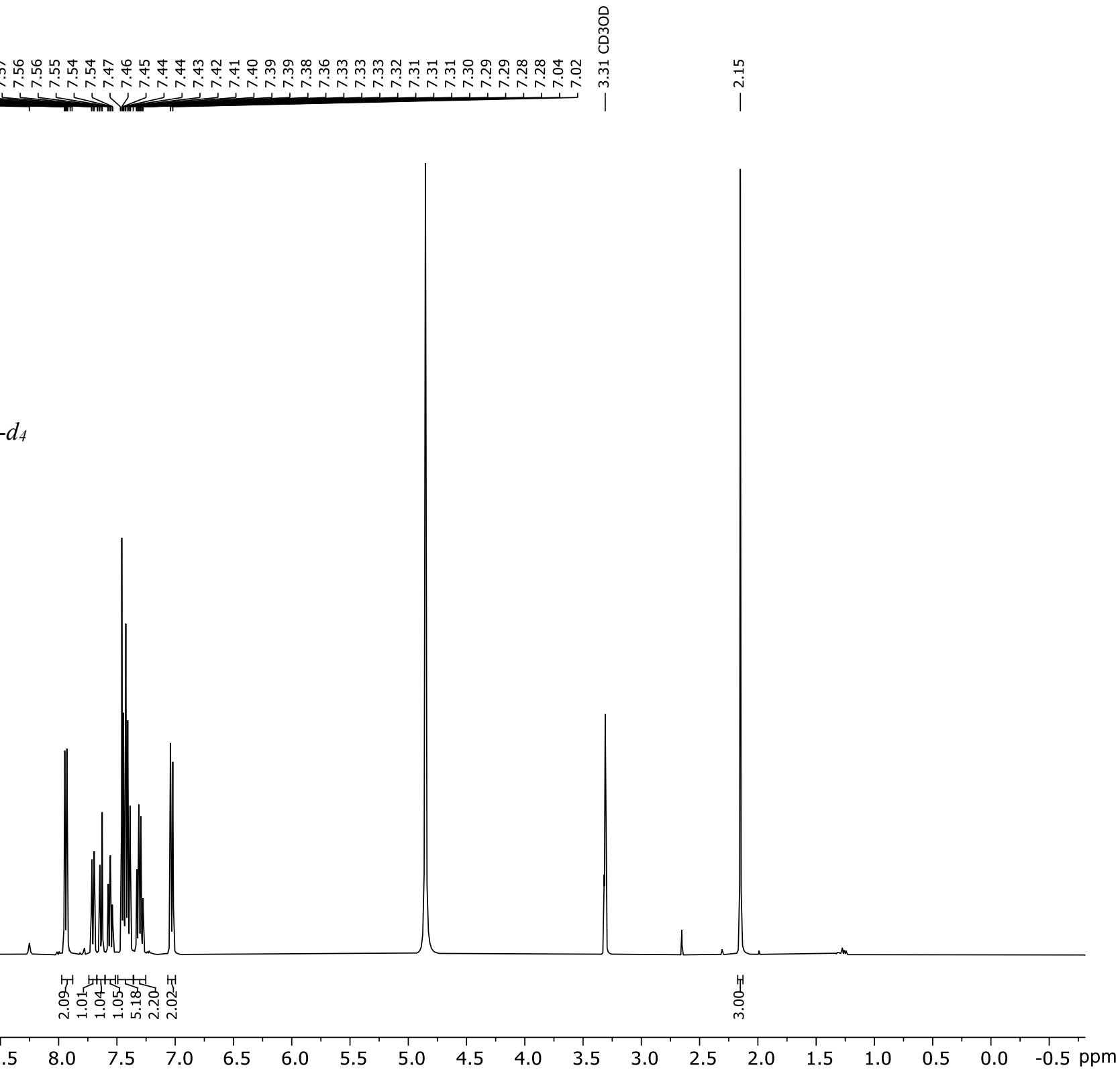

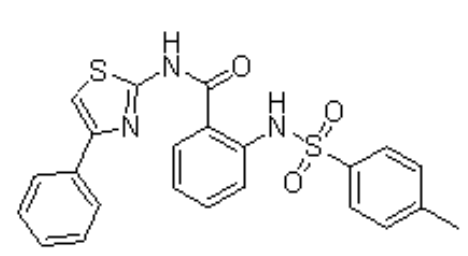

35
^{13}C NMR, 100 MHz, 300 K, MeOD-d_4

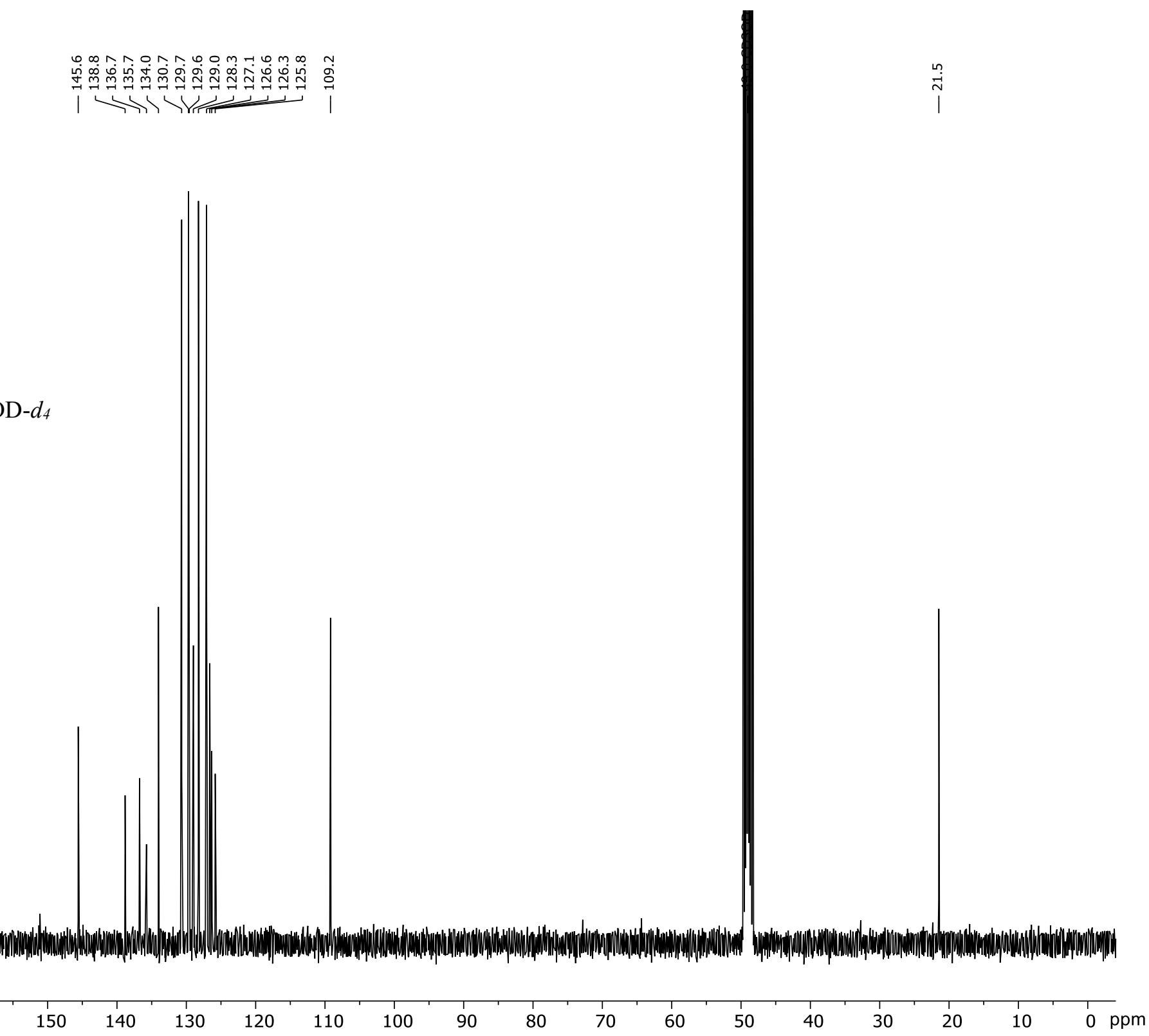

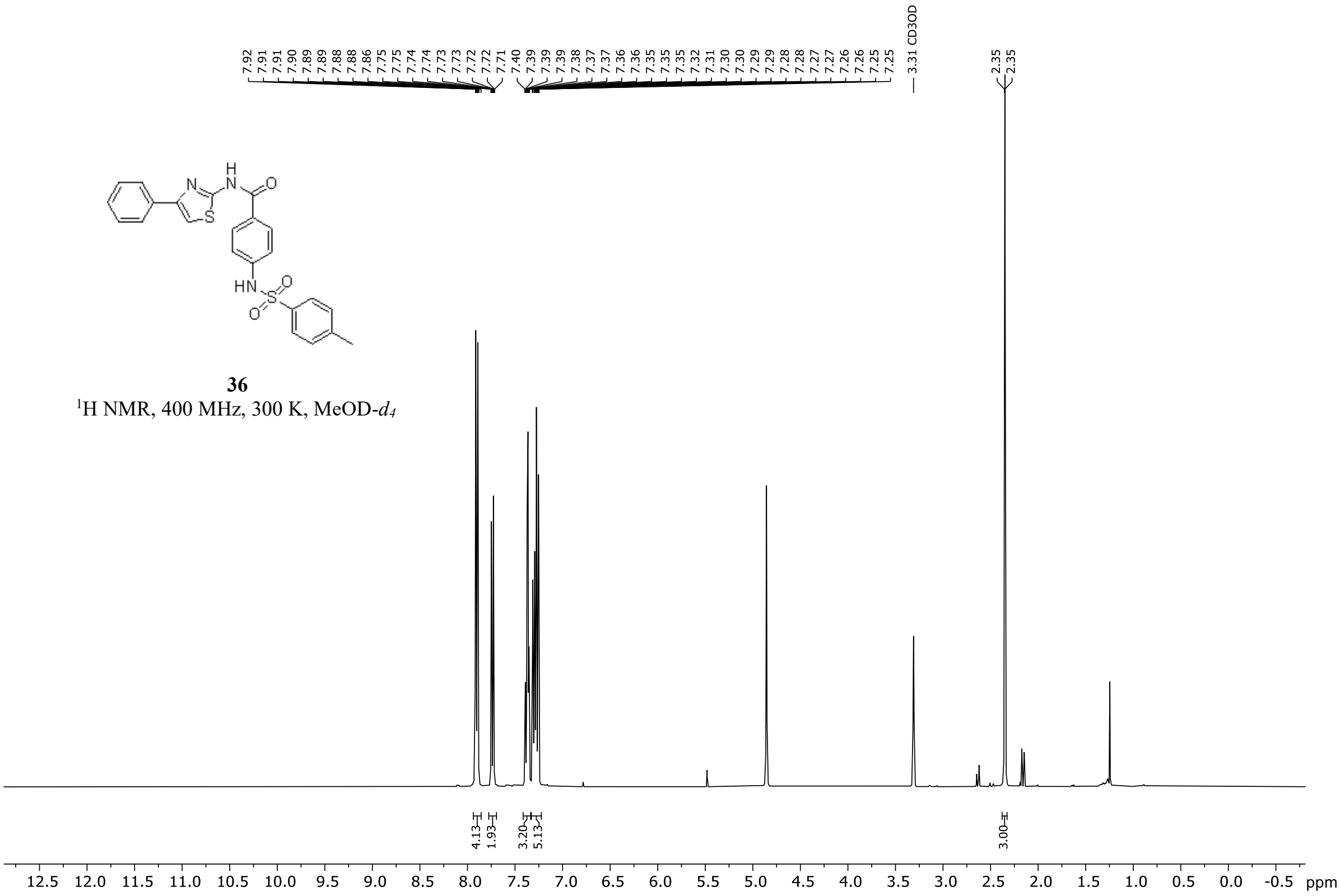

36

^{1}H NMR, 400 MHz, 300 K, MeOD-d_4

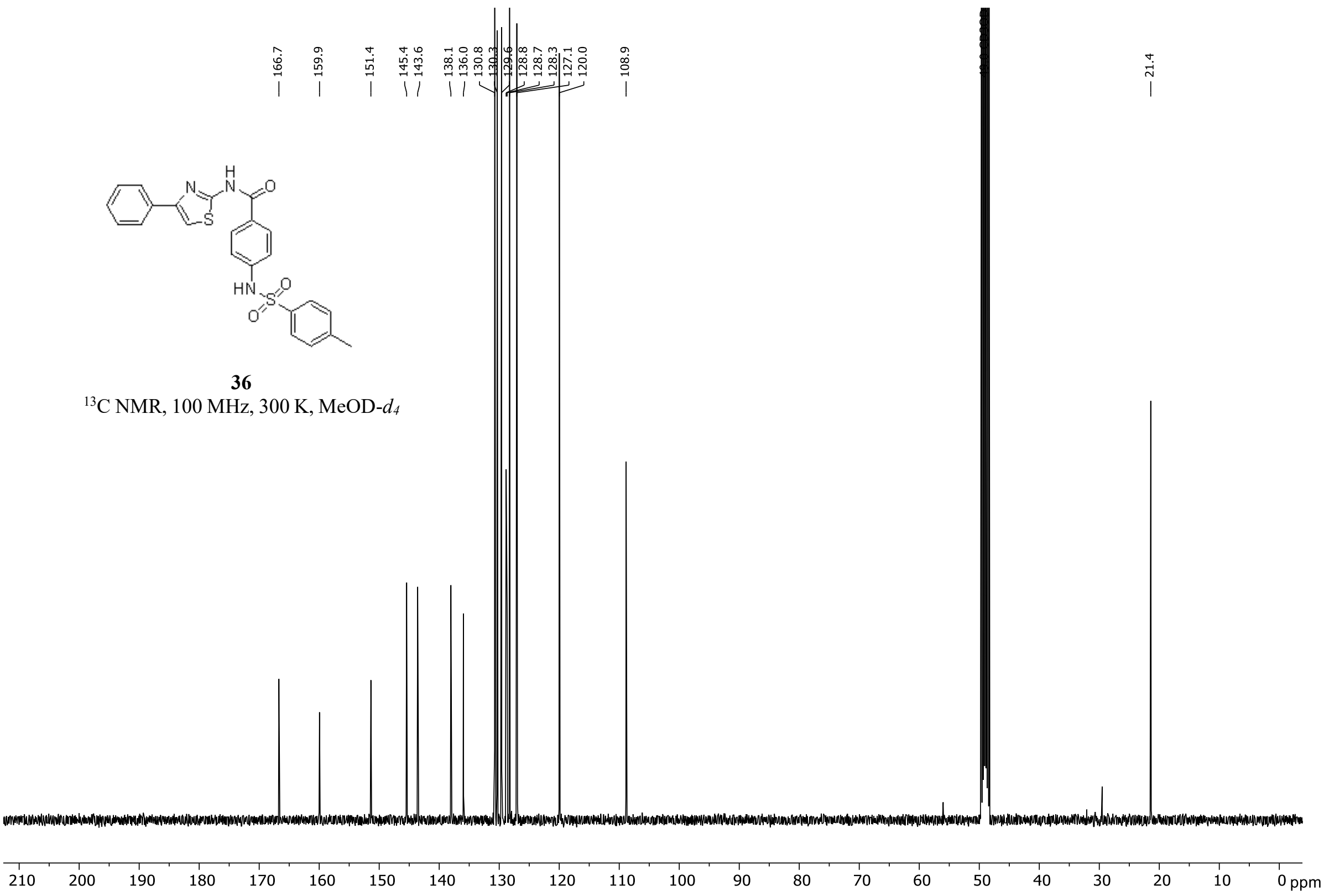

36

^{13}C NMR, 100 MHz, 300 K, MeOD-d_4

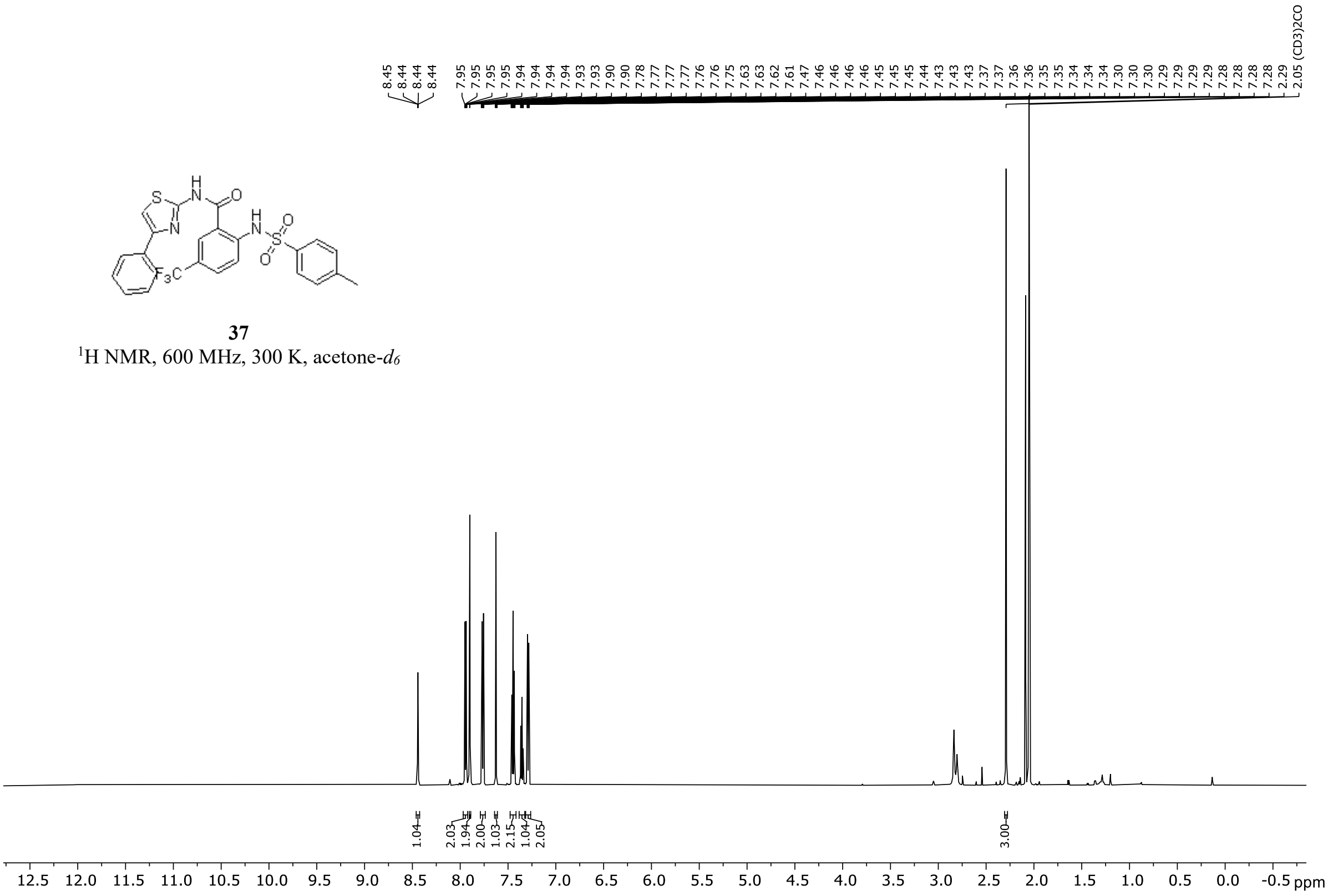

37

^{1}H NMR, 600 MHz, 300 K, acetone-d_6

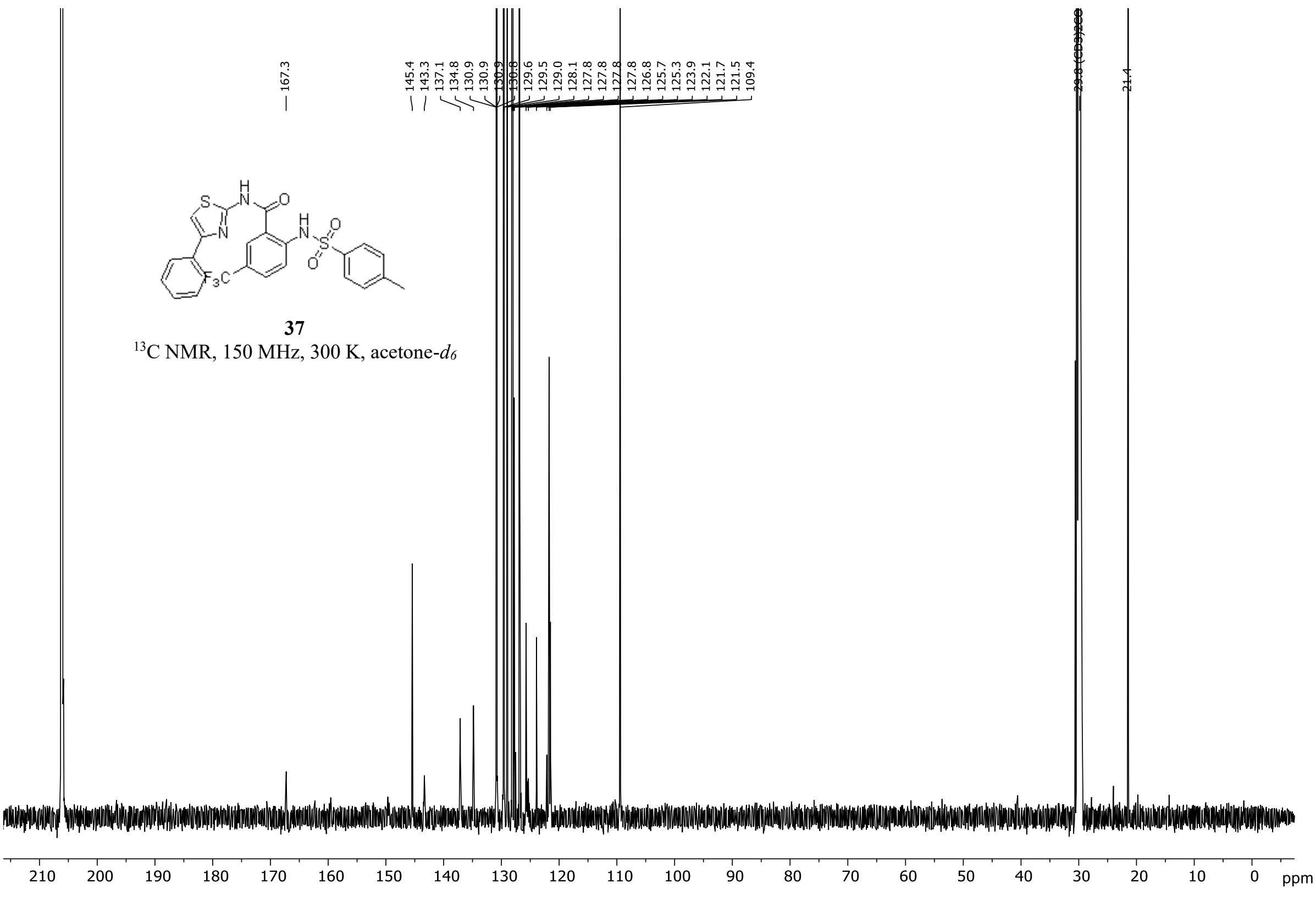

37

^{13}C NMR, 150 MHz, 300 K, acetone-d_6

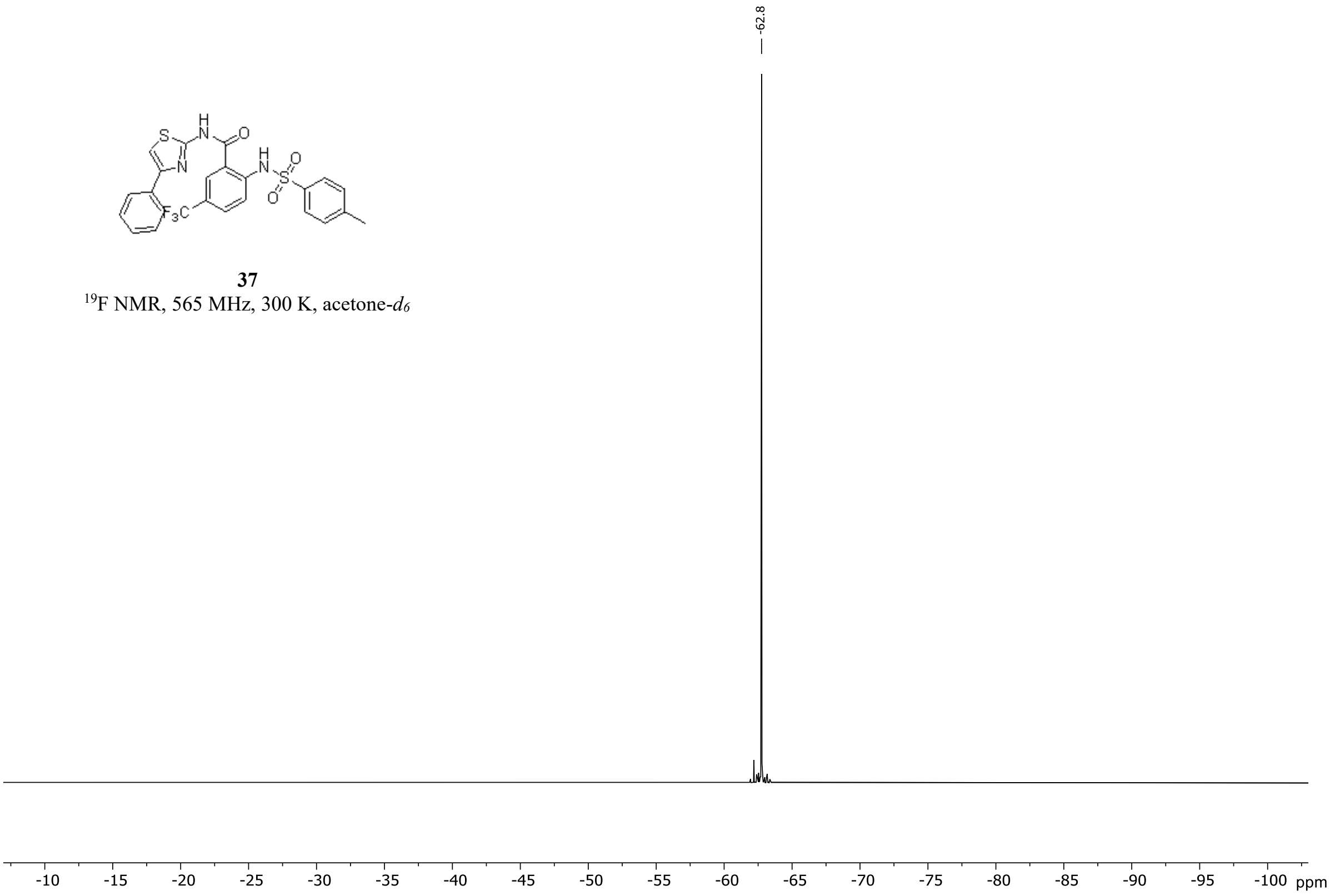

37

^{19}F NMR, 565 MHz, 300 K, acetone-d_6
